# Nations within a nation: variations in epidemiological transition across the states of India, 1990–2016 in the Global Burden of Disease Study

**DOI:** 10.1016/S0140-6736(17)32804-0

**Published:** 2017-12-02

**Authors:** India State-level Disease Burden Initiative Collaborators, Lalit Dandona, Rakhi Dandona, G Anil Kumar, D K Shukla, Vinod K Paul, Kalpana Balakrishnan, Dorairaj Prabhakaran, Nikhil Tandon, Sundeep Salvi, A P Dash, A Nandakumar, Vikram Patel, Sanjay K Agarwal, Prakash C Gupta, R S Dhaliwal, Prashant Mathur, Avula Laxmaiah, Preet K Dhillon, Subhojit Dey, Manu R Mathur, Ashkan Afshin, Christina Fitzmaurice, Emmanuela Gakidou, Peter Gething, Simon I Hay, Nicholas J Kassebaum, Hmwe Kyu, Stephen S Lim, Mohsen Naghavi, Gregory A Roth, Jeffrey D Stanaway, Harvey Whiteford, Vineet K Chadha, Sunil D Khaparde, Raghuram Rao, Kirankumar Rade, Puneet Dewan, Melissa Furtado, Eliza Dutta, Chris M Varghese, Ravi Mehrotra, P Jambulingam, Tanvir Kaur, Meenakshi Sharma, Shalini Singh, Rashmi Arora, Reeta Rasaily, Ranjit M Anjana, Viswanathan Mohan, Anurag Agrawal, Arvind Chopra, Ashish J Mathew, Deeksha Bhardwaj, Pallavi Muraleedharan, Parul Mutreja, Kelly Bienhoff, Scott Glenn, Rizwan S Abdulkader, Ashutosh N Aggarwal, Rakesh Aggarwal, Sandra Albert, Atul Ambekar, Monika Arora, Damodar Bachani, Ashish Bavdekar, Gufran Beig, Anil Bhansali, Anurag Bhargava, Eesh Bhatia, Bilali Camara, D J Christopher, Siddharth K Das, Paresh V Dave, Sagnik Dey, Aloke G Ghoshal, N Gopalakrishnan, Randeep Guleria, Rajeev Gupta, Subodh S Gupta, Tarun Gupta, M D Gupte, G Gururaj, Sivadasanpillai Harikrishnan, Veena Iyer, Sudhir K Jain, Panniyamamkal Jeemon, Vasna Joshua, Rajni Kant, Anita Kar, Amal C Kataki, Kiran Katoch, Tripti Khanna, Ajay Khera, Sanjay Kinra, Parvaiz A Koul, Anand Krishnan, Avdhesh Kumar, Raman K Kumar, Rashmi Kumar, Anura Kurpad, Laishram Ladusingh, Rakesh Lodha, P A Mahesh, Rajesh Malhotra, Matthews Mathai, Dileep Mavalankar, Murali Mohan BV, Satinath Mukhopadhyay, Manoj Murhekar, G V S Murthy, Sanjeev Nair, Sreenivas A Nair, Lipika Nanda, Romi S Nongmaithem, Anu M Oommen, Jeyaraj D Pandian, Sapan Pandya, Sreejith Parameswaran, Sanghamitra Pati, Kameshwar Prasad, Narayan Prasad, Manorama Purwar, Asma Rahim, Sreebhushan Raju, Siddarth Ramji, Thara Rangaswamy, Goura K Rath, Ambuj Roy, Yogesh Sabde, K S Sachdeva, Harsiddha Sadhu, Rajesh Sagar, Mari J Sankar, Rajendra Sharma, Anita Shet, Shreya Shirude, Rajan Shukla, Sharvari R Shukla, Gagandeep Singh, Narinder P Singh, Virendra Singh, Anju Sinha, Dhirendra N Sinha, R K Srivastava, A Srividya, Vanita Suri, Rajaraman Swaminathan, P N Sylaja, Babasaheb Tandale, J S Thakur, Kavumpurathu R Thankappan, Nihal Thomas, Srikanth Tripathy, Mathew Varghese, Santosh Varughese, S Venkatesh, K Venugopal, Lakshmi Vijayakumar, Denis Xavier, Chittaranjan S Yajnik, Geevar Zachariah, Sanjay Zodpey, J V R Prasada Rao, Theo Vos, K Srinath Reddy, Christopher J L Murray, Soumya Swaminathan

## Abstract

**Background:**

18% of the world's population lives in India, and many states of India have populations similar to those of large countries. Action to effectively improve population health in India requires availability of reliable and comprehensive state-level estimates of disease burden and risk factors over time. Such comprehensive estimates have not been available so far for all major diseases and risk factors. Thus, we aimed to estimate the disease burden and risk factors in every state of India as part of the Global Burden of Disease (GBD) Study 2016.

**Methods:**

Using all available data sources, the India State-level Disease Burden Initiative estimated burden (metrics were deaths, disability-adjusted life-years [DALYs], prevalence, incidence, and life expectancy) from 333 disease conditions and injuries and 84 risk factors for each state of India from 1990 to 2016 as part of GBD 2016. We divided the states of India into four epidemiological transition level (ETL) groups on the basis of the ratio of DALYs from communicable, maternal, neonatal, and nutritional diseases (CMNNDs) to those from non-communicable diseases (NCDs) and injuries combined in 2016. We assessed variations in the burden of diseases and risk factors between ETL state groups and between states to inform a more specific health-system response in the states and for India as a whole.

**Findings:**

DALYs due to NCDs and injuries exceeded those due to CMNNDs in 2003 for India, but this transition had a range of 24 years for the four ETL state groups. The age-standardised DALY rate dropped by 36·2% in India from 1990 to 2016. The numbers of DALYs and DALY rates dropped substantially for most CMNNDs between 1990 and 2016 across all ETL groups, but rates of reduction for CMNNDs were slowest in the low ETL state group. By contrast, numbers of DALYs increased substantially for NCDs in all ETL state groups, and increased significantly for injuries in all ETL state groups except the highest. The all-age prevalence of most leading NCDs increased substantially in India from 1990 to 2016, and a modest decrease was recorded in the age-standardised NCD DALY rates. The major risk factors for NCDs, including high systolic blood pressure, high fasting plasma glucose, high total cholesterol, and high body-mass index, increased from 1990 to 2016, with generally higher levels in higher ETL states; ambient air pollution also increased and was highest in the low ETL group. The incidence rate of the leading causes of injuries also increased from 1990 to 2016. The five leading individual causes of DALYs in India in 2016 were ischaemic heart disease, chronic obstructive pulmonary disease, diarrhoeal diseases, lower respiratory infections, and cerebrovascular disease; and the five leading risk factors for DALYs in 2016 were child and maternal malnutrition, air pollution, dietary risks, high systolic blood pressure, and high fasting plasma glucose. Behind these broad trends many variations existed between the ETL state groups and between states within the ETL groups. Of the ten leading causes of disease burden in India in 2016, five causes had at least a five-times difference between the highest and lowest state-specific DALY rates for individual causes.

**Interpretation:**

Per capita disease burden measured as DALY rate has dropped by about a third in India over the past 26 years. However, the magnitude and causes of disease burden and the risk factors vary greatly between the states. The change to dominance of NCDs and injuries over CMNNDs occurred about a quarter century apart in the four ETL state groups. Nevertheless, the burden of some of the leading CMNNDs continues to be very high, especially in the lowest ETL states. This comprehensive mapping of inequalities in disease burden and its causes across the states of India can be a crucial input for more specific health planning for each state as is envisioned by the Government of India's premier think tank, the National Institution for Transforming India, and the National Health Policy 2017.

**Funding:**

Bill & Melinda Gates Foundation; Indian Council of Medical Research, Department of Health Research, Ministry of Health and Family Welfare, Government of India; and World Bank

## Introduction

India has a population of 1·34 billion spread across 29 states and seven union territories. Many of the states have populations of similar sizes to large countries; ten states had more than 60 million people in 2017.^1^ The largest state, Uttar Pradesh, with a population of more than 220 million people, ranked fifth largest among all nations of the world.^2^ India has more than 2000 ethnic groups with genetically distinct ancestry and diverse lifestyles^3^ and has undergone heterogeneous economic growth over the past few decades, which would be expected to lead to wide variations in health and disease distribution in different parts of the country. The overall economic growth rate in India has been one the fastest in the world in the past decade.^4^ This growth should be used to enhance major long-term enablers of societal development, of which population health is a crucial aspect that would further boost economic growth.

**Research in context**

**Evidence before this study**

Existing evidence suggests that India has been going through an epidemiological transition with an increase in the proportion of disease burden attributable to non-communicable diseases (NCDs). Attempts have been made to understand the epidemiological transition of India as a whole for its population of 1·3 billion people. The burden from major communicable diseases such as diarrhoea, lower respiratory infections, and tuberculosis, as well as neonatal disorders, continues to be quite high in India relative to other countries. State-level estimates of key indicators such as neonatal, infant, and under-5 mortality rates are provided by the Sample Registration Survey of India annually for the states of the country. The Sample Registration Survey has also reported causes of death from verbal autopsy for aggregate causes by regions of the country. State-level estimates for HIV are produced by the National AIDS Control Organization of India. The major national surveys, the National Family Health Survey, District Level Household Survey, and the Annual Health Survey have provided valuable periodic data on key health indicators, though mostly related to child and reproductive health. Data on the prevalence of diabetes, ischaemic heart disease, and their risk factors are increasingly being generated for a number of states by several studies. However, a comprehensive assessment of all major diseases and risk factors across all states of India providing estimates over an extended period of time, which is needed for an informed health-system and policy development in each state, has not previously been published to our knowledge.

**Added value of this study**

For the first time to our knowledge, this study provides estimates of 333 disease conditions and injuries and 84 risk factors for every state of India from 1990 to 2016, using all available data identified through an extensive effort involving over 200 leading health scientists and policy makers in India from 103 institutions. The generation of estimates and their interpretation have benefited from the insights of domain experts through an intensive collaborative process over 2 years. The findings from this study have enabled a comprehensive mapping of the epidemiological transition in each state of India, which has revealed that grouping the states into four groups by different epidemiological transition levels is a useful intermediate step in understanding disease burden and risk factor trends across the country. The specific state-level findings presented to some extent in this paper, and in more detail with a profile of each state in the policy report being presented to the Government of India and the state governments, are crucial valuable additions to state-specific health policy making in India.

**Implications of all the available evidence**

The evidence now explicitly describes the extent of epidemiological transition, burden of broad disease groups and specific diseases, and risk factors in each state of India and in the four state groups by epidemiological transition level. Although the burden due to NCDs and injuries as a whole has overtaken the burden due to communicable, maternal, neonatal, and nutritional disorders (CMNNDs) in every state of the country, the extent of this varies widely. Accordingly, the enhancement of interventions to control NCDs and injuries must happen in every state of the country, but, in parallel to this, the burden of CMNNDs has to be addressed with vigour, commensurate with its magnitude in each state. This specific titration of health policy will be key to achieving an appropriate balance of interventions needed to reduce the vast inequalities in health status among the states of India.

Some research has tried to understand the epidemiological transition that India is undergoing,^5^, ^6^, ^7^, ^8^, ^9^ but a comprehensive understanding of the changes in disease burden and risk factor trends with large-scale robust data for each state of India is not readily available. The social development status of the states in India varies widely. For example, the state of Kerala has been reported to have had much better health indicators than the rest of India for the past several decades.^10^ The Government of India focuses more development efforts on the Empowered Action Group (EAG) states in north India and the states of the northeast region of India, which often have poorer health indicators than the rest of India.^11^ Diversity in the magnitude and causes of disease burden, as well as the risk factors, is generally anticipated between and within the broad state groupings, but no systematic and comprehensive analysis of the state-level variations for these is available to inform specific state-level planning. Although the central government policies have significant influence on health initiatives across the country, health is a state subject in the Indian federal structure.^12^ Of total government spending on health at the state level, on average two-thirds is from the state budget and one-third from the central budget.^13^ A robust disaggregated understanding of the disease burden and risk factors trends in each state of India is essential for effective health-system and policy action to improve population health.

The India State-level Disease Burden Initiative was launched in October, 2015, to address this crucial gap that hinders informed health-system and policy development commensurate with the state-level variations in diseases and risk factors. This initiative is a collaboration involving 103 institutions with the aim of producing robust state-level disease burden trends from 1990 onward as part of the Global Burden of Disease Study (GBD), using all identifiable epidemiological data from India and the expertise of a large number of leading health scientists and thinkers in India. More information on this initiative is provided in the appendix (p 4). This effort is consistent with the recent inclusion of disease burden tracking using disability-adjusted life-years (DALYs) as a specific objective in the India National Health Policy 2017, and the emphasis by the National Institution for Transforming India (NITI Aayog; the premier thinktank of the Government of India) on developing robust systems for disaggregated data to inform policy, indicating high-level policy interest in using reliable disease burden estimation to guide improvements in population health.^14^, ^15^, ^16^, ^17^

In this paper we report findings from the first comprehensive assessment by the India State-level Disease Burden Initiative produced as part of GBD 2016, highlighting that the country is in different phases of epidemiological transition, which have resulted in massive variations in disease burden across the Indian states. This has fundamental implications for state-specific health-system and policy efforts to improve the health of the 18% of the world's population that lives in India.

## Methods

### Overview

The network of the India State-level Disease Burden Initiative collaborators worked closely on the data sources, analyses, and interpretation of the findings for the calculation of state-level disease burden and risk factor estimates as part of GBD 2016. This collaborative work benefitted immensely from the deliberations of the 14 expert groups formed under the India State-level Disease Burden Initiative. The work of this initiative is approved by the Health Ministry Screening Committee of the Indian Council of Medical Research, and by the ethics committee of the Public Health Foundation of India.

A comprehensive description of data sources, data quality, statistical modelling and analyses, and metrics for GBD 2016 have been reported elsewhere.^18^, ^19^, ^20^, ^21^, ^22^ GBD 2016 estimated disease burden due to 333 diseases and injuries (appendix pp 5–11) and 84 risk factors (appendix pp 12–13). The GBD cause list is hierarchical and includes three broad categories at the top level: communicable, maternal, neonatal, and nutritional diseases (CMNNDs); non-communicable diseases (NCDs); and injuries.^19^, ^21^

The findings in this paper are presented for 31 geographical units in India: 29 states, Union Territory of Delhi, and the union territories other than Delhi (combining the six smaller union territories of Andaman and Nicobar Islands, Chandigarh, Dadra and Nagar Haveli, Daman and Diu, Lakshadweep, and Puducherry). The states of Chhattisgarh, Uttarakhand, and Jharkhand were created from existing larger states in 2000, and the state of Telangana was created in 2014. For trends from 1990 onward, the data for these four new states were disaggregated from their parent states on the basis of data from the districts that now constitute these states.

### Mortality, causes of death, and YLLs

The estimation process of all-cause mortality includes estimation of under-5 mortality, adult mortality, age–sex mortality estimation, adjustment for HIV/AIDS mortality, and the effects of fatal discontinuities such as wars, disasters, and pandemics. Life expectancy was computed at birth and at each age category for India and for states by age, sex, and time period. The major data sources for estimation of mortality in India include sample registration system (SRS) and vital registration, censuses, and large-scale national household surveys such as the National Family Health Surveys and District Level Household Surveys (appendix pp 14–121).

Causes of death were estimated on the basis of the GBD cause list using Cause of Death Ensemble model (CODEm), negative binomial models for rare causes, natural history models, subcause proportion models, and prevalence-based models. To generate the cause-of-death estimates, the completeness of death records was assessed by dividing registered deaths in each location–year by all-age death estimates and using statistical models. We mapped revisions of the International Classification of Diseases into a consistent classification for causes of deaths, and redistributed deaths assigned to causes that were not underlying causes of death (garbage codes) to specific underlying causes proportionately or using regression models. The sum of the predicted deaths from these models in an age-sex-state-year group do not necessarily equal the number of deaths from all causes in the mortality envelopes, and hence we made these consistent with the results from all-cause mortality estimation using the CoDCorrect algorithm.^19^ The data sources used for the causes of deaths estimation in India were verbal autopsy from SRS, Medically Certified Causes of Deaths, cancer registries, and smaller verbal autopsy studies (appendix pp 14–121). We obtained the years of life lost (YLLs) because of premature death by multiplying each death by the normative standard life expectancy at each age.^19^

The verbal autopsy cause of death data for 455 460 deaths covered by SRS from 2004 to 2013 across all states and union territories of India was a major additional data source for GBD 2016. The SRS in India is operated by the Office of the Registrar General of India working under the Ministry of Home Affairs, Government of India.^23^ Using the 2001 census, 7597 geographic units, 4433 (58·4%) of which were rural, were sampled for the 2004–13 SRS to represent the population of each state and union territory of India, ultimately with a sample of 6·7 million people that was equivalent to 0·7% of India's population. The SRS cause-of-death data for 2004–06, 2007–09, and 2010–13 were provided for each state and union territory by the Office of the Registrar General of India for use in the state-level disease burden estimation. We used 2005, 2008, and 2012 as midpoint years for these three time periods. The inclusion of SRS 2004–13 data in this analysis offers a comprehensive picture of causes of death in India. The Office of the Registrar General of India was not involved with the production of the GBD modelled estimates, and therefore their estimates might differ from those presented here.

### YLDs and DALYs

We estimated non-fatal health outcomes mostly using DisMod-MR, version 2.1, an updated Bayesian-regression analytic tool, to synthesise consistent estimates of disease incidence, prevalence, remission, excess mortality, and cause-specific mortality rates. Details of this and other estimation methods, severity distributions and disability weights, which are used to quantify the relative severity of GBD causes, including the sources used for India, are published elsewhere.^20^

The major input data sources used to quantify the non-fatal burden of disease in India were representative population-level surveys and cohort studies, programme-level data on disease burden from government agencies, surveillance system data on disease burden, administrative records of health-service encounters, disease registries, and a wide range of other studies done across India (appendix pp 14–121). These studies included published literature as well as unpublished studies that were identified and accessed through a network of expert group members and collaborators in India.

Years lived with disability (YLDs) are calculated by multiplying the prevalence of each sequela by its disability weight, developed using population-based surveys.^20^ The computation of YLDs involved the estimation of prevalence of disease, injuries, and their sequelae. To compute YLDs for the particular sequela, the prevalence of each sequela was multiplied by the disability weight for the corresponding health state.^20^ The sum of all YLDs for relevant sequelae equated to overall YLDs for each disease, because sequelae in GBD are mutually exclusive and collectively exhaustive. We computed DALYs for India and states by summing YLLs and YLDs for each cause, age, and sex. For some causes that had reduction in DALY rates over time, we also assessed the change in their prevalence or incidence rates over time to understand whether the lower DALY rates were due to improving health care or decreases in these prevalence or incidence rates.

### Risk factors

A detailed description of comparative risk assessment for exposures and estimation of attributable risks and the GBD 2016 risk factor hierarchy is available elsewhere.^22^ To calculate risk-attributable fractions of disease burden by cause, we modelled the effects of risk exposure levels, documented relative risks associated with risk exposure and specific health outcomes, and computed theoretical minimum risk counterfactual levels of risk exposure on estimates for India and state-level deaths, YLLs, YLDs, and DALYs.

The input data sources for the estimation of risk factors in India include large-scale national household surveys, population-level surveys provided by collaborators, programme-level data from government agencies, and systematic reviews of epidemiological studies (appendix pp 14–121). These sources provided empirical estimates of risk factor exposure with incorporation of relevant predictive covariates in statistical models to obtain a summary measure of exposure for each risk, called the summary exposure value (SEV). This metric captures risk-weighted exposure for a population, or risk-weighted prevalence of an exposure, the details of which are described elsewhere.^22^ The scale for SEV spans from 0% to 100%, with an SEV of 0% reflecting no risk exposure in a population and 100% showing that an entire population is exposed to the maximum possible risk. We then combined the estimates for SEV with relative risk estimation for health outcomes with sufficient evidence of a causal relationship to provide estimates of population attributable fractions of disease caused by each risk factor.

All estimates in GBD are strengthened by using covariates that are associated with the variable being estimated. This is particularly useful when data for a variable are scarce.

### Grouping of states

We grouped the states of India according to their epidemiological transition level (ETL) in 2016, which was defined as the ratio of all-age DALYs due to CMNNDs versus those due to NCDs and injuries together. A smaller ratio indicates advancing epidemiological transition—ie, higher burden of NCDs and injuries than CMNNDs. The states with ratios of 0·56–0·75 in 2016 were considered to have low ETLs (Bihar, Jharkhand, Uttar Pradesh, Rajasthan, Meghalaya, Assam, Chhattisgarh, Madhya Pradesh, and Odisha; total population 626 million in 2016), those with ratios of 0·41–0·55 had lower-middle ETLs (Arunachal Pradesh, Mizoram, Nagaland, Uttarakhand, Gujarat, Tripura, Sikkim, and Manipur; total population 92 million), those with ratios of 0·31–0·40 had higher-middle ETLs (Haryana, Delhi, Telangana, Andhra Pradesh, Jammu and Kashmir, Karnataka, West Bengal, Maharashtra, and union territories other than Delhi; total population 446 million), and those with ratios less than 0·31 had high ETLs (Himachal Pradesh, Punjab, Tamil Nadu, Goa, and Kerala; total population 152 million). Kerala had the lowest ratio of 0·16. The highest ETL group could have been split into two groups, with Kerala and Goa in one group and the other three states in the other groups, but this would have led to very small groups. We preferred state groups with at least five states, because describing very small groups of states would not be very different from describing individual states. The ranges of ratios that we used to define the ETL groups varied, with a range of 0·2 (0·56–0·75 inclusive) for low ETL, 0·15 (0·41–0·55 inclusive) for lower-middle ETL, and 0·1 (0·31–0·40 inclusive) for higher-middle ETL. The logic for this is that as the ratio increases towards 1, a larger range captures similar levels of CMNNDs. A combination of this logic and breaks in between clusters of ratios were used to define the ETL groups.

We present in this paper trends of epidemiological transition, deaths, DALYs, YLLs, YLDs, prevalence, incidence, risk factors, and life expectancy for the Indian states from 1990 to 2016, highlighting major variations between ETL state groups and states, which are relevant to inform more nuanced health-system and policy development across the states of India.

We related the epidemiological transition ratios across the states with their Socio-Demographic Index (SDI), which is based on an equal weighting of lag-distributed income per capita, average years of education in the population older than 15 years, and total fertility rate.^19^ We present results as all-age rates to show the disease or risk factor burden that the states have to deal with in reality at any given time, as well as age-standardised rates using a global population reference age structure to assess epidemiological differences after adjusting for differences in population age structure.

GBD computes 95% uncertainty intervals (UIs) around estimates. We assessed whether the DALY estimates for the causes and risk factors for each state were significantly different from the national mean, using 95% UIs based on 1000 draws.

### Role of the funding source

Some staff of the Indian Council of Medical Research and the Bill & Melinda Gates Foundation are coauthors on this paper as they contributed to various aspects of this study. The other funder of this study had no role in the study design, data collection, data analysis, data interpretation, or writing of this paper. The corresponding author had full access to all the data in the study and had final responsibility for the decision to submit for publication.

## Results

**Figure 1:**
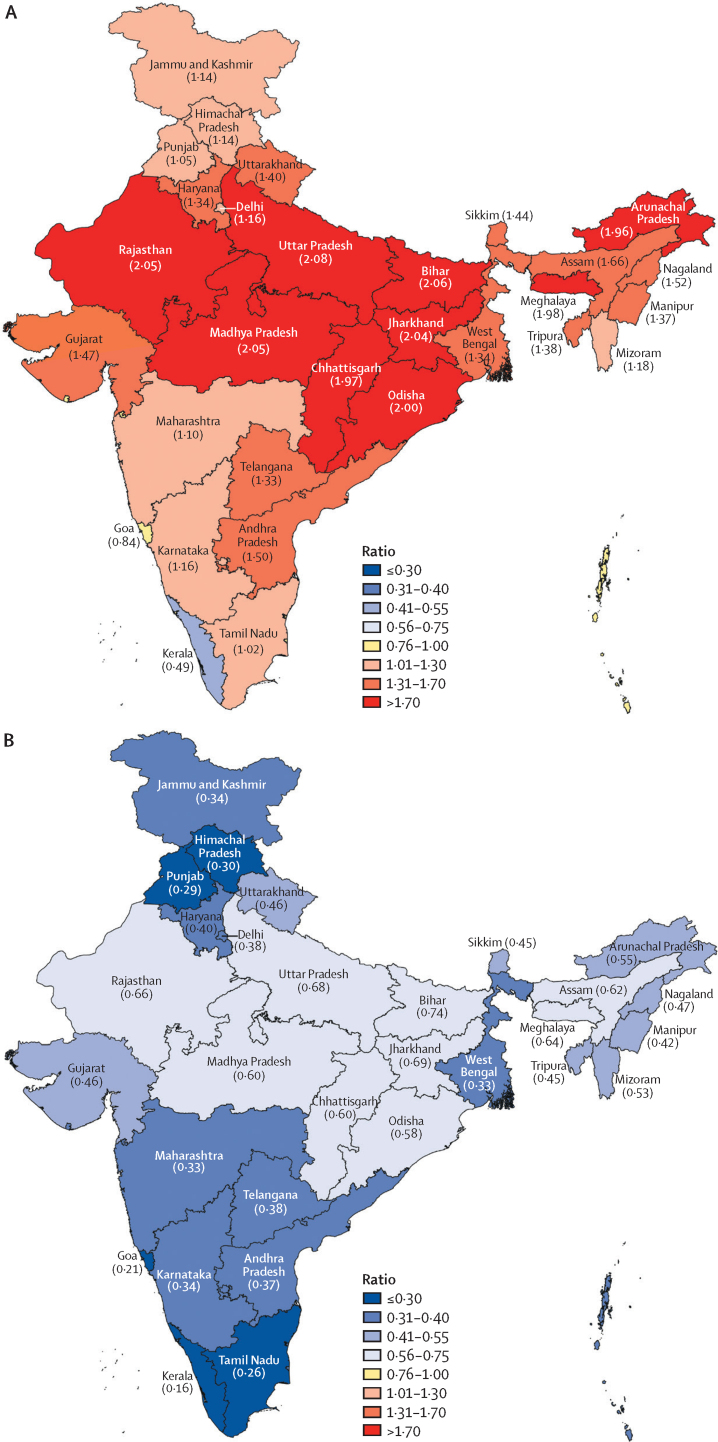
Epidemiological transition ratios of the states of India (A) 1990 and (B) 2016. The states of Chhattisgarh, Jharkhand, Telangana, and Uttarakhand did not exist in 1990, as they were created from existing larger states in 2000 or later. Data for these four new states were disaggregated from their parent states based on their current district composition. These states are shown in the 1990 map for comparison with 2016.

**Figure 2:**
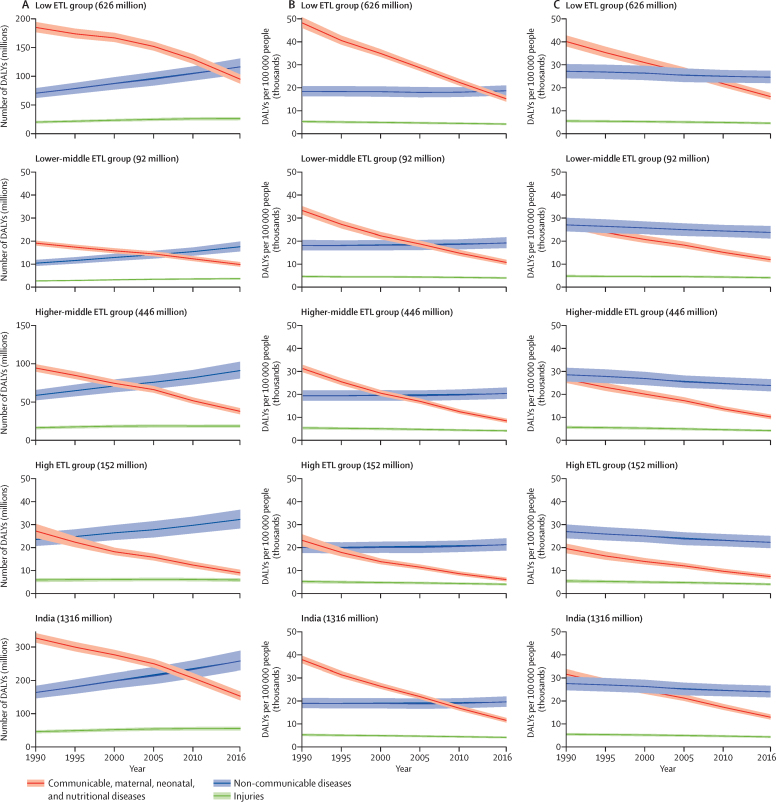
DALYS for states grouped by epidemiological transition level and all of India from 1990 to 2016 (A) Total DALYs in millions. (B) All-age DALY rates per 100 000 people. (C) Age-standardised DALY rates per 100 000 people. DALY=disability-adjusted life-year. ETL=epidemiological transition level.

All states of India had epidemiological transition ratios of 0·75 or less in 2016 (figure 1). By contrast, in 1990, only Kerala (ETL 0·49) had a ratio of 0·75 or less, Goa (0·84) and the union territories other than Delhi (0·85) had ratios 0·76–1·00 and all other states had ratios of one or more, showing a major shift across all states between 1990 and 2016. The percent reduction of epidemiological transition ratios ranged from 55·2% to 75·4% across the states of India (appendix p 122). The year in which the epidemiological transition ratio dropped to less than 1—and thus, the point at which NCDs and injuries accounted for more DALYs than CMNNDs—was 1986 for the high ETL state group, 1996 for the higher-middle ETL state group, 2000 for the lower-middle ETL state group, and 2010 for the low ETL state group, showing a 24-year variation across the groups; this epidemiological transition year for India as a whole was 2003 (figure 2). A significant inverse relationship existed between the epidemiological transition ratio and the SDI of the states, with a correlation coefficient of −5·82 in 1990 and −1·81 in 2016. This shows that the relationship between SDI and epidemiological transition ratio was stronger in 1990 than in 2016 (appendix p 130).

In 2016, deaths due to CMNNDs were 34·7% (95% UI 31·9–39·7) and those due to NCDs were 55·2% (50·6–58·1) of the total number of deaths in the low ETL group, and 15·9% (13·9–19·3) and 72·3% (68·9–74·2) in the high ETL state group (table 1). For India as a whole, 27·5% (95% UI 25·4–31·5) of deaths were due to CMNNDs, 61·8% (58·2–64·0) due to NCDs, and 10·7% (9·6–11·2) due to injuries in 2016 (table 2). For the disease categories within CMNNDs, the proportional contribution to deaths decreased from the lowest to the highest ETL groups. In the NCD categories, the proportion of deaths due to cardiovascular diseases was highest in the high ETL state group and lowest in the low ETL group, but deaths due to chronic respiratory diseases were highest in the low ETL group and lowest in the high ETL group. The proportion of total deaths in the 0–14 years age group was highest in the low ETL state group (14·2%) and lowest in the high ETL state group (4·1%). In this age group, CMNNDs were responsible for the majority of deaths, contributing to 82·5% (95% UI 80·3–84·5) in the low and 72·1% (68·2–76·6) in the high ETL state groups. The proportion of deaths due to injuries was highest in the 15–39 years age group, with 33·6% (95% UI 31·1–35·3) in the low ETL state group and 43·0% (39·6–45·1) in the high ETL state group. The proportion of deaths due to cardiovascular diseases was similar between the 40–69 years and 70 years or older age groups, but, when comparing these two age groups, the proportion of deaths due to neoplasms was higher in the 40–69 years age group, whereas the proportion of deaths due to chronic respiratory diseases was higher in the 70 years or older age group; this trend was similar across the ETL state groups.

**Table 1:** Percentage contribution of disease categories to total deaths by age groups in states grouped by ETL, 2016

	Low ETL group (ratios 0·56–0·75)*					Lower-middle ETL group (ratios 0·41–0·55)*					Higher-middle ETL group (ratios 0·31–0·40)*					High ETL group (ratios ≤0·30)*				
	All ages	0–14 years (14·2% of total deaths)	15–39 years (11·8% of total deaths)	40–69 years (38·6% of total deaths)	≥70 years (35·4% of total deaths)	All ages	0–14 years (10·5% of total deaths)	15–39 years (12·0% of total deaths)	40–69 years (39·6% of total deaths)	≥70 years (37·9% of total deaths)	All ages	0–14 years (6·9% of total deaths)	15–39 years (11·5% of total deaths)	40–69 years (51·7% of total deaths)	≥70 years (40·0% of total deaths)	All ages	0–14 years (4·1% of total deaths)	15–39 years (9·1% of total deaths)	40–69 years (41·0% of total deaths)	≥70 years (45·8% of total deaths)
**Communicable, maternal, neonatal, and nutritional diseases**	**34·7 (31·9–39·7)**	**82·5 (80·3–84·5)**	**34·6 (32·2–38·2)**	**21·9 (19·7–26·1)**	**29·6 (24·4–38·0)**	**26·0 (23·9–28·5)**	**79·3 (77·3–81·4)**	**28·7 (27·0–30·7)**	**17·0 (15·8–18·9)**	**19·8 (16·3–24·4)**	**20·9 (19·1–24·2)**	**77·9 (75·5–80·2)**	**23·6 (21·9–25·8)**	**13·4 (12·2–15·7)**	**18·2 (14·9–23·5)**	**15·9 (13·9–19·3)**	**72·1 (68·2–76·6)**	**18·3 (16·6–20·6)**	**10·9 (9·5–13·2)**	**14·8 (12·1–20·0)**
HIV/AIDS and tuberculosis	6·4 (6·0–6·8)	1·1 (0·9–1·2)	12·7 (11·9–13·5)	8·6 (8·0–9·1)	4·1 (3·6–4·6)	6·9 (6·5–7·3)	1·4 (1·1–1·6)	14·4 (13·2–15·8)	8·7 (8·0–9·3)	4·2 (3·7–4·6)	4·2 (3·9–4·5)	1·2 (1·1–1·3)	10·2 (9·5–11·1)	5·1 (4·7–5·5)	2·1 (1·9–2·3)	3·4 (3·2–3·7)	0·9 (0·8–1·1)	7·7 (6·9–8·5)	4·1 (3·9–4·5)	2·1 (1·9–2·3)
Diarrhoea, lower respiratory, and other common infectious diseases	19·8 (16·9–25·3)	38·4 (35·3–41·6)	12·5 (9·9–17·0)	11·0 (8·7–15·5)	24·4 (18·8–33·4)	12·4 (10·4–15·2)	31·0 (28·2–33·8)	8·2 (6·8–10·2)	6·6 (5·4–8·5)	14·7 (11·1–19·5)	11·7 (9·7–15·3)	28·5 (25·7–31·8)	7·8 (6·2–10·3)	6·7 (5·4–9·2)	15·2 (11·9–20·7)	9·7 (7·8–13·2)	27·6 (24·1–31·5)	6·9 (5·3–9·3)	5·7 (4·4–8·1)	12·2 (9·3–17·5)
Neglected tropical diseases and malaria	1·1 (0·6–1·5)	4·0 (1·9–6·0)	1·7 (0·9–2·2)	0·7 (0·3–0·8)	0·2 (0·1–0·3)	0·9 (0·4–1·3)	4·4 (1·9–6·8)	1·4 (0·6–1·9)	0·6 (0·2–0·7)	0·2 (0·1–0·3)	0·6 (0·3–0·8)	2·9 (1·5–4·0)	1·2 (0·5–1·6)	0·5 (0·2–0·6)	0·2 (0·1–0·2)	0·4 (0·2–0·5)	2·5 (1·4–3·7)	0·8 (0·4–1·2)	0·4 (0·2–0·5)	0·2 (0·1–0·2)
Maternal disorders	0·7 (0·6–0·8)	0·0 (0·0–0·0)	5·1 (4·3–5·9)	0·2 (0·1–0·2)	NA	0·3 (0·3–0·4)	0·0 (0·0–0·0)	2·6 (2·1–3·1)	0·1 (0·1–0·1)	NA	0·3 (0·3–0·3)	0·0 (0·0–0·0)	2·3 (2·0–2·6)	0·1 (0·1–0·1)	NA	0·2 (0·1–0·2)	0·0 (0·0–0·0)	1·7 (1·4–2·0)	0·0 (0·0–0·0)	NA
Neonatal disorders	4·9 (4·5–5·4)	34·7 (33·3–36·2)	NA	NA	NA	4·1 (3·5–5·0)	39·5 (37·3–42·1)	NA	NA	NA	2·9 (2·6–3·3)	42·2 (40·8–43·6)	NA	NA	NA	1·6 (1·3–1·9)	38·8 (36·0–42·2)	NA	NA	NA
Nutritional deficiencies	0·7 (0·6–0·8)	2·5 (2·1–3·0)	0·4 (0·4–0·5)	0·4 (0·4–0·4)	0·4 (0·3–0·4)	0·3 (0·3–0·4)	1·5 (1·2–1·9)	0·2 (0·2–0·2)	0·2 (0·2–0·2)	0·2 (0·2–0·2)	0·3 (0·3–0·4)	1·6 (1·4–1·9)	0·3 (0·2–0·3)	0·3 (0·2–0·3)	0·2 (0·2–0·3)	0·2 (0·1–0·2)	1·0 (0·8–1·3)	0·1 (0·1–0·2)	0·1 (0·1–0·1)	0·1 (0·1–0·1)
Other communicable, maternal, neonatal, and nutritional diseases	1·1 (1·0–1·2)	1·8 (1·4–2·2)	2·3 (2·0–2·4)	1·0 (0·9–1·1)	0·5 (0·4–0·5)	0·9 (0·9–1·0)	1·5 (1·2–2·0)	1·9 (1·7–2·1)	0·9 (0·8–1·0)	0·5 (0·4–0·5)	0·8 (0·8–0·9)	1·6 (1·3–1·9)	1·8 (1·7–1·9)	0·8 (0·8–0·9)	0·4 (0·4–0·5)	0·5 (0·4–0·5)	1·3 (1·0–1·8)	1·0 (0·9–1·2)	0·5 (0·4–0·5)	0·3 (0·2–0·3)
**Non-communicable diseases**	**55·2 (50·6–58·1)**	**10·8 (9·2–12·5)**	**31·9 (30·3–33·5)**	**69·1 (65·3–71·4)**	**65·5 (57·7–70·8)**	**63·3 (60·8–65·4)**	**13·3 (11·9–14·8)**	**35·3 (33·8–37·5)**	**74·1 (72·2–75·5)**	**74·8 (70·4–78·1)**	**67·9 (64·9–69·8)**	**13·9 (12·4–15·5)**	**37·0 (35·7–39·4)**	**77·2 (75·0–78·5)**	**76·5 (71·3–79·5)**	**72·3 (68·9–74·2)**	**18·5 (14·9–21·7)**	**38·7 (37·1–41·6)**	**78·3 (76·0–79·9)**	**78·3 (73·2–81·0)**
Neoplasms	7·9 (7·3–8·3)	0·8 (0·6–0·9)	6·0 (5·6–6·4)	12·6 (11·9–13·1)	6·2 (5·5–6·7)	8·4 (8·0–8·8)	1·2 (1·0–1·4)	5·8 (5·5–6·2)	13·1 (12·5–13·7)	6·2 (5·8–6·7)	8·7 (8·3–9·0)	1·4 (1·2–1·6)	6·3 (6·0–6·6)	12·8 (12·3–13·2)	6·4 (6·0–6·7)	9·0 (8·5–9·3)	1·7 (1·4–2·0)	5·8 (5·5–6·2)	13·4 (12·8–14·0)	6·3 (5·8–6·5)
Cardiovascular diseases	21·9 (20·1–23·1)	0·4 (0·4–0·5)	10·5 (9·9–11·1)	28·2 (26·5–29·3)	27·4 (24·3–29·5)	28·9 (27·6–30·1)	0·6 (0·5–0·7)	13·6 (12·8–14·5)	34·7 (33·4–35·8)	35·5 (33·5–37·4)	34·1 (32·5–35·1)	0·7 (0·6–0·8)	14·6 (14·0–15·4)	39·3 (38·0–40·3)	40·0 (37·4–41·6)	37·4 (35·6–38·7)	0·8 (0·7–1·0)	17·4 (16·3–18·4)	40·3 (38·8–41·4)	42·1 (39·3–44·0)
Chronic respiratory diseases	12·2 (10·9–13·5)	0·3 (0·2–0·5)	2·3 (2·1–2·8)	14·2 (13·0–15·4)	18·1 (15·7–20·3)	11·8 (10·9–12·6)	0·4 (0·3–0·5)	2·5 (2·3–2·8)	12·1 (11·1–13·0)	17·5 (16·2–18·8)	10·0 (9·3–11·0)	0·3 (0·2–0·4)	1·9 (1·8–2·3)	9·8 (9·2–10·7)	14·3 (13·1–15·9)	7·4 (6·7–9·1)	0·3 (0·2–0·5)	1·6 (1·5–2·1)	6·8 (6·2–8·2)	9·6 (8·6–12·2)
Cirrhosis and other chronic liver diseases	1·8 (1·6–2·4)	0·2 (0·2–0·4)	2·9 (2·6–3·7)	2·8 (2·6–3·8)	0·9 (0·7–1·2)	2·1 (1·9–2·5)	0·2 (0·2–0·5)	3·5 (3·1–4·1)	3·2 (2·9–3·9)	1·0 (0·9–1·2)	2·7 (2·4–2·9)	0·3 (0·2–0·5)	4·4 (3·8–5·0)	4·0 (3·5–4·5)	1·1 (1·0–1·3)	1·8 (1·6–2·7)	0·3 (0·2–0·6)	2·9 (2·5–4·4)	2·8 (2·5–4·2)	0·9 (0·8–1·2)
Digestive diseases	2·6 (2·3–2·8)	0·8 (0·6–1·1)	3·2 (2·7–3·4)	3·4 (2·9–3·7)	2·3 (2·0–2·6)	1·8 (1·6–2·3)	0·6 (0·4–0·7)	2·0 (1·8–2·6)	2·2 (2·0–2·9)	1·7 (1·5–2·1)	1·7 (1·5–2·4)	0·6 (0·5–0·7)	1·9 (1·7–2·7)	2·0 (1·7–2·9)	1·5 (1·3–2·0)	1·5 (1·3–2·2)	0·5 (0·4–0·7)	1·7 (1·4–2·5)	1·8 (1·5–2·7)	1·3 (1·1–1·9)
Neurological disorders	1·8 (1·5–2·1)	0·5 (0·4–0·6)	1·3 (1·2–1·5)	0·8 (0·7–0·9)	3·6 (2·9–4·5)	2·3 (2·0–2·7)	0·8 (0·7–0·9)	1·7 (1·5–1·9)	1·0 (0·9–1·1)	4·4 (3·7–5·3)	2·2 (2·0–2·6)	0·7 (0·6–0·9)	1·4 (1·4–1·6)	0·9 (0·8–1·0)	4·1 (3·4–5·0)	2·9 (2·5–3·4)	0·9 (0·7–1·2)	1·5 (1·4–1·7)	1·0 (0·9–1·1)	4·9 (4·1–6·0)
Mental and substance use disorders	0·4 (0·3–0·4)	0·0 (0·0–0·0)	1·0 (0·8–1·2)	0·6 (0·4–0·6)	0·1 (0·1–0·1)	0·4 (0·3–0·5)	0·0 (0·0–0·0)	1·2 (0·9–1·4)	0·6 (0·4–0·7)	0·1 (0·1–0·1)	0·4 (0·3–0·5)	0·0 (0·0–0·0)	1·1 (0·9–1·3)	0·5 (0·4–0·6)	0·1 (0·1–0·1)	0·4 (0·3–0·4)	0·0 (0·0–0·0)	1·2 (0·9–1·4)	0·5 (0·4–0·6)	0·1 (0·1–0·1)
Diabetes, urogenital, blood, and endocrine diseases	5·2 (4·7–6·0)	0·7 (0·6–0·8)	3·7 (3·5–4·5)	6·1 (5·7–7·0)	6·5 (5·7–7·4)	6·4 (6·0–6·8)	0·9 (0·8–1·0)	4·2 (3·7–4·8)	6·9 (6·5–7·3)	8·1 (7·5–8·7)	7·0 (6·7–7·3)	0·9 (0·8–1·0)	4·4 (4·2–4·9)	7·4 (7·0–7·7)	8·5 (7·9–9·0)	11·0 (9·9–11·6)	1·1 (0·9–1·2)	5·5 (5·1–5·9)	11·2 (9·9–11·8)	12·9 (11·6–13·7)
Musculoskeletal disorders	0·1 (0·1–0·1)	0·0 (0·0–0·0)	0·1 (0·1–0·1)	0·1 (0·1–0·1)	0·1 (0·1–0·1)	0·1 (0·1–0·1)	0·0 (0·0–0·0)	0·1 (0·1–0·1)	0·1 (0·1–0·1)	0·1 (0·1–0·1)	0·1 (0·1–0·1)	0·0 (0·0–0·0)	0·1 (0·1–0·1)	0·1 (0·1–0·1)	0·1 (0·1–0·1)	0·1 (0·1–0·1)	0·0 (0·0–0·0)	0·1 (0·1–0·1)	0·1 (0·1–0·1)	0·1 (0·1–0·1)
Other non-communicable diseases	1·3 (1·0–1·6)	7·1 (5·6–8·7)	0·8 (0·6–1·0)	0·3 (0·2–0·4)	0·2 (0·1–0·3)	1·2 (1·0–1·4)	8·7 (7·1–10·0)	0·7 (0·6–0·8)	0·3 (0·2–0·4)	0·2 (0·1–0·3)	0·9 (0·8–1·1)	9·0 (7·6–10·4)	0·8 (0·7–0·9)	0·3 (0·2–0·4)	0·2 (0·1–0·3)	0·8 (0·6–1·0)	13·0 (8·8–16·0)	1·0 (0·7–1·1)	0·3 (0·3–0·4)	0·2 (0·1–0·3)
**Injuries**	**10·1 (9·1–10·7)**	**6·7 (5·6–7·7)**	**33·6 (31·1–35·3)**	**9·0 (7·9–9·6)**	**4·9 (4·2–5·4)**	**10·7 (9·9–11·2)**	**7·4 (6·4–8·3)**	**36·0 (33·9–37·7)**	**8·9 (8·2–9·4)**	**5·4 (4·8–6·0)**	**11·2 (10·0–11·7)**	**8·2 (7·0–9·4)**	**39·5 (36·4–41·3)**	**9·4 (8·2–9·9)**	**5·4 (4·6–5·9)**	**11·9 (9·9–12·6)**	**9·3 (7·9–10·7)**	**43·0 (39·6–45·1)**	**10·8 (8·9–11·5)**	**6·8 (4·9–7·6)**
Transport injuries	2·9 (2·7–3·0)	1·1 (0·9–1·3)	11·2 (10·4–12·1)	2·9 (2·7–3·1)	0·7 (0·6–0·8)	2·9 (2·7–3·1)	1·3 (1·1–1·6)	11·3 (10·3–12·4)	2·8 (2·6–3·0)	0·7 (0·7–0·8)	2·9 (2·8–3·1)	1·5 (1·3–1·7)	11·7 (11·0–12·6)	2·9 (2·7–3·0)	0·7 (0·7–0·8)	3·2 (3·0–3·4)	1·9 (1·5–2·3)	13·8 (12·7–15·2)	3·5 (3·2–3·8)	0·9 (0·8–1·0)
Unintentional injuries	5·0 (4·1–5·4)	5·2 (4·1–6·0)	9·9 (8·0–10·9)	4·4 (3·5–4·8)	3·9 (3·3–4·3)	4·9 (4·3–5·3)	5·5 (4·7–6·3)	9·3 (7·6–10·2)	4·0 (3·5–4·3)	4·3 (3·7–4·8)	4·8 (4·1–5·1)	6·0 (4·8–6·8)	9·2 (7·8–9·9)	3·9 (3·4–4·2)	4·1 (3·5–4·6)	5·2 (3·9–5·7)	6·4 (5·1–7·5)	8·6 (7·2–9·2)	4·3 (3·3–4·7)	5·3 (3·5–6·0)
Self-harm and interpersonal violence	2·3 (2·1–2·5)	0·4 (0·3–0·5)	12·4 (11·4–13·5)	1·7 (1·5–1·9)	0·3 (0·3–0·4)	2·8 (2·6–3·1)	0·5 (0·4–0·6)	15·3 (14·1–16·7)	2·0 (1·8–2·3)	0·4 (0·3–0·4)	3·5 (2·8–3·8)	0·7 (0·6–0·9)	18·5 (15·8–20·2)	2·6 (1·9–2·9)	0·5 (0·4–0·6)	3·4 (2·6–3·8)	1·0 (0·8–1·2)	20·6 (17·1–22·8)	3·0 (2·0–3·4)	0·6 (0·4–0·7)
Forces of nature, conflict and terrorism, and executions and police conflict	0·0 (0·0–0·0)	0·0 (0·0–0·0)	0·0 (0·0–0·1)	0·0 (0·0–0·0)	0·0 (0·0–0·0)	0·0 (0·0–0·0)	0·0 (0·0–0·0)	0·0 (0·0–0·1)	0·0 (0·0–0·0)	0·0 (0·0–0·0)	0·0 (0·0–0·0)	0·0 (0·0–0·1)	0·0 (0·0–0·1)	0·0 (0·0–0·0)	0·0 (0·0–0·0)	0·0 (0·0–0·0)	0·1 (0·0–0·1)	0·1 (0·0–0·1)	0·0 (0·0–0·0)	0·0 (0·0–0·0)

Data are % (95% uncertainty interval). ETL=epidemiological transition level. NA=not applicable.

* Epidemiological transition ratios for ETL group.

**Table 2:** Percentage contribution of disease categories to total deaths by age groups for all of India, 2016

	**All ages**	**0–14 years (10·4% of total deaths)**	**15–39 years (11·4% of total deaths)**	**40–69 years (39·9% of total deaths)**	**≥70 years (38·2% of total deaths)**
**Communicable, maternal, neonatal, and nutritional diseases**	**27·5 (25·4–31·5)**	**80·8 (78·7–82·8)**	**29·1 (27·2–31·9)**	**17·4 (15·8–20·5)**	**23·0 (19·3–29·4)**
HIV/AIDS and tuberculosis	5·4 (5·1–5·6)	1·1 (1·0–1·2)	11·5 (11·0–12·1)	6·9 (6·5–7·2)	3·1 (2·8–3·4)
Diarrhoea, lower respiratory, and other common infectious diseases	15·5 (13·3–19·9)	35·3 (32·4–38·4)	10·1 (8·1–13·6)	8·7 (7·0–12·1)	19·0 (14·9–25·6)
Neglected tropical diseases and malaria	0·8 (0·4–1·1)	3·7 (1·8–5·3)	1·4 (0·7–1·8)	0·6 (0·3–0·7)	0·2 (0·1–0·3)
Maternal disorders	0·5 (0·4–0·5)	0·0 (0·0–0·0)	3·7 (3·3–4·1)	0·1 (0·1–0·1)	NA
Neonatal disorders	3·8 (3·6–4·1)	36·9 (35·7–38·2)	NA	NA	NA
Nutritional deficiencies	0·5 (0·4–0·5)	2·2 (1·9–2·5)	0·3 (0·3–0·4)	0·3 (0·3–0·3)	0·3 (0·2–0·3)
Other communicable, maternal, neonatal, and nutritional diseases	0·9 (0·9–1·0)	1·7 (1·4–2·0)	2·0 (1·8–2·1)	0·9 (0·8–0·9)	0·4 (0·4–0·5)
**Non-communicable diseases**	**61·8 (58·2–64·0)**	**12·0 (10·6–13·5)**	**34·4 (33·1–36·4)**	**73·2 (70·4–74·9)**	**71·6 (65·5–75·4)**
Neoplasms	8·3 (7·9–8·6)	1·0 (0·8–1·1)	6·1 (5·8–6·3)	12·8 (12·2–13·2)	6·3 (5·8–6·6)
Cardiovascular diseases	28·1 (26·5–29·1)	0·5 (0·4–0·6)	12·7 (12·1–13·3)	33·8 (32·4–34·7)	34·3 (31·5–35·8)
Chronic respiratory diseases	10·9 (9·9–12·0)	0·3 (0·2–0·5)	2·1 (1·9–2·6)	11·7 (10·9–12·7)	15·6 (13·9–17·4)
Cirrhosis and other chronic liver diseases	2·1 (1·9–2·5)	0·2 (0·2–0·4)	3·4 (3·1–4·0)	3·3 (3·0–3·9)	1·0 (0·9–1·2)
Digestive diseases	2·2 (2·0–2·4)	0·7 (0·6–0·9)	2·5 (2·4–2·8)	2·7 (2·5–3·1)	1·9 (1·7–2·2)
Neurological disorders	2·1 (1·8–2·5)	0·6 (0·5–0·7)	1·4 (1·3–1·5)	0·9 (0·8–1·0)	4·0 (3·3–4·9)
Mental and substance use disorders	0·4 (0·3–0·4)	0·0 (0·0–0·0)	1·1 (0·8–1·2)	0·5 (0·4–0·6)	0·1 (0·1–0·1)
Diabetes, urogenital, blood, and endocrine diseases	6·5 (6·2–6·9)	0·8 (0·7–0·9)	4·2 (3·9–4·7)	7·2 (6·9–7·6)	8·2 (7·5–8·7)
Musculoskeletal disorders	0·1 (0·1–0·1)	0·0 (0·0–0·0)	0·1 (0·1–0·1)	0·1 (0·1–0·1)	0·1 (0·1–0·1)
Other non-communicable diseases	1·1 (0·9–1·3)	7·9 (6·7–9·2)	0·8 (0·7–1·0)	0·3 (0·2–0·4)	0·2 (0·1–0·3)
**Injuries**	**10·7 (9·6–11·2)**	**7·2 (6·1–8·2)**	**36·5 (34·0–38·1)**	**9·4 (8·2–9·8)**	**5·4 (4·5–5·9)**
Transport injuries	2·9 (2·8–3·1)	1·2 (1·1–1·4)	11·6 (11·0–12·4)	3·0 (2·8–3·1)	0·8 (0·7–0·8)
Unintentional injuries	4·9 (4·1–5·3)	5·4 (4·4–6·2)	9·5 (7·8–10·3)	4·2 (3·4–4·5)	4·2 (3·4–4·6)
Self-harm and interpersonal violence	2·8 (2·4–3·1)	0·5 (0·4–0·6)	15·4 (13·8–16·5)	2·2 (1·7–2·4)	0·4 (0·3–0·5)
Forces of nature, conflict and terrorism, and executions and police conflict	0·0 (0·0–0·0)	0·0 (0·0–0·0)	0·0 (0·0–0·1)	0·0 (0·0–0·0)	0·0 (0·0–0·0)

Data are % (95% uncertainty interval). NA=not applicable.

Of the top ten individual causes of death in India in 2016, deaths due to all NCD causes increased between 1990 and 2016; the all-age death rate increased significantly for ischaemic heart disease (percentage change 54·5% [95% UI 44·1 to 66·4%]), diabetes (130·8% [111·1 to 150·4%]), and chronic kidney disease (32·7% [18·4 to 49·3%]); and the age-standardised death rate increased for ischaemic heart disease (12·0% [4·5 to 21·3%]) and diabetes (63·7% [48·1 to 79·1%]), but decreased for chronic obstructive pulmonary disease (COPD; −40·2% [–47·4 to −28·2%]) and cerebrovascular disease (−23·7% [–31·2 to −15·3%]; appendix p 131). Of the leading injury causes, number of deaths increased from road injuries and self-harm (suicide), the all-age and age-standardised death rates increased for road injuries, and the age-standardised death rate decreased for self-harm between 1990 and 2016. The deaths and the all-age and age-standardised death rates decreased significantly for the leading CMNND causes of death such as diarrhoeal diseases, lower respiratory infections, and tuberculosis.

The all-age and age-standardised death rates reduced significantly in India from 1990 to 2016, with a greater reduction in women than that in men (appendix p 132). The reduction in all-age death rates was highest in the low ETL state group (−32·8% [95% UI −36·2 to −29·4]) and lowest in the high ETL state group (−14% [–20·6 to −6·9]), but the reduction in age-standardised death rates was similar across the ETL groups. The ratio of the highest to lowest was 1·9 for all-age death rates and 2·0 for age-standardised death rates between the states in 2016; this ratio was higher for women than for men.

The low ETL state group had 39·9% (95% UI 37·7 to 42·7) of the total DALYs from CMNNDs, 49% (46·4 to 51·0) from NCDs and 11·1% (10·2 to 11·9) from injuries in 2016, and the high ETL state group had 19·5% (18·0 to 21·3), 67·4% (65·5 to 68·9) and 13·1% (11·8 to 14·1) DALYs, respectively (appendix p 124). The number of DALYs due to NCDs increased by 36·4% (28·5 to 45·8) in the high ETL state group from 1990 to 2016, whereas the number increased by 55·0% (47·6 to 62·1) in the higher-middle ETL group, 68·5% (58·2 to 79·5) in the lower-middle group, and 64·9% (55·3 to 76·1) in the low group (appendix p 125). The all-age DALY rates due to NCDs remained almost the same across all ETL state groups over this period, but the age-standardised rates decreased significantly in all four ETL groups, with a minimum decrease of 9·4% (95% UI 14·2 to 4·0) in the low ETL state group and a maximum decrease of 17·3% (21·8 to 12·0) in the high ETL state group. The number of DALYs due to injuries increased significantly from 1990 to 2016 in the lowest (29·5% [95% UI 18·0 to 45·5]), lower-middle (36·6% [23·9 to 51·3]), and higher-middle (13·5% [4·4 to 23·8]) ETL state groups, but the change in the high ETL group was not significant −0·2 (−9·9 to 10·1). However, both the all-age and age-standardised DALY rates due to injuries significantly decreased across all ETL state groups. The number of DALYs decreased significantly for CMNNDs from 1990 to 2016 across all ETL state groups (minimum decrease 48·6% [95% UI 53·6 to 43·5] in the lower-middle ETL group, maximum decrease 65·8% [70·5 to 60·9] in the high ETL group), as did the age-standardised rates (minimum decrease 56·2% [60·1 to 51·9] in the lower-middle ETL group, maximum decrease 62·1% [66·7 to 57·2] in the high ETL group).

The disease categories causing 5% or more of total DALYs in India in 2016 were cardiovascular diseases (14·1% [95% UI 12·9–15·3]), diarrhoea, lower respiratory and other common infectious diseases (12·7% [11·1–15·0]), neonatal disorders (7·9% [7·2–8·8]), chronic respiratory diseases (6·4% [5·8–7·0)), diabetes, urogenital, and endocrine diseases (5·6% [5·2–6·0]), mental and substance abuse disorders (5·6% [4·5–6·7]), unintentional injuries (5·4% [4·7–5·8]), and neoplasms (5% [4·6–5·5]; appendix p 124). The contribution of most CMNNDs to the proportion of DALYs decreased, and that of most NCDs and injuries increased from 1990 to 2016 across all ETL state groups.

**Figure 3:**
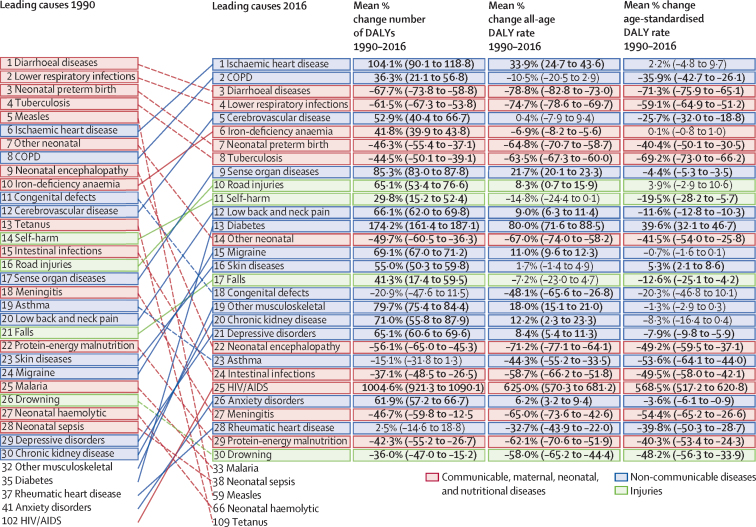
Change in DALY number and percent change in rates for the leading 30 causes 1990–2016, India Causes are connected by lines between time periods. Three measures of change are shown: percent change in the number of DALYs, percent change in all-age DALY rate and percent change in age-standardised DALY rate. COPD=chronic obstructive pulmonary disease. DALY=disability-adjusted life-year.

The top five individual causes of disease burden in India in 1990 were CMNNDs, whereas in 2016, three of the top five causes were NCDs, showing a shift toward NCDs (figure 3). The number of DALYs due to most NCDs increased from 1990 to 2016. Of the individual NCDs that are in the top 30 leading causes of DALYs in 2016, the increase in all-age DALY rate between 1990 and 2016 was highest for diabetes (80·0% [95% UI 71·6–88·5]), ischaemic heart disease (33·9% [24·7–43·6]), and sense organ diseases (mainly vision and hearing loss disorders; 21·7% [20·1–23·3]); the rates for low back and neck pain, migraine, other musculoskeletal disorders, chronic kidney disease, depressive disorders, and anxiety disorders were also significantly increased (figure 3). The age-standardised DALY rate increased significantly only for diabetes (39·6% [95% UI 32·1–46·7]) and skin diseases (5·3% [2·1–8·6]). Number of DALYs for COPD increased significantly by 36·3% (95% UI 21·1–56·8) and cerebrovascular disease by 52·9% (40·4–66·7); however, their all-age DALY rates did not change significantly and their age-standardised DALY rates decreased significantly. DALYs due to each of the three leading causes of injury in India increased from 1990 to 2016 (road injuries 65·1% [95% UI 53·4 to 76·6], falls 41·3% [17·4 to 59·5], and self-harm 29·8% [15·2 to 52·4]), the all-age DALY rate increased significantly for road injuries (8·3% [0·7 to 15·9]), and the age-standardised DALY rates decreased significantly for self-harm (−19·5% [–28·2 to −5·7) and falls (−12·6% [–25·1 to −4·2]). The number, all-age rates, and age-standardised rates of all CMNNDs in the leading 30 causes in 1990 decreased substantially by 2016, except for iron-deficiency anaemia, for which the number of DALYs increased by 41·8% (95% UI 39·9–43·8), and the all-age DALY rate decreased by 6·9% with no significant change in age-standardised rate. In 2016, road injuries and self-harm were among the top ten causes of DALYs for men, whereas no injury cause was in the top ten for women (appendix pp 132–133). Iron-deficiency anaemia, migraine, and low back and neck pain were among the top ten causes of DALYs for women, but not for men.

The all-age prevalence increased from 1990 to 2016 for most of the leading NCD causes of DALYs, but the age-standardised prevalence increased only for ischaemic heart disease, cerebrovascular disease, diabetes and skin diseases, whereas all other causes remained unchanged or had minor decreases (appendix p 126). The percent increase in prevalence was more than or similar to the percent change in the DALY rates for most of the leading NCDs. Similarly, among the leading causes of injuries, the percent increases in the incidence rate of road injuries were much higher than the percent increase in DALY rates; for self-harm and falls the DALY rates decreased whereas the incidence did not change markedly.

**Figure 4:**
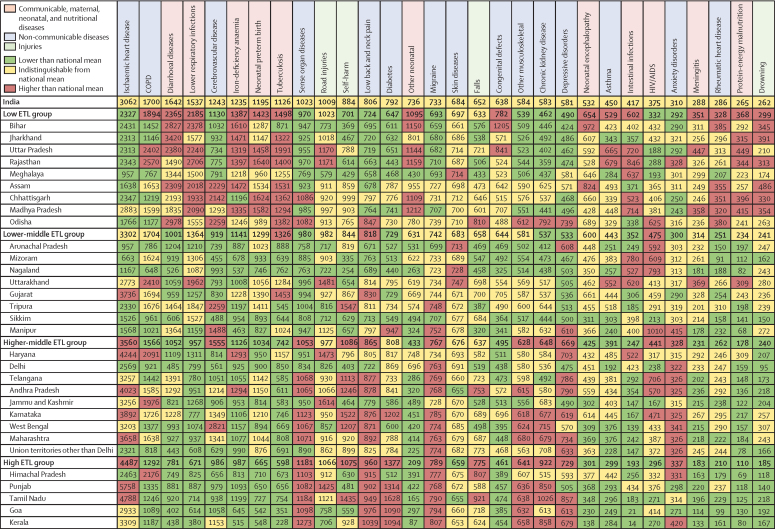
All-age DALY rates of the leading 30 causes of DALYs in the states of India, 2016 Green shows values lower than the national mean all-age DALY rate for that cause, yellow shows values indistinguishable from the national mean, and red shows value higher than the national mean. COPD=chronic obstructive pulmonary disease. DALY=disability-adjusted life-year. ETL=epidemiological transition level.

The DALY rates were not consistent across the state ETL groups for individual NCDs and injuries (figure 4; appendix p 134). Compared with the other ETL state groups, the all-age DALY rates for ischaemic heart disease, diabetes, sense organ disease, self-harm, low back and neck pain, migraine, falls, other musculoskeletal disorders, chronic kidney disease, depressive disorders, and anxiety disorders were highest in the high ETL state group; and the rates for COPD, asthma, congenital defects, rheumatic heart disease, and drowning were higher in the low ETL state group. The ETL group with the highest DALY rate for cerebrovascular disease was the higher-middle ETL group, but the individual states with the highest DALY rates were spread over the low, lower-middle, and higher-middle ETL groups (figure 4). Some variations were recorded within the ETL state groups: within the high ETL state group, all-age DALY rates were about two-times higher for ischaemic heart disease in Punjab and Tamil Nadu than in Himachal Pradesh, and within the low ETL state group, rates of COPD were markedly higher in Rajasthan and Uttar Pradesh than other states. The all-age DALY rates for the leading CMNNDs were generally highest in the low ETL state group, with substantial variations between individual states within ETL groups. For example, in the low ETL state group, Odisha and Jharkhand had the highest DALY rates for diarrhoeal diseases but had some of the lowest rates for lower respiratory infections. The range of all-age DALY rates across the states was wide for many of the leading causes, and the highest state-specific rate was more than five times the lowest state-specific rate for five of the top ten causes in 2016 (appendix p 135).

The ratio of the observed versus expected DALY rate based on SDI were assessed (appendix p 127). The highest ratios for ischaemic heart disease, diabetes, and chronic kidney disease were in the high ETL state group, and the highest ratios for COPD and asthma were in the low ETL state group, similar to the trends of DALY rates of these diseases across the ETL groups (figure 4). By contrast, the highest ratio of the observed versus expected DALY rate for diarrhoeal diseases and iron-deficiency anaemia was in the high ETL state group, whereas the DALY rates for these were the lowest in the high ETL state group. For India as a whole, the ratios of the observed versus expected DALY rates were 2·10–3·00 for COPD, diarrhoeal diseases, iron-deficiency anaemia, other neonatal disorders and rheumatic heart disease; 3·61 for tuberculosis and 4·19 for HIV/AIDS; and 61·75 for intestinal infectious diseases (mainly typhoid and paratyphoid fevers; appendix p 127).

DALY rates reduced significantly in India by 43·1% (95% UI 45·9–40·3; all-age rate) and 36·2% (38·6–33·8; age-standardised rate) from 1990 to 2016 (appendix p 128). The reductions were slightly greater for women than for men. The ratio of the all-age DALY rate in the low ETL to the high ETL state group dropped from 1·48 in 1990 to 1·20 in 2016, but this ratio did not change much for age-standardised DALY rates. The highest age-standardised DALY rate of an individual state in 2016 was 1·8 times the lowest.

**Figure 5:**
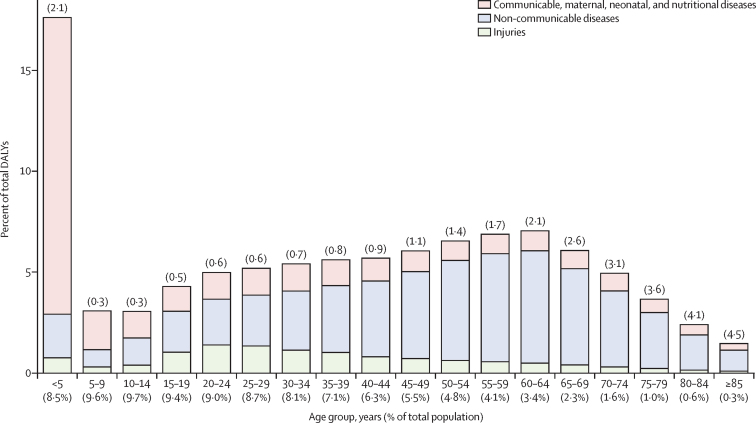
Percent of total DALYs by age groups in India, 2016 The number on top of each vertical bar is the ratio of percent DALYs to population for that age group. DALY=disability-adjusted life-year.

We assessed DALY burden in India in 2016 by age group, taking into account the proportion of the total population that each age group contributes. The age groups of younger than 5 years and 45 years or older all had a higher proportion of the total DALY burden relative to their proportion of the population (ratio >1; figure 5). The younger than 5 years group had 17·6% of the DALYs and constituted 8·5% of the population, a ratio of 2·1, which was an improvement from a ratio of 3·6 in 1990 (appendix p 136). This ratio of DALYs to population in 2016 increased from 1·1 in the 45–49 years group to 2·1 in the 60–64 years group, and further to 4·5 in the 85 years and older group. The highest proportion of DALYs attributed to CMNNDs were in children younger than 5 years (83·4%), and the lowest was in the 50–54 year age group (14·7%). The proportion of DALYs due to NCDs exceeded 50% in the 30–34 years group and was highest at 78·8% in the 65–69 years group. The proportion of total DALYs due to injuries was highest in the age groups from 15 years to 39 years (range 18·3–28·1%).

Of the total DALYs in India, 67·2% were YLLs and 32·8% were YLDs in 2016, whereas 82·5% were YLLs and 17·5% were YLDs in 1990 (appendix p 137). In 2016, the YLD proportion increased with ETL, from 29·6% in the low group to 39·1% in the high ETL state group. Among the leading 30 disease burden causes in India in 2016, sense organ diseases, low back and neck pain, migraine, depressive disorders, and anxiety disorders contributed only YLDs; and iron-deficiency anaemia, skin diseases, musculoskeletal disorders, and protein-energy malnutrition contributed more YLDs than YLLs (appendix p 138). The leading causes of YLDs were quite different from the leading causes of YLLs. Low back and neck pain, migraine, skin diseases, depressive disorders, other musculoskeletal disorders, diabetes, and anxiety disorders featured in the top ten causes of YLDs, but not in the top ten causes of DALYs. Migraine, depressive disorders, and anxiety disorders had a higher YLD ranking among women than men (data not shown).

**Figure 6:**
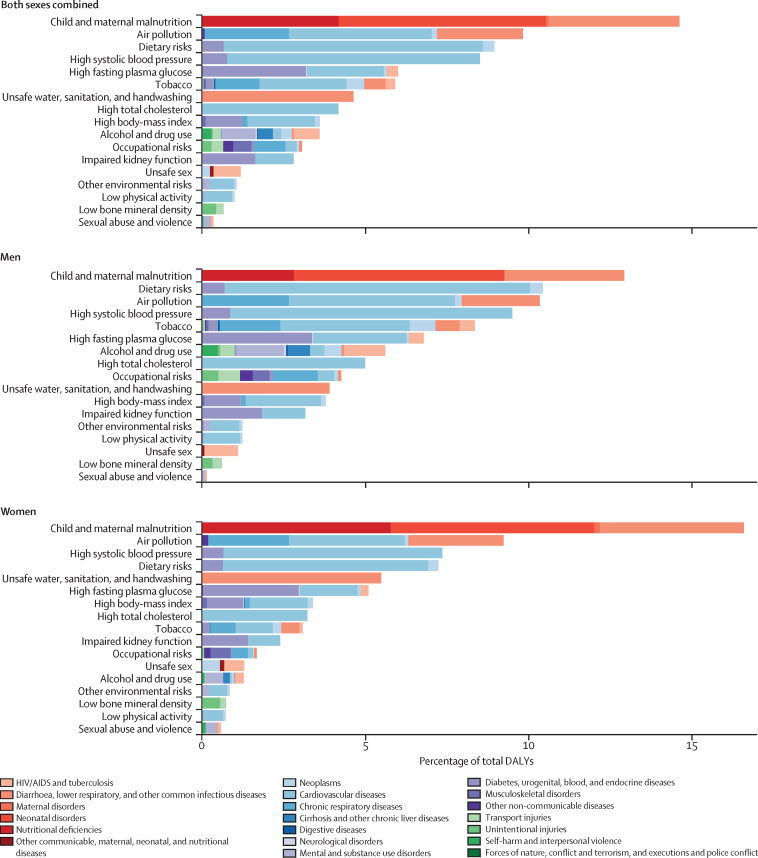
DALYs attributable to risk factors in India, 2016 DALY=disability-adjusted life-year.

The leading risk factors in India in 2016 responsible for more than 5% of the total DALYs each were child and maternal malnutrition (undernutrition; 14·6%), air pollution (9·8%), dietary risks (unhealthy diet; 8·9%), high systolic blood pressure (8·5%), high fasting plasma glucose (6%), and tobacco use (includes smoking, second-hand smoke, and smokeless tobacco; 5·9%; figure 6). Child and maternal malnutrition consisted of child growth failure (underweight, wasting, and stunting), low birthweight and short gestation, suboptimal breastfeeding, iron-deficiency anaemia, vitamin A deficiency, and zinc deficiency. Dietary risks comprised of ten components that are protective such as low fruit, low vegetables, low whole grains, and low nuts and seeds, and five components that are harmful such as high sodium, high trans-fats and high red meat. Dietary risks, systolic blood pressure, high total cholesterol, tobacco, and alcohol and drug use contributed more to the DALYs in men than in women, whereas child and maternal malnutrition and unsafe water, sanitation, and handwashing contributed more in women than in men. The proportion of total DALYs due to child and maternal malnutrition and unsafe water sanitation and handwashing were much higher in the low than in the high ETL state group (appendix p 139). The proportion for air pollution was also higher in the low than in the high ETL state group. Conversely, the proportion of total DALYs due to dietary risks, high systolic blood pressure, high fasting plasma glucose, high cholesterol, and high body-mass index were highest in the high ETL state group.

**Figure 7:**
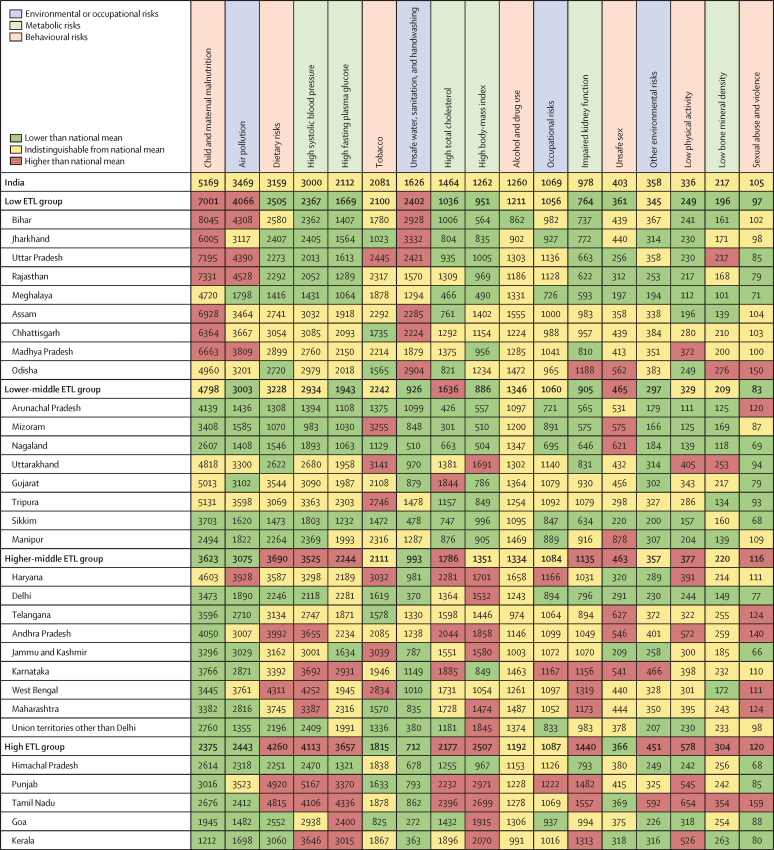
All-age DALY rates attributable to risk factors in the states of India, 2016 DALY=disability-adjusted life-year. ETL=epidemiological transition level.

The all-age DALY rate due to child and maternal malnutrition increased with decreasing ETL, with a rate three-times higher in the low ETL group than in the high ETL state group (figure 7). The DALY rate due to unsafe water sanitation and handwashing was 3·4-times higher in the low than in the high ETL state group. The DALY rate due to air pollution was also highest in the low ETL state group. In the low ETL group, the DALY rate due to outdoor ambient air pollution was 1·5-times higher and the rate due to household air pollution was 2·6-times higher than the high ETL state group. By contrast, the DALY rates due to dietary risks, high systolic blood pressure, high fasting plasma glucose, high total cholesterol, high body-mass index, and impaired kidney function were higher in the high ETL state group, with ratios of 1·7–2·6 between the rates in the high versus low ETL groups. The DALY rate due to tobacco was lower in the high ETL state group than in the other ETL groups. Some significant variations were recorded within a group between the states. For example, within the high ETL state group, Himachal Pradesh had much lower DALY rates due to dietary risks, high systolic blood pressure, high fasting plasma glucose, high total cholesterol, high body-mass index, and impaired kidney function than most of the other states, and Goa too had a significantly lower DALY rate due to dietary risks than most of the other states. Age-standardised DALY rates due to each risk factor in the states of India are shown in the appendix (p 140).

**Figure 8:**
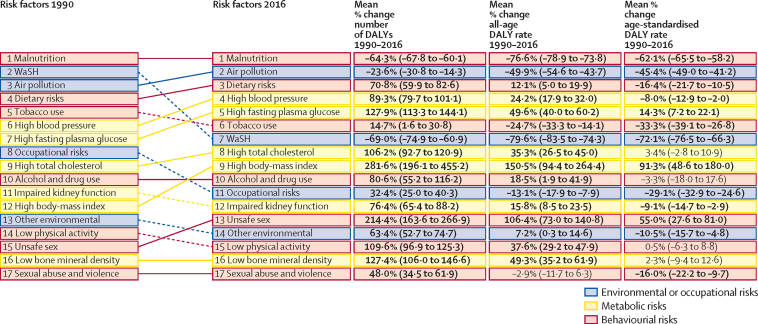
Change in DALYs attributable to risk factors and percent change in rates between 1990 and 2016 in India DALY=disability-adjusted life-year. Malnutrition=child and maternal malnutrition. WaSH=unsafe water, sanitation, and handwashing.

Child and maternal malnutrition mainly contributed to DALYs from neonatal disorders, nutritional deficiencies, and diarrhoea, lower respiratory, and other common infectious diseases (figure 6). These DALYs decreased by 64·3% (95% UI 67·8–60·1) from 1990 to 2016, but child and maternal malnutrition was still the top risk factor, causing the highest disease burden in India in 2016 as it was in 1990, when it caused 35·5% of the DALYs (figure 8). For individual risks under child and maternal malnutrition, the SEV of child wasting decreased by 27·2% (30·3–24·4) from 1990 to 2016 in India (table 3). The SEVs of related individual risks also decreased, for child stunting by 32·7% (95% UI 29·6–36·6) and child underweight by 43·4% (40·0–47·0) from 1990 to 2016 in India (data not shown because their contributions to total DALYs were <2%). The smallest decrease was in the low ETL state group for child stunting, and the greatest decrease was in the low ETL group for child wasting. Negligible changes were noted in the SEVs of short gestation, low birthweight, and iron deficiency between 1990 and 2016 across all of the ETL state groups.

**Table 3:** Change in summary exposure value of individual leading risk factors in states grouped by ETL from 1990 to 2016

	**Percentage contribution to DALYs in India, 2016**	**Summary exposure value 2016**					**Percentage change from 1990 to 2016**				
		Low ETL group (0·56–0·75)	Lower-middle ETL group (0·41–0·55)	Higher-middle ETL group (0·31–0·40)	High ETL group (<0·31)	India	Low ETL group (0·56–0·75)	Lower-middle ETL group (0·41–0·55)	Higher-middle ETL group (0·31–0·40)	High ETL group (<0·31)	India
**Child and maternal malnutrition**											
Short gestation for birthweight	6·5	11·6 (10·8 to 12·7)	12·2 (11·3 to 13·3)	12·3 (11·4 to 13·4)	12·0 (11·2 to 13·1)	11·9 (11·1 to 13·0)	2·3 (0·9 to 4·2)	3·5 (2·3 to 5·0)	2·3 (1·0 to 3·8)	4·0 (2·5 to 5·8)	2·5 (1·3 to 4·1)
Iron deficiency	3·5	14·9 (11·7 to 18·7)	13·9 (10·9 to 17·4)	13·9 (10·8 to 17·5)	13·0 (10·1 to 16·4)	14·3 (11·1 to 18·0)	1·4 (1·1 to 1·7)	0·9 (0·4 to 1·3)	0·9 (0·7 to 1·1)	−0·2 (−0·7 to 0·2)	1·3 (1·1 to 1·6)
Low birthweight for gestation	3·4	8·7 (7·9 to 9·8)	8·4 (7·8 to 9·2)	8·3 (7·7 to 9·1)	8·3 (7·7 to 9·0)	8·5 (7·8 to 9·4)	−0·6 (−1·5 to 0·3)	−1·7 (−2·6 to −0·9)	−1·9 (−2·9 to −1·1)	−1·5 (−2·1 to −0·8)	−1·1 (−1·8 to −0·4)
Child wasting	3·3	8·6 (7·3 to 9·7)	9·7 (8·2 to 11·0)	9·8 (8·3 to 10·9)	9·1 (7·7 to 10·4)	9·1 (7·8 to 10·3)	−31·5 (−36·3 to −27·2)	−22·1 (−30·1 to −13·8)	−25·5 (−29·8 to −21·2)	−17·5 (−24·1 to −10·9)	−27·2 (−30·3 to −24·4)
**Unsafe water, sanitation, and handwashing**											
Unsafe water source	3·3	37·9 (30·6 to 41·8)	24·0 (17·1 to 29·2)	28·5 (21·5 to 32·1)	23·0 (14·0 to 27·3)	32·0 (24·8 to 35·5)	−11·0 (−15·5 to −6·7)	−30·9 (−41·4 to −17·9)	−22·3 (−27·6 to −16·8)	−28·4 (−36·5 to −20·1)	−16·9 (−20·1 to −13·7)
Unsafe sanitation	2·5	59·8 (56·7 to 63·2)	35·8 (30·2 to 42·0)	38·3 (33·0 to 45·1)	28·8 (24·6 to 33·3)	47·3 (44·2 to 51·2)	−31·8 (−35·4 to −28·5)	−54·7 (−61·2 to −47·8)	−53·0 (−58·9 to −46·9)	−62·9 (−67·9 to −57·6)	−43·5 (−46·2 to −40·6)
**Air pollution**											
Ambient particulate matter pollution	6·4	76·9 (63·0 to 88·3)	62·3 (51·6 to 75·8)	63·4 (53·7 to 74·3)	49·8 (41·5 to 59·4)	68·2 (57·1 to 79·3)	14·9 (8·9 to 17·5)	18·3 (13·6 to 23·2)	20·6 (14·7 to 25·9)	2·3 (−2·7 to 7·8)	16·6 (11·1 to 20·4)
Household air pollution from solid fuels	4·8	38·3 (31·3 to 46·4)	19·3 (14·9 to 24·3)	19·4 (15·7 to 23·8)	12·1 (9·3 to 15·4)	27·6 (22·5 to 33·5)	−40·0 (−45·3 to −35·4)	−62·3 (−68·5 to −55·6)	−63·3 (−67·2 to −59·1)	−77·0 (−81·1 to −73·1)	−52·2 (−55·7 to −49·1)
**Metabolic risks**											
High systolic blood pressure	8·5	22·3 (20·9 to 24·0)	24·0 (22·5 to 25·9)	23·6 (22·1 to 25·4)	25·8 (24·1 to 27·8)	23·3 (21·8 to 25·0)	0·7 (−0·3 to 1·7)	5·9 (4·1 to 7·7)	7·0 (5·9 to 8·1)	10·6 (9·1 to 12·0)	4·2 (3·6 to 4·9)
High fasting plasma glucose	6·0	2·8 (1·6 to 4·1)	2·8 (1·6 to 4·3)	3·6 (2·2 to 5·4)	6·2 (4·1 to 8·9)	3·5 (2·1 to 5·0)	42·8 (18·5 to 83·1)	53·3 (24·3 to 99·3)	32·5 (16·3 to 56·4)	49·4 (20·3 to 87·8)	37·2 (20·6 to 62·1)
High total cholesterol	4·1	9·7 (7·0 to 12·9)	12·4 (9·1 to 16·2)	12·7 (9·5 to 16·5)	15·5 (11·8 to 19·9)	11·5 (8·6 to 15·2)	11·0 (6·7 to 15·9)	17·2 (10·5 to 24·7)	19·2 (14·2 to 24·3)	21·5 (15·7 to 27·8)	14·8 (11·4 to 18·5)
High body-mass index	3·6	3·8 (2·6 to 5·4)	4·0 (2·7 to 5·8)	5·5 (3·8 to 8·0)	7·2 (5·0 to 10·1)	4·8 (3·3 to 6·8)	86·1 (64·7 to 109·6)	114·8 (77·7 to 155·3)	143·2 (112·2 to 174·8)	181·1 (140·3 to 227·5)	119·3 (99·1 to 140·5)
Impaired kidney function	2·8	4·8 (2·7 to 10·1)	4·8 (2·7 to 10·2)	5·3 (3·0 to 10·7)	5·4 (3·2 to 10·6)	5·0 (2·9 to 10·4)	4·9 (−2·1 to 9·5)	4·7 (−4·0 to 10·2)	4·5 (−4·7 to 10·0)	3·3 (−6·9 to 10·7)	4·1 (−3·9 to 9·0)
**Dietary risks**											
Diet low in fruits	2·8	74·5 (53·6 to 92·0)	83·0 (63·5 to 95·5)	72·4 (53·1 to 87·0)	62·5 (44·4 to 77·6)	73·0 (53·6 to 88·6)	−16·3 (−23·5 to −7·4)	−9·4 (−14·5 to −4·2)	−16·7 (−22·2 to −10·1)	−22·8 (−29·8 to −14·8)	−16·4 (−22·2 to −9·1)
Diet low in nuts and seeds	2·3	88·4 (67·6 to 99·3)	90·2 (70·1 to 99·8)	65·2 (46·4 to 80·7)	51·3 (34·9 to 65·5)	76·4 (56·9 to 89·0)	−3·7 (−6·1 to −0·7)	−0·7 (−1·8 to 0·5)	−23·8 (−29·2 to −17·0)	−30·2 (−36·9 to −22·7)	−12·2 (−15·2 to −8·4)
**Tobacco use**											
Smoking	4·5	9·0 (7·4 to 11·8)	10·6 (8·8 to 13·4)	9·3 (7·7 to 11·9)	7·2 (5·5 to 9·9)	9·0 (7·5 to 11·7)	−21·1 (−31·6 to −6·4)	−13·4 (−25·6 to −1·4)	−22·0 (−29·8 to −14·1)	−37·9 (−48·0 to −25·9)	−22·9 (−29·7 to −14·8)
**Alcohol and drug use**											
Alcohol use	2·9	3·2 (2·3 to 4·0)	3·6 (2·5 to 4·7)	4·0 (3·1 to 5·0)	4·4 (3·1 to 5·8)	3·6 (2·9 to 4·3)	70·6 (20·0 to 146·2)	75·0 (14·0 to 156·5)	86·8 (35·9 to 155·6)	95·4 (21·9 to 203·2)	76·3 (42·4 to 117·4)

Individual risk factors that contributed to more than 2% of disability-adjusted life-years in India in 2016 are included. DALY=disability-adjusted life-year. ETL=epidemiological transition level.

Unsafe water, sanitation, and handwashing contributed to DALYs from diarrhoeal diseases and other infections (figure 6). These types of DALYs decreased by 69·0% (95% UI 74·9–60·9) from 1990 to 2016, with their ranking changing from second to seventh leading cause of DALYs (figure 8). The SEV of unsafe sanitation decreased by 43·5% (95% UI 46·2–40·6) and that of unsafe water source decreased by 16·9% (20·1–13·7) from 1990 to 2016 in India (table 3); the smallest decreases were in the low ETL state group for both risk factors.

Air pollution mainly contributed to disease burden from cardiovascular disease, chronic respiratory disease, and lower respiratory infections (figure 6). DALYs due to air pollution decreased by 23·6% (95% UI 30·8–14·3) in India from 1990 to 2016, mainly due to reduction in household air pollution (figure 8). The SEV of ambient air pollution increased by 16·6% in India during this period, with increases in all ETL state groups except the high ETL group (table 3). The SEV of household air pollution decreased by 40% (95% UI 45·3–35·4) in the low ETL state group and 77% (81·1–73·1) in the high ETL group between 1990 and 2016.

Metabolic risks such as high systolic blood pressure, high fasting plasma glucose, high total cholesterol, and high body-mass index, along with dietary risks, which are predominantly associated with cardiovascular disease and diabetes, together were responsible for 15·9% and 8·9% of the DALYs in India in 2016, respectively, as compared with 7·0% and 4·5% of the DALYs in 1990 (data not shown). The number of DALYs and the all-age DALY rates from each of these risk factors increased in India from 1990 to 2016 (figure 8). The SEV of high systolic blood pressure did not significantly change in the low ETL state group between 1990 and 2016, but increased 5·9–10·6% in the other ETL state groups (table 3). The SEV of high fasting plasma glucose increased across all the ETL state groups by 32·5–53·3%. The SEV of high total cholesterol increased by 11·0% (95% UI 6·7–15·9) in the low ETL state group and by 21·5% (15·7–27·8) in the high ETL state group. Large increases were recorded in the SEV of high body-mass index, ranging from 86·1% (95% UI 64·7–109·6) in the low ETL state group to 181·1% (140·3–227·5) in the high ETL state group. For the two leading individual risks under dietary risks, diet low in fruits and diet low in nuts and seeds, the 2016 SEVs were quite high (table 3). A modest reduction in these SEVs was recorded between 1990 and 2016, and the reduction was greatest in the high ETL state group.

The all-age and age-standardised DALY rates due to tobacco use decreased in India from 1990 to 2016, but tobacco still contributed 8·3% of total DALYs in men and 3·0% in women (Figure 6, Figure 8). The SEV of smoking decreased in India during this period by 22·9% (95% UI 29·7–14·8), with the highest individual group decrease of 37·9% (48·0–25·9) in the high ETL state group (table 3).

Life expectancy at birth in India was 66·9 years (95% UI 66·2–67·6) for men and 70·3 years (69·6–71·0) for women in 2016, an increase of 8·6 years (7·8–9·5) for men and 10·6 (9·7–11·6) for women since 1990 (appendix p 129). The difference between the life expectancy increases for men and women was least for the high ETL state group (0·8 years greater increase for women than for men); for the other state groups this difference ranged from 1·9 years to 2·5 years. In 2016, the life expectancy of women was 4·9 years more than that of men in the high ETL state group, and 2·0 years more in the low ETL state group.

## Discussion

The age-standardised DALY rate in India dropped by 36% from 1990 to 2016, indicating overall progress in reducing disease burden. Behind this, however, are huge variations in the magnitude and progress across the states of India for the various diseases and risk factors. We offer insights into the challenges that need to be addressed to more effectively improve health across one of the most populous countries in the world. The NITI Aayog has articulated a progressive action agenda for improving health in the country from 2017 to 2020, which includes data-driven and decentralised health planning that is focused on the specific needs of each state.^16^ The state-level disease burden and risk factor estimates reported by the India State-level Disease Burden Initiative can serve as a crucial aid in this health-planning approach suggested by the premier thinktank of the Government of India.

Eight north Indian states that have low development indicators, which are referred to as the EAG states, along with the eight northeastern states and the two states of Himachal Pradesh and Jammu and Kashmir, have been the focus since 2005 of the National Rural Health Mission, which was renamed the National Health Mission in 2013.^11^ The findings in this paper show that generally, the EAG states have the lowest ETLs, followed by the north-eastern states, and then the others. However, some exceptions to this trend do exist, and significant variations were seen in the distribution of diseases and risk factors within these state groups that should be considered while planning health improvements in each state. In this Article we present the trends in disease burden and risk factors for states grouped by level of epidemiological transition as well as the key findings for individual states. The India State-level Disease Burden Initiative policy report, which is being released Nov 14, 2017, provides detailed findings for individual states, including a profile of each state.^24^ These granular findings are expected to better define the health inequalities between the states, thereby leading to more focused attention on addressing these inequalities. Our findings can provide important inputs into how to fine tune in each state the components of the National Health Assurance efforts that the Government of India has undertaken.^25^ India's 2017 National Health Policy has set out a series of disease-reduction targets.^14^ Monitoring the trends across the states with robust findings is crucial to understand where more effort is needed to meet the national targets.

The epidemiological transition ratio (DALYs due to CMNNDs *vs* NCDs and injuries combined) ranged from 0·16 for Kerala to 0·74 for Bihar in 2016, a greater than four-times difference. The transition of disease epidemiology in India towards a dominance of NCDs and injuries from 1990 to 2016 is remarkable, with all states having a higher disease burden from NCDs and injuries than CMNNDs in 2016, in contrast to 1990, when the majority of disease burden in most states was due to CMNNDs. NCDs and injuries became the contributor to the majority of overall disease burden for India in 2003; but this event occurred from 1986 to 2010 for the four ETL state groups. The large low and higher-middle ETL state groups with 48% and 34% of India's total population in 2016, contributed more to the overall India trend than the other two smaller ETL groups. The epidemiological transition ratio had a significant inverse relation with SDI, but the slope of this association had reduced by about half from 1990 to 2016, indicating reducing differences with increasing SDI over time. We stratified states by epidemiological transition ratio because we were interested in understanding disease and risk factor variations between and within the epidemiological transition levels. Another approach that could offer additional insights would be to assess variations by SDIs of the states.

Although the burden of CMNNDs has dropped substantially across all ETL state groups in India from 1990 to 2016, the ratio of the observed to expected DALY rate for the SDI level of India is quite high for most of these diseases, indicating that India suffers a disproportionately higher burden of these diseases than other parts of the world with similar SDIs. The DALY rates due to the leading CMNNDs continue to be much higher in the low and lower-middle ETL states, showing the need for greater efforts in reducing the burden due to lower respiratory diseases, diarrhoeal diseases, neonatal disorders, iron-deficiency anaemia, and tuberculosis in these states.

The high neonatal and under-5 disease burden relative to other age groups, predominantly due to the leading CMNNDs, continues to be a major priority for India. Intensive efforts to reduce this burden are necessary to meet the Sustainable Development Goals targets in 2030.^26^ India has adopted the Newborn Action Plan, which is in synchrony with the Global Every Newborn Action Plan, focusing on 187 priority districts.^27^, ^28^ Remarkably, child and maternal malnutrition continues to be the leading risk factor in India, responsible for 15% of total DALYs in 2016; and unsafe water, sanitation, and handwashing still causes 5% of total DALYs in India. This trend continues despite major programmes in India for several decades to address these risk factors. The Government programme, Integrated Child Development Services, was launched in 1975 to provide supplementary nutrition, nutrition and health education, and other preschool development services across India; it had a total annual budget of more than US$2 billion in the 2015–16 fiscal year.^29^ The Mid Day Meal Scheme, launched by the Government of India in 1995, provides free lunch to more than 120 million primary and upper-primary school children and has an annual public expenditure of more than US$2 billion.^30^ In 2013, the Government of India legislated the National Food Security Act with the objective of providing food and nutritional security to the country's population through provision of subsidised food grains and focused nutritional support to women and children.^31^ The Rural Sanitation Programme was launched by the Government of India in 1986, and has had several transformations since; the current more elaborate version is the Clean India Mission (Swachh Bharat Abhiyan), which was launched in 2014 by the Prime Minister of India as a major campaign to clean India and eliminate open defecation, with an estimated cost of about US$30 billion over 5 years.^32^, ^33^ Notably, the burden of these risk factors continues to be the highest in the states with lower ETLs, with Bihar having the highest DALY rate in India due to child and maternal malnutrition and Jharkhand due to unsafe water, sanitation, and handwashing. Although some improvements have been seen in overall nutrition, the exposure to iron deficiency in India has not improved much, though interventions for this risk have been attempted.^34^ An important challenge that needs to be addressed for a higher impact of interventions is efforts at behavioural change along with provision of better nutrition, safe water, and safe sanitation for higher uptake by those who need these most.^35^

India has the highest tuberculosis burden among the countries of the world, with a DALY rate more than three-times higher than can be explained by its SDI level.^36^ India has scaled up basic tuberculosis services in the public health system, but the rate of decline in tuberculosis seems too slow to meet the 2030 Sustainable Development Goals and the 2035 End TB targets.^26^, ^36^, ^37^ Major challenges have been delayed detection and treatment of tuberculosis, inadequate surveillance, poor notification, and absence of coordination with the private health-care sector. A National Strategic Plan for Tuberculosis Elimination was announced in 2017 by India's Revised National Tuberculosis Control Programme to achieve a 10–15% annual decline in the incidence of tuberculosis; this plan is estimated to cost US$2·5 billion over 5 years.^36^ The burden of tuberculosis varies markedly across the states of India, with the DALY rate of the highest-burden state at seven times that of the lowest-burden state. Control or elimination of malaria and some neglected tropical diseases, including visceral leishmaniasis, lymphatic filariasis, and leprosy have also been specified by the National Healthy Policy 2017 and NITI Aayog action agenda as priorities.^14^ The estimates produced by the India State-level Disease Burden Initiative could be a useful reference for titrating the efforts according to the diverse epidemiology of tuberculosis and neglected tropical diseases across the states of India.

The all-age prevalence of most leading NCDs increased substantially in India from 1990 to 2016, but the age-standardised prevalence increased only for diabetes, cerebrovascular disease, ischaemic heart disease, and skin diseases. This trend implies that the overall increase in NCD prevalence in India is a mixed phenomenon, with ageing of the population responsible for the increase in many NCDs plus an additional increase due to changes in risk exposure for the causes that have an age-standardised increase in prevalence. From 1990 to 2016, all ETL state groups had a substantial increase in the number of NCD DALYs and no significant change in the all-age DALY rate but a significant modest decrease in the age-standardised DALY rates. These results imply that the improving health interventions in India have started blunting the NCD DALY burden to some degree. However, the interventions need to be greatly enhanced to achieve steeper declines in both the prevalence of DALYs and DALY rates from all NCDs. In 2016, the observed DALY rate in India exceeded the rate expected for its SDI level for several leading NCDs, namely ischaemic heart disease, COPD, sense organ disease (mainly vision and hearing loss disorders), migraine, asthma, and rheumatic heart disease.

The trajectory of the major risk factors for ischaemic heart disease, cerebrovascular disease, and diabetes has been on the rise across all ETL state groups in India. Dietary risks, high systolic blood pressure, high fasting plasma glucose, high total cholesterol, and high body-mass index together contributed about a quarter of the DALYs in India in 2016, which is more than twice their contribution in 1990. Tobacco use contributed 6% of DALYs in India in 2016. Intervention planning for major NCDs has picked up in India over the past decade or so. The National Programme for Prevention and Control of Cancer, Diabetes, Cardiovascular Diseases and Stroke was launched in India in 2010.^38^ The Government of India enacted the Cigarettes and Other Tobacco Products Act in 2003 to discourage the use of tobacco products, and the National Tobacco Control Programme was launched in 2007.^39^ The National Mental Health Programme has been in place in India since 1982,^40^ and the Mental Health Care Act was enacted in 2017.^41^ Although these national programmes and legislative acts indicate the interest of the Government of India in controlling the increasing burden of NCDs, the absence of strong declining trends for the prevalence of DALYs and DALY rates of most NCDs suggest that progress in the control of NCDs in India needs a bigger and more organised effort, supported by commensurate financial and human resources. These efforts would have to include extensive intersectoral collaborations, because many of the interventions needed for the control of NCDs go beyond the traditional health sector. The recent National Health Policy 2017 and the NITI Aayog action agenda have set targets for reduction of premature death and morbidity due to major NCDs in India.^14^, ^16^ Monitoring of this progress would be aided by the ongoing production of reliable state-level estimates of disease burden and risk factors.

Exposure to air pollution in India is among the highest in the world,^22^ contributing to both NCDs and communicable diseases. Disease burden due to air pollution is highest in the low ETL state group, with Rajasthan, Uttar Pradesh, and Bihar having the highest DALY rates. The burden from household air pollution is on the decline across all ETL state groups in India on account of the decreasing use of solid fuels for cooking. However, this decline was least in the low ETL group, suggesting that targeted subsidies to accelerate the transition to clean fuels is warranted. A recent initiative by the Prime Minister of India, the Pradhan Mantri Ujjwala Yojna, is expected to further increase access to clean cooking gas for households that are below the poverty line.^42^ However, the burden attributable to ambient air pollution continues to pose substantial challenges, because the current trajectory of emissions and dust from the power, industrial, transport, and construction sectors is likely to contribute to continuing increases in exposure across all ETL state groups. Policies are needed that effectively help increase the use of technologies that produce less emissions and dust in the sectors that are contributing to ambient air pollution. Enhanced monitoring of particulate matter smaller than 2·5 μm at more sites across India by the Ministry of Environment and by the Ministry of Earth Sciences is expected to facilitate more granular understanding of air pollution trends.^43^, ^44^

The number of DALYs caused by injuries increased significantly from 1990 to 2016 in all ETL state groups except the high ETL group, while both the all-age and age-standardised DALY rates for injuries decreased across all ETL state groups, indicating that the increase in number of DALYs was due to increase in population size. The all-age and age-standardised incidence rate of road injuries increased substantially during this period, and the all-age rates of self-harm and falls increased modestly but the age-standardised rates did not. The ratio of the observed DALY rate in India to the rate expected for its SDI level was close to two for self-harm and falls. However, injuries have typically received very little attention from policy makers and researchers in India.^45^, ^46^ India does not have a comprehensive policy for injury prevention, and the multisectoral nature of interventions needed for the control of injuries is not addressed adequately. A National Road Safety Policy under the Ministry of Road Transport and Highways was announced in 2010, and the Ministry of Health has a capacity-building programme for trauma care facilities on the national highways, which have been implemented to varying extents.^47^, ^48^ The National Highways Authority of India announced plans in 2017 to provide more prompt trauma care on highways.^49^ However, a comprehensive road injuries prevention and care approach is needed with balanced attention to safe road infrastructure, safe use of roads with enforcement, and appropriate trauma care. The multitude of reasons contributing to self-harm mean that the social determinants of self-harm must be addressed more effectively and better preventive mental health services should be provided.^50^, ^51^, ^52^ Prevention of falls and adequate management to reduce their population-level burden requires a systematic effort in India that currently does not exist. Falls could be made a specific focus in the National Programme for Health Care of the Elderly.^53^

Notably, the estimated risk factors explain only about half of the disease burden in India, pointing to the need for enhancing the understanding of additional broader determinants of health.^54^, ^55^ Two factors that will pose major challenges to the Indian health system over the next few decades are urbanisation and ageing of the population. Increasing unplanned urbanisation is a major challenge in India, with half of the population projected to be urban by 2050, up from a third at present.^56^ With increasing life expectancy and reducing premature mortality, the contribution of YLDs (disability) to the total DALYs (disease burden) will continue to increase. Long-term policy responses to these ongoing major transitions will be needed as part of comprehensive health planning for the states of India.

The main strengths of the findings presented in this paper are the following: (1) extensive efforts were made to identify, access, and use all available data that could contribute to the estimates for each state of India; (2) standardised GBD methods were used; and (3) a large network of leading health scientists and policy makers from India contributed to the analysis and interpretation of the estimates. The limitations of the findings include the general limitations of the GBD approach that are described elsewhere.^18^, ^19^, ^20^, ^21^, ^22^ Other limitations were specific to the India findings. First, India does not have an adequately functional cause-of-death reporting system. The Medical Certification of Cause of Death (MCCD) system under the Office of the Registrar General of India covered only 22% of the deaths in India in 2015, with the coverage less than 20% in 15 states, 20–50% in ten states and union territories, and more than 50% in some states and union territories with less than 3% of India's population.^57^ SRS provides cause-of-death data for all states in India using verbal autopsy. Verbal autopsy is considered a reasonable alternative for cause-of-death data when these data are not adequately available from the vital registration system.^58^, ^59^, ^60^ Although the SRS cause-of-death data for the years 2004–13 were very useful for the state-level disease burden estimates in this paper, a long-term plan and investment is needed to improve the coverage and quality of the MCCD system in India for more robust cause-of-death data. Second, disaggregated data for estimation of state-level population disease morbidity were scarce for some major conditions, including musculoskeletal disorders, chronic kidney disease, chronic respiratory diseases, cerebrovascular disease, and mental health and substance abuse disorders. When data are scarce for a disease or risk factor, GBD uses covariates and techniques that borrow strength from proximity and over time to arrive at the best possible estimates. Third, data on some risk factors were sparse across the states, including urban dietary intake and drug use at the population level. Findings released in 2017 from a multistate urban diet survey will enhance the estimates in the next GBD cycle. Fourth, GBD does not separately estimate the burden of Japanese encephalitis, chikungunya, and fluorosis. These conditions are important for India, and future GBD cycles are expected to estimate these. Broadly, India needs to systematically develop a comprehensive health information system that can provide adequate data for ongoing and reliable mortality, morbidity, and risk factor estimation at suitable levels of geographic disaggregation, a notion supported by both the recent NITI Aayog action agenda and the National Health Policy. Details of the data gaps identified as part of the work of the India State-level Disease Burden Initiative can be used to inform development of an adequate health information system in India.

In conclusion, this analysis of epidemiological transition, disease burden, and risk factors across the states of India from 1990 to 2016 is perhaps the most comprehensive attempt so far to understand the entire landscape of disease epidemiology in India. The findings presented for groups of states at similar ETLs and for individual states can provide crucial and robust disaggregated inputs for steering health policy in India to improve population health in each state and union territory of the country. The ongoing work of the India State-level Disease Burden Initiative could be a useful tool for NITI Aayog's recently articulated vision of transforming health services and health outcomes in India over the next 15 years and for tracking progress in the goals and targets set by the National Health Policy 2017.^14^, ^16^ To achieve its optimal development potential, India should improve the health and nutritional status of its people in earnest now, investing more resources in social sectors as a result of its continuing impressive economic progress and using the increasing understanding of health heterogeneity across the country in a manner that reduces the major health inequalities between the nations within this nation, which comprises almost a fifth of the world's population.

Correspondence to: Prof Lalit Dandona, Public Health Foundation of India, Gurugram 122002, National Capital Region, India **lalit.dandona@phfi.org**

**This online publication has been corrected. The corrected version first appeared at thelancet.com on November 30, 2017**
