# Department of Error

**DOI:** 10.1016/S0140-6736(17)33053-2

**Published:** 2017-12-02

*India State-Level Disease Burden Initiative Collaborators. Nations within a nation: variations in epidemiological transition across the states of India, 1990-2016 in the Global Burden of Disease Study.* Lancet *2017;* **390:** *2437–60—*In this Article (published online first on Nov 13, 2017), changes have been made to the list of India State-Level Disease Burden Initiative Collaborators and affiliations. This correction has been made to the online version as of Nov 30, 2017, and the printed Article is correct.

