# Supplementary material for: Nations within a nation: variations in epidemiological transition across the states of India, 1990–2016 in the Global Burden of Disease Study
Source: Lancet. 2017 Dec 2;390(10111):2437–60. doi: 10.1016/S0140-6736(17)32804-0 (PMC5720596; doi:10.1016/S0140-6736(17)32804-0)
Supplement: Supplementary appendix [file mmc1.pdf]

THE LANCET

Supplementary appendix

This appendix formed part of the original submission and has been peer reviewed. We post it as supplied by the authors.

Supplement to: India State-level Disease Burden Initiative Collaborators. Nations within a nation: variations in epidemiological transition across the states of India, 1990–2016 in the Global Burden of Disease Study. *Lancet* 2017; published online Nov 14. http://dx.doi.org/10.1016/S0140-6736(17)32804-0.

Nations within a nation: variations in epidemiological transition across the states of India, 1990–2016 in the Global Burden of Disease Study

India State-level Disease Burden Initiative Collaborators

Web Appendix

Published online 14 November 2017

Correspondence to: Prof Lalit Dandona, lalit.dandona@phfi.org

Road map of the India State-level Disease Burden Initiative ………………………... 4

Table 1: Global Burden of Disease Study 2016 cause list…………… ……………..… 5

Table 2: GBD 2016 risk factors list ……………………………………………………. 12

Table 3: GBD 2016 India data input list.....……………………………………...… 14

Table 4: Percent change in epidemiological transition ratio for the states of India from 1990 to 2016………………………………………..…………………………..... 122

Table 5: Change in all-age and age-standardised death rates in the states of India from 1990 to 2016…………………… .....……………………….…………..……………….…. 123

Table 6: Percent contribution of disease categories to total DALYs in states grouped by epidemiological transition level in 1990 and 2016……………..……………...…….. 124

Table 7: Percent change of DALY number and rates for major disease groups in states grouped by epidemiological transition level from 1990 to 2016...………………......… 125

Table 8: Comparison of the percent change in prevalence of NCDs and incidence rate of injuries with the percent change in their DALY rate from 1990 to 2016, India..........… 126

Table 9: Ratio of the observed to expected DALY rate based on SDI for the top 30 causes in states grouped by epidemiological transition level, 2016...…………….…. 127

Table 10: Change in all-age and age-standardised DALY rates in each state of India from 1990 to 2016...………………………………………...……………………..… 128

Table 11: Change in life expectancy in the states of India from 1990 to 2016. ………….. 129

Figure 1: Association between epidemiological transition ratio and socio-demographic index for the states of India, 1990 and 2016……..……….…………………….....…. 130

Figure 2: Change in death number and percent change in rates for the top 30 causes from 1990 to 2016, India ……………………..………………………………….…... 131

Figure 3: Change in DALYs number and percent change in rates for the top 30 causes from 1990 to 2016, men and women, India…………….………………………….……… 132

Figure 4: Age-standardised DALY rates of the top 30 causes in the states of India, 2016…………………………………………………………………….......……… 134

Figure 5: Range of all-ages DALY rates across the states of India for the top 10 causes, 2016…………………………………………………………………...… 135

Figure 6: Percent of total DALYs by age group in India, 1990…...…………………..…. 136

Figure 7: Contribution of YLLs and YLDs to DALYs in states grouped by epidemiological transition level, 1990 and 2016……………………………..……... 137

Figure 8: Comparison of top 30 causes of DALYs, YLLs and YLDs in India, 2016………………………………………………………………………… 138

Figure 9: Percent contribution of top 10 risk factors to DALYs in states grouped by epidemiological transition level in 2016………………………………………………. 139

Figure 10: Age-standardised DALY rates attributable to risk factors in the states of India, 2016………………………….……………………………………………. 140

Road map of the India State-level Disease Burden Initiative

- The India State-level Disease Burden Initiative is a collaboration between the Indian Council of Medical Research, Public Health Foundation of India, Institute for Health Metrics and Evaluation at the University of Washington, and experts and stakeholders currently from close to 100 institutions across India, which was launched in October 2015.
- The Advisory Board of this Initiative is chaired by a former Health Secretary to the Government of India, and includes some of the leading health policy makers of the country. This Initiative has fourteen domain expert groups that are closely involved with the estimation process and interpretation of findings.
- The goal of this initiative is to produce the best possible state-level disease burden and risk factors trends 1990 onward as part of the Global Burden of Disease Study, utilizing all identifiable epidemiological data from India and in close engagement with the leading health scientists of India.
- The first comprehensive set of state-level disease burden and risk factors estimates are being disseminated in this technical paper and as a policy report to be presented to the Government of India and the state governments on 14 November 2017. An open access interactive visualization tool that will bring to life in an easily understandable manner the disease burden and risk factors trends over time across the Indian states is also being made available.
- More detailed topic-specific publications and policy reports will be produced in 2018 for major diseases and risk factors for further granular insights to plan their control.
- Extensive engagement with central and state-level policy makers is anticipated for utilization of the estimates. The major anticipated utilization of findings to inform policy include:
    - Planning of state health budgets
    - Prioritization of interventions relevant to each state
    - Inform the government's Health Assurance Mission in each state
    - Monitoring of health-related Sustainable Development Goals targets in each state
    - Assessing impact of large-scale interventions based on time trends of disease burden
    - Forecasting population health under various scenarios in each state
- The data gaps identified in the estimation process will be articulated systematically to inform enhancement of the health information system of India.
- Annual production of state-level disease burden estimates is planned, with estimates improving with increasing availability of data.
- Additional disaggregation of estimates is planned, for example, rural-urban estimates for each state next year and geospatial mapping at a fine-grid level for key diseases and risk factors.
- Capacity building in India to generate and analyse large-scale health data using strong methods is anticipated over the next five years of this work.

Table 1: GBD 2016 cause list	Cause of death	Cause of disability
**Communicable, maternal, neonatal, and nutritional diseases**	X	X
**HIV/AIDS and tuberculosis**	X	X
Tuberculosis	X	X
Drug-sensitive tuberculosis	X	X
Multidrug-resistant tuberculosis without	X	X
Extensively drug-resistant tuberculosis	X	X
HIV/AIDS	X	X
Drug-sensitive HIV/AIDS - Tuberculosis	X	X
Multidrug-resistant HIV/AIDS - Tuberculosis	X	X
Extensively drug-resistant HIV/AIDS -	X	X
HIV/AIDS resulting in other diseases	X	X
**Diarrhea, lower respiratory, and other common infectious diseases**	X	X
Diarrheal diseases	X	X
Intestinal infectious diseases	X	X
Typhoid fever	X	X
Paratyphoid fever	X	X
Other intestinal infectious diseases	X	X
Lower respiratory infections	X	X
Upper respiratory infections	X	X
Otitis media	X	X
Meningitis	X	X
Pneumococcal meningitis	X	X
H influenzae type B meningitis	X	X
Meningococcal meningitis	X	X
Other meningitis	X	X
Encephalitis	X	X
Diphtheria	X	X
Whooping cough	X	X
Tetanus	X	X
Measles	X	X
Varicella and herpes zoster	X	X
**Neglected tropical diseases and malaria**	X	X
Malaria	X	X
Chagas disease	X	X
Leishmaniasis	X	X
Visceral leishmaniasis	X	X
Cutaneous and mucocutaneous leishmaniasis		X
African trypanosomiasis	X	X
Schistosomiasis	X	X
Cysticercosis	X	X
Cystic echinococcosis	X	X
Lymphatic filariasis		X
Onchocerciasis		X
Trachoma		X
Dengue	X	X
Yellow fever	X	X
Rabies	X	X
Intestinal nematode infections	X	X
Ascariasis	X	X
Trichuriasis		X
Hookworm disease		X

Legend
GBD level 1
GBD level 2
GBD level 3
GBD level 4

Table 1: GBD 2016 cause list	Cause of death	Cause of disability
Food-borne trematodiases		X
Leprosy		X
Ebola	X	X
Zika virus	X	X
Guinea worm disease		X
Other neglected tropical diseases	X	X
**Maternal disorders**	**X**	**X**
Maternal hemorrhage	X	X
Maternal sepsis and other maternal infections	X	X
Maternal hypertensive disorders	X	X
Maternal obstructed labor and uterine rupture	X	X
Maternal abortion, miscarriage, and ectopic pregnancy	X	X
Indirect maternal deaths	X	
Late maternal deaths	X	
Maternal deaths aggravated by HIV/AIDS	X	
Other maternal disorders	X	X
**Neonatal disorders**	**X**	**X**
Neonatal preterm birth complications	X	X
Neonatal encephalopathy due to birth asphyxia and	X	X
Neonatal sepsis and other neonatal infections	X	X
Hemolytic disease and other neonatal jaundice	X	X
Other neonatal disorders	X	X
**Nutritional deficiencies**	**X**	**X**
Protein-energy malnutrition	X	X
Iodine deficiency	X	X
Vitamin A deficiency		X
Iron-deficiency anemia	X	X
Other nutritional deficiencies	X	X
**Other communicable, maternal, neonatal, and nutritional diseases**	**X**	**X**
Sexually transmitted diseases excluding HIV	X	X
Syphilis	X	X
Chlamydial infection	X	X
Gonococcal infection	X	X
Trichomoniasis		X
Genital herpes		X
Other sexually transmitted diseases	X	X
Hepatitis	X	X
Acute hepatitis A	X	X
Hepatitis B	X	X
Hepatitis c	X	X
Acute hepatitis E	X	X
Other infectious diseases	X	X
**Non-communicable diseases**	**X**	**X**
**Neoplasms**	**X**	**X**
Lip and oral cavity cancer	X	X
Nasopharynx cancer	X	X
Other pharynx cancer	X	X
Esophageal cancer	X	X
Stomach cancer	X	X
Colon and rectum cancer	X	X
Liver cancer	X	X

Table 1: GBD 2016 cause list		
	Cause of death	Cause of disability
Liver cancer due to hepatitis B	X	X
Liver cancer due to hepatitis C	X	X
Liver cancer due to alcohol use	X	X
Liver cancer due to other causes	X	X
Gallbladder and biliary tract cancer	X	X
Pancreatic cancer	X	X
Larynx cancer	X	X
Tracheal, bronchus, and lung cancer	X	X
Malignant skin melanoma	X	X
Non-melanoma skin cancer	X	X
Non-melanoma skin cancer (squamous-cell	X	X
Non-melanoma skin cancer (basal-cell		X
Breast cancer	X	X
Cervical cancer	X	X
Uterine cancer	X	X
Ovarian cancer	X	X
Prostate cancer	X	X
Testicular cancer	X	X
Kidney cancer	X	X
Bladder cancer	X	X
Brain and nervous system cancer	X	X
Thyroid cancer	X	X
Mesothelioma	X	X
Hodgkin lymphoma	X	X
Non-Hodgkin lymphoma	X	X
Multiple myeloma	X	X
Leukemia	X	X
Acute lymphoid leukemia	X	X
Chronic lymphoid leukemia	X	X
Acute myeloid leukemia	X	X
Chronic myeloid leukemia	X	X
Other leukemia	X	X
Other neoplasms	X	X
Other neoplasms	X	X
**Cardiovascular diseases**	**X**	**X**
Rheumatic heart disease	X	X
Ischemic heart disease	X	X
Cerebrovascular disease	X	X
Ischemic stroke	X	X
Hemorrhagic stroke	X	X
Hypertensive heart disease	X	X
Cardiomyopathy and myocarditis	X	X
Myocarditis	X	X
Alcoholic cardiomyopathy	X	X
Other cardiomyopathy	X	X
Atrial fibrillation and flutter	X	X
Aortic aneurysm	X	
Peripheral artery disease	X	X
Endocarditis	X	X
Other cardiovascular and circulatory diseases	X	X
**Chronic respiratory diseases**	**X**	**X**

Table 1: GBD 2016 cause list	Cause of death	Cause of disability
Chronic obstructive pulmonary disease	X	X
Pneumoconiosis	X	X
Silicosis	X	X
Asbestosis	X	X
Coal workers pneumoconiosis	X	X
Other pneumoconiosis	X	X
Asthma	X	X
Interstitial lung disease and pulmonary sarcoidosis	X	X
Other chronic respiratory diseases	X	X
**Cirrhosis and other chronic liver diseases**	**X**	**X**
Cirrhosis and other chronic liver diseases due to	X	X
Cirrhosis and other chronic liver diseases due to	X	X
Cirrhosis and other chronic liver diseases due to alcohol	X	X
Cirrhosis and other chronic liver diseases due to other	X	X
**Digestive diseases**	**X**	**X**
Peptic ulcer disease	X	X
Gastritis and duodenitis	X	X
Appendicitis	X	X
Paralytic ileus and intestinal obstruction	X	X
Inguinal, femoral, and abdominal hernia	X	X
Inflammatory bowel disease	X	X
Vascular intestinal disorders	X	X
Gallbladder and biliary diseases	X	X
Pancreatitis	X	X
Other digestive diseases	X	X
**Neurological disorders**	**X**	**X**
Alzheimer disease and other dementias	X	X
Parkinson disease	X	X
Epilepsy	X	X
Multiple sclerosis	X	X
Motor neuron disease	X	X
Migraine		X
Tension-type headache		X
Other neurological disorders	X	X
**Mental and substance use disorders**	**X**	**X**
Schizophrenia		X
Alcohol use disorders	X	X
Drug use disorders	X	X
Opioid use disorders	X	X
Cocaine use disorders	X	X
Amphetamine use disorders	X	X
Cannabis use disorders		X
Other drug use disorders	X	X
Depressive disorders		X
Major depressive disorder		X
Dysthymia		X
Bipolar disorder		X
Anxiety disorders		X
Eating disorders	X	X
Anorexia nervosa	X	X
Bulimia nervosa	X	X

Table 1: GBD 2016 cause list	Cause of death	Cause of disability
Autistic spectrum disorders		X
Autism		X
Asperger syndrome and other autistic spectrum		X
Attention-deficit/hyperactivity disorder		X
Conduct disorder		X
Idiopathic developmental intellectual disability		X
Other mental and substance use disorders		X
**Diabetes, urogenital, blood, and endocrine diseases**	**X**	**X**
Diabetes mellitus	X	X
Acute glomerulonephritis	X	X
Chronic kidney disease	X	X
Chronic kidney disease due to diabetes mellitus	X	X
Chronic kidney disease due to hypertension	X	X
Chronic kidney disease due to	X	X
Chronic kidney disease due to other causes	X	X
Urinary diseases and male infertility	X	X
Interstitial nephritis and urinary tract infections	X	X
Urolithiasis	X	X
Benign prostatic hyperplasia		X
Male infertility		X
Other urinary diseases	X	X
Gynecological diseases	X	X
Uterine fibroids	X	X
Polycystic ovarian syndrome	X	X
Female infertility		X
Endometriosis	X	X
Genital prolapse	X	X
Premenstrual syndrome		X
Other gynecological diseases	X	X
Hemoglobinopathies and hemolytic anemias	X	X
Thalassemias	X	X
Thalassemias trait		X
Sickle cell disorders	X	X
Sickle cell trait		X
G6PD deficiency	X	X
G6PD trait		X
Other hemoglobinopathies and hemolytic	X	X
Endocrine, metabolic, blood, and immune disorders	X	X
**Musculoskeletal disorders**	**X**	**X**
Rheumatoid arthritis	X	X
Osteoarthritis		X
Low back and neck pain		X
Low back pain		X
Neck pain		X
Gout		X
Other musculoskeletal disorders	X	X
**Other non-communicable diseases**	**X**	**X**
Congenital birth defects	X	X
Neural tube defects	X	X
Congenital heart anomalies	X	X
Cleft lip and cleft palate	X	X

Table 1: GBD 2016 cause list	Cause of death	Cause of disability
Down syndrome	X	X
Turner syndrome		X
Klinefelter syndrome		X
Other chromosomal abnormalities	X	X
Congenital musculoskeletal and limb anomalies	X	X
Urogenital congenital anomalies	X	X
Digestive congenital anomalies	X	X
Other congenital birth defects	X	X
Skin and subcutaneous diseases	X	X
Dermatitis		X
Psoriasis		X
Cellulitis	X	X
Pyoderma	X	X
Scabies		X
Fungal skin diseases		X
Viral skin diseases		X
Acne vulgaris		X
Alopecia areata		X
Pruritus		X
Urticaria		X
Decubitus ulcer	X	X
Other skin and subcutaneous diseases	X	X
Sense organ diseases		X
Glaucoma		X
Cataract		X
Macular degeneration		X
Refraction and accommodation disorders		X
Age-related and other hearing loss		X
Other vision loss		X
Other sense organ diseases		X
Oral disorders		X
Deciduous caries		X
Permanent caries		X
Periodontal diseases		X
Edentulism and severe tooth loss		X
Other oral disorders		X
Sudden infant death syndrome	X	
**Injuries**	**X**	**X**
**Transport injuries**	**X**	**X**
Road injuries	X	X
Pedestrian road injuries	X	X
Cyclist road injuries	X	X
Motorcyclist road injuries	X	X
Motor vehicle road injuries	X	X
Other road injuries	X	X
Other transport injuries	X	X
**Unintentional injuries**	**X**	**X**
Falls	X	X
Drowning	X	X
Fire, heat, and hot substances	X	X
Poisonings	X	X

Table 1: GBD 2016 cause list	Cause of death	Cause of disability
Exposure to mechanical forces	X	X
Unintentional firearm injuries	X	X
Unintentional suffocation	X	X
Other exposure to mechanical forces	X	X
Adverse effects of medical treatment	X	X
Animal contact	X	X
Venomous animal contact	X	X
Non-venomous animal contact	X	X
Foreign body	X	X
Pulmonary aspiration and foreign body in airway	X	X
Foreign body in eyes		X
Foreign body in other body part	X	X
Environmental heat and cold exposure	X	X
Other unintentional injuries	X	X
**Self-harm and interpersonal violence**	**X**	**X**
Self-harm	X	X
Self-harm by firearm	X	X
Self-harm by other specified means	X	X
Interpersonal violence	X	X
Physical violence by firearm	X	X
Physical violence by sharp object	X	X
Sexual violence		X
Physical violence by other means	X	X
**Forces of nature, conflict and terrorism, and state actor violence**	**X**	**X**
Exposure to forces of nature	X	X
Conflict and terrorism	X	X
State actor violence	X	X

Table 2: GBD 2016 risk factors list

Risk factor			
Environmental/occupational risks			
	Unsafe water, sanitation, and handwashing		
		Unsafe water source	
		Unsafe sanitation	
		No access to handwashing facility	
	Air pollution		
		Ambient particulate matter pollution	
		Household air pollution from solid fuels	
		Ambient ozone pollution	
	Other environmental risks		
		Residential radon	
		Lead exposure	
	Occupational risks		
		Occupational carcinogens	
			Occupational exposure to asbestos
			Occupational exposure to arsenic
			Occupational exposure to benzene
			Occupational exposure to beryllium
			Occupational exposure to cadmium
			Occupational exposure to chromium
			Occupational exposure to diesel engine exhaust
			Occupational exposure to secondhand smoke
			Occupational exposure to formaldehyde
			Occupational exposure to nickel
			Occupational exposure to polycyclic aromatic hydrocarbons
			Occupational exposure to silica
			Occupational exposure to sulfuric acid
			Occupational exposure to trichloroethylene
		Occupational asthmagens	
		Occupational particulate matter, gases, and fumes	
		Occupational noise	
		Occupational injuries	
		Occupational ergonomic factors	
Behavioral risks			
	Child and maternal malnutrition		
		Suboptimal breastfeeding	
			Non-exclusive breastfeeding
			Discontinued breastfeeding
		Child growth failure	
			Childhood underweight
			Childhood wasting
			Childhood stunting
		Low birth weight and short gestation	
			Short gestation for birth weight
			Low birth weight for gestation
		Iron deficiency	
		Vitamin A deficiency	
		Zinc deficiency	
	Tobacco use		
		Smoking	
		Smokeless tobacco	
		Secondhand smoke	

Legend
GBD level 1
GBD level 2
GBD level 3
GBD level 4

Table 2: GBD 2016 risk factors list
**Risk factor**
Alcohol and drug use
Alcohol use
Drug use
Dietary risks
Diet low in fruits
Diet low in vegetables
Diet low in legumes
Diet low in whole grains
Diet low in nuts and seeds
Diet low in milk
Diet high in red meat
Diet high in processed meat
Diet high in sugar-sweetened beverages
Diet low in fiber
Diet low in calcium
Diet low in seafood omega-3 fatty acids
Diet low in polyunsaturated fatty acids
Diet high in trans fatty acids
Diet high in sodium
Sexual abuse and violence
Childhood sexual abuse
Intimate partner violence
Unsafe sex
Low physical activity
**Metabolic risks**
High fasting plasma glucose
High total cholesterol
High systolic blood pressure
High body-mass index
Low bone mineral density
Impaired kidney function

Table 3: GBD 2016 India data inputs
Aarhus University, Addiction Switzerland Research Institute, Alcohol Research Group, Public Health Institute, Centre for Addiction and Mental Health, Centre for Alcohol Policy Research, Turning Point Alcohol and Drug Centre, Kettil Bruun Society for Social and Epidemiological Research on Alcohol, University of North Dakota. India - Karnataka Gender, Alcohol and Culture: An International Study (GENACIS) 2003.
Aarogyasri Health Trust, Government of Telangana. India - Telangana State Aarogyasri Dialysis 2014-2017 and Renal Transplant 2015-2017 Data. [Data shared for this analysis]
Aaron R, Joseph A, Abraham S, Muliyil J, George K, Prasad J, Minz S, Abraham VJ, Bose A. Suicides in young people in rural southern India. Lancet. 2004; 363(9415): 1117-8.
Abel R, Rajaratnam J, Gnanasekaran VJ, Jayaraman P. Prevalence of anaemia and iron deficiency in three trimesters in Rural Vellore district, South India. Trop Doct. 2001; 31(2): 86-9.
Abel R, Rajaratnam J, Kalaimani A, Kirubakaran S. Can iron status be improved in each of the three trimesters? A community-based study. Eur J Clin Nutr. 2000; 54(6): 490-3.
Abel R, Sampathkumar V. Tamil Nadu nutritional survey comparing children aged 0-3 years with the NCHS/CDC reference population. Indian J Pediatr. 1998; 65(4): 565-72.
Abraham A, Ramachandran R, Mohan D, Kutty R, Nair S. Pattern and determinants of respiratory mortality in Kerala, South India. Int J Med Public Health. 2014; 4(4): 467.
Acharya D, Prasanna K, Nair S, Rao R. Acute respiratory infections in children: a community based longitudinal study in south India. Indian J Public Health. 2003; 47(1): 7–13.
Acharya SK, Batra Y, Bhatkal B, Ojha B, Kaur K, Hazari S, Saraya A, Panda SK. Seroepidemiology of hepatitis A virus infection among school children in Delhi and north Indian patients with chronic liver disease: implications for HAV vaccination. J Gastroenterol Hepatol. 2003; 18(7): 822-7.
Ackerson LK, Kawachi I, Barbeau EM, Subramanian SV. Effects of individual and proximate educational context on intimate partner violence: a population-based study of women in India. Am J Public Health. 2008; 98(3): 507-14.
Action Aid International, Regional Medical Research Centre - Port Blair, Indian Council of Medical Research, National Institute of Mental Health and Neurosciences. Report on alcohol consumption prevalence and patterns in Andaman and Nicobar Islands 2007. Available from: http://nimhans.ac.in/cam/sites/default/files/Publications/10.pdf
Adamson PC, Krupp K, Freeman AH, Klausner JD, Reingold AL, Madhivanan P. Prevalence & correlates of primary infertility among young women in Mysore, India. Indian J Med Res. 2011; 440-6.
Adhikari P, Haldar JP. Prevalence of bancroftian filariasis in Burdwan district, West Bengal: II. Vector and microfilariae density in colliery and non-colliery areas. J Commun Dis. 1995; 27(3): 181-5.
Agarwal AK, Sen AK, Kalra NK, Gupta N. Prevalence of anaemia during pregnancy in district Burdwan, West Bengal. Indian J Public Health. 1999; 43(1): 26-31.
Agarwal AK, Yunus M, Ahmad J, Khan A. Rheumatic heart disease in India. J R Soc Promot Health. 1995; 115(5): 303-9.
Agarwal D, Pandey C, Agarwal K. Vitamin A administration and preschool child mortality. Nutr Res. 1995; 15(5): 669–80. as it appears in Imdad A, Herzer K, Mayo-Wilson E, Yakoob MY, Bhutta ZA. Vitamin A supplementation for preventing morbidity and mortality in children from 6 months to 5 years of age. Cochrane Database Syst Rev. 2010; CD008524.
Agarwal D. Natural history of measles in rural and urban community of Varanasi. J Commun Dis. 1986; 8: 289-98.
Agarwal DK, Agarwal KN, Tripathi AM. Nutritional status in rural pregnant women of Bihar and Uttar Pradesh. Indian Pediatr. 1987; 24(2): 119-25.
Agarwal DK, Bhardwaj B, Singla PN, Tripathi AM, Agarwal KN. Etiology of maternal and early childhood deficiency anemia. Indian J Pediatr. 1986; 53(3): 389-95.
Agarwal DK, Bhatia BD, Agarwal KN. Simple approach to acute respiratory infection in rural under five children. Indian Pediatr. 1993; 5(30): 629-35.

**Table 3: GBD 2016 India data inputs**
Agarwal DK, Upadhyay SK, Agarwal KN, Singh RD, Tripathi AM. Anaemia and mental functions in rural primary school children. Ann Trop Paediatr. 1989; 9(4): 194-8.
Agarwal KN, Agarwal DK, Mishra KP. Impact of anaemia prophylaxis in pregnancy on maternal haemoglobin, serum ferritin & birth weight. Indian J Med Res. 1991; 277-80.
Agarwal KN, Gomber S, Bisht H, Som M. Anemia prophylaxis in adolescent school girls by weekly or daily iron-folate supplementation. Indian Pediatr. 2003; 40(4): 296-301.
Agarwal N, Naik S, Aggarwal R, Singh H, Somani SK, Kini D, Pandey R, Choudhuri G, Saraswat VA, Naik SR. Occult hepatitis B virus infection as a cause of cirrhosis of liver in a region with intermediate endemicity. Indian J Gastroenterol. 2003; 22(4): 127-31.
Agarwal SK, Dash SC, Irshad M, Raju S, Singh R, Pandey RM. Prevalence of chronic renal failure in adults in Delhi, India. Nephrol Dial Transplant. 2005; 20(8): 1638-42.
Agarwal SK. Chronic renal failure in Delhi adults study dataset 2002-2003. [Data shared for this analysis]
Aggarwal AN, Gupta D, Agarwal R, Sethi S, Thakur JS, Anjinappa SM, Chadha VK, Kumar R, Sharma M, Behera D, Jindal SK. Prevalence of pulmonary tuberculosis among adults in a north Indian district. PLoS One. 2015; 10(2): e0117363.
Agrawal RP, Ola V, Bishnoi P, Gothwal S, Sirohi P, Agrawal R. Prevalence of micro and macrovascular complications and their risk factors in type-2 diabetes mellitus. J Assoc Physicians India. 2014; 62(6): 504–8.
Agrawal S. Effect of indoor air pollution from biomass and solid fuel combustion on prevalence of self-reported asthma among adult men and women in India: findings from a nationwide large-scale cross-sectional survey. J Asthma. 2012; 49(4): 355-65.
Agrawal D, Mohanty BB, Sarangi R, Kumar S, Mahapatra SK, Chinara PK. Study of incidence and prevalence of musculoskeletal anomalies in a tertiary care hospital of eastern India. J Clin Diagn Res. 2014; 8(5): AC04-6.
Agricultural Research Service, U.S. Department of Agriculture (USDA). USDA National Nutrient Database for Standard Reference, Release 27. Washington D.C., United States: USDA; 2014.
Ahamed M, Verma S, Kumar A, Siddiqui MKJ. Blood lead levels in children of Lucknow, India. Environ Toxicol. 2010; 25(1): 48-54.
Ahmad A, Khalique N, Khan Z, Amir A. Prevalence of psychosocial problems among school going male adolescents. Indian J Community Med. 2007; 32(3): 219-21.
Ahsan T, Shahid M, Mahmood T, Jabeen R, Jehangir U, Saleem M, Ahmed N, Shaheer A. Role of dexamethasone in acute bacterial meningitis in adults. J Pak Med Assoc. 2002; 52(6): 233-9.
Ajinkya S, Kaur D, Gursale A, Jadhav P. Prevalence of parent-rated attention deficit hyperactivity disorder and associated parent-related factors in primary school children of Navi Mumbai--a school based study. Indian J Pediatr. 2013; 80(3): 207–10.
Ajjampur SSR, Liakath FB, Kannan A, Rajendran P, Sarkar R, Moses PD, Simon A, Agarwal I, Mathew A, O'Connor R, Ward H, Kang G. Multisite study of cryptosporidiosis in children with diarrhea in India. J Clin Microbiol. 2010; 48(6): 2075-81.
Ali MK, Bhaskarapillai B, Shivashankar R, Mohan D, Fatmi ZA, Pradeepa R, Masood Kadir M, Mohan V, Tandon N, Narayan KM, Prabhakaran D, CARRS investigators. Socioeconomic status and cardiovascular risk in urban South Asia: The CARRS Study. Eur J Prev Cardiol. 2016; 23(4): 408-19.
All India Institute of Medical Sciences, Bangabandhu Sheikh Mujib Medical University, De Soysa Maternity Hospital, Gadjah Mada University, Institute of Medicine, Tribhuvan University, Queen Sirikt National Institute of Child Health, World Health Organization - Regional Office for South-East Asia (WHO SEARO). WHO South East Asia Regional Neonatal-Perinatal Database Report 2007-2008. New Delhi, India: WHO SEARO; 2008.
All India Institute of Medical Sciences, Bangur Institute of Neurology, Indian Statistical Institute, National Neurosciences Center. Kolkata Study for Epidemiology of Neurological Disorders 2003-2004.
All India Institute of Medical Sciences, Government Medical Colleges of Kerala, International Council for the Control of Iodine Deficiency Disorders, United Nations Children's Fund. Tracking Progress Towards Sustainable Elimination of Iodine Deficiency Disorders, Kerala 2001.

Table 3: GBD 2016 India data inputs
All India Institute of Medical Sciences. India Multi-centric Collaborative Study on the Impact of Global Warming and Ultra Violet Radiation Exposure on Ocular Health in India 2013-2015.
Allam RR, Murhekar MV, Bhatnagar T, Uthappa CK, Chava N, Rewari BB, Venkatesh S, Mehendale S. Survival probability and predictors of mortality and retention in care among patients enrolled for first-line antiretroviral therapy, Andhra Pradesh, India, 2008-2011. Trans R Soc Trop Med Hyg. 2014; 108(4): 198–205.
Alvarez JL, Dent N, Browne L, Myatt M, Briend A. Putting Child Kwarshiorkor on the Map. Community-Based Management of Acute Malnutrition (CMAM) Forum Technical Brief, March 2016.
Alvarez-Uria G, Naik PK, Pakam R, Midde M. Factors associated with attrition, mortality, and loss to follow up after antiretroviral therapy initiation: data from an HIV cohort study in India. Glob Health Action. 2013; 21682.
Amarapurkar DN, Dharod M, Gautam S, Patel N. Risk of development of hepatocellular carcinoma in patients with NASH-related cirrhosis. Trop Gastroenterol. 2013; 34(3): 159-63.
Amarapurkar DN, Patel ND, Kamani PM. Impact of diabetes mellitus on outcome of HCC. Ann Hepatol. 2008; 7(2): 148-51.
Ambade VN, Godbole HV. Comparison of wound patterns in homicide by sharp and blunt force. Forensic Sci Int. 2006; 156(2-3): 166-70.
Ambekar A, Tripathi BM, National Drug Dependence Treatment Centre, All India Institute of Medical Sciences, New Delhi (AIIMS), Joint United Nations Programme on HIV/AIDS (UNAIDS), Society for the Promotion of Youth and Masses. Report on Size Estimation of Injecting Drug Use in Punjab and Haryana. New Delhi, India: UNAIDS; 2008.
Amin V, Patwari AK, Kumar G, Anand VK, Diwan N, Peshin S. Clinical profile of cholera in young children--a hospital based report. Indian Pediatr. 1995; 32(7): 755–61.
Anand K, Kant S, Kumar G, Kapoor SK. "Development" is not essential to reduce infant mortality rate in India: experience from the Ballabgarh project. J Epidemiol Community Health. 2000; 54(4): 247-53.
Anand K, Patro BK, Paul E, Kapoor SK. Management of Sick Children by Health Workers in Ballabgarh: Lessons for Implementation of IMCI in India. J Trop Pediatr. 2004; 50(1): 41-7.
Anand S, Shivashankar R, Ali MK, Kondal D, Binukumar B, Montez-Rath ME, Ajay VS, Pradeepa R, Deepa M, Gupta R, Mohan V, Narayan KM, Tandon N, Chertow GM, Prabhakaran D. Prevalence of chronic kidney disease in two major Indian cities and projections for associated cardiovascular disease. Kidney Int. 2015; 88(1): 178-85.
Anima H, Malay M, Santanu H, Rajashree R, Sita C, Baran SA. A study on determinants of occurrence of complications and fatality among diphtheria cases admitted to ID and BG Hospital of Kolkata. J Commun Dis. 2008; 40(1): 53-8.
Anita, Gaur DR, Vohra AK, Subhash S, Khurana H. Prevalence Of Psychiatric Morbidity Among 6 To 14 Years Old Children. Indian J Community Med. 2003; 28: 133-7.
Anjana RM, Deepa M, Pradeepa R, Mahanta J, Narain K, Das HK, et al. Prevalence of diabetes and prediabetes in 15 states of India: results from the ICMR–INDIAB population-based cross-sectional study. Lancet Diabetes Endocrinol. 2017; 5(8):585–96.
Anjana RM, Pradeepa R, Das AK, Deepa M, Bhansali A, Joshi SR, et al. Physical activity and inactivity patterns in India - results from the ICMR-INDIAB study (Phase-1) (ICMR-INDIAB-5). Int J Behav Nutr Phys Act. 2014 Feb 26; 11(1):26.
Anjana RM, Pradeepa R, Deepa M, Datta M, Sudha V, Unnikrishnan R, Bhansali A, Joshi SR, Joshi PP, Yajnik CS, Dhandhania VK, Nath LM, Das AK, Rao PV, Madhu SV, Shukla DK, Kaur T, Priya M, Nirmal E, Parvathi SJ, Subhashini S, Subashini R, Ali MK, Mohan V. Prevalence of diabetes and prediabetes (impaired fasting glucose and/or impaired glucose tolerance) in urban and rural India: phase I results of the Indian Council of Medical Research-INdia DIABetes (ICMR-INDIAB) study. Diabetologia. 2011; 54(12): 3022-7.
Ansari M, Sharma Y, Roy A, Biswas S, Sharma P. Epidemiologic investigations of a malaria outbreak in northern Delhi area. J Am Mosq Control Assoc. 2001; 17(4): 216-20.
Ansel Vishal L, Nazeer Y, Ravishankaran R, Mahalakshmi N, Kaliraj P. Evaluation of rapid blood sample collection in the detection of circulating filarial antigens for epidemiological survey by rWbSXP-1 capture assay. PLoS One. 2014; 9(7): e102260.

Table 3: GBD 2016 India data inputs
Antonisamy B, Raghupathy P, Christopher S, Richard J, Rao PSS, Barker DJP, et al. Cohort Profile: The 1969-73 Vellore birth cohort study in South India. International Journal of Epidemiology. 2009; 38(3): 663–9.
Anuradha K, Singh HM, Gopal KVT, Rama Rao GR, Ramani TV, Padmaja J. Herpes simplex virus 2 infection: a risk factor for HIV infection in heterosexuals. Indian J Dermatol Venereol Leprol. 2008; 74(3): 230–3.
Anvikar AR, Rao VG, Savargaonkar DD, Rajiv Y, Bhondeley MK, Tiwari B, Karkare A, Luke C, Gadge V, Ukey M, Patel P. Seroprevalence of sexually transmitted viruses in the tribal population of Central India. Int J Infect Dis. 2009; 13(1): 37–9.
Aon Benfield, Impact Forecasting. Global Catastrophe: Recap September 2016. London, United Kingdom: Aon Benfield; 2016.
Arankalle VA, Chadha MS, Chitambar SD, Walimbe AM, Chobe LP, Gandhe SS. Changing epidemiology of hepatitis A and hepatitis E in urban and rural India (1982-98). J Viral Hepat. 2001; 8(4): 293-303.
Arankalle VA, Tsarev SA, Chadha MS, Alling DW, Emerson SU, Banerjee K, Purcell RH. Age-specific prevalence of antibodies to hepatitis A and E viruses in Pune, India, 1982 and 1992. J Infect Dis. 1995; 171(2): 447-50.
Archana BR, Prasad SR, Beena PM, Okade R, Sheela SR, Beeregowda YC. Maternal and congenital syphilis in Karnataka, India. Southeast Asian J Trop Med Public Health. 2014; 45(2): 430-4.
Arjunan A. Prevalence of the ßeta-s Gene and Sickle Cell Disease in India [Master's Thesis]. Pittsburgh, Pennsylvania: University of Pittsburgh; 2013.
Arunachalam N, Mariappan T, Vijayakumar KN, Sabesan S, Panicker KN. Mattancherry urban agglomeration, a diminishing focus of lymphatic filariasis in Kerala. J Commun Dis. 1996; 28(3): 168-70.
Arya S, Chellani H, Pandey J. Evaluation of safety of oral vitamin "A" megadose co-administered with measles vaccination. Indian Pediatr. 2000; 37(12): 1341–7. as it appears in Imdad A, Herzer K, Mayo-Wilson E, Yakoob MY, Bhutta ZA. Vitamin A supplementation for preventing morbidity and mortality in children from 6 months to 5 years of age. Cochrane Database Syst Rev. 2010; CD008524.
Ashok GV, Nagaiah N, Shiva Prasad NG. Indoor radon concentration and its possible dependence on ventilation rate and flooring type. Radiat Prot Dosimetry. 2012; 148(1): 92-100.
Ashok S, Ramu M, Deepa R, Mohan V. Prevalence of neuropathy in type 2 diabetic patients attending a diabetes centre in South India. J Assoc Physicians India. 2002; 546–50.
Ashraf M, Chowdhary J, Khajuria K, Reyaz AM. Spectrum of congenital heart diseases in Kashmir, India. Indian Pediatr. 2009; 46(12): 1107-8.
Asim M, Sarma MP, Kar P. Etiological and molecular profile of hepatocellular cancer from India. Int J Cancer. 2013; 133(2): 437-45.
Asthma Bhawan Jaipur, International Study of Asthma and Allergies in Childhood (ISAAC) Steering Committee, ISAAC Phase Three Study Group, ISAAC International Data Centre Auckland. India-ISAAC Phase-3 data 2001-2003 [Data shared for this analysis].
Atilola O, Stevanovic D, Balhara YPS, Avicenna M, Kandemir H, Knez R, Petrov P, Franic T, Vostanis P. Role of personal and family factors in alcohol and substance use among adolescents: an international study with focus on developing countries. J Psychiatr Ment Health Nurs. 2014; 21(7): 609–17.
Augustine AM, Jana AK, Kuruvilla KA, Danda S, Lepcha A, Ebenezer J, Paul RR, Tyagi A, Balraj A. Neonatal hearing screening--experience from a tertiary care hospital in southern India. Indian Pediatr. 2014; 51(3): 179–83.
Awasthi S, Awasthi R, Pande VK, Srivastav RC, Frumkin H. Blood lead in pregnant women in the urban slums of Lucknow, India. Occup Environ Med. 1996; 53(12): 836-40.
Awasthi S, Das R, Verma T, Vir S. Anemia and undernutrition among preschool children in Uttar Pradesh, India. Indian Pediatr. 2003; 40(10): 985-90.
Awasthi S, Pande VK. Cause-specific mortality in under fives in the urban slums of Lucknow, north India. J Trop Pediatr. 1998; 44(6): 358-61.
Awasthi S, Peto R, Read S, Clark S, Pande V, Bundy D, DEVTA (Deworming and Enhanced Vitamin A) team. Vitamin A supplementation every 6 months with retinol in 1 million pre-school children in north India: DEVTA, a cluster-randomised trial. Lancet. 2013; 381(9876): 1469-77.

Table 3: GBD 2016 India data inputs
Babji S, Arumugam R, Peters A, Ramani S, Kang G. Detection and characterisation of rotaviruses from children less than 5 years hospitalised with acute gastroenteritis in Nagercoil. Indian J Med Microbiol. 2013; 31(1): 69-71.
Babji S, Arumugam R, Sarvanabhavan A, Moses PD, Simon A, Aggarwal I, et al. Multi-center surveillance of rotavirus diarrhea in hospitalized children <5 years of age in India, 2009-2012. Vaccine. 2014 ;32 Suppl 1:A10-2.
Babu BV, Acharya AS, Mallick G, Jangid PK, Nayak AN, Satyanarayana K. Lymphatic filariasis in Khurda district of Orissa, India: an epidemiological study. Southeast Asian J Trop Med Public Health. 2001; 32(2): 240-3.
Babu CS, Satishchandra P, Sinha S, Subbakrishna DK. Co-morbidities in people living with epilepsy: hospital based case-control study from a resource-poor setting. Epilepsy Res. 2009; 86(2-3): 146-52.
Bahadur RA, Bhat BV. Congenital musculoskeletal malformations in neonates. J Indian Med Assoc. 1989; 87(2): 27-9.
Bahl R, Ray P, Subodh S, Shambharkar P, Saxena M, Parashar U, Gentsch J, Glass R, Bhan MK, Delhi Rotavirus Study Group. Incidence of severe rotavirus diarrhea in New Delhi, India, and G and P types of the infecting rotavirus strains. J Infect Dis. 2005; S114-119.
Baily GVJ, Narain R, Mayurnath S, Vallishayee SRS, Guld J. Trial of BCG vaccines in south India for tuberculosis prevention: first report--Tuberculosis Prevention Trial. Bull World Health Organ. 1979; 57(5):819-27.
Bains K, Mann SK. Physical Fitness in Relation to Energy and Iron Status of Female College Students. Food Nutr Bull. 2000; 21(3):305–10.
Baird GS, Fitzgerald RL, Aggarwal SK, Herold DA. Blood Lead Analysis by Negative Chemical Ionization GC-MS. Proceedings of the 42nd ASMS Conference on Mass Spectrometry and Allied Topics. 1994: 1137.
Bal MS, Beuria MK, Mandal NN, Das MK. Antigenemia in young children living in Wuchereria bancrofti-endemic areas of Orissa, India. Trans R Soc Trop Med Hyg. 2009; 103(3): 262-5.
Balagopal P, Kamalamma N, Patel TG, Misra R. A community-based participatory diabetes prevention and management intervention in rural India using community health workers. Diabetes Educ. 2012; 38(6): 822-34.
Balakrishnan K, Sambandam S, Ramaswamy P, Mehta S, Smith KR. Exposure assessment for respirable particulates associated with household fuel use in rural districts of Andhra Pradesh, India. J Expo Anal Environ Epidemiol. 2004; 14(Suppl 1): S14-25.
Balakrishnan K, Sankar S, Parikh J, Padmavathi R, Srividya K, Venugopal V, Prasad S, Pandey VL. Daily average exposures to respirable particulate matter from combustion of biomass fuels in rural households of southern India. Environ Health Perspect. 2002; 110(11): 1069-75.
Balakrishnan N, Ramaiah KD, Pani SP. Efficacy of bi-annual administration of DEC in the control of bancroftian filariasis. J Commun Dis. 1992; 24(2): 87-91.
Balgir RS. Do tribal communities show an inverse relationship between sickle cell disorders and glucose-6-phosphate dehydrogenase deficiency in malaria endemic areas of Central-Eastern India? Homo. 2006; 57(2): 163-76.
Bamji MS, V S Murthy PV, Williams L, Vardhana Rao MV. Maternal nutritional status & practices & perinatal, neonatal mortality in rural Andhra Pradesh, India. Indian J Med Res. 2008; 127(1): 44-51.
Bandyopadhyay R, Sengupta A, Dasgupta A, Biswas R, Mukherjee S, Biswas AB. A comparative study of common ear morbidity pattern among the primary school children of an urban slum of Kolkata and rural area of Hooghly. J Indian Med Assoc. 2005; 103(8): 428, 430-2.
Banerjee A, Chakravarty R, Mondal PN, Chakraborty MS. Hepatitis B virus genotype D infection among antenatal patients attending a maternity hospital in Calcutta, India: assessment of infectivity status. Southeast Asian J Trop Med Public Health. 2005; 36(1): 203-6.
Banerjee I, Gladstone BP, Le Fevre AM, Ramani S, Iturriza-Gomara M, Gray JJ, Brown DW, Estes MK, Muliyil JP, Jaffar S, Kang G. Neonatal infection with G10P11 rotavirus did not confer protection against subsequent rotavirus infection in a community cohort in Vellore, South India. J Infect Dis. 2007; 195(5): 625-32.
Banerjee TK, Dutta S, Das S, Ghosal M, Ray BK, Biswas A, Hazra A, Chaudhuri A, Paul N, Das SK. Epidemiology of dementia and its burden in the city of Kolkata, India. Int J Geriatr Psychiatry. 2017; 32: 605-614.

Table 3: GBD 2016 India data inputs
Banerjee TK, Dutta S, Ray BK, Ghosal M, Hazra A, Chaudhuri A, Das SK. Epidemiology of epilepsy and its burden in Kolkata, India. Acta Neurol Scand. 2015; 132(3): 203-11.
Banerjee TK, Hazra A, Biswas A, Ray J, Roy T, Raut DK, Chaudhuri A, Das SK. Neurological disorders in children and adolescents. Indian J Pediatr. 2009; 76(2): 139-46.
Banerjee TK, Mukherjee CS, Dutt A, Shekhar A, Hazra A. Cognitive dysfunction in an urban Indian population – some observations. Neuroepidemiology. 2008; 31(2): 109-14.
Banerjee TK, Mukherjee CS, Sarkhel A. Stroke in the urban population of Calcutta – an epidemiological study. Neuroepidemiology. 2001; 20(3): 201-7.
Banerjee TK, Ray BK, Das SK, Hazra A, Ghosal MK, Chaudhuri A, Roy T, Raut DK. A longitudinal study of epilepsy in Kolkata, India. Epilepsia. 2010; 51(12): 2384-91.
Bang AT, Bang RA, Baitule SB, Reddy MH, Deshmukh MD. Effect of home-based neonatal care and management of sepsis on neonatal mortality: field trial in rural India. Lancet. 1999; 354(9194): 1955-61.
Bang AT, Bang RA, Reddy HM, Deshmukh MD, Baitule SB. Reduced incidence of neonatal morbidities: effect of home-based neonatal care in rural Gadchiroli, India. J Perinatol. 2005; 25 (Suppl 1): S51-61.
Bang AT, Bang RA, Tale O, Sontakke P, Solanki J, Wargantiwar R, Kelzarkar P. Reduction in pneumonia mortality and total childhood mortality by means of community-based intervention trial in Gadchiroli, India. Lancet. 1990; 336(8709): 201-6.
Bansal D, Gudala K, Muthyala H, Esam HP, Nayakallu R, Bhansali A. Prevalence and risk factors of development of peripheral diabetic neuropathy in type 2 diabetes mellitus in a tertiary care setting. J Diabetes Investig. 2014; 5(6): 714–21.
Bansal PD, Barman R. Psychopathology of school going children in the age group of 10-15 years. Int J Appl Basic Med Res. 2011; 1(1): 43-7.
Bapat U, Alcock G, More NS, Das S, Joshi W, Osrin D. Stillbirths and newborn deaths in slum settlements in Mumbai, India: a prospective verbal autopsy study. BMC Pregnancy Childbirth. 2012; 12: 39.
Baqui AH, Darmstadt GL, Williams EK, Kumar V, Kiran TU, Panwar D, Srivastava VK, Ahuja R, Black RE, Santosham M. Rates, timing and causes of neonatal deaths in rural India: implications for neonatal health programmes. Bull World Health Organ. 2006; 84(9): 706-13.
Barnett S, Nair N, Tripathy P, Borghi J, Rath S, Costello A. A prospective key informant surveillance system to measure maternal mortality -- findings from indigenous populations in Jharkhand and Orissa, India. BMC Pregnancy Childbirth. 2008; 8(6): 6.
Bartra C, Mittal P, Adak T, Sharma V. Malaria investigation in District Jodhpur, Rajasthan, during the summer season. Indian J Malariol. 1998; 36(3-4): 75-80.
Baruah J, Kusre G, Bora R. Pattern of Gross Congenital Malformations in a Tertiary Referral Hospital in Northeast India. Indian J Pediatr. 2015; 82(10): 917–22.
Basa S, Ranjan Das R, Khan JA. Root-cause analytical survey for measles outbreak: vaccination or vaccine?- a study from Madhepura district, Bihar, India. J Clin Diagn Res. 2015; 9(6): SC04-SC07.
Basavaraj P, Sunil MK, Nagarajappa R, Ashish S, Ramesh G. Correlation Between Oral Health and Child-OIDP Index in 12- and 15-Year-Old Children From Modinagar, India. Asia Pac J Public Health. 2013; 26(4): 390-400.
Batra Y, Bhatkal B, Ojha B, Kaur K, Saraya A, Panda SK, Acharya SK. Vaccination against hepatitis A virus may not be required for schoolchildren in northern India: results of a seroepidemiological survey. Bull World Health Organ. 2002; 80(9): 728-31.
Baul MK, Manjusha. Maternal mortality--a ten-year study. J Indian Med Assoc. 2004; 102(1): 18-9, 25.
Baxi AJ, Balakrishnan V, Sanghvi LD. Deficiency of glucose-6-phosphate dehydrogenase observations on a sample from Bombay. Curr Sci. 1961; 30(1): 16-7.
Becker ML, Ramesh BM, Washington RG, Halli S, Blanchard JF, Moses S. Prevalence and determinants of HIV infection in South India: a heterogeneous, rural epidemic. AIDS. 2007; 21(6): 739–47.

Table 3: GBD 2016 India data inputs
Beegom R, Singh RB. Association of higher saturated fat intake with higher risk of hypertension in an urban population of Trivandrum in South India. Int J Cardiol. 1997; 58(1): 63-70.
Beegom R. Diet, central obesity and prevalence of hypertension in the urban population of South India. Int J Cardiol. 1995; 51(2): 183-91.
Behari JR, Singh S, Tandon SK, Wahal AK. Lead poisoning among Indian silver jewelry makers. Ann Occup Hyg. 1983; 27(1): 107-9.
Behera P, Sharan P, Mishra AK, Nongkynrih B, Kant S, Gupta SK. Prevalence and determinants of depression among elderly persons in a rural community from northern India. Natl Med J India. 2016; 29(3): 129–35.
Behl RK, Kashyap S, Sarkar M. Prevalence of bronchial asthma in school children of 6-13 years of age in Shimla city. Indian J Chest Dis Allied Sci. 2010; 52(3): 145-8.
Bellinger DC, Hu H, Kalaniti K, Thomas N, Rajan P, Sambandam S, Ramaswamy P, Balakrishnan K. A pilot study of blood lead levels and neurobehavioral function in children living in Chennai, India. Int J Occup Environ Health. 2005; 11(2): 138-43.
Bellizzi S, Ali MM, Abalos E, Betran AP, Kapila J, Pileggi-Castro C, Vogel JP, Merialdi M. Are hypertensive disorders in pregnancy associated with congenital malformations in offspring? Evidence from the WHO Multicountry cross sectional survey on maternal and newborn health. BMC Pregnancy Childbirth. 2016; 16(1): 198.
Benara SK, Singh P. Validity of causes of infant death by verbal autopsy. Indian Pediatr. 1999; 66(5): 647-50.
Bera DK, Banerjee P. A lymphatic filarial survey revealing a focus of Brugia malayi in the coastal region of west Bengal. J Indian Med Assoc. 1996; 94(8): 296-7, 310.
Bera P, Das S, Saha R, Ramachandran VG, Shah D. Cryptosporidium in children with diarrhea: a hospital-based Study. Indian Pediatr. 2014; 51(11): 906–8.
Berg CJ, Ajay VS, Ali MK, Kondal D, Khan HM, Shivashankar R, et al. A cross-sectional study of the prevalence and correlates of tobacco Use in Chennai, Delhi, and Karachi: data from the CARRS study. BMC Public Health. 2015; 15: 483.
Berry N, Chakravarti A, Kar P, Das BC, Santhanam, Mathur MD. Association of Hepatitis C virus and Hepatitis B virus in chronic liver disease. Indian J Med Res. 1998; 108(6): 255-9.
Bettadapura GS, Donthi K, Datti NP, Ranganath BG, Ramaswamy SB, Jayaram TS. Assessment of avoidable blindness using the rapid assessment of avoidable blindness methodology. N Am J Med Sci. 2012; 4(9): 389-93.
Beuria MK, Bal MS, Mandal NN, Das MK. Age-dependent prevalence of asymptomatic amicrofilaraemic individuals in a Wuchereria bancrofti-endemic region of India. Trans R Soc Trop Med Hyg. 2003; 97(3): 297-8.
Bhagyalaxmi A, Atul T, Shikha J. Prevalence of risk factors of non-communicable diseases in a District of Gujarat, India. J Health Popul Nutr. 2013; 31(1): 78-85.
Bhalla P, Garg S, Kakkar M, Sharma VK. Community-based study of hepatitis B markers in women of reproductive age. Indian J Gastroenterol. 2003; 22(1): 33-4.
Bhan MK, Bhandari N, Sazawal S, Clemens J, Raj P, Levine MM, Kaper JB. Descriptive epidemiology of persistent diarrhoea among young children in rural northern India. Bull World Health Organ. 1989; 67(3): 281-8.
Bhandari N, Bahl R, Taneja S, Martines J, Bhan MK. Pathways to infant mortality in urban slums of Delhi, India: implications for improving the quality of community- and hospital-based programmes. J Health Popul Nutr. 2002; 20(2): 148-55.
Bhandari N, Mazumder S, Taneja S, Sommerfelt H, Strand TA. Effect of implementation of Integrated Management of Neonatal and Childhood Illness (IMNCI) programme on neonatal and infant mortality: cluster randomised controlled trial. BMJ. 2012; 344: e1634.
Bhandari N, Rongsen-Chandola T, Bavdekar A, John J, Antony K, Taneja S, Goyal N, Kawade A, Kang G, Rathore SS, Juvekar S, Muliyil J, Arya A, Shaikh H, Abraham V, Vrati S, Proschan M, Kohberger R, Thiry G, Glass R, Greenberg HB, Curlin G, Mohan K, Harshavardhan GV, Prasad S, Rao TS, Boslego J, Bhan MK. Efficacy of a monovalent human-bovine (116E) rotavirus vaccine in Indian infants: a randomised, double-blind, placebo-controlled trial. Lancet. 2014; 383(9935): 2136-43.

Table 3: GBD 2016 India data inputs
Bhansali A, Dhandania VK, Deepa M, Anjana RM, Joshi SR, Joshi PP, Madhu SV, Rao PV, Subashini R, Sudha V, Unnikrishnan R, Das AK, Shukla DK, Kaur T, Mohan V, Pradeepa R. Prevalence of and risk factors for hypertension in urban and rural India: the ICMR-INDIAB study. J Hum Hypertens. 2015; 29(3): 204–9.
Bharaj P, Sullender WM, Kabra SK, Mani K, Cherian J, Tyagi V, Chahar HS, Kaushik S, Dar L, Broor S. Respiratory viral infections detected by multiplex PCR among pediatric patients with lower respiratory tract infections seen at an urban hospital in Delhi from 2005 to 2007. Virol J. 2009; 6: 89.
Bharati DR, Pal R, Kar S, Rekha R, Yamuna TV, Basu M. Prevalence and determinants of diabetes mellitus in Puducherry, South India. J Pharm Bioallied Sci. 2011; 3(4): 513-8.
Bharati DR, Pal R, Rekha R, Yamuna TV. Evaluation of the burden of type 2 diabetes mellitus in population of Puducherry, South India. Diabetes Metab Syndr. 2011; 5(1): 12-6.
Bharati P, Ghosh R, Gupta R. Socioeconomic Condition and Anaemia among the Mahishya Population of Southern West Bengal, India. Malays J Nutr. 2004; 10(1): 23-30.
Bharati S, Pal M, Bhattacharya BN, Bharati P. Prevalence and causes of chronic energy deficiency and obesity in Indian women. Hum Biol. 2007; 79(4): 395-412.
Bhardwaj AK, Nayar D, Ramachandran S, Kapil U. Assessment of iodine deficiency in district Bikaner, Rajasthan. Indian J Matern Child Health. 1997; 8(1): 18-20.
Bhardwaj R, Kandoria A, Marwah R, Vaidya P, Singh B, Dhiman P, Sharma A. Prevalence of rheumatic fever and rheumatic heart disease in rural population of Himachal--a population based study. J Assoc Physicians India. 2012; 60:13–4.
Bhardwaj R, Kandoria A, Marwah R, Vaidya P, Singh B, Dhiman P, Sood A, Sharma A. Prevalence of congenital heart disease in rural population of Himachal - A population-based study. Indian Heart J. 2016; 68(1): 48-51.
Bhargava M, Iyer PU, Kumar R, Ramji S, Kapani V, Bhargava SK. Relationship of maternal serum ferritin with foetal serum ferritin, birth weight and gestation. J Trop Pediatr. 1991; 37(4): 149-52.
Bhargava SK, Singh KK, Saxena BN, India Council of Medical Research (ICMR). ICMR Task Force National Collaborative Study on Identification of High Risk Families, Mothers and Outcome of their Off-springs with particular reference to the problem of maternal nutrition, low birth weight, perinatal and infant morbidity and mortality in rural and urban slum communities. Summary, conclusions and recommendations. 1991; 28(12): 1473-80.
Bharucha NE, Bharucha EP, Bharucha AE, Bhise AV, Schoenberg BS. Prevalence of epilepsy in the Parsi community of Bombay. Epilepsia. 1988; 29(2): 111-5.
Bharucha NE, Bharucha EP, Bharucha AE, Bhise AV, Schoenberg BS. Prevalence of Parkinson's disease in the Parsi community of Bombay, India. Arch Neurol. 1988; 45(12): 1321-3.
Bharucha NE, Bharucha EP, Dastur HD, Schoenberg BS. Pilot survey of the prevalence of neurologic disorders in the Parsi community of Bombay. Am J Prev Med. 1987; 3(5): 293-9.
Bharucha NE, Bharucha EP, Wadia NH, Singhal BS, Bharucha AE, Bhise AV, Kurtzke JF, Schoenberg BS. Prevalence of multiple sclerosis in the Parsis of Bombay. Neurology. 1988; 38(5): 727-9.
Bhat AW, Churoo BA, Iqbal Q, Sheikh MA, Iqbal J, Aziz R. Complication of exchange transfusion at a tertiary care hospital. Curr Pediatr Res. 2011; 15(2): 97-9.
Bhat BV, Babu L. Congenital malformations at birth--a prospective study from south India. Indian J Pediatr. 1998; 65(6): 873-81.
Bhat J, Rao VG, Gopi PG, Yadav R, Selvakumar N, Tiwari B, Gadge V, Bhondeley MK, Wares F. Prevalence of pulmonary tuberculosis amongst the tribal population of Madhya Pradesh, central India. Int J Epidemiol. 2009; 38(4): 1026-32.

Table 3: GBD 2016 India data inputs
Bhatia JC, Cleland J, Bhagavan L, Rao N. Levels and determinants of gynecological morbidity in a district of south India. Stud Fam Plann. 1997; 28(2): 95-103.
Bhatia JC. Levels and causes of maternal mortality in southern India. Stud Fam Plann. 1993; 24(5): 310-8.
Bhatia R. Measles outbreak in village Tophema in Nagaland. J Commun Dis. 1985; 17(2): 185-9.
Bhatt RM, Sharma SN, Uragayala S, Dash AP, Kamaraju R. Effectiveness and durability of Interceptor long-lasting insecticidal nets in a malaria endemic area of central India. Malar J. 2012; 11: 189.
Bhattacharya D, Sugunan AP, Bhattacharjee H, Thamizhmani R, Sayi DS, Thanasekaran K, Manimunda SP, Ghosh AR, Bharadwaj AP, Singhania M, Roy S. Antimicrobial resistance in Shigella--rapid increase & widening of spectrum in Andaman Islands, India. Indian J Med Res. 2012; 135: 365-70.
Bhattacharya P, Chandra P-K, Datta S, Banerjee A, Chakraborty S, Rajendran K, Basu S-K, Bhattacharya S-K, Chakravarty R. Significant increase in HBV, HCV, HIV and syphilis infections among blood donors in West Bengal, Eastern India 2004-2005: exploratory screening reveals high frequency of occult HBV infection. World J Gastroenterol. 2007; 13(27): 3730-3.
Bhattacharya SS, Das U, Choudhury BK. Occurrence and antibiogram of Salmonella Typhi and S. Paratyphi A isolated from Rourkela, Orissa. Indian J Med Res. 2011; 133(4): 431-3.
Bhaya M, Panwar S, Beniwal R, Panwar RB. High prevalence of rheumatic heart disease detected by echocardiography in school children. Echocardiography. 2010; 27(4): 448-53.
Bhowate RR, Borle SR, Chinchkhede DH, Gondhalekar RV. Dental health amongst 11-15-year-old children in Sevagram, Maharashtra. Indian J Dent Res. 1994; 5(2): 65-8.
Bhutani VK, Zipursky A, Blencowe H, Khanna R, Sgro M, Ebbesen F, Bell J, Mori R, Slusher TM, Fahmy N, Paul VK, Du L, Okolo AA, de Almeida MF, Olusanya BO, Kumar P, Cousens S, Lawn JE. Neonatal hyperbilirubinemia and Rhesus disease of the newborn: incidence and impairment estimates for 2010 at regional and global levels. Pediatr Res. 2013; 74(Suppl 1): 86-100.
Biran A, Schmidt WP, Wright R, Jones T, Seshadri M, Isaac P, Nathan NA, Hall P, McKenna J, Granger S, Bidinger P, Curtis V. The effect of a soap promotion and hygiene education campaign on handwashing behaviour in rural India: a cluster randomised trial. Trop Med Int Health. 2009; 14(10): 1303-14.
Birth Defects Registry of India, Fetal Care Research Foundation. Site-wise Birth Defects Registry Data 2008. Chennai, India: Fetal Care Research Foundation. [Data shared for this analysis]
Birth Defects Registry of India, Fetal Care Research Foundation. Site-wise Birth Defects Registry Data 2009. Chennai, India: Fetal Care Research Foundation. [Data shared for this analysis]
Birth Defects Registry of India, Fetal Care Research Foundation. Site-wise Birth Defects Registry Data 2010. Chennai, India: Fetal Care Research Foundation. [Data shared for this analysis]
Bisai S, Mahalanabis D, Sen A, Bose K, Datta N. Maternal early second trimester pregnancy weight in relation to birth outcome among Bengalee Hindus of Kolkata, India. Ann Hum Biol. 2007; 34(1): 91-101.
Bisoi S, Chakraborty S, Chattopadhyay D, Biswas B, Ray S. Glucose-6-phosphate dehydrogenase screening of babies born in a tertiary care hospital in West Bengal. Indian J Public Health. 2012; 56(2): 146–8.
Biswas AB, Chakraborty I, Das DK, Biswas S, Nandy S, Mitra J. Iodine deficiency disorders among school children of Malda, West Bengal, India. J Health Popul Nutr. 2002; 20(2): 180-3.
Biswas AB, Chakraborty I, Das DK, Chakraborty A, Ray D, Mitra K. Elimination of iodine deficiency disorders--current status in Purba Medinipur district of West Bengal, India. Indian J Public Health. 2008; 52(3): 130-5.

Table 3: GBD 2016 India data inputs
Biswas D, Borkakoty B, Mahanta J, Walia K, Saikia L, Akoijam BS, Jampa L, Kharkongar A, Zomawia E. Seroprevalence and risk factors of herpes simplex virus type-2 infection among pregnant women in Northeast India. BMC Infect Dis. 2011; 11: 325.
Biswas M, Manna CK. Prevalence of hypertension and sociodemographic factors within the Scheduled Caste community of the District Nadia, West Bengal, India. High Blood Press Cardiovasc Prev. 2011; 18(4): 179–85.
Biswas R, Biswas AB, Manna B, Bhattacharya SK, Dey R, Sarkar S. Effect of vitamin A supplementation on diarrhoea and acute respiratory tract infection in children. A double blind placebo controlled trial in a Calcutta slum community. Eur J Epidemiol. 1994; 10(1): 57–61. as it appears in Imdad A, Herzer K, Mayo-Wilson E, Yakoob MY, Bhutta ZA. Vitamin A supplementation for preventing morbidity and mortality in children from 6 months to 5 years of age. Cochrane Database Syst Rev. 2010; CD008524.
Biswas S, Seth RK, Tyagi PK, Sharma SK, Dash AP. Naturally Acquired Immunity and Reduced Susceptibility to falciparum Malaria in Two Subpopulations of Endemic Eastern India. Scand J Immunol. 2008; 67(2): 177-84.
Blencowe H, Cousens S, Jassir FB, Say L, Chou D, Mathers C, Hogan D, Shiekh S, Qureshi ZU, You D, Lawn JE; Lancet Stillbirth Epidemiology Investigator Group. National, regional, and worldwide estimates of stillbirth rates in 2015, with trends from 2000: a systematic analysis. Lancet Glob Health. 2016; 4(2): e98-108.
Bora D, Meena VR, Bhagat H, Dhariwal AC, Lal S. Soil transmitted helminthes prevalence in school children of Pauri Garhwal District, Uttaranchal state. J Commun Dis. 2006; 38(1): 112–4.
Bourne R, Global Burden of Disease Vision Loss Expert Group. Vision Loss Database - Survey Data on Vision Loss by Severity and Etiology. [Data shared for this analysis]
Bowman RM, McLone DG, Grant JA, Tomita T, Ito JA. Spina bifida outcome: a 25-year prospective. Pediatr Neurosurg. 2001; 34(3): 114-20.
BP. BP Statistical Review of World Energy 2016. London, United Kingdom: BP; 2016.
Brabin L, Nicholas S, Gogate A, Gogate S, Karande A. High prevalence of anaemia among women in Mumbai, India. Food Nutr Bull. 1998; 19(3): 205-9.
Brahmam G, Madhavan NK, Laxmaiah A, Reddy C, Ranganathan S, Vishnuvardhana Rao M, Nadamuni Naidu A, Vijayaraghavan K, Sivakumar B, Kamala Krishnaswamy, Gowarinth Sastry J, Mohan Ram M, Praldad Rao N, Vinodini R. Community Trials with Iron and Iodine Fortified Salt (Double Fortified Salt). In: Geertman RM, ed. Proceedings of the 8th World Salt Symposium. Amsterdam, The Netherlands: Elsevier; 2000. 955-60.
Brahmbhatt S, Brahmbhatt RM, Boyages SC. Thyroid ultrasound is the best prevalence indicator for assessment of iodine deficiency disorders: a study in rural/tribal schoolchildren from Gujarat (Western India). Eur J Endocrinol. 2000; 143(1): 37-46.
Brinkhof MWG, Dabis F, Myer L, Bangsberg DR, Boulle A, Nash D, Schechter M, Laurent C, Keiser O, May M, Sprinz E, Egger M, Anglaret X, ART-LINC, IeDEA. Early loss of HIV-infected patients on potent antiretroviral therapy programmes in lower-income countries. Bull World Health Organ. 2008; 86(7): 559-67.
British Geological Survey, Natural Environment Research Council. World Mineral Production 2007-2011. Nottingham, United Kingdom: British Geological Survey; 2013.
Brooks WA, Goswami D, Rahman M, Nahar K, Fry AM, Balish A, Iftekharuddin N, Azim T, Xu X, Klimov A, Bresee J, Bridges C, Luby S. Influenza is a major contributor to childhood pneumonia in a tropical developing country. Pediatr Infect Dis J. 2010; 29(3): 216–21.
Broor S, Parveen S, Bharaj P, Prasad VS, Srinivasulu KN, Sumanth KM, Kapoor SK, Fowler K, Sullender WM. A prospective three-year cohort study of the epidemiology and virology of acute respiratory infections of children in rural India. PLoS One. 2007; 6(2): e491.
Broughton E. The Bhopal disaster and its aftermath: a review. Environ Health. 2005; 4(1): 6.
Brown I, Elliott P. Sodium Intakes Around the World. Background document prepared for the Forum and Technical Meeting on Reducing Salt Intake in Populations (Paris 5-7th October 2006). Geneva, Switzerland: World Health Organization; 2007.

Table 3: GBD 2016 India data inputs
Buist AS, McBurnie MA, Vollmer WM, Gillespie S, Burney P, Mannino DM, Menezes AM, Sullivan SD, Lee TA, Weiss KB, Jensen RL, Marks GB, Gulsvik A,Nizankowska-Mogilnicka E, BOLD Collaborative Research Group. International variation in the prevalence of COPD (the BOLD Study): a population-based prevalence study. Lancet. 2007; 9589(9589): 741-50.
Cancer Institute (Women's India Association), International Agency for Research on Cancer. Dindigul Ambilikkai Cancer Registry 2012-2013. [Data shared for this analysis]
Cancer Institute (Women's India Association), Ministry of Health and Family Welfare, Government of Tamil Nadu. Tamil Nadu Cancer Registry Project (TNCRP) 2012-2013. [Data shared for this analysis]
Cardiological Society of India. India - Kerala Acute Coronary Syndrome Registry 2007-2009. [Data shared for this analysis]
Cardiological Society of India. India - Kerala Coronary Artery Disease Risk Factors Prevalence Study 2011. [Data shared for this analysis]
Carpio A, Bharucha N, Jallon P, Beghi E, Campostrini R, Zorzetto S, Mounkoro P. Mortality of epilepsy in developing countries. Epilepsia. 2005; 46(Suppl 11): 28-32.
Carter Center, International Trachoma Initiative, London School of Hygiene and Tropical Medicine. Global Atlas of Trachoma. Decatur, United States: International Trachoma Initiative.
Center for Addiction Medicine, National Institute of Mental Health and Neurosciences, Government of India, World Health Organization Collaborative Programme. Report on Patterns and Consequences of Alcohol Misuse in India: An Epidemiological Survey 2011-2012. Available from: http://nimhans.ac.in/cam/sites/default/files/Publications/WHO_ALCOHOL%20IMPACT_REPORT-FINAL21082012.pdf
Center for Vaccine Development, University of Maryland, Centers for Disease Control and Prevention, Department of Medical Microbiology and Immunology, Göteborg University, International Vaccine Institute, National Institute of Cholera and Enteric Diseases, Perry Point Cooperative Studies Program Coordinating Center, U.S. Department of Veterans Affairs, School of Medicine, University of Virginia, University of Chile. India - Kolkata Global Enteric Multicenter Study 2007-2011. Baltimore, MD, United States: Center for Vaccine Development, University of Maryland.
Center for Vaccine Development, University of Maryland, Centers for Disease Control and Prevention, Department of Medical Microbiology and Immunology, Göteborg University, International Vaccine Institute, National Institute of Cholera and Enteric Diseases, Perry Point Cooperative Studies Program Coordinating Center, U.S. Department of Veterans Affairs, School of Medicine, University of Virginia, University of Chile. India - Kolkata Global Enteric Multicenter Study 2011-2013. Baltimore, United States: Center for Vaccine Development, University of Maryland.
Central Board of Secondary Education, Department of School Education and Literacy, Ministry of Human Resource Development, Government of India, Centers for Disease Control and Prevention, World Health Organization (WHO). India Global School-Based Student Health Survey 2007. Geneva, Switzerland: WHO; 2007.
Central Bureau of Health Intelligence, Directorate General of Health Services, Ministry of Health and Family Welfare, Government of India, World Health Organization, Advent Healthcare Group. India National Health Profile 2005. New Delhi, India: Central Bureau of Health Intelligence; 2006.
Central Bureau of Health Intelligence, Directorate General of Health Services, Ministry of Health and Family Welfare, Government of India, World Health Organization. India National Health Profile 2006. New Delhi, India: Central Bureau of Health Intelligence; 2007.
Central Bureau of Health Intelligence, Directorate General of Health Services, Ministry of Health and Family Welfare, Government of India. India National Health Profile 2007. New Delhi, India: Central Bureau of Health Intelligence; 2008.
Central Bureau of Health Intelligence, Directorate General of Health Services, Ministry of Health and Family Welfare, Government of India. India National Health Profile 2008. New Delhi, India: Central Bureau of Health Intelligence; 2009.
Central Bureau of Health Intelligence, Directorate General of Health Services, Ministry of Health and Family Welfare, Government of India. India National Health Profile 2009. New Delhi, India: Central Bureau of Health Intelligence; 2010.

Table 3: GBD 2016 India data inputs
Central Bureau of Health Intelligence, Directorate General of Health Services, Ministry of Health and Family Welfare, Government of India. India National Health Profile 2010. New Delhi, India: Central Bureau of Health Intelligence; 2011.
Central Bureau of Health Intelligence, Directorate General of Health Services, Ministry of Health and Family Welfare, Government of India. India National Health Profile 2011. New Delhi, India: Central Bureau of Health Intelligence; 2012.
Central Bureau of Health Intelligence, Directorate General of Health Services, Ministry of Health and Family Welfare, Government of India. India National Health Profile 2013. New Delhi, India: Central Bureau of Health Intelligence; 2014.
Central Bureau of Health Intelligence, Directorate General of Health Services, Ministry of Health and Family Welfare, Government of India. India National Health Profile 2015. New Delhi, India: Central Bureau of Health Intelligence; 2015.
Central Pollution Control Board (CPCB), Ministry of Environment, Forest and Climate Change, Government of India. India - National Air Quality Monitoring Programme Data 2012. New Delhi, India: CPCB.
Central Pollution Control Board (CPCB), Ministry of Environment, Forest and Climate Change, Government of India. India - National Air Quality Monitoring Programme Data 2013. New Delhi, India: CPCB.
Central Pollution Control Board, Ministry of Environment, Forest and Climate Change, Government of India. National Water Quality Monitoring Network Data 1995-2011. Available from: http://cpcb.nic.in/water.php
Central Tuberculosis Division, Directorate General of Health Services, Ministry of Health and Family Welfare, Government of India. Mortality surveys in Andhra Pradesh and Odisha 2005-2006. [Data shared for this analysis]
Central Tuberculosis Division, Directorate General of Health Services, Ministry of Health and Family Welfare, Government of India. Tuberculosis Case Notifications by State 1999-2016.
Central Tuberculosis Division, Directorate General of Health Services, Ministry of Health and Family Welfare, Government of India. Tuberculosis Survey in Banda, Uttar Pradesh, 2008-2009. [Data shared for this analysis]
Central Tuberculosis Division, Directorate General of Health Services, Ministry of Health and Family Welfare, Government of India. Tuberculosis Survey in Chennai, Tamil Nadu, 2009-2010. [Data shared for this analysis]
Central Tuberculosis Division, Directorate General of Health Services, Ministry of Health and Family Welfare, Government of India. Tuberculosis Survey in Faridabad, Haryana, 2008-2009. [Data shared for this analysis]
Central Tuberculosis Division, Directorate General of Health Services, Ministry of Health and Family Welfare, Government of India. Tuberculosis Survey in Gujarat 2011-2012. [Data shared for this analysis]
Central Tuberculosis Division, Directorate General of Health Services, Ministry of Health and Family Welfare, Government of India. Tuberculosis Survey in Jabalpur, Madhya Pradesh, 2007-2009. [Data shared for this analysis]
Central Tuberculosis Division, Directorate General of Health Services, Ministry of Health and Family Welfare, Government of India. Tuberculosis Survey in Nelamangala, Karnataka, 2007-2009. [Data shared for this analysis]
Central Tuberculosis Division, Directorate General of Health Services, Ministry of Health and Family Welfare, Government of India. Tuberculosis Survey in Wardha, Maharashtra, 2007-2009. [Data shared for this analysis]
Centre for Chronic Disease Control, All India Institute of Medical Sciences, Madras Diabetes Research Foundation, Public Health Foundation of India, Emory University, Aga Khan University. Center for Cardio-Metabolic Risk Reduction in South Asia Surveillance Baseline Survey 2010-2011. [Data shared for this analysis]
Centre for Chronic Disease Control, All India Institute of Medical Sciences, Madras Diabetes Research Foundation, Public Health Foundation of India, Emory University, Aga Khan University. Center for Cardio-Metabolic Risk Reduction in South Asia Surveillance Follow-up Survey 2013. [Data shared for this analysis]
Centre for Chronic Disease Control, Indian Council of Medical Research. India Diet and Lifestyle Interventions for Hypertension RisR reduction through Anganwandi Workers and Accredited Social Health Activists Baseline Study Dataset 2013-2014. [Data shared for this analysis]

Table 3: GBD 2016 India data inputs
Centre for Chronic Disease Control. India Prevalence of Coronary Heart Disease and its Risk Factors in Residents of Urban and Rural Areas of NCR Survey Dataset 2010-2012. [Data shared for this analysis]
Centre for Chronic Disease Control. India Prevalence of Coronary Heart Disease and its Risk Factors in Residents of Urban and Rural Areas of NCR Survey Report 2010-2012. New Delhi, India: Centre for Chronic Disease Control.
Centre for Research on the Epidemiology of Disasters (CRED), Catholic University of Leuven. EM-DAT: The OFDA/CRED International Disaster Database. Brussels, Belgium: Catholic University of Leuven.
Chadha MS, Chitambar SD, Shaikh NJ, Arankalle VA. Exposure of Indian children to hepatitis A virus & vaccination age. Indian J Med Res. 1999; 109 (1): 11-5.
Chadha SK, Sayal A, Malhotra V, Agarwal AK. Prevalence of preventable ear disorders in over 15,000 schoolchildren in northern India. J Laryngol Otol. 2013; 127(1): 28-32.
Chadha SL, Gopinath N, Shekhawat S. Urban-rural differences in the prevalence of coronary heart disease and its risk factors in Delhi. Bull World Health Organ. 1997; 75(1): 31-8.
Chadha SL, Singh N, Shukla DK. Epidemiological study of congenital heart disease. Indian J Pediatr. 2001; 68(6): 507-10.
Chadha VK, Agarwal SP, Kumar P, Chauhan LS, Kollapan C, Jaganath PS, et al. Annual risk of tuberculous infection in four defined zones of India: a comparative picture. Int J Tuberc Lung Dis. 2005 ;9(5):569–575.
Chadha VK, Anjinappa SM, Gowda U, Srivastava R, Ahmed J, Kumar P. Annual risk of tuberculosis infection in a rural population of South India and its relationship with prevalence of smear positive pulmonary tuberculosis. Indian Journal of Tuberculosis. 2013 Oct; 60(4): 227-232.
Chadha VK, Jagannath PS, Suryanarayana H, others. Tuberculin sensitivity in BCG vaccinated children and its implications for ARI estimation. Indian J Tuberc. 2000; 47(3): 139–146.
Chadha VK, Jagannatha PS, Savanur SJ. Annual Risk of Tuberculosis Infection in Bangalore City. Indian J Tuberc. 2001; 48(2):63-71.
Chadha VK, Jithendra R, Kumar P, Kirankumar R, Shashidharan AN, Suganthi P, Gupta J, Jaganath PS. Change in the risk of tuberculous infection over an 8-year period among schoolchildren in Bangalore City. Int J Tuberc Lung Dis. 2008; 12(10): 1116-1121.
Chadha VK, Kumar P, Gupta J, Jagannatha PS, Magesh V, Jameel A, et al. The annual risk of tuberculous infection in the eastern zone of India. Int J Tuberc Lung Dis. 2004; 8(5):537–544.
Chadha VK, Kumar P, Satyanarayanan AVV, Chauhan LS, Gupta J, Singh S, Magesh V, Ahmed J, Srivastava R. Annual risk of tuberculous infection in Andhra Pradesh, India. Indian J Tuberc. 2007; 54(4): 177.
Chadha VK, Sarin R, Narang P, John KR, Chopra KK, Jitendra R, et al. Trends in the annual risk of tuberculous infection in India. Int J Tuberc Lung Dis. 2013;17(3):312–9.
Chadha VK, Vaidyanathan PS, Jagannatha PS, Unnikrishnan KP, Mini PA. Annual risk of tuberculous infection in the northern zone of India. Bull World Health Organ. 2003;81(8): 573–580.
Chadha VK, Vaidyanathan PS, Jagannatha PS, Unnikrishnan KP, Savanur SJ, Mini PA. Annual risk of tuberculous infection in the western zone of India. Int J Tuberc Lung Dis. 2003; 7(6):536–542.
Chadha VK, Kumar P, Anjinappa SM, Singh S, Narasimhaiah S, Joshi MV, Gupta J, Lakshminarayana, Ramchandra J, Velu M, Papkianathan S, Babu S, Krishna H. Prevalence of pulmonary tuberculosis among adults in a rural sub-district of South India. PLoS One. 2012; 7(8): e42625.
Chadha VK. Tuberculosis epidemiology in India: a review. Int J Tuberc Lung Dis. 2005; 9(10): 1072-82.
Chakma T, Rao PV, Pall S, Kaushal LS, Datta M, Tiwary RS. Survey of pulmonary tuberculosis in a primitive tribe of Madhya Pradesh. Indian J Tuberc. 1996; 43: 85-89.
Chakma T, Rao PV, Tiwary RS. Prevalence of anaemia and worm infestation in tribal areas of Madhya Pradesh. J Indian Med Assoc. 2000; 98(9): 567-571.

Table 3: GBD 2016 India data inputs
Chakraborty A.K, WHO Global Tuberculosis Programme. Prevalence and incidence of tuberculosis in India. A comprehensive review. World Health Organization;. 1997; p231.
Chakraborty AK, Chaudhuri K, Sreenivas TR, Murthy MK, Shashidhara AN, Channabasavaiah R. Tuberculous infection in a rural population of south India: 23-year trend. Tuber Lung Dis. 1992; 73(4): 213–218.
Chakraborty AK, Ganapathy KT, Gothi GD. Prevalence of infection among unvaccinated children for tuberculosis surveillance. Indian J Med Res. 1980; 72: 7–12.
Chakraborty AK, Singh H, Srikantan K, Rangaswamy KR, Krishnamurthy MS, Stephen JA, others. Tuberculosis in a rural population of south India: Report on five surveys. Indian J Tuberc. 1982; 29: 153–167.
Chakraborty B, Bhattacharya AR. Xerophthalmia in under-five children in West Bengal. Indian Pediatr. 1987; 24(7): 600-2.
Chakravarti A, Verma V, Jain M, Kar P. Characteristics of dual infection of hepatitis B and C viruses among patients with chronic liver disease: a study from tertiary care hospital. Trop Gastroenterol. 2005; 26(4): 183-7.
Chambial S, Shukla KK, Dwivedi S, Bhardwaj P, Sharma P. Blood Lead Level (BLL) in the Adult Population of Jodhpur: A Pilot Study. Indian J Clin Biochem. 2015; 30(3): 357-9.
Chand G, Barde PV, Singh N. Emergence of new foci of filariasis in Madhya Pradesh, India. Trans R Soc Trop Med Hyg. 2013; 107(7): 462-4.
Chand G, Pandey GD, Tiwary RS. Prevalence of Wuchereria bancrofti infection among the tribals of Panna district of Madhya Pradesh. J Commun Dis. 1996; 28(4): 304-7.
Chand G, Roy SK, Tiwary RS. Malaria epidemic in Lamta PHC of Balaghat district – a rice cultivating ecosystem. J Commun Dis. 1997; 29(2): 179-81.
Chand P, Rai RN, Chawla U, Tripathi KC, Datta KK. Epidemiology of measles--a thirteen years prospective study in a village. J Commun Dis. 1989; 21(3): 190-9.
Chandola TR, Taneja S, Goyal N, Rathore SS, Appaiahgari MB, Mishra A, Singh S, Vrati S, Bhandari N. Descriptive epidemiology of rotavirus infection in a community in North India. Epidemiol Infect. 2013; 141(10): 2094-100.
Chandra AK, Debnath A, Tripathy S. Iodine nutritional status among school children in selected areas of Howrah District in West Bengal, India. J Trop Pediatr. 2008; 54(1): 54-7.
Chandra AK, Ray I. Dietary supplies of iodine and thiocyanate in the etiology of endemic goiter in Tripura. Indian J Pediatr. 2001; 68(5): 399-404.
Chandra AK, Tripathy S, Lahari D, Mukhopadhyay S. Iodine nutritional status of school children in a rural area of Howrah district in the Gangetic West Bengal. Indian J Physiol Pharmacol. 2004; 48(2): 219-24.
Chandra BK, Singh G, Taneja N, Pahil S, Singhi S, Sharma M. Diarrhoeagenic Escherichia coli as a predominant cause of paediatric nosocomial diarrhoea in India. J Med Microbiol. 2012; 61(Pt 6): 830-6.
Chandra G, Chatterjee SN, Das S, Sarkar N. Lymphatic filariasis in the coastal areas of Digha, West Bengal, India. Trop Doct. 2007; 37(3): 136-9.
Chandra G, Hati AK. Filariasis survey in a rural area of West Bengal. J Commun Dis. 1996; 28(3): 206-8.
Chandra V, Ganguli M, Pandav R, Johnston J, Belle S, DeKosky ST. Prevalence of Alzheimer's disease and other dementias in rural India: the Indo-US study. Neurology. 1998; 51(4): 1000-8.
Chandrashekhar TS, Bhat HV, Pai RP, Nair SK. Prevalence of blindness and its causes among those aged 50 years and above in rural Karnataka, South India. Trop Doct. 2007; 37(1): 18-21.
Changlani TD, Jose A, Sudhakar A, Rojal R, Kunjikutty R, Vaidyanathan B. Outcomes of Infants with Prenatally Diagnosed Congenital Heart Disease Delivered in a Tertiary-care Pediatric Cardiac Facility. Indian Pediatr. 2015; 52(10): 852-6.
Charan R, Dogra MR, Gupta A, Narang A. The incidence of retinopathy of prematurity in a neonatal care unit. Indian J Ophthalmol. 1995; 43(3): 123-6.

Table 3: GBD 2016 India data inputs
Chatterjea JB. Haemoglobinopathies, glucose-6-phosphate dehydrogenase deficiency and allied problems in the Indian subcontinent. Bull World Health Organ. 1966; 35(6): 837-56.
Chaturvedi S, Kapil U, Gnanasekaran N, Sachdev HP, Pandey RM, Bhanti T. Nutrient intake amongst adolescent girls belonging to poor socioeconomic group of rural area of Rajasthan. Indian Pediatr. 1996; 33(3): 197-201.
Chaudhuri S, Mukherjee B, Roy Choudhury AK, Ghosh J. Study of blood groups and haemoglobin variants of the Sikhs in Calcutta. J Hered. 1967; 58(5): 213-4.
Chaudhuri V, Nanu A, Panda SK, Chand P. Evaluation of serologic screening of blood donors in India reveals a lack of correlation between anti-HBc titer and PCR-amplified HBV DNA. Transfusion. 2003; 43(10): 1442-8.
Chauhan P, Chauhan VKS, Shrivastava P. Maternal mortality among tribal women at a tertiary level of care in Bastar, Chhattisgarh. Glob J Health Sci. 2012; 4(2): 132-41.
Chavan BS, Arun P, Bhargava R, Singh GP. Prevalence of alcohol and drug dependence in rural and slum population of Chandigarh: A community survey. Indian J Psychiatry. 2007; 49(1): 44–8.
Chawla R, Bhalla P, Bhalla K, Singh MM, Garg S. Community-based study on seroprevalence of herpes simplex virus type 2 infection in New Delhi. Indian J Med Microbiol. 2008; 26(1): 34–9.
Cherian A, Syam UK, Sreevidya D, Jayaraman T, Oommen A, Rajshekhar V, Radhakrishnan K, Thomas SV. Low seroprevalence of systemic cysticercosis among patients with epilepsy in Kerala--South India. J Infect Public Health. 2014; 7(4): 271-6.
Cherian AG, Jamkhandi D, George K, Bose A, Prasad J, Minz S. Prevalence of Congenital Anomalies in a Secondary Care Hospital in South India: A Cross-Sectional Study. J Trop Pediatr. 2016; 62(5): 361–7.
Cherian T, Joseph A, John TJ. Low antibody response in infants with measles and children with subclinical measles virus infection. J Trop Med Hyg. 1984; 87(1): 27-31.
Chest Research Foundation Pune, KEM Hospital Research Centre Pune, Population Health and Occupational Disease, National Heart & Lung Institute, Imperial College London. India - Pune Burden of Obstructive Lung Disease Initiative Survey (BOLD) 2008-2009. [Data shared for this analysis]
Chhabra P, Garg S, Mittal SK, Satyanarayan L, Mehra M, Sharma N. Magnitude of acute respiratory infections in under five. Indian Pediatr. 1993; 11(30): 1315-9.
Chhabra S, Tyagi S, Bhavani M, Gosawi M. Late postpartum eclampsia. J Obstet Gynaecol. 2012; 32(3): 264-6.
Chhotray GP, Mohapatra M, Acharya AS, Ranjit MR. A clinico-epidemiological perspective of lymphatic filariasis in Satyabadi block of Puri district, Orissa. Indian J Med Res. 2001; 114: 65-71.
Chhotray GP, Ranjit MR, Khuntia HK, Acharya AS. Precontrol observations on lymphatic filariasis and geo-helminthiases in two coastal districts of rural Orrisa. Indian J Med Res. 2005; 122(5): 388-94.
Chidambaram N, Sethupathy S, Saravanan N, Mori M, Yamori Y, Garg AK, Chockalingam A. Relationship of sodium and magnesium intakes to hypertension proven by 24-hour urianalysis in a South Indian Population. J Clin Hypertens (Greenwich). 2014; 16: 581-586.
Child and Adolescent Health, Ministry of Health and Family Welfare, Government of India. State-wise data from India National Deworming Day 2015. New Delhi, India: Ministry of Health and Family Welfare. [Data shared for this analysis]
Child and Adolescent Health, Ministry of Health and Family Welfare, Government of India. State-wise data from India National Deworming Day 2016. New Delhi, India: Ministry of Health and Family Welfare. [Data shared for this analysis]
Chiplonkar S, Agte V, Mengale S. Relative importance of micronutrient deficiencies in iron deficiency anemia. Nutr Res. 2003; 23(10): 1355-67.

Table 3: GBD 2016 India data inputs
Chitambar SD, Chadha MS, Joshi MS, Arankalle VA. Prevalence of hepatitis a antibodies in western Indian population: changing pattern. Southeast Asian J Trop Med Public Health. 1999; 30(2): 273-6.
Chitsulo L, Engels D, Montresor A, Savioli L. The global status of schistosomiasis and its control. Acta Trop. 2000; 77(1): 41-51.
Cho SH, Lin HC, Ghoshal AG, Muttalif BA, Razak A, Thanaviratananich S, et al. Respiratory disease in the Asia-Pacific region: cough as a key symptom. In: Allergy Asthma Proc. OceanSide Publications, Inc; 2016; 37(2): 131–40.
Chockalingam K, Vedhachalam C, Rangasamy S, Sekar G, Adinarayanan S, Swaminathan S, Menon PA. Prevalence of tobacco use in urban, semi urban and rural areas in and around Chennai City, India. PLoS One. 2013; 8(10): e76005.
CHOICE Study Group. Multicenter, randomized, double-blind clinical trial to evaluate the efficacy and safety of a reduced osmolarity oral rehydration salts solution in children with acute watery diarrhea. Pediatrics. 2001; 107(4): 613-8.
Chopra A, Saluja M, Patil J, Tandale HS. Pain and disability, perceptions and beliefs of a rural Indian population: A WHO-ILAR COPCORD study. WHO-International League of Associations for Rheumatology. Community Oriented Program for Control of Rheumatic Diseases. J Rheumatol. 2002; 29(3): 614-21.
Chopra K, Chadha VK, Ramachandra J, Aggarwal N. Trend in Annual Risk of Tuberculous Infection in North India. McBryde ES, editor. PLoS ONE. 2012; 7(12):e51854.
Choudhary A, Gulati S, Sagar R, Kabra M, Sapra S. Behavioral comorbidity in children and adolescents with epilepsy. J Clin Neurosci. 2014; 21(8): 1337-40.
Choudhary M, Grover K, Javed M. Nutritional profiles of urban and rural men of Punjab with regard to dietary fat intake. Ecol Food Nutr. 2014; 53(4): 436–52.
Choudhury N, Ramesh V, Saraswat S, Naik S. Effectiveness of mandatory transmissible diseases screening in Indian blood donors. Indian J Med Res. 1995; 101: 229-32.
Chow C, Cardona M, Raju PK, Iyengar S, Sukumar A, Raju R, Colman S, Madhav P, Raju R, Reddy KS, Celermajer D, Neal B. Cardiovascular disease and risk factors among 345 adults in rural India--the Andhra Pradesh Rural Health Initiative. Int J Cardiol. 2007; 116(2): 180-5.
Chowdhary A, Malhotra VL, Deb M, Rai U. Screening for chlamydial infections in women with pelvic inflammatory diseases. J Commun Dis. 1998; 30(3): 163-6.
Chowdhary N, Patel V. The effect of spousal violence on women's health: findings from the Stree Arogya Shodh in Goa, India. J Postgrad Med. 2008; 54(4): 306–12.
Chowdhury A, Santra A, Chakravorty R, Banerji A, Pal S, Dhali GK, Datta S, Banerji S, Manna B, Chowdhury SR, Bhattacharya SK, Mazumder DG. Community-based epidemiology of hepatitis B virus infection in West Bengal, India: prevalence of hepatitis B e antigen-negative infection and associated viral variants. J Gastroenterol Hepatol. 2005; 20(11): 1712-20.
Chowdhury A, Santra A, Chaudhuri S, Dhali GK, Chaudhuri S, Maity SG, Naik TN, Bhattacharya SK, Mazumder DN. Hepatitis C virus infection in the general population: a community-based study in West Bengal, India. Hepatology. 2003; 37(4): 802-9.
Chowdhury S, Kumar R, Ganguly NK, Kumar L, Walia BNS. Effect of vitamin A supplementation on childhood morbidity and mortality. Indian J Med Sci. 2002; 56(6): 259-64. as it appears in Imdad A, Herzer K, Mayo-Wilson E, Yakoob MY, Bhutta ZA. Vitamin A supplementation for preventing morbidity and mortality in children from 6 months to 5 years of age. Cochrane Database Syst Rev. 2010; CD008524.
Chowgule RV, Shetye VM, Parmar JR, Bhosale AM, Khandagale MR, Phalnitkar SV, Gupta PC. Prevalence of respiratory symptoms, bronchial hyperreactivity, and asthma in a megacity. Results of the European community respiratory health survey in Mumbai (Bombay). Am J Respir Crit Care Med. 1998; 158(2): 547-54.
Christensen LB, Petersen PE, Bhambal A. Oral health and oral health behaviour among 11-13-year-olds in Bhopal, India. Community Dent Health. 2003; 20(3): 153-8.

**Table 3: GBD 2016 India data inputs**
Christian Medical College - Vellore, Fogarty International Center, National Institutes of Health, Foundation for the National Institutes of Health. India - Vellore Malnutrition and Enteric Disease (MAL-ED) Study 2009-2014. [Data shared for this analysis]
Christian Medical College - Vellore, MRC Epidemiology Resource Center, University of Southampton. India - Vellore Birth Cohort Study 1998-2002. [Data shared for this analysis]
Christian Medical College - Vellore. India Prevalence of Risk Factors for Non-Communicable Diseases in Rural and Urban Tamil Nadu 2010-2012. [Data shared for this analysis]
Chudasama RK, Verma PB, Mahajan RG. Iodine nutritional status and goiter prevalence in 6-12 years primary school children of Saurashtra region, India. World J Pediatr. 2010; 6(3): 233-7.
Climatic Research Unit, University of East Anglia. Climatic Research Unit-Time Series v.3.24 High Resolution Gridded Time-Series Dataset 1901-2015. Norwich, United Kingdom: University of East Anglia.
Coal Controller's Organization, Ministry of Coal, Government of India. India Provisional Coal Statistics 2014-2015. Kolkata, India: Coal Controller's Organization, Ministry of Coal; 2015.
Congenital Heart Anomalies Mortality Risk With No Diagnosis or Care Estimates as provided by the Global Burden of Disease 2010 Congenital Anomaly Expert Group. [Data shared for this analysis]
Corsi DJ, Subramanian SV, Lear SA, Teo KK, Boyle MH, Raju PK, Joshi R, Neal B, Chow CK. Tobacco use, smoking quit rates, and socioeconomic patterning among men and women: a cross-sectional survey in rural Andhra Pradesh, India. Eur J Prev Cardiol. 2014; 21(10): 1308-18.
Curado MP, Edwards B, Shin HR, Storm H, Ferlay J, Heanue M, Boyle P, eds. Cancer Incidence in Five Continents, Vol. IX. International Agency for Research on Cancer (IARC) Scientific Publications, No. 160, Lyon, France: IARC; 2007.
Cystic Echinococcosis Endemicity Estimates identified through systematic review and personal communication, as provided by GBD 2015 expert. [Data shared for this analysis]
Dahiya S, Kapoor AC. Anaemia in women in a selected rural area of Haryana: effect of dietary intake level. Indian J Nutr Diet. 1995; 224-8.
Dalal PM, Malik S, Bhattacharjee M, Trivedi ND, Vairale J, Bhat P, Deshmukh S, Khandelwal K, Mathur VD. Population-based stroke survey in Mumbai, India: incidence and 28-day case fatality. Neuroepidemiology. 2008; 31(4): 254-61.
Damle SC, Patel AR. Caries prevalence and treatment need amongst children of Dharavi, Bombay, India. Community Dent Oral Epidemiol. 1994; 22(1): 62-3.
Danawala SA, Arora M, Stigler MH. Analysis of motivating factors for smokeless tobacco use in two Indian states. Asian Pac J Cancer Prev. 2014; 15(16): 6553-8.
Dandi KK, Rao EV, Margabandhu S. Dental pain as a determinant of expressed need for dental care among 12-year-old school children in India. Indian J Dent Res. 2011; 22(4): 611.
Dandinarasaiah M, Vikram BK, Krishnamurthy N, Chetan AC, Jain A. Diphtheria Re-emergence: Problems Faced by Developing Countries. Indian J Otolaryngol Head Neck Surg. 2013; 65(4): 314-8.
Dandona L, Dandona R, Naduvilath TJ, McCarty CA, Srinivas M, Mandal P, Nanda A, Rao GN. Burden of moderate visual impairment in an urban population in southern India. Ophthalmology. 1999; 106(3): 497-504.
Dandona L, Dandona R, Srinivas M, Giridhar P, Vilas K, Prasad MN, John RK, McCarty CA, Rao GN. Blindness in the Indian state of Andhra Pradesh. Invest Ophthalmol Vis Sci. 2001; 42(5): 908-16.
Dandona R, Dandona L, Srinivas M, Giridhar P, Prasad MN, Vilas K, McCarty CA, Rao GN. Moderate visual impairment in India: the Andhra Pradesh Eye Disease Study. Br J Ophthalmol. 2002; 86(4): 373-7.
Dandona R, Kumar GA, Ameer MA, Ahmed GM, Dandona L. Hyderabad household road traffic injuries study 2005-06. [Data shared for this analysis]

Table 3: GBD 2016 India data inputs
Dandona R, Kumar GA, Ameer MA, Ahmed GM, Dandona L. Incidence and burden of road traffic injuries in urban India. Inj Prev. 2008; 14(6): 354-9.
Dandona R, Kumar GA, Ivers R, Joshi R, Neal B, Dandona L. Characteristics of non-fatal fall injuries in rural India. Inj Prev. 2010; 16(3): 166–71.
Dandona R, Rewari BB, Kumar GA, Tanwar S, Kumar SGP, Vishnumolakala VS, Duber HC, Gakidou E, Dandona L. Survival outcomes for first-line antiretroviral therapy in India's ART program. BMC Infect Dis. 2016; 16(1): 555.
Daniel CR, Prabhakaran D, Kapur K, Graubard BI, Devasenapathy N, Ramakrishnan L, George PS, Shetty H, Ferrucci LM, Yurgalevitch S, Chatterjee N, Reddy KS, Rastogi T, Gupta PC, Mathew A, Sinha R. A cross-sectional investigation of regional patterns of diet and cardio-metabolic risk in India. Open Nutr J. 2011; 10(1): 12.
Das D, Kumar S, Sahoo PK, Dash AP. A survey of bancroftian filariasis for microfilariae & circulating antigenaemia in two villages of Madhya Pradesh. Indian J Med Res. 2005; 121(6): 771-5.
Das D, Misra J, Mitra M, Bhattacharya B, Bagchi A. Prevalence of dental caries and treatment needs in children in coastal areas of West Bengal. Contemp Clin Dent. 2013; 4(4): 482-7.
Das DK, Chakraborty I, Biswas AB, Saha I, Mazumder P, Saha S. Goitre prevalence, urinary iodine and salt iodisation level in a district of West Bengal, India. J Am Coll Nutr. 2008; 27(3): 401-5.
Das K, Kar P, Chakraborty A, Gupta S, Das BC. Is a vaccination program against hepatitis A needed in India?. Indian J Gastroenterol. 1998; 17(4): 158.
Das LK, Pani SP, Vanamail P, Vijayalakshmi G, DeBritto LJ. Cost-effective antigen testing for delimitation, monitoring and evaluation in bancroftian filariasis. Eur J Clin Microbiol Infect Dis. 2012; 31(8): 2069-75.
Das M, Sharma SK, Sekhon GS, Saikia BJ, Mahanta J, Phukan RK. Promoter methylation of MGMT gene in serum of patients with esophageal squamous cell carcinoma in North East India. Asian Pac J Cancer Prev. 2014; 15(22): 9955-60.
Das NG, Bhuyan M, Das SC. Entomological and epidemiological studies on malaria in Rajmahal range, Bihar. Indian J Malariol. 2000; 37(3/4): 88-96.
Das PK, Das LK, Parida SK, Patra KP, Jambulingam P. Lambdacyhalothrin treated bed nets as an alternative method of malaria control in tribal villages of Koraput District, Orissa State, India. Southeast Asian J Trop Med Public Health. 1993; 24(3): 513-21.
Das PK, Harris VK, Shoma B, Bose YN, Annie S. Trend of hepatitis B virus infection in southern Indian blood donors. Indian J Gastroenterol. 1999; 18(4): 182.
Das PK, Manoharan A, Subramanian S, Ramaiah KD, Pani SP, Rajavel AR, Rajagopalan PK. Bancroftian filariasis in Pondicherry, south India--epidemiological impact of recovery of the vector population. Epidemiol Infect. 1992; 108(3): 483-93.
Das PK, Nair SC, Harris VK, Rose D, Mammen JJ, Bose YN, Sudarsanam A. Distribution of ABO and Rh-D blood groups among blood donors in a tertiary care centre in South India. Trop Doct. 2001; 31(1): 47-8.
Das PK, Ramaiah KD, Vanamail P, Pani SP, Yuvaraj J, Balarajan K, Bundy DAP. Placebo-controlled community trial of four cycles of single-dose diethylcarbamazine or ivermectin against Wuchereria bancrofti infection and transmission in India. Trans R Soc Trop Med Hyg. 2001; 95(3): 336-41.
Das S, Bapat U, More NS, Alcock G, Fernandez A, Osrin D. Nutritional status of young children in Mumbai slums: a follow-up anthropometric study. Open Nutr J. 2012; 11(1): 100.
Das S, Gupta S. Diversity of Vibrio cholerae Strains Isolated in Delhi, India, During 1992-2000. J Health Popul Nutr. 2005; 23(1): 44–51.
Das S, S. Neuroepidemiology of major neurological disorders in rural Bengal. Neurol India. 1996; 44(2): 47-58.

Table 3: GBD 2016 India data inputs
Das S, Sanyal K, Moitra A. A pilot study on neuroepidemiology in urban Bengal. Indian J Public Health. 1998; 42(2): 34-6.
Das SK, Banerjee TK, Biswas A, Roy T, Raut DK, Mukherjee CS, Chaudhuri A, Hazra A, Roy J. A Prospective Community-Based Study of Stroke in Kolkata, India. Stroke. 2007; 38(3): 906-10.
Das SK, Biswas A, Roy T, Banerjee TK, Mukherjee CS, Raut DK, Chaudhuri A. A random sample survey for prevalence of major neurological disorders in Kolkata. Indian J Med Res. 2006; 124(2): 163-72.
Das SK, Misra AK, Ray BK, Hazra A, Ghosal MK, Chaudhuri A, Roy T, Banerjee TK, Raut DK. Epidemiology of Parkinson disease in the city of Kolkata, India: a community-based study. Neurology. 2010; 75(15): 1362-9.
Das VNR, Siddiqui NA, Kumar N, Verma N, Verma RB, Dinesh DS, Kar SK, Das P. A pilot study on the status of lymphatic filariasis in a rural community of Bihar. J Commun Dis. 2006; 38(2): 169-75.
Dash S, Chhanhimi L, Chhakchhuak L, Zomawaia E. Screening for haemoglobinopathies and G6PD deficiency among the Mizos of Mizoram: a preliminary study. Indian J Pathol Microbiol. 2005; 48(1): 17–8.
Dass Hazarika R, Deka NM, Khyriem AB, Lyngdoh WV, Barman H, Duwarah SG, Jain P, Borthakur D. Invasive meningococcal infection: analysis of 110 cases from a tertiary care centre in North East India. Indian J Pediatr. 2013; 80(5): 359-64.
Datta M, Gopi PG, Appegowda BN, Bhima Rao KR, Gopalan BN. Tuberculosis in North Arcot District of Tamil Nadu - a sample survey. Indian J Tuberc. 2000; 47(3): 147-54.
Datta N, Kumar V, Kumar L, Singhi S. Application of case management to the control of acute respiratory infections in low-birth-weight infants: a feasibility study. Bull World Health Organ. 1987; 1(65): 77-82.
Dave U, Shetty N, Mehta L. A community genetics approach to population screening in India for mental retardation – a model for developing countries. Ann Hum Biol. 2005; 32(2): 195-203.
De SK, Chandra G. Studies on the filariasis vector--Culex quinquefasciatus at Kanchrapara, West Bengal (India). Indian J Med Res. 1994; 99: 255-8.
Deedwania PC, Gupta R, Sharma KK, Achari V, Gupta B, Maheshwari A, Gupta A. High prevalence of metabolic syndrome among urban subjects in India: a multisite study. Diabetes Metab Syndr. 2014; 8(3): 156–61.
Deepa M, Anjana RM, Manjula D, Narayan KMV, Mohan V. Convergence of prevalence rates of diabetes and cardiometabolic risk factors in middle and low income groups in urban India: 10-year follow-up of the Chennai Urban Population Study. J Diabetes Sci Technol. 2011; 5(4): 918–27.
Deepa M, Grace M, Binukumar B, Pradeepa R, Roopa S, Khan HM, et al. High burden of prediabetes and diabetes in three large cities in South Asia: The Center for cArdio-metabolic Risk Reduction in South Asia (CARRS) Study. Diabetes Res Clin Pract. 2015 Nov; 110(2):172–82.
Deivasigamani TR. Psychiatric morbidity in primary school children – an epidemiological study. Indian J Psychiatry. 1990; 32(3): 235-40.
Department of Communicable Disease Surveillance and Response, World Health Organization (WHO). WHO Report on Global Surveillance of Epidemic-prone Infectious Diseases 2000. Geneva, Switzerland: WHO; 2000.
Department of Health and Family Welfare, Government of Punjab, Postgraduate Institute of Medical Education & Research Chandigarh, University of Michigan. India - Punjab Noncommunicable Disease Risk Factors Survey 2014-2015. [Data shared for this analysis]
Department of Mental Health and Substance Abuse, World Health Organization (WHO). Alcohol, Gender, and Drinking Problems: Perspectives From Low and Middle Income Countries 1999-2003. Geneva, Switzerland: WHO; 2005.

Table 3: GBD 2016 India data inputs
Department of Pulmonary Medicine, Postgraduate Institute of Medical Education & Research. India Study on Epidemiology of Asthma, Respiratory Symptoms and Chronic Bronchitis (INSEARCH) 2004-2005. [Data shared for this analysis]
Department of Pulmonary Medicine, Postgraduate Institute of Medical Education & Research. India Study on Epidemiology of Asthma, Respiratory Symptoms and Chronic Bronchitis (INSEARCH) 2007-2009. [Data shared for this analysis]
Department of Pulmonary Medicine, Postgraduate Institute of Medical Education & Research. India Study on Epidemiology of Asthma, Respiratory Symptoms and Chronic Bronchitis 2004-2005. [Data shared for this analysis]
Department of Women and Child Development, Ministry of Human Resource Development, Government of India, United Nations Children's Fund (UNICEF) - India Country Office. India Summary Report on the Multiple Indicator Cluster Survey 2000. New Delhi, India: UNICEF India Country Office; 2000.
Department of Women and Child Development, Ministry of Human Resource Development, Government of India. India Nutrition Profile Survey 1995-1996. New Delhi, India: Ministry of Human Resource Development.
Derman RJ, Kodkany BS, Goudar SS, Geller SE, Naik VA, Bellad MB, Patted SS, Patel A, Edlavitch SA, Hartwell T, Chakraborty H, Moss N. Oral misoprostol in preventing postpartum haemorrhage in resource-poor communities: a randomised controlled trial. Lancet. 2006; 368(9543): 1248-53.
Desai Meena P, Khatkhatay MI, Bhanu Prakash KV, Savardekar LS, Shah RS, Ansari Z. Hormonal profiles and biochemical indices of bone turnover in Indian women. Osteoporos Int. 2007; 18(7): 923-9.
Desai, Sonalde, Reeve Vanneman, National Council of Applied Economic Research, University of Michigan. India Human Development Survey 2005. Ann Arbor, Michigan: Inter-University Consortium for Political and Social Research.
Deshmukh V, Lahariya C, Krishnamurthy S, Das MK, Pandey RM, Arora NK. Taken to Health Care Provider or Not, Under-Five Children Die of Preventable Causes: Findings from Cross-Sectional Survey and Social Autopsy in Rural India. Indian J Community Med. 2016; 41(2): 108–19.
Deshmukh VV, Sharma KD. Deficiency of erythrocyte glucose-6-phosphate dehydrogenase and sickle cell trait: a survey of Mahar students at Aurangabad, Maharashtra. Indian J Med Res. 1968; 56(6): 821-5.
Desjeux P, Ghosh RS, Dhalaria P, Strub-Wourgaft N, Zijlstra EE. Report of the Post Kala-Azar Dermal Leishmaniasis (PKDL) consortium meeting, New Delhi, India, 27–29 June 2012. Parasit Vectors. 2013; 6: 196.
Dev V, Phookan S, Sharma VP, Anand SP. Physiographic and entomologic risk factors of malaria in Assam, India. Am J Trop Med Hyg. 2004; 71(4): 451-6.
Dev V, Phookan S, Sharma VP, Dash AP, Anand SP. Malaria parasite burden and treatment seeking behavior in ethnic communities of Assam, Northeastern India. J Infect. 2006; 52(2): 131-9.
Dev V, Raghavendra K, Barman K, Phookan S, Dash AP. Wash-resistance and field efficacy of Olyset net, a permethrin-incorporated long-lasting insecticidal netting, against Anopheles minimus-transmitted malaria in Assam, Northeastern India. Vector Borne Zoonotic Dis. 2010; 10(4): 403-10.
Dev V. Malaria survey in Tarajulie tea estate and adjoining hamlets in Sonitpur District, Assam. Indian J Malariol. 1996; 33(1): 21-9.
Dhabadi BB, Athavale A, Meundi A, Rekha R, Suruliraman M, Shreeranga A, Gururaj S. Prevalence of asthma and associated factors among schoolchildren in rural South India. Int J Tuberc Lung Dis. 2012; 16(1): 120-5.
Dhake PV, Dole K, Khandekar R, Deshpande M. Prevalence and causes of avoidable blindness and severe visual impairment in a tribal district of Maharashtra, India. Oman J Ophthalmol. 2011; 4(3): 129-34.
Dhall K, Sarkar A, Sokhey C, Dhall GI, Ganguly NK. Incidence of gonococcal infection and its clinicopathological correlation in patients attending gynaecological outpatients department. J Obstet Gynaecol India. 1991; 40(3): 410-13.
Dhamija RK, Dhamija SB. Prevalence of stroke in rural community – an overview of Indian experience. J Assoc Physicians India. 1998; 46(4): 351-4.

Table 3: GBD 2016 India data inputs
Dhanaraj B, Papanna MK, Adinarayanan S, Vedachalam C, Sundaram V, Shanmugam S, Sekar G, Menon PA, Wares F, Swaminathan S. Prevalence and Risk Factors for Adult Pulmonary Tuberculosis in a Metropolitan City of South India. PLoS One. 2015; 10(4): e0124260.
Dhanoa J, Cowan B. Measles in the community: A study in non-hospitalised young children in Punjab. J Trop Pediatr. 1982; 28(2): 59-61.
Dhawan PS, Shah SS, Alvares JF, Kher A, Shankaran, Kandoth PW, Sheth PN, Kamath H, Kamath A, Koppikar GV, Kalro RH. Seroprevalence of hepatitis A virus in Mumbai, and immunogenicity and safety of hepatitis A vaccine. Indian J Gastroenterol. 1998; 17(1): 16-8.
Dhiman S, Goswami D, Rabha B, Yadav K, Chattopadhyay P, Veer V. Absence of asymptomatic malaria in a cohort of 133 individuals in a malaria endemic area of Assam, India. BMC Public Health. 2015; 15: 919.
Dikshit RP, Kanhere S. Tobacco habits and risk of lung, oropharyngeal and oral cavity cancer: a population-based case-control study in Bhopal, India. Int J Epidemiol. 2000; 29(4): 609-14.
Directorate of Economics & Statistics and Office of Chief Registrar (Births & Deaths), Government of National Capital Territory of Delhi. Report on Medical Certification of Cause of Deaths in Delhi-2011. New Delhi, India: Directorate of Economics & Statistics and Office of Chief Registrar (Births & Deaths); 2012.
Directorate of Economics & Statistics and Office of Chief Registrar (Births & Deaths), Government of National Capital Territory of Delhi. Report on Medical Certification of Cause of Deaths in Delhi-2012. New Delhi, India: Directorate of Economics & Statistics and Office of Chief Registrar (Births & Deaths); 2014.
Directorate of Economics & Statistics and Office of Chief Registrar (Births & Deaths), Government of National Capital Territory of Delhi. Report on Medical Certification of Cause of Deaths in Delhi-2013. New Delhi, India: Directorate of Economics & Statistics and Office of Chief Registrar (Births & Deaths); 2014.
Dixit V, Kurup AV, Gupta AK, Kataria OM, Prasad GB. Bancroftian filariasis in south east Madhya Pradesh: Pre-control epidemilogical observations. Indian J Clin Biochem. 1997; 12(Suppl 1): 39-43.
Dodd NS, Samuel AM. Iodine deficiency in adolescents from Bombay slums. Natl Med J India. 1993; 6(3): 110-3.
Dogra S, Kumar B. Epidemiology of skin diseases in school children: a study from northern India. Pediatr Dermatol. 2003; 20(6): 470-3.
Dogra V, Khanna R, Jain A, Kumar AMV, Shewade HD, Majumdar SS. Neonatal mortality in India’s rural poor: Findings of a household survey and verbal autopsy study in Rajasthan, Bihar and Odisha. J Trop Pediatr. 2015; 61(3): 210–4.
Doll R,Muir CS, Waterhouse JAH, eds. Cancer Incidence in Five Continents, Vol. II. Geneva, Switzerland: Union Internationale Contre le Cancer; 1970.
Dorairaj SK, Bandrakalli P, Shetty C, R V, Misquith D, Ritch R. Childhood blindness in a rural population of southern India: prevalence and etiology. Ophthalmic Epidemiol. 2008; 15(3): 176-82.
Dr N T R Vaidya Seva Trust, Government of Andhra Pradesh. India - Andhra Pradesh State Aarogyasri Dialysis 2007-2017 and Renal Transplant 2007-2017 Data. [Data shared for this analysis]
D’Sa JA. Epidemiology of Pertussis. In: Jucker E, editor. Progress in Drug Research. Basel: Birkhäuser Basel; 1975. p. 257–62.
D'Souza SJ, Narurkar LM, Narurkar MV. Effect of environmental exposures to lead and cadmium on human lymphocytic detoxifying enzymes. Bull Environ Contam Toxicol. 1994; 53(3): 458-63.
Dubey ML, Sharma SK, Ganguly NK, Mahajan RC. Seroepidemiological study of malaria in a rural population of Chandigarh. Indian J Malariol. 1989; 26(4): 187-90.
Dutta AK, Aggarwal A, Kapoor AK, Ray GN, Batra S. Seroepidemiology of hepatitis A in Delhi. Indian J Pediatr. 2000; 67(2): 77-9.

Table 3: GBD 2016 India data inputs
Dyson PA, Anthony D, Fenton B, Matthews DR, Stevens DE, Community Interventions for Health Collaboration. High rates of child hypertension associated with obesity: a community survey in China, India and Mexico. Paediatr Int Child Health. 2014; 34(1): 43–9.
Elkins DB, Haswell-Elkins M, Anderson RM. The importance of host age and sex to patterns of reinfection with Ascaris lumbricoides following mass anthelmintic treatment in a South Indian fishing community. Parasitology. 1988; 96 (Pt 1): 171–84.
Ellwood P, International Study of Asthma and Allergies in Childhood (ISAAC) Steering Committee, ISAAC Phase Three Study Group, ISAAC International Data Centre. ISAAC: International Study of Asthma and Allergies in Childhood: phase three manual. Auckland, New Zealand: ISAAC International Data Centre; 2000.
Embassy of the United States - New Delhi, United States Environmental Protection Agency. United States Mission India NowCast Air Quality Data 2014. New Delhi, India: Embassy of the United States.
Ercumen A, Arnold BF, Kumpel E, Burt Z, Ray I, Nelson K, Colford JM. Upgrading a Piped Water Supply from Intermittent to Continuous Delivery and Association with Waterborne Illness: A Matched Cohort Study in Urban India. PLoS Med. 2015; 12(10): e1001892.
Esser MB, Gururaj G, Rao GN, Jernigan DH, Murthy P, Jayarajan D, Lakshmanan S, Benegal V, Collaborators Group on Epidemiological Study of Patterns and Consequences of Alcohol Misuse in India. Harms to Adults from Others' Heavy Drinking in Five Indian States. Alcohol Alcohol. 2016; 51(2): 177–85.
Euromonitor International. Euromonitor Passport - Cigarette Statistics 1999-2016. London, United Kingdom: Euromonitor International.
Euromonitor International. Euromonitor Passport - Dairy Market Statistics 2002-2016. London, United Kingdom: Euromonitor International.
Euromonitor International. Euromonitor Passport - Drinking Milk Products Market Statistics. London, United Kingdom: Euromonitor International. Available from: http://www.euromonitor.com/drinking-milk-products
Euromonitor International. Euromonitor Passport - Fresh Foods Market Statistics 2011-2016. London, United Kingdom: Euromonitor International.
Euromonitor International. Euromonitor Passport - Fruit Market Statistics 2001-2016. London, United Kingdom: Euromonitor International.
Euromonitor International. Euromonitor Passport - Meat Market Statistics 2001-2016. London, United Kingdom: Euromonitor International.
Euromonitor International. Euromonitor Passport - Nuts Market Statistics 2001-2016. London, United Kingdom: Euromonitor International.
Euromonitor International. Euromonitor Passport - Processed Meat and Seafood Market Statistics 2002-2016. London, United Kingdom: Euromonitor International.
Euromonitor International. Euromonitor Passport - Pulses Market Statistics 2001-2016. London, United Kingdom: Euromonitor International.
Euromonitor International. Euromonitor Passport - Vegetables Market Statistics 2001-2016. London, United Kingdom: Euromonitor International.
Euromonitor International. Partially Hydrogenated Vegetable Oil Sales Database 1998-2010. London, United Kingdom: Euromonitor International.
European Community Respiratory Health Survey Study Group. European Community Respiratory Health Survey 1992-2003 as provided by the Global Burden of Disease 2010 Respiratory Diseases Expert Group. [Data shared for this analysis]
Fakhir S, Srivastava I, Ahmad P, Hasan SS. Prevalence of xerophthalmia in pre-school children in an urban slum. Indian Pediatr. 1993; 30(5): 668-70.

Table 3: GBD 2016 India data inputs
Fall CHD, Sachdev HS, Osmond C, Lakshmy R, Biswas SD, Prabhakaran D, Tandon N, Ramji S, Reddy KS, Barker DJP, Bhargava SK. Adult metabolic syndrome and impaired glucose tolerance are associated with different patterns of BMI gain during infancy: Data from the New Delhi Birth Cohort. Diabetes Care. 2008; 31(12): 2349-56.
FAO Supply Utilization Accounts 1961-2013. Personal Correspondence with Dr. Josef Schmidhuber, 2016. [Data shared for this analysis]
Farooqui H, Jit M, Heymann DL, Zodpey S. Burden of Severe Pneumonia, Pneumococcal Pneumonia and Pneumonia Deaths in Indian States: Modelling Based Estimates. PLoS One. 2015; 10(6): e0129191.
Feldon K, Bahl S, Bhatnagar P, Wenger J. Severe vitamin A deficiency in India during pulse polio immunization. Indian J Med Res. 2005; 122(3): 265-7.
Ferri CP, Schoenborn C, Kalra L, Acosta D, Guerra M, Huang Y, Jacob KS, Rodriguez JJL, Salas A, Sosa AL, Williams JD, Liu Z, Moriyama T, Valhuerdi A, Prince MJ. Prevalence of stroke and related burden among older people living in Latin America, India and China. J Neurol Neurosurg Psychiatr. 2011; 82(10): 1074-82.
Flohr C, Weiland SK, Weinmayr G, Björkstén B, Bråbäck L, Brunekreef B, Büchele G, Clausen M, Cookson WOC, von Mutius E, Strachan DP, Williams HC, ISAAC Phase Two Study Group. The role of atopic sensitization in flexural eczema: findings from the International Study of Asthma and Allergies in Childhood Phase Two. J Allergy Clin Immunol. 2008; 121(1): 141-147.
Flohr C, Weinmayr G, Weiland SK, Addo-Yobo E, Annesi-Maesano I, Björkstén B, Bråbäck L, Büchele G, Chico M, Cooper P, Clausen M, El Sharif N, Martinez Gimeno A, Mathur RS, von Mutius E, Morales Suarez-Varela M, Pearce N, Svabe V, Wong GWK, Yu M, Zhong NS, Williams HC, ISAAC Phase Two Study Group. How well do questionnaires perform compared with physical examination in detecting flexural eczema? Findings from the International Study of Asthma and Allergies in Childhood (ISAAC) Phase Two. Br J Dermatol. 2009; 161(4): 846-53.
Food and Agriculture Organization of the United Nations (FAO). FAOSTAT Commodity Balances - Crops Primary Equivalent. Rome, Italy: FAO.
Food and Agriculture Organization of the United Nations (FAO). FAOSTAT Food Balance Sheets, April 2015. Rome, Italy: FAO.
Food and Agriculture Organization of the United Nations (FAO). FAOSTAT Food Balance Sheets, October 2014. Rome, Italy: FAO.
Ford Foundation, Nutrition Foundation of India. India Profiles of Undernutrition and Underdevelopment: Studies of Poor Communities in Seven Regions of the Country. New Delhi, India: Nutrition Foundation of India. (Scientific Report Series No. 8).
Forman D, Bray F, Brewster DH, Gombe Mbalawa C, Kohler B, Piñeros M, Steliarova-Foucher E, Swaminathan R,Ferlay J, eds. Cancer Incidence in Five Continents, Vol. X. International Agency for Research on Cancer (IARC) Scientific Publications, No. 164. Lyon, France: IARC; 2014.
Friberg L, Vahter M. Assessment of exposure to lead and cadmium through biological monitoring: results of a UNEP/WHO global study. Environ Res. 1983; 30(1): 95-128.
Frimodt-Møller J. A community-wide tuberculosis survey in a South Indian rural population, 1950-55. Bull World Health Organ. 1960; 22(1–2): 61.
Gadgil PS, Fadnis RS, Joshi MS, Rao PS, Chitambar SD. Seroepidemiology of hepatitis A in voluntary blood donors from Pune, western India (2002 and 2004-2005). Epidemiol Infect. 2008; 136(3): 406-9.
Gajalakshmi V, Peto R, Kanimozhi VC, Whitlock G, Veeramani D. Cohort Profile: the Chennai prospective study of mortality among 500,000 adults in Tamil Nadu, South India. Int J Epidemiol. 2007; 36(6): 1190-5.
Gambhir IS, Jaiswal JP, Nath G. Significance of Cryptosporidium as an aetiology of acute infectious diarrhoea in elderly Indians. Trop Med Int Health. 2003; 8(5): 415-9.

Table 3: GBD 2016 India data inputs
Ganguly S, Saha P, Guha SK, Biswas A, Das S, Kundu PK, Maji AK. High prevalence of asymptomatic malaria in a tribal population in eastern India. J Clin Microbiol. 2013; 51(5): 1439-44.
Ganju SA, Goel A. Sero-surveillance of HIV, HBV and HCV infections in antenatal and STD clinic attendees. J Commun Dis. 2004; 36(1): 60-2.
Garai R, Chakraborty AK. Measles in a rural community. Indian J Public Health. 1981; 24(3): 150-3.
Garcia-Marcos L, Robertson CF, Ross Anderson H, Ellwood P, Williams HC, Wong GW. Does migration affect asthma, rhinoconjunctivitis and eczema prevalence? Global findings from the international study of asthma and allergies in childhood. Int J Epidemiol. 2014; 43(6): 1846-54.
Garg A, Bhatia BD, Chaturvedi P, Garg S. G6PD deficiency in newborn infants. Indian J Pediatr. 1984; 51(408): 29-33.
Garg R, Kaur S, Aseri R, Aggarwal S, Singh JP, Mann S, Kumar S, Kaur S. Hepatitis B & C among farmers - a seroprevalence study. J Clin Diagn Res. 2014; 8.0(11): MC07-9.
Garg S, Mathur DR, Garg DK. Comparison of seropositivity of HIV, HBV, HCV and syphilis in replacement and voluntary blood donors in western India. Indian J Pathol Microbiol. 2001; 44(4): 409-12.
Gaur DR, Sood AK, Gupta VP. Goitre in school girls of the Mewat area of Haryana. Indian Pediatr. 1989; 26(3): 223-7.
Gayen P, Maitra S, Datta S, Babu SPS. Evidence for Wolbachia symbiosis in microfilariae of Wuchereria bancrofti from West Bengal, India. J Biosci. 2010; 35(1): 73-7.
GD Gothi, AK Chakraborty, SS Nair, KT Ganapathy, GC Banerjee. Prevalence of tuberculosis in a South Indian District - twelve years after inital survey. Indian J Tuberc. 1979; 26: 121-35.
Geetha L, Deepa M, Anjana RM, Mohan V. Prevalence and clinical profile of metabolic obesity and phenotypic obesity in Asian Indians. J Diabetes Sci Technol. 2011; 5(2): 439–46.
Gemmy Cheung CM, Li X, Cheng C-Y, Zheng Y, Mitchell P, Wang JJ, Jonas JB, Nangia V, Wong TY. Prevalence and risk factors for age-related macular degeneration in Indians: a comparative study in Singapore and India. Am J Ophthalmol. 2013; 155(4): 764–73.
George B, Padmam MSR, Nair MKC, Indira MS, Syamalan K, Padmamohan J. Hypoxic ischemic encephalopathy developmental outcome at 12 years. Indian Pediatr. 2009; 46(Suppl): S67-70.
George K, Prasad J, Singh D, Minz S, Albert DS, Muliyil J, Joseph KS, Jayaraman J, Kramer MS. Perinatal outcomes in a South Asian setting with high rates of low birth weight. BMC Pregnancy Childbirth. 2009 Feb; 9:5.
George S, Levecke B, Kattula D, Velusamy V, Roy S, Geldhof P, Sarkar R, Kang G. Molecular Identification of Hookworm Isolates in Humans, Dogs and Soil in a Tribal Area in Tamil Nadu, India. PLoS Negl Trop Dis. 2016; 10(8): e0004891.
Ghanwat GH, Patil AJ, Patil JA, Kshirsagar MS, Sontakke A, Ayachit RK. Biochemical effects of lead exposure on oxidative stress and antioxidant status of battery manufacturing workers of Western Maharashtra, India. J Basic Clin Physiol Pharmacol. 2016; 27(2): 141-6.
Ghosh A, Bala SK. Anthropometric, body composition, and blood pressure measures among rural elderly adults of Asian Indian origin: the Santiniketan aging study. J Nutr Gerontol Geriatr. 2011; 30(3): 305–13.
Ghosh I, Ghosh P, Bharti AC, Mandal R, Biswas J, Basu P. Prevalence of human papillomavirus and co-existent sexually transmitted infections among female sex workers, men having sex with men and injectable drug abusers from eastern India. Asian Pac J Cancer Prev. 2012; 13(3): 799–802.
Ghosh R, Bharati P. Haemoglobin status of adult women of two ethnic groups living in a peri-urban area of Kolkata city, India: a micro-level study. Asia Pac J Clin Nutr. 2003; 12(4): 451-9.
Ghosh R, Sharma AK. Determinants of tetanus and sepsis among the last neonatal deaths at household level in a peri-urban area of India. Postgrad Med J. 2011; 87(1026): 257-63.
Ghosh S, Samanta A, Mukherjee S. Patterns of alcohol consumption among male adults at a slum in Kolkata, India. J Health Popul Nutr. 2012; 30(1): 73–81.

Table 3: GBD 2016 India data inputs
Ghosh SK, Tiwari SN, Sathyanarayan TS, Sampath TRR, Sharma VP, Nanda N, Joshi H, Adak T, Subbarao SK. Larvivorous fish in wells target the malaria vector sibling species of the Anopheles culicifacies complex in villages in Karnataka, India. Trans R Soc Trop Med Hyg. 2005; 99(2): 101-5.
Ghosh SK, Yadav RS, Das BS, Sharma VP. Influence of nutritional and haemoglobin status on malaria infection in children. Indian J Pediatr. 1995; 62(3): 321-6.
Ghosh U, Banerjee T, Banerjee PK, Saha N. Distribution of haemoglobin and glucose-6-phosphate dehydrogenase phenotypes among different caste groups of Bengal. Hum Hered. 1981; 31(2): 119-21.
Ghulam R, Rahman I, Naqvi S, Gupta SR. An epidemiological study of drug abuse in urban population of Madhya Pradesh. Indian J Psychiatry. 1996; 38(3): 160–5.
GIDEON Informatics. Global Infectious Disease and Epidemiology Online Network (GIDEON) Database - Cholera. Los Angeles, United States: GIDEON Informatics. Available from: https://www.gideononline.com/about/gideon/
Gilbert CE, Ellwein LB. Prevalence and causes of functional low vision in school-age children: results from standardized population surveys in Asia, Africa, and Latin America. Invest Ophthalmol Vis Sci. 2008; 49(3): 877-81.
Gill HK, Yadav SB, Ramesh V, Bhatia E. A prospective study of prevalence and association of peripheral neuropathy in Indian patients with newly diagnosed type 2 diabetes mellitus. J Postgrad Med. 2014; 60(3): 270–5.
Girish G, Chawla D, Agarwal R, Paul VK, Deorari AK. Efficacy of two dose regimes of intravenous immunoglobulin in Rh hemolytic disease of newborn--a randomized controlled trial. Indian Pediatr. 2008; 45(8): 653-9.
Gladstone BP, Das AR, Rehman AM, Jaffar S, Estes MK, Muliyil J, Kang G, Bose A. Burden of illness in the first 3 years of life in an Indian slum. J Trop Pediatr. 2010; 56(4): 221-6.
Global Burden of Disease Health Financing Collaborator Network, Institute for Health Metrics and Evaluation (IHME). Global Development Assistance for Health, Government, Prepaid Private, and Out-of-Pocket Health Spending 1995-2014. Seattle, United States: IHME; 2017.
Gnanappa GK, Ganigara M, Prabhu A, Varma SK, Murmu U, Varghese R, Valliatu J, Kumar RNS. Outcome of complex adult congenital heart surgery in the developing world. Congenit Heart Dis. 2011; 6(1): 2-8.
Goel D, Agarwal A, Dhanai JS, Semval VD, Mehrotra V, Saxena V, Maithili B. Comprehensive rural epilepsy surveillance programme in Uttarakhand state of India. Neurol India. 2009; 57(3): 355–6.
Goel D, Dhanai JS, Agarwal A, Mehlotra V, Saxena V. Neurocysticercosis and its impact on crude prevalence rate of epilepsy in an Indian community. Neurol India. 2011; 59(1): 37-40.
Gogte ST, Basu N, Sinclair S, Ghai OP, Bhide NK. Blood lead levels of children with pica and surma use. Indian J Pediatr. 1991; 58(4): 513-9.
Gomber S, Kumar S, Rusia U, Gupta P, Agarwal KN, Sharma S. Prevalence & etiology of nutritional anaemias in early childhood in an urban slum. Indian J Med Res. 1998; 107: 269-73.
Gopaldas T, Kale M. Prophylactic iron supplementation for underprivileged school boys. I. Two levels of dosing and efficacy of teacher-distributions. Indian Pediatr. 1985; 22(10): 731-6.
Gopi PG, Subramani R, Radhakrishna S, Kolappan C, Sadacharam K, Devi TS, Frieden TR, Narayanan PR. A baseline survey of the prevalence of tuberculosis in a community in south India at the commencement of a DOTS programme. Int J Tuberc Lung Dis. 2003; 7(12): 1154-62.
Goswami AK, Gupta SK, Kalaivani M, Nongkynrih B, Pandav CS. Burden of Hypertension and Diabetes among Urban Population Aged ≥ 60 years in South Delhi: A Community Based Study. J Clin Diagn Res. 2016; 10(3): LC01-05.
Goswami D, Rathore AM, Batra S, Dubey C, Tyagi S, Wadhwa L. Facility-based review of 296 maternal deaths at a tertiary centre in India: Could they be prevented?. J Obstet Gynaecol Res. 2013; 39(12): 1569-79.

**Table 3: GBD 2016 India data inputs**
Goudar SS, Goco N, Somannavar MS, Vernekar SS, Mallapur AA, Moore JL, Wallace DD, Sloan NL, Patel A, Hibberd PL, Koso-Thomas M, McClure EM, Goldenberg RL. Institutional deliveries and perinatal and neonatal mortality in Southern and Central India. Reprod Health. 2015; 12(Suppl 2): S13.
Gourie-Devi M, Gururaj G, Satishchandra P, Subbakrishna DK. Neuro-epidemiological pilot survey of an urban population in a developing country. A study in Bangalore, south India. Neuroepidemiology. 1996; 15(6): 313-20.
Gourie-Devi M, Gururaj G, Satishchandra P, Subbakrishna DK. Prevalence of neurological disorders in Bangalore, India: a community-based study with a comparison between urban and rural areas. Neuroepidemiology. 2004; 23(6): 261-8.
Government Medical College - Thiruvananthapuram, Ananthapuri Hospitals and Research Institute, Cosmopolitan Hospital, Jubilee Memorial Hospital. India - Trivandrum Heart Failure Registry 2013. [Data shared for this analysis]
Government of Orissa, Micronutritent Initiative, United Nations Children's Fund, World Health Organization.Orissa Impact of Vitamin A Supplementation Delivered with Oral Polio Vaccine as Part of Immunization Campaign [Draft final report]. Bhubheshwar, India: Government of Orissa; 2001.
Goyal A, Gauba K, Chawla HS, Kaur M, Kapur A. Epidemiology of dental caries in Chandigarh school children and trends over the last 25 years. J Indian Soc Pedod Prev Dent. 2007; 25(3): 115-8.
Graham DY, Adam E, Reddy GT, Agarwal JP, Agarwal R, Evans DJ Jr, Malaty HM, Evans DG. Seroepidemiology of Helicobacter pylori infection in India. Comparison of developing and developed countries. Dig Dis Sci. 1991; 36(8): 1084-8.
Greenland K, Dixon R, Khan SA, Gunawardena K, Kihara JH, Smith JL, Drake L, Makkar P, Raman S, Singh S, Kumar S. The epidemiology of soil-transmitted helminths in Bihar State, India. PLoS Negl Trop Dis. 2015; 9(5): e0003790.
Grewal H, Verma M, Kumar A. Prevalence of dental caries and treatment needs amongst the school children of three educational zones of urban Delhi, India. Indian J Dent Res. 2011; 22(4): 517-9.
Grills N, Grills C, Spelman T, Stoove M, Hellard M, El-Hayek C, Singh R. Prevalence survey of dermatological conditions in mountainous north India. Int J Dermatol. 2012; 51(5): 579-87.
Grimsrud A, Balkan S, Casas EC, Lujan J, Van Cutsem G, Poulet E, Myer L, Pujades-Rodriguez M. Outcomes of antiretroviral therapy over a 10-year period of expansion: a multicohort analysis of African and Asian HIV programs. J Acquir Immune Defic Syndr. 2014; 67(2): e55–66.
Grover S, Ranyal RK, Bedi MK. A cross section of skin diseases in rural Allahabad. Indian J Dermatol. 2008; 53(4): 179-81.
Gubler DJ, Meltzer M. Impact of dengue/dengue hemorrhagic fever on the developing world. Adv Virus Res. 1999; 53: 35-70.
Guddattu V, Swathi A, Nair NS. Household and environment factors associated with asthma among Indian women: a multilevel approach. J Asthma. 2010; 47(4): 407-11.
Guerra M, Prina AM, Ferri CP, Acosta D, Gallardo S, Huang Y, Jacob KS, Jimenez-Velazquez IZ, Llibre Rodriguez JJ, Liu Z, Salas A, Sosa AL, Williams JD, Uwakwe R, Prince M. A comparative cross-cultural study of the prevalence of late life depression in low and middle income countries. J Affect Disord. 2016; 190: 362–8.
Guin G, Sahu B, Khare S, Kavishwar A. Trends in Maternal Mortality and Impact of Janani Suraksha Yojana (JSY) on Maternal Mortality Ratio in a Tertiary Referral Hospital. J Obstet Gynaecol India. 2012; 62(3): 307-11.
Gupta A, Gupta R, Sarna M, Rastogi S, Gupta VP, Kothari K. Prevalence of diabetes, impaired fasting glucose and insulin resistance syndrome in an urban Indian population. Diabetes Res Clin Pract. 2003; 61(1): 69-76.
Gupta B, Sharma S. Measles Outbreak in a rural area near Shimla. Indian J Community Med. 2006; 31(2): 106.
Gupta BP, Swami HM, Bhardwaj AK, Vaidya NK, Sharma CD, Kaushal RK. An outbreak of measles in a remote tribal area of Himachal Pradesh. Indian J Community Health. 1985; 5: 25-28.
Gupta DN, Sircar BK, Sengupta PG, Ghosh S, Banu MK, Mondal SK, Saha DR, De SP, Sikdar SN, Manna B, Dutta S, Saha NC. Epidemiological and clinical profiles of acute invasive diarrhoea with special reference to mucoid episodes: a rural community-based longitudinal study. Trans R Soc Trop Med Hyg. 1996; 90(5): 544-7.

Table 3: GBD 2016 India data inputs
Gupta I, Gupta ML, Parihar A, Gupta CD. Epidemiology of rheumatic and congenital heart diseases in school children. J Indian Med Assoc. 1992; 90(3): 57-9.
Gupta M, Kumar R, Deb AK, Bhattacharya SK, Bose A, John J, Balraj V, Ganguly NK, Kant L, Kapoor AN, Watt J, Shearer J, Santosham M, Hib study working group. Multi-center surveillance for pneumonia & meningitis among children (<2 yr) for Hib vaccine probe trial preparation in India. Indian J Med Res. 2010; 131: 649–58.
Gupta N, Kumar S, Saxena NC, Nandan D, Saxena BN. Maternal mortality in seven districts of Uttar Pradesh - an ICMR task force study. Indian J Public Health. 2006; 50(3): 173-8.
Gupta N, Kumar V, Kaur A. Seroprevalence of HIV, HBV, HCV and syphilis in voluntary blood donors. Indian J Med Sci. 2004; 58(6): 255-7.
Gupta PC, Mehta HC. Cohort study of all-cause mortality among tobacco users in Mumbai, India. Bull World Health Organ. 2000;78(7):877–883.
Gupta PC, Sinor PN, Bhonsle RB, Pawar VS, Mehta HC. Oral submucous fibrosis in India: a new epidemic? Natl Med J India. 1998; 11(3): 113-6.
Gupta PC, Subramoney S, Sreevidya S. Smokeless tobacco use, birth weight, and gestational age: population based, prospective cohort study of 1217 women in Mumbai, India. BMJ. 2004; 328(7455): 1538.
Gupta PC. Mumbai Cohort Study Datasets on Tobacco Use, 1991-1997. [Data shared for this analysis]
Gupta PC. Tobacco associated mortality in Mumbai (Bombay) India. Results of the Bombay Cohort Study. Int J Epidemiol. 2005;34(6):1395–402.
Gupta R, Deedwania PC, Achari V, Asirvatham AJ, Bhansali A, Gupta A, Gupta B, Gupta S, Jali MV, Mahanta TG, Maheshwari A, Saboo B, Singh J. India Heart Watch Study 2005-2009. [Data shared for this analysis]
Gupta R, Gupta VP, Ahluwalia NS. Educational status, coronary heart disease, and coronary risk factor prevalence in a rural population of India. BMJ. 1994 Nov 19; 309(6965):1332–6.
Gupta R, Gupta VP, Sarna M, Bhatnagar S, Thanvi J, Sharma V, et al. Prevalence of coronary heart disease and risk factors in an urban Indian population: Jaipur Heart Watch-2. Indian Heart J. 2002 Feb;54(1):59–66.
Gupta R, Gupta VP, Sarna M, Prakash H, Rastogi S, Gupta KD. Serial epidemiological surveys in an urban Indian population demonstrate increasing coronary risk factors among the lower socioeconomic strata. J Assoc Physicians India. 2003 May; 51:470–7.
Gupta R, Lodha S, Sharma KK, Sharma SK, Gupta S, Asirvatham AJ, et al. Evaluation of statin prescriptions in type 2 diabetes: India Heart Watch-2. BMJ Open Diabetes Res Care. 2016;4(1): e000275.
Gupta R, Misra A, Vikram NK, Kondal D, Gupta SS, Agrawal A, Pandey RM. Younger age of escalation of cardiovascular risk factors in Asian Indian subjects. BMC Cardiovasc Disord. 2009; 9:28.
Gupta R, Pandey RM, Misra A, Agrawal A, Misra P, Dey S, Rao S, Menon VU, Kamalamma N, Vasantha Devi KP, Revathi K, Vikram NK, Sharma V, Guptha S. High prevalence and low awareness, treatment and control of hypertension in Asian Indian women. J Hum Hypertens. 2012; 26(10): 585–93.
Gupta R, Prakash H, Majumdar S, Sharma S, Gupta VP. Prevalence of coronary heart disease and coronary risk factors in an urban population of Rajasthan. Indian Heart J. 1995 Aug; 47(4):331–8.
Gupta R, Sarna M, Thanvi J, Rastogi P, Kaul V, Gupta VP. High prevalence of multiple coronary risk factors in Punjabi Bhatia community: Jaipur Heart Watch-3. Indian Heart J. 2004; 56(6): 646-52.
Gupta R, Sharma AK, Gupta VP, Bhatnagar S, Rastogi S, Deedwania PC. Increased variance in blood pressure distribution and changing hypertension prevalence in an urban Indian population. J Hum Hypertens. 2003; 17(8): 535-40.
Gupta R, Sharma KK, Gupta BK, Gupta A, Saboo B, Maheshwari A, et al. Geographic epidemiology of cardiometabolic risk factors in middle class urban residents in India: cross-sectional study. J Glob Health. 2015 Jun;5(1):010411.

Table 3: GBD 2016 India data inputs
Gupta R. India - Jaipur Heart Watch Study Data on Blood Pressure, Cholesterol, BMI, and Fasting Blood Glucose 1993-2001. [Data shared for this analysis]
Gupta SK, Pal DK, Tiwari R, Garg R, Shrivastava AK, Sarawagi R, Patil R, Agarwal L, Gupta P, Lahariya C. Impact of Janani Suraksha Yojana on institutional delivery rate and maternal morbidity and mortality: an observational study in India. J Health Popul Nutr. 2012; 30(4): 464-71.
Gupta V, Dawood FS, Rai SK, Broor S, Wigh R, Mishra AC, Lafond K, Mott JA, Widdowson M-A, Lal RB, Krishnan A. Validity of clinical case definitions for influenza surveillance among hospitalized patients: results from a rural community in North India. Influenza Other Respir Viruses. 2013; 7(3): 321–9.
Gupta V, Kaur J, Chander J. An increase in enteric fever cases due to Salmonella Paratyphi A in and around Chandigarh. Indian J Med Res. 2009; 129(1): 95-8.
Gupta V, Shukla K. Epidemiology of anaemia in pre-school children from a rural and a slum community of Varanasi. Indian J Prev Soc Med. 1985; 85-9.
Gupte SC, Patel PU, Ranat JM. G6PD deficiency in Vataliya Prajapati community settled in Surat. Indian J Med Sci. 2005; 59(2): 51-6.
Gupte SC, Shaw AN, Shah KC. Hematological findings and severity of G6PD deficiency in Vataliya Prajapati subjects. J Assoc Physicians India. 2005; 53: 1027–30.
Gurukartick J, Dongre AR, Shah D. Social Determinants of Dementia and Caregivers' Perspectives in the Field Practice Villages of Rural Health Training Centre, Thiruvennainallur. Indian J Palliat Care. 2016; 22(1): 25-32.
Guruprasad BS, Krishnamurthy D, Narendra DP, Ranganath BG, Shamanna RB. Changing scenario of cataract blindness in Kolar District, Karnataka, South India. The utility of rapid assessment of avoidable blindness in reviewing programs. Ophthalmic Epidemiol. 2013; 20(2): 89-95.
Gyan C, Rs T. Parasitological Observations on Prevalence, Seasonality and Transmission of Malaria among Tribals of Surguja District. Indian J Community Med. 2001; 26(4): 183–3.
Hackett R, Hackett, Latha, Bhakta, Preeta, Gowers, Simon. The Prevalence and Associations of Psychiatric Disorder in Children in Kerala, South India. J Child Psychol Psychiatry. 1999; 40(5): 801-7.
Hackett RJ, Hackett L, Bhakta P. The prevalence and associated factors of epilepsy in children in Calicut District, Kerala, India. Acta Paediatr. 1997; 86(11): 1257-60.
Hara HS, Gupta A, Singh M, Raj R, Singh H, Pawar G, Hara PK, Singh J. Epilepsy in Punjab (India): A Population-Based Epidemiologic Study. Neuroepidemiology. 2015; 45(4): 273-81.
Haricharan KR, Shrinivasa BM, Kumari V. Clinical and bacteriological study of acute diarrhoea in children. Journal of Evolution of Medical and Dental Sciences. 2013; 2(23): 4229–4238.
Harikrishnan S, Sanjay G, Anees T, Viswanathan S, Vijayaraghavan G, Bahuleyan CG, et al. Clinical presentation, management, in-hospital and 90-day outcomes of heart failure patients in Trivandrum, Kerala, India: the Trivandrum Heart Failure Registry. Eur J Heart Fail. 2015; 17(8):794–800.
Harrison G, Hopper K, Craig T, Laska E, Siegel C, Wanderling J, Dube KC, Ganev K, Giel R, an der Heiden W, Holmberg SK, Janca A, Lee PW, León CA, Malhotra S, Marsella AJ, Nakane Y, Sartorius N, Shen Y, Skoda C, Thara R, Tsirkin SJ, Varma VK, Walsh D, Wiersma D. Recovery from psychotic illness: a 15- and 25-year international follow-up study. Br J Psychiatry. 2001; 178: 506-17.
Hashibe M, Sankaranarayanan R, Thomas G, Kuruvilla B, Mathew B, Somanathan T, Parkin DM, Zhang ZF. Alcohol drinking, body mass index and the risk of oral leukoplakia in an Indian population. Int J Cancer. 2000; 88(1): 129-34.
Hasker E, Singh SP, Malaviya P, Picado A, Gidwani K, Singh RP, Menten J, Boelaert M, Sundar S. Visceral leishmaniasis in rural Bihar, India. Emerg Infect Dis. 2012; 18(10): 1662-4.

Table 3: GBD 2016 India data inputs
Hassan F, Sadowski LS, Bangdiwala SI, Vizcarra B, Ramiro L, De Paula CS, Bordin IA, Mitra MK. Physical intimate partner violence in Chile, Egypt, India and the Philippines. Inj Control Saf Promot. 2004; 11(2): 111-6.
Haswell-Elkins MR, Elkins DB, Manjula K, Michael E, Anderson RM. An investigation of hookworm infection and reinfection following mass anthelmintic treatment in the south Indian fishing community of Vairavankuppam. Parasitology. 1988; 96: 565–77.
Havaldar PV. Diphtheria in the eighties: experience in a south Indian district hospital. J Indian Med Assoc. 1992; 90(6): 155-6.
Healis-Sekhsaria Institute for Public Health, Madhya Pradesh Voluntary Health Association, Cancer Foundation of India, School of Preventive Oncology, University of Waterloo. International Tobacco Control Policy Evaluation Project: India Tobacco Control Survey 2010-2011. Navi Mumbai, India and Waterloo, Canada: Healis-Sekhsaria Institute for Public Health and University of Waterloo.
Health Department - Chandigarh Administration, Postgraduate Institute of Medical Education & Research (PGIMER) Chandigarh, Tata Memorial Center. Cancer Incidence and Mortality in Chandigarh Union Territory 2013. Chandigarh, India: PGIMER Chandigarh.
Health Management Information System (HMIS), National Health Mission, Ministry of Health and Family Welfare, Government of India. India HMIS Standard Report - Data Itemwise Monthly 2009-2010. New Delhi, India: Ministry of Health and Family Welfare.
Health Management Information System (HMIS), National Health Mission, Ministry of Health and Family Welfare, Government of India. India HMIS Standard Report - Data Itemwise Monthly 2010-2011. New Delhi, India: Ministry of Health and Family Welfare.
Health Management Information System (HMIS), National Health Mission, Ministry of Health and Family Welfare, Government of India. India HMIS Standard Report - Data Itemwise Monthly 2011-2012. New Delhi, India: Ministry of Health and Family Welfare.
Health Management Information System (HMIS), National Health Mission, Ministry of Health and Family Welfare, Government of India. India HMIS Standard Report - Data Itemwise Monthly 2013-2014. New Delhi, India: Ministry of Health and Family Welfare.
Health Management Information System (HMIS), National Health Mission, Ministry of Health and Family Welfare, Government of India. India Performance Related to Immunisation 2007-2009. New Delhi, India: Ministry of Health and Family Welfare.
Health Management Information System (HMIS), National Health Mission, Ministry of Health and Family Welfare, Government of India. India Performance Related to Immunisation 2008-2010. New Delhi, India: Ministry of Health and Family Welfare.
Health Management Information System (HMIS), National Health Mission, Ministry of Health and Family Welfare, Government of India. India Performance Related to Immunisation 2009-2011. New Delhi, India: Ministry of Health and Family Welfare.
Health Management Information System (HMIS), National Health Mission, Ministry of Health and Family Welfare, Government of India. India Performance Related to Immunisation 2010-2012. New Delhi, India: Ministry of Health and Family Welfare.
Health Management Information System (HMIS), National Health Mission, Ministry of Health and Family Welfare, Government of India. India Performance Related to Immunisation 2011-2013. New Delhi, India: Ministry of Health and Family Welfare.
Health Management Information System (HMIS), National Health Mission, Ministry of Health and Family Welfare, Government of India. India Performance Related to Immunisation 2012-2014. New Delhi, India: Ministry of Health and Family Welfare.

Table 3: GBD 2016 India data inputs
Health Management Information System (HMIS), National Health Mission, Ministry of Health and Family Welfare, Government of India. India Performance Related to Immunisation 2013-2015. New Delhi, India: Ministry of Health and Family Welfare.
Health Management Information System (HMIS), National Health Mission, Ministry of Health and Family Welfare, Government of India. India Performance Related to Immunisation 2014-2016. New Delhi, India: Ministry of Health and Family Welfare.
Health Management Information System (HMIS), National Health Mission, Ministry of Health and Family Welfare, Government of India. India Performance Related to Immunisation 2015-2017. New Delhi, India: Ministry of Health and Family Welfare.
Health Management Information System (HMIS), National Health Mission, Ministry of Health and Family Welfare, Government of India.India HMIS Standard Report - Data Itemwise Monthly 2012-2013. New Delhi, India: Ministry of Health and Family Welfare.
Health Management Information System (HMIS), National Health Mission, Ministry of Health and Family Welfare, Government of India.India HMIS Standard Report - Data Itemwise Monthly 2014-2015. New Delhi, India: Ministry of Health and Family Welfare.
Health Management Information System (HMIS), National Health Mission, Ministry of Health and Family Welfare, Government of India.India HMIS Standard Report - Data Itemwise Monthly 2015-2016. New Delhi, India: Ministry of Health and Family Welfare.
Hebert JR, Gupta PC, Bhonsle RB, Sinor PN, Mehta H, Mehta FS. Development and testing of a quantitative food frequency questionnaire for use in Gujarat, India. Public Health Nutr. 1999; 2(1): 39–50.
Hegde A, Ballal M, Shenoy S. Detection of diarrheagenic Escherichia coli by multiplex PCR. Indian J Med Microbiol. 2012; 30(3): 279-84.
Hemalatha R, Swetha GK, Seshacharyulu M, Radhakrishna KV. Respiratory syncitial virus in children with acute respiratory infections. Indian J Pediatr. 2010; 77(7): 755-8.
Hirve S, Ganatra B. A prospective cohort study on the survival experience of under five children in rural western India. Indian Pediatr. 1997; 34(11): 995-1001.
Hirve S, Singh SP, Kumar N, Banjara MR, Das P, Sundar S, Rijal S, Joshi A, Kroeger A, Varghese B, Thakur CP, Huda MM, Mondal D. Effectiveness and feasibility of active and passive case detection in the visceral leishmaniasis elimination initiative in India, Bangladesh, and Nepal. Am J Trop Med Hyg. 2010; 83(3): 507-11.
Hochberg CH, Schneider JA, Dandona R, Lakshmi V, Kumar GA, Sudha T, et al. Population and dyadic-based seroincidence of herpes simplex virus-2 and syphilis in southern India. Sexually Transmitted Infections. 2015 Aug;91(5):375–82.
Huffman MD, Prabhakaran D, Osmond C, Fall CHD, Tandon N, Lakshmy R, Ramji S, Khalil A, Gera T, Prabhakaran P, Biswas SKD, Reddy KS, Bhargava SK, Sachdev HS, New Delhi Birth Cohort. Incidence of cardiovascular risk factors in an Indian urban cohort results from the New Delhi birth cohort. J Am Coll Cardiol. 2011; 57(17): 1765-74.
Huilan S, Zhen LG, Mathan MM, Mathew MM, Olarte J, Espejo R, Khin Maung U, Ghafoor MA, Khan MA, Sami Z. Etiology of acute diarrhoea among children in developing countries: a multicentre study in five countries. Bull World Health Organ. 1991; 69(5): 549-55.
Hunt GM, Oakeshott P. Outcome in people with open spina bifida at age 35: prospective community based cohort study. BMJ. 2003; 326(7403): 1365-6.

Table 3: GBD 2016 India data inputs
Hussain T, Kulshreshtha KK, Sinha S, Yadav VS, Katoch VM. HIV, HBV, HCV, and syphilis co-infections among patients attending the STD clinics of district hospitals in Northern India. Int J Infect Dis. 2006; 10(5): 358-63.
Hussain, T, Kulshreshtha, KK, Yadav VS, Sengupta U, Katoch VM. Seroprevalence of Human Immunodeficiency Virus and Syphilis Infections among Patients Attending the STD, Gyn. and Obs., ANC Clinics of Northern India. Am J Infect Dis. 2006; 2(1): 28–35.
Hussein J, Ramani KV, Kanguru L, Patel K, Bell J, Patel P, Walker L, Mehta R, Mavalankar D. The effect of surveillance and appreciative inquiry on puerperal infections: a longitudinal cohort study in India. PLoS One. 2014; 9(1): e87378.
Hyma B, Ramesh A, Gunasekaran K. Lymphatic filariasis in Madras, India. Soc Sci Med. 1989; 29(8): 983-90.
Imdad A, Herzer K, Mayo-Wilson E, Yakoob MY, Bhutta ZA. Vitamin A supplementation for preventing morbidity and mortality in children from 6 months to 5 years of age. Cochrane Database Syst Rev. 2010; CD008524.
INDEPTH Network. Africa, Asia, Oceania - INDEPTH Network Cause-Specific Mortality - Release 2014. Accra, Ghana: INDEPTH Network; 2014.
India Lead Exposure Data 1991 from literature review, as provided by the Global Burden of Disease 2010 Lead Exposure Expert Group.
India Neonatal Preterm Birth Complications Data 1995 as provided by the Global Burden of Disease 2010 Neonatal Conditions Expert Group. [Data shared for this analysis]
India Plasmodium Falciparum Parasite Rate Data, Personal Communication with S.K. Sharma 2006.
India Plasmodium Falciparum Parasite Rate Data, Personal Communication with S.K. Sharma 2008.
Indian Council of Medical Research (ICMR), Madras Diabetes Research Foundation. Indian Council of Medical Research India Diabetes Study (ICMR-INDIAB) - North East 2012-2015. [Data shared for this analysis]
Indian Council of Medical Research (ICMR), Madras Diabetes Research Foundation. Indian Council of Medical Research India Diabetes Study (ICMR-INDIAB) 2008-2010. [Data shared for this analysis]
Indian Council of Medical Research (ICMR), Madras Diabetes Research Foundation. Indian Council of Medical Research India Diabetes Study (ICMR-INDIAB) 2012-2013. [Data shared for this analysis]
Indian Council of Medical Research (ICMR). India Jai Vigyan Mission Mode Project: Community Control of Rheumatic Fever/Rheumatic Heart Disease in India Comprehensive Project Report 2001-2010. New Delhi, India: ICMR.
Indian Council of Medical Research (ICMR). India Study on Causes of Death by Verbal Autopsy 2003. New Delhi, India: ICMR. [Data shared for this analysis]
Indian Council of Medical Research, National Institute of Medical Statistics, National JALMA Institute for Leprosy and Other Mycobacterial Diseases. India National Sample Survey to Assess Disease Burden in Leprosy 2010-2011. [Data shared for this analysis]
Indian Council of Medical Research, World Health Organization. India STEPS Noncommunicable Disease Risk Factors Survey 2003-2005. [Data shared for this analysis]
Indian Institute of Health Management Research, International Institute of Population Sciences, Ministry of Health and Family Welfare, Government of India, Performance Monitoring and Accountability 2020 (PMA2020) Project, Bill & Melinda Gates Institute for Population and Reproductive Health, Johns Hopkins Bloomberg School of Public Health. PMA 2016 Rajasthan Round 1 Survey Data as part of PMA2020 Project. Baltimore, United States: PMA2020.
Indian Society of Nephrology. Indian Society for Nephrology Chronic Kidney Disease Registry 2008-2013. [Data shared for this analysis]

Table 3: GBD 2016 India data inputs
Institute of Health Systems, World Health Organization (WHO). WHO Multi-country Survey Study Report on Health and Health System Responsiveness, Andhra Pradesh 2000-2001.
Institute of Health Systems. India Cause of Death Dataset Version 1.3 1980-1998. Hyderabad, India: Institute of Health Systems; 2002.
International Clearinghouse for Birth Defects Surveillance and Research. Annual Report 2010. Rome, Italy: International Clearinghouse for Birth Defects Surveillance and Research; 2011.
International Clearinghouse for Birth Defects Surveillance and Research. Annual Report 2011. Rome, Italy: International Clearinghouse for Birth Defects Surveillance and Research; 2012.
International Clearinghouse for Birth Defects Surveillance and Research. Annual Report 2012. Rome, Italy: International Clearinghouse for Birth Defects Surveillance and Research; 2013.
International Clinical Epidemiology Network (INCLEN). INCLEN Verbal Autopsy Data 2005-2006. [Data shared for this analysis]
International Institute for Population Sciences (IIPS), Ministry of Health and Family Welfare, Government of India, Harvard T. H. Chan School of Public Health, RAND Corporation, Monash University, University of California Los Angeles, Indian Academy of Geriatrics, National AIDS Research Institute, Columbia University. Longitudinal Aging Study in India, Pilot Data 2010. Mumbai, India: IIPS.
International Institute for Population Sciences (IIPS), Ministry of Health and Family Welfare, Government of India, ICF. India National Family Health Survey (NFHS-4) 2015-2016: National and State-level Factsheets. Mumbai, India: IIPS.
International Institute for Population Sciences (IIPS), Ministry of Health and Family Welfare, Government of India, Macro International. India National Family Health Survey (NFHS-1) 1992-1993: National Report. Mumbai, India: IIPS.
International Institute for Population Sciences (IIPS), Ministry of Health and Family Welfare, Government of India, Macro International. India National Family Health Survey (NFHS-3) 2005-2006: National and State Reports. Mumbai, India: IIPS.
International Institute for Population Sciences (IIPS), Ministry of Health and Family Welfare, Government of India, Macro International. India National Family Health Survey Data (NFHS-1) 1992-1993. Mumbai, India: IIPS.
International Institute for Population Sciences (IIPS), Ministry of Health and Family Welfare, Government of India, Macro International. India National Family Health Survey Data (NFHS-3) 2005-2006. Mumbai, India: IIPS.
International Institute for Population Sciences (IIPS), Ministry of Health and Family Welfare, Government of India, ORC Macro. India National Family Health Survey (NFHS-2) 1998-1999: National and State Reports. Mumbai, India: IIPS.
International Institute for Population Sciences (IIPS), Ministry of Health and Family Welfare, Government of India, ORC Macro. India National Family Health Survey Data (NFHS-2) 1998-1999. Mumbai, India: IIPS.
International Institute for Population Sciences (IIPS), Ministry of Health and Family Welfare, Government of India. India District Level Household and Facility Survey (DLHS-3) 2007-2008: National and State Reports. Mumbai, India: IIPS.
International Institute for Population Sciences (IIPS), Ministry of Health and Family Welfare, Government of India. India District Level Household and Facility Survey Data (DLHS-3) 2007-2008. Mumbai, India: IIPS.
International Institute for Population Sciences (IIPS), Ministry of Health and Family Welfare, Government of India. India District Level Household and Facility Survey Data (DLHS-4) 2012-2013: State Reports. Mumbai, India: IIPS.
International Institute for Population Sciences (IIPS), Ministry of Health and Family Welfare, Government of India. India District Level Household Survey (DLHS-1) 1998-1999: National Report. Mumbai, India: IIPS.
International Institute for Population Sciences (IIPS), Ministry of Health and Family Welfare, Government of India. India District Level Household Survey (DLHS-2) 2002-2004: National and State Reports. Mumbai, India: IIPS.
International Institute for Population Sciences (IIPS), Ministry of Health and Family Welfare, Government of India. India District Level Household Survey Data (DLHS-1) 1998-1999. Mumbai, India: IIPS.

Table 3: GBD 2016 India data inputs
International Institute for Population Sciences (IIPS), Ministry of Health and Family Welfare, Government of India. India District Level Household Survey Data (DLHS-2) 2002-2004. Mumbai, India: IIPS.
International Institute for Population Sciences, Ministry of Health and Family Welfare, Government of India. India District Level Household and Facility Survey Data (DLHS-4) 2012-2013. Mumbai, India: IIPS.
International Institute for Population Sciences, Population Council, Ministry of Health and Family Welfare, Government of India. Youth in India: Situation and Needs Study 2006–2007. Mumbai, India: IIPS.
International Institute for Population Sciences, World Health Organization (WHO). India WHO Study on Global Ageing and Adult Health 2007-2008.
International Institute for Population Sciences, World Health Organization (WHO). India WHO Study on Global Ageing and Adult Health 2014-2015.
International Institute for Population Sciences, World Health Organization. India World Health Survey 2003.
International Institute for Strategic Studies. Armed Conflict Database. London, United Kingdom: International Institute for Strategic Studies.
International Labour Organization (ILO). International Labour Statistics Database (ILOSTAT) - Employment by Sex and Economic Activity. Geneva, Switzerland: ILO.
International Labour Organization (ILO). International Labour Statistics Database (ILOSTAT) - Employment to Population Ratio. Geneva, Switzerland: ILO.
International Labour Organization (ILO). International Labour Statistics Database (ILOSTAT) - Fatal Injuries by Sex and Economic Activity. Geneva, Switzerland: ILO.
International Road Federation. World Road Statistics 1963-1999. Geneva, Switzerland: International Road Federation.
International Road Federation. World Road Statistics 2015. Geneva, Switzerland: International Road Federation; 2015.
International Road Federation. World Road Statistics 2016. Geneva, Switzerland: International Road Federation; 2016.
International Road Federation. World Road Statistics Database 2007. Geneva, Switzerland: International Road Federation.
International Road Federation. World Road Statistics Database 2009. Geneva, Switzerland: International Road Federation.
International Social Survey Programme (ISSP), GESIS - Leibniz Institute of Social Sciences. ISSP: Family and Changing Gender Roles IV, ZA5900 (Version 4.0.0). Mannheim, Germany: GESIS - Leibniz Institute of Social Sciences; 2012.
Intersalt Cooperative Research Group. Intersalt: an international study of electrolyte excretion and blood pressure. Results for 24 hour urinary sodium and potassium excretion. BMJ. 1988;297(6644): 319-28.
Isharwal S, Arya S, Misra A, Wasir JS, Pandey RM, Rastogi K, Vikram NK, Luthra K, Sharma R. Dietary nutrients and insulin resistance in urban Asian Indian adolescents and young adults. Ann Nutr Metab. 2008; 52(2): 145–51.
Iyengar K, Iyengar SD, Suhalka V, Dashora K. Pregnancy-related deaths in rural Rajasthan, India: exploring causes, context, and care-seeking through verbal autopsy. J Health Popul Nutr. 2009; 27(2): 293-302.
Iyer S, Sengupta C, Velumani A. Lead toxicity: An overview of prevalence in Indians. Clin Chim Acta. 2015; 451(Pt B): 161-4.
Iyer V, Azhar GS, Choudhury N, Dhruwey VS, Dacombe R, Upadhyay A. Infectious disease burden in Gujarat (2005-2011): comparison of selected infectious disease rates with India. Emerg Health Threats J. 2014; 19(7): 22838.
Jacqueline L. The high burden of cholera in children: comparison of incidence from endemic areas in Asia and Africa. PLoS Negl Trop Dis. 2008; 2(2): e173.
Jain A, Gupta SC, Misra SK, Singh R, Bhagoliwal AK, Kaushal SK. Trend and causes of maternal mortality among women delivering in S. N. Medical College Hospital, Agra. Indian J Public Health. 2009; 53(1): 47-8.
Jain A, Vinod Bhat H, Acharya D. Prevalence of bronchial asthma in rural Indian children: a cross sectional study from South India. Indian J Pediatr. 2010; 77(1): 31-5.

Table 3: GBD 2016 India data inputs
Jain D, Sanon S, Sadowski L, Hunter W. Violence against women in India: evidence from rural Maharashtra, India. Rural Remote Health. 2004; 4(4): 304.
Jain D, Sinha S, Prasad KN, Pandey CM. Campylobacter species and drug resistance in a north Indian rural community. Trans R Soc Trop Med Hyg. 2005; 99(3): 207–14.
Jain DC, Jain RK, Ichhpujani RL, Sharma RS. Prevalence of hepatitis B virus in pregnant women. J Commun Dis. 1994; 26(4): 233-4.
Jain DL, Sarathi V, Upadhye D, Gulhane R, Nadkarni AH, Ghosh K, Colah RB. Newborn screening shows a high incidence of sickle cell anemia in Central India. Hemoglobin. 2012; 36(4): 316–22.
Jain RC, Soni SB. Detection of HBsAg and HIV carriage among blood donors or rural population of Loni areas. J Assoc Physicians India. 1995; 43(5): 378.
Jain RC. G-6PD deficiency in malaria endemic areas of Udaipur District in Rajasthan. J Assoc Physicians India. 1992; 40(10): 662–3.
Jain S, Chopra H, Garg SK, Bhatnagar M, Singh JV. Anemia in children: early iron supplementation. Indian J Pediatr. 2000; 67(1): 19-21.
Jain Y, Bansal M, Tiwari R, Kasar PK. Causes of Neonatal Mortality: A community based study using verbal autopsy tool. Natl J Community Med. 2013; 4(3): 499-502.
Jajoo UN, Chhabra S, Gupta OP, Jain AP. Measles epidemic in a rural community near Sevagram (Vidarbha). Indian J Public Health. 1984; 28(4): 204-7.
Jambulingam P, Mohapatra SS, Govardhini P, Das LK, Manoharan A, Pani SP, Das PK. Microlevel epidemiological variations in malaria & its implications on control strategy. Indian J Med Res. 1991; 371-8.
Jashnani KD, Rupani AB, Wani RJ. Maternal mortality: an autopsy audit. J Postgrad Med. 2009; 55(1): 12-6.
Jaya J, Hindin MJ. Nonconsensual Sexual Experiences of Adolescents in Urban India. J Adolesc Health. 2007; 40(6): 573e7–573e.
Jayalekshmi PA, Gangadharan P, Akiba S, Koriyama C, Nair RR. Oral cavity cancer risk in relation to tobacco chewing and bidi smoking among men in Karunagappally, Kerala, India: Karunagappally cohort study. Cancer Sci. 2011; 102(2.0): 460-7.
Jayalekshmi PA, Gangadharan P, Akiba S, Nair RR, Tsuji M, Rajan B. Tobacco chewing and female oral cavity cancer risk in Karunagappally cohort, India. Br J Cancer. 2009; 100(5.0): 848-52.
Jayaprakash P, Bhansali S, Bhansali A, Dutta P, Anantharaman R. Magnitude of foot problems in diabetes in the developing world: a study of 1044 patients. Diabet Med. 2009; 26(9): 939-42.
Jennings JM, Louis TA, Ellen JM, Srikrishnan AK, Sivaram S, Mayer K, Solomon S, Kelly R, Celentano DD. Geographic prevalence and multilevel determination of community-level factors associated with herpes simplex virus type 2 infection in Chennai, India. Am J Epidemiol. 2008; 167(12): 1495–503.
Jet Propulsion Laboratory, National Aeronautics and Space Administration. Aerosol Optical Depth data from Multi-angle Imaging Spectro Radiometer. Available from: https://www-misr.jpl.nasa.gov/getData/accessData/
Jindal SK, Aggarwal AN, Gupta D, Agarwal R, Kumar R, Kaur T, Chaudhry K, Shah B. Indian study on epidemiology of asthma, respiratory symptoms and chronic bronchitis in adults (INSEARCH). Int J Tuberc Lung Dis. 2012; 16(9): 1270-7.
Jog S, Soman R, Singhal T, Rodrigues C, Mehta A, Dastur FD. Enteric fever in Mumbai – clinical profile, sensitivity patterns and response to antimicrobials. J Assoc Physicians India. 2008; 56: 237-40.

Table 3: GBD 2016 India data inputs
John J, Van Aart CJ, Grassly NC. The burden of typhoid and paratyphoid in India: systematic review and meta-analysis. PLoS Negl Trop Dis. 2016;10(4):e0004616.
John S, Sanghi S, Prasad S, Bose A, George K. Two doses of measles vaccine: are some states in India ready for it?. J Trop Pediatr. 2009; 55(4): 253-6.
John TJ, Cherian T, Steinhoff MC, Simoes EA, John M. Etiology of acute respiratory infections in children in tropical southern India. Rev Infect Dis. 1991; 13 Suppl 6: S463-9.
John TJ, Joseph A, George T, Radhakrishnan J, Singh RPD, George K. Epidemiology and prevention of measles in rural south India. Indian J Med Res. 1980; 72: 153-8.
Johnson C, Mohan S, Rogers K, Shivashankar R, Thout SR, Gupta P, He FJ, MacGregor GA, Webster J, Krishnan A, Maulik PK, Reddy KS, Prabhakaran D, Neal B. Mean Dietary Salt Intake in Urban and Rural Areas in India: A Population Survey of 1395 Persons. J Am Heart Assoc. 2017; 6(1):e004547.
Johnson C, Praveen D, Pope A, Raj TS, Pillai RN, Land MA, Neal B. Mean population salt consumption in India: a systematic review. J Hypertens. 2017; 35(1): 3-9.
Johnson P, Balakrishnan K, Ramaswamy P, Ghosh S, Sadhasivam M, Abirami O, et al. Prevalence of chronic obstructive pulmonary disease in rural women of Tamilnadu: implications for refining disease burden assessments attributable to household biomass combustion. Glob Health Action. 2011;4(1):7226.
Joint Research Centre (JRC), European Commission, Center for International Earth Science Information Network, Columbia University. GHS population grid, derived from GPW4, multitemporal (1975, 1990, 2000, 2015). Brussels, Belgium: JRC, European Commission; 2015. Available from: http://data.europa.eu/89h/jrc-ghsl-ghs_pop_gpw4_globe_r2015a
Joint United Nations Program on HIV/AIDS (UNAIDS), United Nations Children's Fund, World Health Organization. India Global AIDS Response Progress Reporting System - Antenatal Care Attendees Positive for Syphilis. Geneva, Switzerland: UNAIDS.
Joint United Nations Program on HIV/AIDS (UNAIDS). UNAIDS Spectrum - National HIV Estimates 2016. Geneva, Switzerland: UNAIDS.
Jose VJ, Gomathi M. Declining prevalence of rheumatic heart disease in rural schoolchildren in India: 2001-2002. Indian Heart J. 2003; 55(2): 158-60.
Joseph N, Kumar GS, Nelliyanil M. Pattern of seizure cases in tertiary care hospitals in Karnataka state of India. Ann Indian Acad Neurol. 2013; 16(3): 347-51.
Joshi A, Shah D, Panchabhai T, Patil P. An autopsy study of maternal mortality: A tertiary healthcare perspective. J Postgrad Med. 2009; 55(1): 8.
Joshi DC, Mishra VN, Bhatnagar M, Singh RB, Garg SK, Chopra H. Socioeconomic factors and prevalence of endemic goitre. Indian J Public Health. 1993; 37(2): 48-53.
Joshi H, Subbarao SK, Valecha N, Sharma VP. Ahaptoglobinemia (HpO) and malaria in India. Indian J Malariol. 2002; 39(1-2): 1-12.
Joshi K, Kumar R, Avasthi A. Morbidity profile and its relationship with disability and psychological distress among elderly people in Northern India. Int J Epidemiol. 2003; 32(6): 978-87.
Joshi N, Rajesh R, Sunitha M. Prevalence of dental caries among school children in Kulasekharam village: a correlated prevalence survey. J Indian Soc Pedod Prev Dent. 2005; 23(3): 138-40.
Joshi N, Sujan S, Joshi K, Parekh H, Dave B. Prevalence, severity and related factors of dental caries in school going children of Vadodara City – an epidemiological study. J Int Oral Health. 2013; 5(4): 35-9.
Joshi PP, Mohanan CJ, Sengupta SP, Salkar RG. Factors precipitating congestive heart failure – role of patient non-compliance. J Assoc Physicians India. 1999; 47(3): 294-5.

Table 3: GBD 2016 India data inputs
Joshi R, Cardona M, Iyengar S, Sukumar A, Raju CR, Raju KR, Raju K, Reddy KS, Lopez A, Neal B. Chronic diseases now a leading cause of death in rural India – mortality data from the Andhra Pradesh Rural Health Initiative. Int J Epidemiol. 2006; 35(6): 1522-9.
Joshi R, Guggilla R, Praveen D, Maulik PK. Suicide deaths in rural Andhra Pradesh--a cause for global health action. Trop Med Int Health. 2015; 20(2): 188–93.
Joshi R, Narang U, Zwerling A, Jain D, Jain V, Kalantri S, Pai M. Predictive value of latent tuberculosis tests in Indian healthcare workers: a cohort study. Eur Respir J. 2011; 38(6): 1475–7.
Joshi SR, Patel RZ, Patel HR, Sukumar S, Colah RB. High prevalence of G6PD deficiency in Vataliya Prajapati community in western India. Haematologia (Budap). 2001; 31(1): 57-60.
Joshi VL, Chopra A. Is there an urban-rural divide? Population surveys of rheumatic musculoskeletal disorders in the Pune region of India using the COPCORD Bhigwan model. J Rheumatol. 2009; 36(3): 614-22.
Joyee AG, Thyagarajan SP, Rajendran P, Hari R, Balakrishnan P, Jeyaseelan L, Kurien T, STD Study Group. Chlamydia trachomatis genital infection in apparently healthy adult population of Tamil Nadu, India: a population-based study. Int J STD AIDS. 2004; 15(1): 51-5.
JSS Medical College and Hospital-Mysore, Population Health and Occupational Disease, National Heart & Lung Institute, Imperial College London. India - Mysore Burden of Obstructive Lung Disease Initiative Survey (BOLD) 2011-2014. [Data shared for this analysis]
Jussawalla DJ, Deshpande VA. Evaluation of cancer risk in tobacco chewers and smokers: an epidemiologic assessment. Cancer. 1971; 28(1): 244-52.
KA Suni, Ali A, Lal AA, Kailas L. Clinico-immunological Profile of Juvenile Idiopathic Arthritis in Children Attending SAT Hospital, Thiruvananthapuram. Acad Med J India. 2015; 3(3): 99-105.
Kakkar N, Kaur R, Dhanoa J. Voluntary donors-need for a second look. Indian J Pathol Microbiol. 2004; 47(3): 381-3.
Kalasapati L, Ivaturi S, Reddy P, Babu S. Incidence of suicides in three villages of Khammam district of South India. AP J Psychol Med. 2014; 15(1): 103-7.
Kaliappan SP, George S, Francis MR, Kattula D, Sarkar R, Minz S, Mohan VR, George K, Roy S, Ajjampur SSR, Muliyil J, Kang G. Prevalence and clustering of soil-transmitted helminth infections in a tribal area in southern India. Trop Med Int Health. 2013; 18(12): 1452–62.
Kalikivayi V, Naduvilath TJ, Bansal AK, Dandona L. Visual impairment in school children in southern India. Indian J Ophthalmol. 1997; 45(2): 129-34.
Kalimuthu M, Sunish IP, Nagaraj J, Munirathinam A, Kumar VA, Arunachalam N, White GB, Tyagi BK. Residual microfilaraemia in rural pockets of South India after five rounds of DEC plus albendazole administration as part of the LF elimination campaign. J Vector Borne Dis. 2015; 52(2): 182-4.
Kalkonde YV, Sahane V, Deshmukh MD, Nila S, Mandava P, Bang A. High Prevalence of Stroke in Rural Gadchiroli, India: A Community-Based Study. Neuroepidemiology. 2016; 46(4): 235-9.
Kalra V, Chitralekha KT, Dua T, Pandey RM, Gupta Y. Blood lead levels and risk factors for lead toxicity in children from schools and an urban slum in Delhi. J Trop Pediatr. 2003; 49(2): 121-3.
Kalra V, Sahu JK, Bedi P, Pandey RM. Blood lead levels among school children after phasing-out of leaded petrol in Delhi, India. Indian J Pediatr. 2013; 80(8): 636-40.
Kamble M, Chatruvedi P. Epidemiology of sickle cell disease in a rural hospital of central India. Indian Pediatr. 2000; 37(4): 391–6.

Table 3: GBD 2016 India data inputs
Kamble P, Deshmukh PR, Garg N. Metabolic syndrome in adult population of rural Wardha, central India. Indian J Med Res. 2010; 132: 701–5.
Kameswaran C, Bhatia BD, Bhat BV, Oumachigui A. Perinatal mortality: a hospital based study. Indian Pediatr. 1993; 30(8): 997-1001.
Kang G, Arora R, Chitambar SD, Deshpande J, Gupte MD, Kulkarni M, Naik TN, Mukherji D, Venkatasubramaniam S, Gentsch JR, Glass RI, Parashar UD. Multicenter, hospital-based surveillance of rotavirus disease and strains among Indian children aged <5 years. J Infect Dis. 2009; 200(Suppl 1): 147-153.
Kang G, Desai R, Arora R, Chitamabar S, Naik TN, Krishnan T, Deshpande J, Gupte MD, Venkatasubramaniam S, Gentsch JR, Parashar UD, Indian Rotavirus Strain Surveillance Network, Mathew A, Anita null, Ramani S, Sowmynarayanan TV, Moses PD, Agarwal I, Simon A, Bose A, Arora R, Chhabra P, Fadnis P, Bhatt J, Shetty SJ, Saxena VK, Mathur M, Jadhav A, Roy S, Mukherjee A, Singh NB. Diversity of circulating rotavirus strains in children hospitalized with diarrhea in India, 2005-2009. Vaccine. 2013; 31(27): 2879–83.
Kansal S, Mehra R, Singh NP. Life time fatality risk assessment due to variation of indoor radon concentration in dwellings in western Haryana, India. Appl Radiat Isot. 2012; 70(7): 1110-2.
Kanungo S, Sah B, Lopez A, Sung J, Paisley A, Sur D, Clemens J, Nair GB. Cholera in India: an analysis of reports, 1997–2006. Bull World Health Organ. 2010; 88(3): 185–91.
Kanungo S, Sur D, Ali M, You YA, Pal D, Manna B, Niyogi SK, Sarkar B, Bhattacharya SK, Clemens JD, Nair GB. Clinical, epidemiological, and spatial characteristics of Vibrio parahaemolyticus diarrhea and cholera in the urban slums of Kolkata, India. BMC Public Health. 2012; 12: 830.
Kapil U, Pathak P, Tandon M, Singh C, Pradhan R, Dwivedi SN. Micronutrient deficiency disorders amongst pregnant women in three urban slum communities of Delhi. Indian Pediatr. 1999; 36(10): 983-9.
Kapil U, Saxena N, Ramachandran S, Balamurugan A, Nayar D, Prakash S. Assessment of iodine deficiency disorders using the 30 cluster approach in the National Capital Territory of Delhi. Indian Pediatr. 1996; 33(12): 1013-7.
Kapil U, Sharma TD, Singh P. Iodine status and goiter prevalence after 40 years of salt iodisation in the Kangra District, India. Indian J Pediatr. 2007; 74(2): 135-7.
Kapil U, Singh P, Pathak P, Singh C. Assessment of iodine deficiency disorders in district Bharatpur, Rajasthan. Indian Pediatr. 2003; 40(2): 147-9.
Kapil U, Sohal KS, Sharma TD, Tandon M, Pathak P. Assessment of iodine deficiency disorders using the 30 cluster approach in district Kangra, Himachal Pradesh, India. J Trop Pediatr. 2000; 46(5): 264-6.
Kapil U, Tandon M, Pradhan R, Pathak P. Status of iodine deficiency in selected hill districts of Uttar Pradesh- a pilot study. Indian J Matern Child Health. 1999; 10: 24-7.
Kapoor D, Agarwal KN, Sharma S, Kela K, Kaur I. Iron status of children aged 9-36 months in an urban slum Integrated Child Development Services project in Delhi. Indian Pediatr. 2002; 39(2): 136-44.
Kapoor G, Aneja S. Nutritional disorders in adolescent girls. Indian Pediatr. 1992; 29(8): 969-73.
Kapoor R, Gupta S. Prevalence of congenital heart disease, Kanpur, India. Indian Pediatr. 2008; 45(4): 309-11.
Kapoor RK, Srivastava AK, Misra PK, Sharma B, Thakur S, Srivastava KI, Singh GK. Perinatal mortality in urban slums in Lucknow. Indian Pediatr. 1996; 33(1): 19-23.
Kapur S, Mittal A. Incidence of HIV infection & its predictors in blood donors in Delhi. Indian J Med Res. 1998; 108(8): 45-50.
Kar SK, Mania J, Kar PK. Clinical filarial disease in two ethnic endemic communities of Orissa, India. J Trop Med Hyg. 1993; 96(5): 311-6.

Table 3: GBD 2016 India data inputs
Kar SK, Mania J, Kar PK. Prevalence of lymphatic nodule in a bancroftian endemic population. Acta Tropica. 1993; 55(1-2): 53-60.
Karunakaran U. Assessment of mass drug administration program to eliminate lymphatic filariasis in an urban endemic area of filariasis in North Kerala, India. Int J Infect Dis. 2012; 16:163
Kate SL, Mukherjee BN, Malhotra KC, Phadke MA, Mutalik GS, Sainani GS. Red cell glucose-6-phosphate dehydrogenase deficiency and haemoglobin variants among ten endogamous groups of Maharshtra and West Bengal. Hum Genet. 1978; 44(3): 339-43.
Katoch K, Chauhan DS, Yadav VK, Katoch V, Upadhay P et al. Prevalence Survey of Bacillary Pulmonary Tuberculosis in Western Uttar Pradesh, India. J Infect Pulm Dis. 2015;1(2): 1-7.
Kattula D, Francis MR, Kulinkina A, Sarkar R, Mohan VR, Babji S, Ward HD, Kang G, Balraj V, Naumova EN. Environmental predictors of diarrhoeal infection for rural and urban communities in south India in children and adults. Epidemiol Infect. 2015; 143(14): 3036-47.
Kattula D, Sarkar R, Rao Ajjampur SS, Minz S, Levecke B, Muliyil J, Kang G. Prevalence & risk factors for soil transmitted helminth infection among school children in south India. Indian J Med Res. 2014; 139(1):76–82.
Katyal R, Bansal R, Agrawal V, Goel K, Chaudhary V. Cross-sectional Study to Acknowledge the Independent Association of the Socio-demographic Determinants of Alcohol Use in an Urban Slum of North India. Int J Prev Med. 2014; 5(6): 749–57.
Kaul PS, Kaul B. Erythrocyte protoporphyrin and blood lead levels of children from Jammu and Srinagar and papier mache trainees. Indian J Pediatr. 1986; 53(5): 641-6.
Kaur M, Das GP, Verma IC. Sickle cell trait & disease among tribal communities in Orissa, Madhya Pradesh & Kerala. Indian J Med Res. 1997; 105: 111–6.
Kaur P, Rao SR, Radhakrishnan E, Ramachandran R, Venkatachalam R, Gupte MD. High prevalence of tobacco use, alcohol use and overweight in a rural population in Tamil Nadu, India. J Postgrad Med. 2011; 57(1): 9-15.
Kaur R, Gupta N, Baveja UK. Seroprevalence of HSV1 and HSV2 infections in family planning clinic attenders. J Commun Dis. 2005; 37(4): 307–9.
Kaur S, Sachdev HPS, Dwivedi SN, Lakshmy R, Kapil U. Prevalence of overweight and obesity amongst school children in Delhi, India. Asia Pac J Clin Nutr. 2008; 17(4): 592-6.
Kaushal SS, DasGupta DJ, Prashar BS, Bhardwaj AK. Electrocardiographic manifestations of healthy residents of a tribal Himalayan village. J Assoc Physicians India. 1995; 43(1): 15-6.
Kaushik SL, Parmar VR, Grover N, Kaushik R. Neonatal mortality rate: relationship to birth weight and gestational age. Indian J Pediatr. 1998; 65(3): 429-33.
Kavatkar AN, Sahasrabudhe NS, Jadhav MV, Deshmukh SD. Autopsy study of maternal deaths. Int J Gynaecol Obstet. 2003; 81(1): 1-8.
Kayina KP, Sharma AK, Agrawal K. Implementation of ICD 10: a study on the doctors' knowledge and coding practices in Delhi. Indian J Public Health. 2015; 59(1): 68–9.
Keet IP, Lee FK, van Griensven GJ, Lange JM, Nahmias A, Coutinho RA. Herpes simplex virus type 2 and other genital ulcerative infections as a risk factor for HIV-1 acquisition. Genitourin Med. 1990; 66(5): 330-3.
Kelkar SD, Purohit SG, Boralkar AN, Verma SP. Prevalence of rotavirus diarrhea among outpatients and hospitalized patients: a comparison. Southeast Asian J Trop Med Public Health. 2001; 32(3): 494-9.
KEM Hospital Research Center. India - Pimpale Cardiovascular Risk Factors Study Blood Pressure, Cholesterol, BMI, Blood Glucose, and Diabetes Incidence Measurements 1994-1999. [Data shared for this analysis]

Table 3: GBD 2016 India data inputs
Kessler RC, Birnbaum HG, Shahly V, Bromet E, Hwang I, McLaughlin KA, Sampson N, Andrade LH, de Girolamo G, Demyttenaere K, Haro JM, Karam AN, Kostyuchenko S, Kovess V, Lara C, Levinson D, Matschinger H, Nakane Y, Browne MO, Ormel J, Posada-Villa J, Sagar R, Stein DJ. Age differences in the prevalence and co-morbidity of DSM-IV major depressive episodes: results from the WHO World Mental Health Survey Initiative. Depress Anxiety. 2010; 27(4): 351–64.
Khadilkar AV, Sanwalka NJ, Chiplonkar SA, Khadilkar VV, Mughal MZ. Normative data and percentile curves for Dual Energy X-ray Absorptiometry in healthy Indian girls and boys aged 5-17 years. Bone. 2011; 48(4): 810-9.
Khalil A, Aggarwal R, Thirupuram S, Arora R. Incidence of congenital heart disease among hospital live births in India. Indian Pediatr. 1994; 31(5): 519-27.
Khan AM, Dutta P, Khan SA, Baruah NK, Sarma CK, Mahanta J. Prevalence of bancroftian filariasis in a foot-hill tea garden of upper Assam. J Commun Dis. 1999; 31(2): 145-6.
Khan AM, Dutta P, Khan SA, Baruah NK, Sharma CK, Mahanta J. Bancroftian filariasis in a weaving community of lower Assam. J Commun Dis. 1999; 31(1): 61-2.
Khan AM, Dutta P, Khan SA, Mahanta J. A focus of lymphatic filariasis in a tea garden worker community of central Assam. J Environ Biol. 2004; 25(4): 437–40.
Khan AM, Dutta P, Sarmah CK, Baruah NK, Das S, Pathak AK, Sarmah P, Hussain ME, Mahanta J. Prevalence of lymphatic filariasis in a tea garden worker population of Dibrugarh (Assam), India after six rounds of mass drug administration. J Vector Borne Dis. 2015; 52(4): 314-20.
Khan MM, Kareem MA, Rao GK. Laboratory diagnosis of malaria infection and its natural history in an urban pocket of Hyderabad City. Indian J Malariol. 1989; 26(4): 215-8.
Khandait DW, Ambadekar NN, Zodpey SP, Vasudeo ND. Maternal age as a risk factor for stillbirth. Indian J Public Health. 2000; 44(1): 28-30.
Khandekar RN, Mishra UC, Vohra KG. Environmental lead exposure of an urban Indian population. Sci Total Environ. 1984; 40: 269-78.
Khandekar RN, Raghunath R, Mishra UC. Levels of lead, cadmium, zinc and copper in the blood of an urban population. Sci Total Environ. 1987; 66: 185-91.
Khanna R, Kumar A, Vaghela JF, Sreenivas V, Puliyel JM. Community based retrospective study of sex in infant mortality in India. BMJ. 2003; 327(7407): 126.
Khanna RC, Marmamula S, Krishnaiah S, Giridhar P, Chakrabarti S, Rao GN. Changing trends in the prevalence of blindness and visual impairment in a rural district of India: systematic observations over a decade. Indian J Ophthalmol. 2012; 60(5): 492-7.
Khumanthem PD, Chanam MS, Samjetshabam RD. Maternal mortality and its causes in a tertiary center. J Obstet Gynaecol India. 2012; 62(2): 168-71.
Khurana S, Aggarwal A, Malla N. Comparative analysis of intestinal parasitic infections in slum, rural and urban populations in and around union Territory, Chandigarh. J Commun Dis. 2005; 37(3): 239–43.
Khuroo MS, Mahajan R, Zargar SA, Javid G, Sapru S. Prevalence of biliary tract disease in India: a sonographic study in adult population in Kashmir. Gut. 1989; 30(2): 201-5.
Kim S, Rifkin S, John SM, Jacob KS. Nature, prevalence and risk factors of alcohol use in an urban slum of Southern India. Natl Med J India. 2013; 26(4): 203–9.

Table 3: GBD 2016 India data inputs
Kimuna SR, Djamba YK, Ciciurkaite G, Cherukuri S. Domestic violence in India: insights from the 2005-2006 National Family Health Survey. J Interpers Violence. 2013; 28(4): 773-807.
Kochupillai N,Pandav CS, Godbole MM, Mehta M, Ahuja MMS. Iodine deficiency and neonatal hypothyroidism. Bull World Health Organ. 1986; 64(4): 547-551.
Kodkany BS, Derman RJ, Honnungar NV, Tyagi NK, Goudar SS, Mastiholi SC, Moore JL, McClure EM, Sloan N, Goldenberg RL. Establishment of a Maternal Newborn Health Registry in the Belgaum District of Karnataka, India. Reprod Health. 2015; 12 Suppl 2: S3.
Koen M, Lemson M, Sampathkumar V, Abel R. Prevalence of anaemia among pregnant mothers in a rural South Indian population. J Obstet Gynaecol India. 1992; 42(6): 283-7.
Kokkat AJ, Verma AK. Prevalence of seizures and paralysis in a rural community. J Indian Med Assoc. 1998; 96(2): 43-5.
Kolappan C, Gopi PG, Subramani R, Chadha VK, Kumar P, Venkatesh Prasad V, et al. Estimation of annual risk of tuberculosis infection (ARTI) among children aged 1–9 years in the south zone of India. Int J Tuberc Lung Dis. 2004; 8(4):418–423.
Kolappan C, Subramani R, Chandrasekaran V, Thomas A. Trend in tuberculosis infection prevalence in a rural area in South India after implementation of the DOTS strategy. Int J Tuberc Lung Dis. 2012; 16(10): 1315–9.
Kolappan C, Subramani R, Radhakrishna S, Santha T, Wares F, Baskaran D, Selvakumar N, Narayanan PR. Trends in the prevalence of pulmonary tuberculosis over a period of seven and half years in a ruralcommunity in south India with DOTS. Indian J Tuberc. 2013; 60(3): 168-76.
Kolappan C, Subramani R, Swaminathan S. Tuberculosis mortality in a rural population from South India. Indian J Tuberc. 2016; 63(2): 100–5.
Kolkata International Society of Nephrology (ISN), Kidney Disease Data Center (KDDC). Chronic Kidney Disease and Cardiovascular Risk Survey 2008-2009. Kolkata, India; ISN-KDDC.
Kothari A, Ramachandran VG, Gupta P, Singh B, Talwar V. Seroprevalence of cytomegalovirus among voluntary blood donors in Delhi, India. J Health Popul Nutr. 2002; 20(4): 348-51.
Kotloff KL, Nataro JP, Blackwelder WC, Nasrin D, Farag TH, Panchalingam S, Wu Y, Sow SO, Sur D, Breiman RF, Faruque AS, Zaidi AK, Saha D, Alonso PL, Tamboura B, Sanogo D, Onwuchekwa U, Manna B, Ramamurthy T, Kanungo S, Ochieng JB, Omore R, Oundo JO, Hossain A, Das SK, Ahmed S, Qureshi S, Quadri F, Adegbola RA, Antonio M, Hossain MJ, Akinsola A, Mandomando I, Nhampossa T, Acácio S, Biswas K, O'Reilly CE, Mintz ED, Berkeley LY, Muhsen K, Sommerfelt H, Robins-Browne RM, Levine MM. Burden and aetiology of diarrhoeal disease in infants and young children in developing countries (the Global Enteric Multicenter Study, GEMS): a prospective, case-control study. Lancet. 2013; 382(9888): 209–22.
Kotwal A, Singh H, Verma AK, Gupta RM, Jain S, Sinha S, Joshi RK, Teli P, Khunga V, Bhatnagar A, Ranjan R. A study of Hepatitis A and E virus seropositivity profile amongst young healthy adults in India. Med J Armed Forces India. 2014; 70(3): 225–9.
Koul R, Motta A, Razdan S. Epidemiology of young strokes in rural Kashmir, India. Acta Neurol Scand. 1990; 82(1): 1-3.
Koul R, Razdan S, Motta A. Prevalence and pattern of epilepsy (Lath/Mirgi/Laran) in rural Kashmir, India. Epilepsia. 1988; 29(2): 116-22.
Kownhar H, Shankar EM, Rajan R, Vengatesan A, Rao UA. Prevalence of Campylobacter jejuni and enteric bacterial pathogens among hospitalized HIV infected versus non-HIV infected patients with diarrhoea in southern India. Scand J Infect Dis. 2007; 39(10): 862–6.

Table 3: GBD 2016 India data inputs
Krishna BV, Patil A, Nadgir SD, Chandrasekhar MR. Incidence of Vibrio cholerae serogroup O139 infection with low virulence in Hubli, Karnataka (India). Indian J Pathol Microbiol. 2003; 46(1): 142–4.
Krishnaiah S, Das T, Nirmalan PK, Nutheti R, Shamanna BR, Rao GN, Thomas R. Risk factors for age-related macular degeneration: findings from the Andhra Pradesh eye disease study in South India. Invest Ophthalmol Vis Sci. 2005; 46(12): 4442-9.
Krishnamurthy S, Mondal N, Narayanan P, Biswal N, Srinivasan S, Soundravally R. Incidence and etiology of acute kidney injury in southern India. Indian J Pediatr. 2013; 80(3): 183–9.
Krishnan A, Amarchand R, Gupta V, Lafond KE, Suliankatchi RA, Saha S, Rai S, Misra P, Purakayastha DR, Wahi A, Sreenivas V, Kapil A, Dawood F, Pandav CS, Broor S, Kapoor SK, Lal R, Widdowson MA. Epidemiology of acute respiratory infections in children - preliminary results of a cohort in a rural north Indian community. BMC Infect Dis. 2015; 15: 462.
Krishnan A, Shah B, Lal V, Shukla DK, Paul E, Kapoor SK. Prevalence of risk factors for non-communicable disease in a rural area of Faridabad district of Haryana. Indian J Public Health. 2008; 52(3): 117-24.
Krishnan MN, Zachariah G, Venugopal K, Mohanan PP, Harikrishnan S, Sanjay G, Jeyaseelan L, Thankappan KR. Prevalence of coronary artery disease and its risk factors in Kerala, South India: a community-based cross-sectional study. BMC Cardiovasc Disord. 2013; 16: 12.
Kshirsagar NA, Gogtay NJ, Garg BS, Deshmukh PR, Rajgor DD, Kadam VS, Kirodian BG, Ingole NS, Mehendale AM, Fleckenstein L, Karbwang J, Lazdins-Helds JK. Safety, tolerability, efficacy and plasma concentrations of diethylcarbamazine and albendazole co-administration in a field study in an area endemic for lymphatic filariasis in India. Trans R Soc Trop Med Hyg. 2004; 98(4): 205-17.
Kulkarni S, Patsute S, Sane S, Chandane M, Vidhate P, Risbud A. Enteric pathogens in HIV infected and HIV uninfected individuals with diarrhea in Pune. Trans R Soc Trop Med Hyg. 2013; 107(10): 648–52.
Kulshrestha M, Vidyanand. An analysis of the risk factors and the outcomes of cerebrovascular diseases in northern India. J Clin Diagn Res. 2013; 7(1): 127–31.
Kumar A, Chand SK. Prevalence of Wuchereria bancrofti infection in some coastal villages of Ganjam, Orissa. J Commun Dis. 1990; 22(3): 209-12.
Kumar A, Dash AP, Mansing GD. Prevalence of filariasis in rural Puri, Orissa. J Commun Dis. 1994; 26(4): 215-20.
Kumar A, Husain S. The Burden of New Leprosy Cases in India: A Population-Based Survey in Two States. ISRN Trop Med. 2013; 329283.
Kumar A, Sachan P, Bajpei A, Singh T. A study to compare the effect of adding 12 days DEC regimen to 6 years annual mass drug administration to eliminate microfilaraemia infection in the community in rural Kanpur, Uttar Pradesh, India. J Commun Dis. 2013; 45.0(2-Jan): 33-40.
Kumar A, Sachan P. Measuring impact on filarial infection status in a community study: role of coverage of mass drug administration (MDA). Trop Biomed. 2014; 31.0(2): 225-9.
Kumar A, Yadav VS, Katoch K, Sachan P. Filariasis in Ghatampur Tahsil of Kanpur Nagar District: indications of high endemicity locus. J Commun Dis. 2006; 38(2): 155-9.
Kumar A. Human filariasis: infection rate as the uniform measurable criterion for filarial endemicity. J Commun Dis. 1996; 28(3): 163-7.
Kumar CSV, Anand Kumar H, Sunita V, Kapur I. Prevalence of anemia and worm infestation in school going girls at Gulbargha, Karnataka. Indian Pediatr. 2003; 40(1): 70-2.

Table 3: GBD 2016 India data inputs
Kumar D, Arora A, Singh NP, Kohli R, Kar P, Das BC. Hepatitis G virus infection in hemodialysis patients from urban Delhi. Ren Fail. 2005; 27(1): 87-93.
Kumar H, Gupta PK, Jaiprakash M. The Role of anti-HBc IgM in Screening of Blood Donors. Med J Armed Forces India. 2007; 63(4): 350-2.
Kumar J, Deshmukh PR, Garg BS. Prevalence and correlates of sustained hypertension in adolescents of rural Wardha, central India. Indian J Pediatr. 2012; 79(9): 1206-12.
Kumar KJ, Prakash P, Murthy DS, Manjunath VG. Prevalence and annual risk of tuberculosis infection in Rural Mysore. Indian Pediatr. 2011; 48(10): 797–799.
Kumar M, Kumar R, Hissar SS, Saraswat MK, Sharma BC, Sakhuja P, Sarin SK. Risk factors analysis for hepatocellular carcinoma in patients with and without cirrhosis: a case-control study of 213 hepatocellular carcinoma patients from India. J Gastroenterol Hepatol. 2007; 22(7): 1104-11.
Kumar M, Thakur S, Puri A, Shukla S, Sharma S, Perumal V, Chawla R, Gupta U. Fetal renal anomaly: factors that predict survival. J Pediatr Urol. 2014; 10(6): 1001-7.
Kumar P, Garhwal S, Chaudhary V. Rheumatic heart disease: a school survey in a rural area of Rajasthan. Indian Heart J. 1992; 44(4): 245-6.
Kumar P, Krishna P, Reddy SC, Gurappa M, Aravind SR, Munichoodappa C. Incidence of type 1 diabetes mellitus and associated complications among children and young adults: results from Karnataka Diabetes Registry 1995-2008. J Indian Med Assoc. 2008; 106(11): 708–11.
Kumar R, Bhave A, Bhargava R, Agarwal GG. Prevalence and risk factors for neurological disorders in children aged 6 months to 2 years in northern India. Dev Med Child Neurol. 2013; 55(4): 348-56.
Kumar R, Bumb RA, Ansari NA, Mehta RD, Salotra P. Cutaneous leishmaniasis caused by Leishmania tropica in Bikaner, India: parasite identification and characterization using molecular and immunologic tools. Am J Trop Med Hyg. 2007; 76(5): 896-901.
Kumar R, Kapoor SK, Krishnan A. Performance of cause-specific childhood mortality surveillance by health workers using a short verbal autopsy tool. Southeast Asia J Public Health. 2012; 1(2): 151-158.
Kumar R, Saraswat MK, Sharma BC, Sakhuja P, Sarin SK. Characteristics of hepatocellular carcinoma in India: a retrospective analysis of 191 cases. QJM. 2008; 101(6): 479-85.
Kumar R, Sharma AK, Barik S, Kumar V. Maternal mortality inquiry in a rural community of north India. Int J Gynaecol Obstet. 1989; 29(4): 313-9.
Kumar R, Vohra H, Chakraborty A, Sharma YP, Bandhopadhya S, Dhanda V, Sagar V, Sharma M, Shah B, Ganguly NK. Epidemiology of group A streptococcal pharyngitis and impetigo: a cross-sectional and follow up study in a rural community of northern India. Indian J Med Res. 2009; 130(6): 765-771.
Kumar R. Anthropometric and behavioral risk factor for noncommunicable diseases: a cluster survey from rural Wardha. Indian J Public Health. 2015; 59(1):61-4.
Kumar RK, Kesaree N. Blood lead levels in urban and rural Indian children. Indian Pediatr. 1999; 36(3): 303-6.
Kumar S, Kaushik A, Kaushik CP. Blood lead levels among populations differentially exposed to vehicular exhaust in Rohtak, India. Environ Pollut. 1993; 80(2): 173-6.
Kumar S, Radhakrishna CV, Jeetendra R, Kumar P, Chauhan LS, Srivastava R. Prevalence of tuberculous infection among school children in Kerala. Indian J Tuberc. 2009; 56(1): 10-16.

Table 3: GBD 2016 India data inputs
Kumar SG, Premarajan KC, Subitha L, Suguna E, Vinayagamoorthy, Kumar V. Prevalence and Pattern of Alcohol Consumption using Alcohol Use Disorders Identification Test (AUDIT) in Rural Tamil Nadu, India. J Clin Diagn Res. 2013; 7(8): 1637-9.
Kumar V, Datta N, Wadhwa SS, Singhi S. Morbidity and mortality in diarrhea in rural Haryana Indian. Indian J Pediatr. 1985; 52(418): 455-61.
Kumar V, Garg BS. Global health and infant mortality: application of verbal autopsy tool to categorize infant deaths, ascertain their causes and identify the gaps in health management information system in India. Int J Curr Res Rev. 2014; 5(15): 87-94.
Kumar V, Singh AJ, Marwaha RK. An epidemiological study of congenital malformations in rural children. Indian Pediatr. 1994; 31(8): 909-14.
Kumari-Indira KS, Sivaraman S, Joshi M, Pillai NS. Annual risk of tuberculosis infection: an estimate from ten year old children in Trivandrum District. Indian J Tuberc. 2000; 47(4): 211–218.
Kurien T, Thyagarajan SP, Jeyaseelan L, Peedicayil A, Rajendran P, Sivaram S, Hansdak SG, Renu G, Krishnamurthy P, Sudhakar K, Varghese JC, STD Study Group. Community prevalence of hepatitis B infection and modes of transmission in Tamil Nadu, India. Indian J Med Res. 2005; 121(5): 670-5.
Kurthkoti AG, Singh H. Changes in the prevalence rates of infection in younger age groups in a rural population of Bangalore district over a period of 5 years. NTI Newsletter. 1985; 21: 28–40.
Kuruvila M, Dubey S, Gahalaut P. Pattern of skin diseases among migrant construction workers in Mangalore. Indian J Dermatol Venereol Leprol. 2006; 72(2): 129-32.
Kusuma Y, Das P. Hypertension in Orissa, India: a cross-sectional study among some tribal, rural and urban populations. Public Health. 2008; 122(10): 1120-3.
Kusuma Y, Gupta S, Pandav C. Migration and hypertension: a cross-sectional study among neo-migrants and settled-migrants in Delhi, India. Asia Pac J Public Health. 2009; 21(4): 497-507.
Kusuma YS, Babu BV, Naidu JM. Blood pressure levels among cross-cultural populations of Visakhapatnam district, Andhra Pradesh, India. Ann Hum Biol. 2002; 29(5): 502-12.
Kutty VR, Soman CR, Joseph A, Pisharody R, Vijayakumar K. Type 2 diabetes in southern Kerala: variation in prevalence among geographic divisions within a region. Natl Med J India. 2000; 13(6): 287-92.
Laharwal MA, Sarmast AH, Ramzan AU, Wani AA, Malik NK, Arif SH, Rizvi M. Epidemiology of the neural tube defects in Kashmir Valley. Surg Neurol Int. 2016; 7: 35.
Lai CK, Beasley R, Crane J, Foliaki S, Shah J, Weiland S. Global variation in the prevalence and severity of asthma symptoms: phase three of the International Study of Asthma and Allergies in Childhood (ISAAC). Thorax. 2009; 64(6): 476-483.
Lakhanpal U, Rathore MS. Epidemiology of measles in rural area of Punjab. J Commun Dis. 1986; 18(3): 185-8.
Latoo JA, Masoodi NA, Bhat NA, Khan GQ, Kadla SA. The ABO and Rh Blood groups in Kashmiri Population. Indian J Pract Dr. 2006; 3(2).
Laxmaiah A, Arlappa N, Balakrishna N, Mallikarjuna Rao K, Galreddy C, Kumar S, Ravindranath M, Brahmam GNV. Prevalence and determinants of micronutrient deficiencies among rural children of eight states in India. Ann Nutr Metab. 2013; 62(3): 231-41.

Table 3: GBD 2016 India data inputs
Libu GK, Bina T, Raphael L, Balakrishnan SE, Biju G, Samson JF, Bindu V. Prevalence and socio-demographic determinants of skin disease among lower primary school children in Calicut, Kerala. Kerala Med J. 2010; 10(5):185–90.
Limburg H, Kumar R. Follow-up study of blindness attributed to cataract in Karnataka State, India. Ophthalmic Epidemiol. 1998; 5(4): 211-23.
Limburg H, Vasavada AR, Muzumdar G, Khan M Y, Vaidyanathan K, Trivedi R, Bhatt D. Rapid assessment of cataract blindness in an urban district of Gujarat. Indian J Ophthalmol. 1999; 47(2): 135-141.
Llibre Rodriguez JJ, Ferri CP, Acosta D, Guerra M, Huang Y, Jacob KS, Krishnamoorthy ES, Salas A, Sosa AL, Acosta I, Dewey ME, Gaona C, Jotheeswaran AT, Li S, Rodriguez D, Rodriguez G, Kumar PS, Valhuerdi A, Prince M, 10/66 Dementia Research Group. Prevalence of dementia in Latin America, India, and China: a population-based cross-sectional survey. Lancet. 2008; 372(9637): 464-74.
Lobo J, Reddaiah V, Kapoor S, Nath L. Epidemiology of measles in a rural community. Indian J Pediatr. 1987; 54(2): 261-5.
Lokeshwar MR, Agrawal A, Subbarao SD, Chakraborty MS, Ram Prasad AV, Weil J, Bock HL, Kanwal S, Shah RC, Shah N. Age related seroprevalence of antibodies to varicella in India. Indian Pediatr. 2000; 37(7): 714-9.
London School of Hygiene and Tropical Medicine. Global Atlas of Helminth Infections - Lymphatic Filariasis. London, United Kingdom: London School of Hygiene and Tropical Medicine.
London School of Hygiene and Tropical Medicine. Global Atlas of Helminth Infections - Soil Transmitted Helminths. London, United Kingdom: London School of Hygiene and Tropical Medicine.
Luwang N, Gupta V. Anaemia in pregnancy in a rural community-influence of dietary intake in the multifactorial aetiology. Indian J Nutr Diet. 1980; 17(11): 414-7.
Mackenzie I. Hearing Impairment in Asia - final report of four country survey, as provided by the Global Burden of Disease 2010 Hearing Impairment Expert Group. [Data shared for this analysis]
Madhavan HN, Priya K. The diagnostic significance of enzyme linked immuno-sorbent assay for herpes simplex, varicella zoster and cytomegalovirus retinitis. Indian J Ophthalmol. 2003; 51(1): 71-5.
Madhivanan P, Bartman MT, Pasutti L, Krupp K, Arun A, Reingold AL, Klausner JD. Prevalence of Trichomonas vaginalis infection among young reproductive age women in India: implications for treatment and prevention. Sex Health. 2009; 6(4): 339-44.
Madhivanan P, Chen Y-H, Krupp K, Arun A, Klausner JD, Reingold AL. Incidence of herpes simplex virus type 2 in young reproductive age women in Mysore, India. Indian J Pathol Microbiol. 2011; 54(1): 96–9.
Madras Diabetes Research Foundation & M. V. Diabetes Specialities Centre. India - Chennai Urban Population Study Blood Glucose, Cholesterol, BMI, and Diabetes Incidence Measurements, 1996-2006. [Data shared for this analysis]
Madras Diabetes Research Foundation & M. V. Diabetes Specialities Centre. India - Chennai Urban Rural Epidemiology Study Blood Glucose, Cholesterol, BMI, and Diabetes Incidence Measurements, 2001-2013. [Data shared for this analysis]
Mahajan RK, Walia TPS, Sumanjit. Stripping voltammetric determination of zinc, cadmium, lead and copper in blood samples of children aged between 3 months and 6 years. Online J Health Allied Sci. 2005; 1(2).
Mahanta TG, Joshi R, Mahanta BN, Xavier D. Prevalence of modifiable cardiovascular risk factors among tea garden and general population in Dibrugarh, Assam, India. J Epidemiol Glob Health. 2013; 3(3): 147–56.

Table 3: GBD 2016 India data inputs
Mahapatra A, Geddam JJ, Marai N, Murmu B, Mallick G, Bulliyya G, Acharya AS, Satyanarayana K. Nutritional status of preschool children in the drought affected Kalahandi district of Orissa. Indian J Med Res. 2000; 111: 90-4.
Mahavarkar SH, Madhu CK, Mule VD. A comparative study of teenage pregnancy. J Obstet Gynaecol. 2008; 28(6): 604-7.
Mahesh PA, Jayaraj BS, Chaya SK, Lokesh KS, McKay AJ, Prabhakar AK, Pape UJ. Variation in the prevalence of chronic bronchitis among smokers: a cross-sectional study. Int J Tuberc Lung Dis. 2014; 18(7): 862-9.
Makroo RN, Hassain G, Koul A, Shah GN. Prevalence of hepatitis B surface antigen in Kashmiri blood donors. Indian J Med Res. 1989; 89: 310-3.
Makroo RN, Walia RS, Chowdhry M, Bhatia A, Hegde V, Rosamma NL. Seroprevalence of anti-HCV antibodies among blood donors of north India. Indian J Med Res. 2013; 138(1): 125–8.
Malaria Atlas Project. Annual Parasite Incidence Database. Oxford, United Kingdom: Malaria Atlas Project.
Malaria Atlas Project. Plasmodium Falciparum Parasite Rate Database. Oxford, United Kingdom: Malaria Atlas Project.
Malhotra P, Kumari S, Kumar R, Jain S, Sharma BK. Prevalence and determinants of hypertension in an un-industrialised rural population of North India. J Hum Hypertens. 1999; 13(7): 467-72.
Malhotra S, Kohli A, Arun P. Prevalence of psychiatric disorders in school children in Chandigarh, India. Indian J Med Res. 2002; 116: 21-8.
Mall ML, Rai RR, Philip M, Naik G, Parekh P, Bhawnani SC, Olowokure B, Shamanna M, Weil J. Seroepidemiology of hepatitis A infection in India: changing pattern. Indian J Gastroenterol. 2001; 20(4): 132-5.
Mallik AK, Pandav CS, Achar DP, Anand K, Lobo J, Karmarkar MG, Nath LM. Iodine deficiency disorders in Car Nicobar (Andaman and Nicobar Islands). Natl Med J India. 1998; 11(1): 9-11.
Malvi S, Appannanavar S, Mohan B, Kaur H, Gautam N, Bharti B, Kumar Y, Taneja N. Comparative analysis of virulence determinants, antibiotic susceptibility patterns and serogrouping of atypical enteropathogenic Escherichia coli versus typical enteropathogenic E. coli in India. J Med Microbiol. 2015; 64(10): 1208-15.
Mandal NN, Bal MS, Das MK, Achary KG, Kar SK. Lymphatic filariasis in children: age dependent prevalence in an area of India endemic for Wuchereria bancrofti infection. Trop Biomed. 2010; 27(1): 41-6.
Mangal N, Shah K, Sitaraman S. Epidemiological study of measles in urban (slum) area of Jaipur. Indian Pediatr. 1990; 27(11): 1216-7.
Mani GG, Rao ST, Madhavi R. Estimation of hookworm intensity by anthelmintic expulsion in primary schoolchildren in south India. Trans R Soc Trop Med Hyg. 1993; 87(6): 634–5.
Mani KS, Rangan G, Srinivas HV, Kalyanasundaram S, Narendran S, Reddy AK. The Yelandur study: a community-based approach to epilepsy in rural South India – epidemiological aspects. Seizure. 1998; 7(4): 281-8.
Mani TR, Rajendran R, Munirathinam A, Sunish IP, Md Abdullah S, Augustin DJ, Satyanarayana K. Efficacy of co-administration of albendazole and diethylcarbamazine against geohelminthiases: a study from South India. Trop Med Int Health. 2002; 7(6): 541–8.
Manimunda SP, Sugunan AP, Sha WA, Singh SS, Shriram AN, Vijayachari P. Tsunami, post-tsunami malaria situation in Nancowry group of islands, Nicobar district, Andaman and Nicobar Islands. Indian J Med Res. 2011;133: 76-82.
Marmamula S, Narsaiah S, Shekhar K, Khanna RC, Rao GN. Visual impairment in the South Indian state of Andhra Pradesh: Andhra Pradesh - rapid assessment of visual impairment (AP-RAVI) project. PLoS One. 2013; 8(7.0): e70120.
Maru AM, Narendran S. Epidemiology of dental caries among adults in a rural area in India. J Contemp Dent Pract. 2012; 13(3): 382-8.
Marwaha RK, Garg MK, Bhadra K, Mithal A, Tandon N. Assessment of lean (muscle) mass and its distribution by dual energy X-ray absorptiometry in healthy Indian females. Arch Osteoporos. 2014; 9: 186.

Table 3: GBD 2016 India data inputs
Mathad V, Metgud C, Mallapur MD. Nutritional status of under-fives in rural area of South India. Indian J Med Sci. 2011; 65(4): 151-6.
Mathan VI, Rajan DP. The prevalence of bacterial intestinal pathogens in a healthy rural population in southern India. J Med Microbiol. 1986; 22(2): 93-6.
Mathers BM, Degenhardt L, Phillips B, Wiessing L,Hickman M, Strathdee SA, Wodak A, Panda S, Tyndall M, Toufik A, Mattick RP. Global epidemiology of injecting drug use and HIV among people who inject drugs: a systematic review. Lancet. 2008; 372: 1733–1745.
Mathias K, Goicolea I, Kermode M, Singh L, Shidhaye R, Sebastian MS. Cross-sectional study of depression and help-seeking in Uttarakhand, North India. BMJ Open. 2015; 5(11): e008992.
Mathur R, Reddy V, Naidu AN, Ravikumar, Krishnamachari KA. Nutritional status and diarrhoeal morbidity: a longitudinal study in rural Indian preschool children . Hum Nutr Clin Nutr. 1985; 39(6): 447-54.
Mathur S, Dubey T, Kulshrestha M, Agarwal H, Mathur G, Mathur A, Mathur S, Prakash P, Rajpurohit V, Gehlot G, Tak P. Clinical profile and mortality among novel influenza A (H1N1) infected patients: 2009-2010 Jodhpur, Rajasthan pandemic. J Assoc Physicians India. 2013; 61(9): 627-32.
Mathuranath PS, Cherian PJ, Mathew R, Kumar S, George A, Alexander A, Ranjith N, Sarma PS. Dementia in Kerala, South India: prevalence and influence of age, education and gender. Int J Geriatr Psychiatry. 2010; 25(3): 290-7.
Matsuzaki M, Kuper H, Kulkarni B, Radhakrishna KV, Viljakainen H, Taylor AE, Sullivan R, Bowen L, Tobias JH, Ploubidis GB, Wells JC, Prabhakaran D, Davey Smith G, Ebrahim S, Ben-Shlomo Y, Kinra S. Life-course determinants of bone mass in young adults from a transitional rural community in India: the Andhra Pradesh Children and Parents Study (APCAPS). Am J Clin Nutr. 2014; 99(6): 1450-9.
Max Planck Institute for Demographic Research, Vienna Institute of Demography. Human Fertility Collection - Total Fertility Rate and Mean Age at Birth. Rostock, Germany: Max Planck Institute for Demographic Research.
Mayurnath S, Vallishayee RS, Radhamani MP, Prabhakar R. Prevalence study of tuberculous infection over fifteen years, in a rural population in Chingleput district (south India). Indian J Med Res. 1991; 93: 74–80.
McClure EM, Pasha O, Goudar SS, Chomba E, Garces A, Tshefu A, Althabe F, Esamai F, Patel A, Wright LL, Moore J, Kodkany BS, Belizan JM, Saleem S, Derman RJ, Carlo WA, Hambidge KM, Buekens P, Liechty EA, Bose C, Koso-Thomas M, Jobe AH, Goldenberg RL; Global Network Investigators. Epidemiology of stillbirth in low-middle income countries: A Global Network Study. Acta Obstet Gynecol Scand. 2011; 90(12): 1379-85.
Medhi GK, Hazarika NC, Shah B, Mahanta J. Study of health problems and nutritional status of tea garden population of Assam. Indian J Med Sci. 2006; 60(12): 496-505.
Medhi GK, Mahanta J, Akoijam BS, Adhikary R. Size estimation of injecting drug users (IDU) using multiplier method in five Districts of India. Subst Abuse Treat Prev Policy. 2012; 7: 9.
Mehra R, Badhan K, Kansal S, Sonkawade RG. Assessment of seasonal indoor radon concentration in dwellings of Western Haryana. Radiat Meas. 2011; 46(12): 1803-6.
Mehrotra SK, Mathur JS, Maheshwari BB. Epidemiological aspects of nutritional anaemia in children below five years. Indian J Pediatr. 1976; 43(340): 132-5.
Mehta P, Joseph A, Verghese A. An epidemiologic study of psychiatric disorders in a rural area in Tamilnadu. Indian J Psychiatry. 1985; 27(2): 153-8.
Mehta S, Kumar P, Narang A. A randomized controlled trial of fluid supplementation in term neonates with severe hyperbilirubinemia. J Pediatr. 2005; 147(6): 781-5.

Table 3: GBD 2016 India data inputs
Memon S, Shaikh S, Bibi S. To compare the outcome (early) of neonates with birth asphyxia inrelation to place of delivery and age at time of admission. J Pak Med Assoc. 2012; 62(12): 1277-81.
Menon NV, Peethambaran G, Puthiyapurayil AT, Nambudakath C, Arakkal R. Clinical profile and juvenile arthritis damage index in children with juvenile idiopathic arthritis: A study from a tertiary care center in south India. Int J Rheum Dis. 2016.
Menon VK, Sarkar R, Moses PD, Agarwal I, Simon A, Kang G. Norovirus genogroup II gastroenteritis in hospitalized children in South India. Am J Trop Med Hyg. 2013; 89(5): 1019-22.
Merikangas KR, Jin R, He J-P, Kessler RC, Lee S, Sampson NA, Viana MC, Andrade LH, Hu C, Karam EG, Ladea M, Medina-Mora ME, Ono Y, Posada-Villa J, Sagar R, Wells JE, Zarkov Z. Prevalence and correlates of bipolar spectrum disorder in the World Mental Health Survey Initiative. Arch Gen Psychiatry. 2011; 68(3): 241-51.
Meshram II, Arlappa N, Balakrishna N, Laxmaiah A, Mallikarjun Rao K, Gal Reddy C, Ravindranath M, Sharad Kumar S, Brahmam GNV. Prevalence and determinants of undernutrition and its trends among pre-school tribal children of Maharashtra State, India. J Trop Pediatr. 2012; 58(2): 125-32.
Meshram II, Arlappa N, Balakrishna N, Mallikharjuna Rao K, Laxmaiah A, Brahmam GN. Trends in the prevalence of undernutrition, nutrient & food intake and predictors of undernutrition among under five year tribal children in India. Asia Pac J Clin Nutr. 2012; 21(4): 568-76.
Meshram II, Balakrishna N, Arlappa N, Rao KM, Laxmaiah A, Brahmam GNV. Prevalence of Undernutrition, Its Determinants, and Seasonal Variation Among Tribal Preschool Children of Odisha State, India. Asia Pac J Public Health. 2014; 26(5): 470-80.
Meshram II, Kodavanti MR, Chitty GR, Manchala R, Kumar S, Kakani SK, Kodavalla V, Avula L, Ginnela Narsimhachary Veera B. Influence of Feeding Practices and Associated Factors on the Nutritional Status of Infants in Rural Areas of Madhya Pradesh State, India. Asia Pac J Public Health. 2015; 27(2): NP1345-61.
Meshram II, Laxmaiah A, Venkaiah K, Brahmam GNV. Impact of feeding and breastfeeding practices on the nutritional status of infants in a district of Andhra Pradesh, India. Natl Med J India. 2012; 25(4): 2016.
Midha T, Idris MZ, Saran RK, Srivastav AK, Singh SK. Prevalence and determinants of hypertension in the urban and rural population of a north Indian district. East Afr J Public Health. 2009; 6(3): 268–73.
Ministry of Health and Family Welfare, Government of India, International Institute for Population Sciences, Centers for Disease Control and Prevention (CDC), Johns Hopkins Bloomberg School of Public Health, Research Triangle Institute, Inc., World Health Organization. India Global Adult Tobacco Survey 2009-2010. Atlanta, United States: CDC.
Ministry of Health and Family Welfare, Government of India, National Institute of Mental Health and Neurosciences, World Health Organization. India National Mental Health Survey Data Tables 2015-2016. [Data shared for this analysis]
Ministry of Health and Family Welfare, Government of India, National Institute of Mental Health and Neurosciences, World Health Organization. India National Mental Health Survey Report 2015-2016. New Delhi, India: Ministry of Health and Family Welfare; 2016.
Ministry of Health and Family Welfare, Government of India, United Nations Children's Fund. India Coverage Evaluation Survey 2001.
Ministry of Health and Family Welfare, Government of India, United Nations Children's Fund. India Coverage Evaluation Survey 2002.

Table 3: GBD 2016 India data inputs
Ministry of Health and Family Welfare, Government of India, United Nations Children's Fund. India Coverage Evaluation Survey 2005.
Ministry of Health and Family Welfare, Government of India, United Nations Children's Fund. India Coverage Evaluation Survey 2007.
Ministry of Health and Family Welfare, Government of India, United Nations Children's Fund. India Coverage Evaluation Survey Data 2009-2010. [Data shared for this analysis]
Ministry of Health and Family Welfare, Government of India, United Nations Children's Fund. India Evaluation of Routine Immunization Coverage Survey 1997-1998.
Ministry of Health and Family Welfare, Government of India, United Nations Children's Fund. India Evaluation of Routine Immunization Coverage Survey 1998-1999.
Ministry of Health and Family Welfare, Government of India, World Health Organization - Regional Office for South-East Asia and Country Office for India, Centers for Disease Control and Prevention. India-Tripura Global Youth Tobacco Survey 2000. New Delhi, India: Ministry of Health and Family Welfare.
Ministry of Health and Family Welfare, Government of India, World Health Organization - Regional Office for South-East Asia and Country Office for India, Centers for Disease Control and Prevention. India-Uttar Pradesh Global Youth Tobacco Survey 2002. New Delhi, India: Ministry of Health and Family Welfare.
Ministry of Health and Family Welfare, Government of India, World Health Organization - Regional Office for South-East Asia and Country Office for India, Centers for Disease Control and Prevention. India-Uttaranchal Global Youth Tobacco Survey 2002. New Delhi, India: Ministry of Health and Family Welfare.
Ministry of Health and Family Welfare, Government of India, World Health Organization - Regional Office for South-East Asia and Country Office for India, Centers for Disease Control and Prevention. India-West Bengal Global Youth Tobacco Survey 2000. New Delhi, India: Ministry of Health and Family Welfare.
Ministry of Health and Family Welfare, Government of India, World Health Organization - Regional Office for South-East Asia and Country Office for India, Centers for Disease Control and Prevention. India Global Youth Tobacco Survey 2006. New Delhi, India: Ministry of Health and Family Welfare.
Ministry of Health and Family Welfare, Government of India, World Health Organization - Regional Office for South-East Asia and Country Office for India, Centers for Disease Control and Prevention. India Global Youth Tobacco Survey 2009. New Delhi, India: Ministry of Health and Family Welfare.
Ministry of Health and Family Welfare, Government of India, World Health Organization - Regional Office for South-East Asia and Country Office for India, Centers for Disease Control and Prevention. India-Arunachal Pradesh Global Youth Tobacco Survey 2000. New Delhi, India: Ministry of Health and Family Welfare.
Ministry of Health and Family Welfare, Government of India, World Health Organization - Regional Office for South-East Asia and Country Office for India, Centers for Disease Control and Prevention. India-Assam Global Youth Tobacco Survey 2000. New Delhi, India: Ministry of Health and Family Welfare.
Ministry of Health and Family Welfare, Government of India, World Health Organization - Regional Office for South-East Asia and Country Office for India, Centers for Disease Control and Prevention. India-Bihar Global Youth Tobacco Survey 2000. New Delhi, India: Ministry of Health and Family Welfare.

Table 3: GBD 2016 India data inputs
Ministry of Health and Family Welfare, Government of India, World Health Organization - Regional Office for South-East Asia and Country Office for India, Centers for Disease Control and Prevention. India-Goa Global Youth Tobacco Survey 2000. NNew Delhi, India: Ministry of Health and Family Welfare.
Ministry of Health and Family Welfare, Government of India, World Health Organization - Regional Office for South-East Asia and Country Office for India, Centers for Disease Control and Prevention. India-Gujarat Global Youth Tobacco Survey 2004. New Delhi, India: Ministry of Health and Family Welfare.
Ministry of Health and Family Welfare, Government of India, World Health Organization - Regional Office for South-East Asia and Country Office for India, Centers for Disease Control and Prevention. India-Gujarat with Ahmedabad Global Youth Tobacco Survey 2004. New Delhi, India: Ministry of Health and Family Welfare.
Ministry of Health and Family Welfare, Government of India, World Health Organization - Regional Office for South-East Asia and Country Office for India, Centers for Disease Control and Prevention. India-Haryana Global Youth Tobacco Survey 2004. New Delhi, India: Ministry of Health and Family Welfare.
Ministry of Health and Family Welfare, Government of India, World Health Organization - Regional Office for South-East Asia and Country Office for India, Centers for Disease Control and Prevention. India-Jammu and Kashmir Global Youth Tobacco Survey 2004. New Delhi, India: Ministry of Health and Family Welfare.
Ministry of Health and Family Welfare, Government of India, World Health Organization - Regional Office for South-East Asia and Country Office for India, Centers for Disease Control and Prevention. India-Karnataka Global Youth Tobacco Survey 2003. New Delhi, India: Ministry of Health and Family Welfare.
Ministry of Health and Family Welfare, Government of India, World Health Organization - Regional Office for South-East Asia and Country Office for India, Centers for Disease Control and Prevention. India-Madhya Pradesh Global Youth Tobacco Survey 2004. New Delhi, India: Ministry of Health and Family Welfare.
Ministry of Health and Family Welfare, Government of India, World Health Organization - Regional Office for South-East Asia and Country Office for India, Centers for Disease Control and Prevention. India-Maharashtra Global Youth Tobacco Survey 2000. New Delhi, India: Ministry of Health and Family Welfare.
Ministry of Health and Family Welfare, Government of India, World Health Organization - Regional Office for South-East Asia and Country Office for India, Centers for Disease Control and Prevention. India-Manipur Global Youth Tobacco Survey 2000. New Delhi, India: Ministry of Health and Family Welfare.
Ministry of Health and Family Welfare, Government of India, World Health Organization - Regional Office for South-East Asia and Country Office for India, Centers for Disease Control and Prevention. India-Meghalaya Global Youth Tobacco Survey 2000. New Delhi, India: Ministry of Health and Family Welfare.
Ministry of Health and Family Welfare, Government of India, World Health Organization - Regional Office for South-East Asia and Country Office for India, Centers for Disease Control and Prevention. India-Mizoram Global Youth Tobacco Survey 2000. New Delhi, India: Ministry of Health and Family Welfare.
Ministry of Health and Family Welfare, Government of India, World Health Organization - Regional Office for South-East Asia and Country Office for India, Centers for Disease Control and Prevention. India-Nagaland Global Youth Tobacco Survey 2000. New Delhi, India: Ministry of Health and Family Welfare.
Ministry of Health and Family Welfare, Government of India, World Health Organization - Regional Office for South-East Asia and Country Office for India, Centers for Disease Control and Prevention. India-Punjab Global Youth Tobacco Survey 2004. New Delhi, India: Ministry of Health and Family Welfare.

Table 3: GBD 2016 India data inputs
Ministry of Health and Family Welfare, Government of India, World Health Organization - Regional Office for South-East Asia and Country Office for India, Centers for Disease Control and Prevention. India-Rajasthan Global Youth Tobacco Survey 2003. New Delhi, India: Ministry of Health and Family Welfare.
Ministry of Health and Family Welfare, Government of India, World Health Organization - Regional Office for South-East Asia and Country Office for India, Centers for Disease Control and Prevention. India-Sikkim Global Youth Tobacco Survey 2000. New Delhi, India: Ministry of Health and Family Welfare.
Ministry of Health and Family Welfare, Government of India, World Health Organization - Regional Office for South-East Asia and Country Office for India, Centers for Disease Control and Prevention. India-Tamil Nadu Global Youth Tobacco Survey 2000. New Delhi, India: Ministry of Health and Family Welfare.
Ministry of Health and Family Welfare, Government of India. India Survey of Blindness 1986-1989. [Data shared for this analysis]
Ministry of Social Justice and Empowerment, Government of India, Department of Health, Government of Punjab, National Drug Dependence Treatment Centre, All India Institute of Medical Sciences, Society for the Promotion of Youth and Masses. India - Punjab Opioid Dependence Survey Report 2015. Available from: http://pbhealth.gov.in/scan0003%20(2).pdf
Ministry of Social Justice and Empowerment, Government of India, United Nations Office on Drugs and Crime. Extent, Pattern and Trends of Drug Abuse in India. New Delhi, India: Ministry of Social Justice and Empowerment; 2004.
Ministry of Statistics and Programme Implementation, Government of India. Common Property Resources, Sanitation & Hygiene Services, National Sample Survey Round 54, January - June 1998. New Delhi, India: Ministry of Statistics and Programme Implementation.
Ministry of Statistics and Programme Implementation, Government of India. Consumer Expenditure Survey, India National Sample Survey Round 55, July 1999-June 2000. New Delhi, India: Ministry of Statistics and Programme Implementation.
Ministry of Statistics and Programme Implementation, Government of India. Consumer Expenditure Survey, National Sample Survey Round 38, January-December 1983. New Delhi, India: Ministry of Statistics and Programme Implementation.
Ministry of Statistics and Programme Implementation, Government of India. Consumer Expenditure Survey, National Sample Survey Round 43, July 1987-June 1988. New Delhi, India: Ministry of Statistics and Programme Implementation.
Ministry of Statistics and Programme Implementation, Government of India. Consumer Expenditure Survey, National Sample Survey Round 46, July 1990-June 1991. New Delhi, India: Ministry of Statistics and Programme Implementation.
Ministry of Statistics and Programme Implementation, Government of India. Consumer Expenditure Survey, National Sample Survey Round 47, July - December 1991. New Delhi, India: Ministry of Statistics and Programme Implementation.
Ministry of Statistics and Programme Implementation, Government of India. Consumer Expenditure Survey, National Sample Survey Round 48, January-December 1992. New Delhi, India: Ministry of Statistics and Programme Implementation.
Ministry of Statistics and Programme Implementation, Government of India. Consumer Expenditure Survey, National Sample Survey Round 49, January-June 1993. New Delhi, India: Ministry of Statistics and Programme Implementation.

Table 3: GBD 2016 India data inputs
Ministry of Statistics and Programme Implementation, Government of India. Consumer Expenditure Survey, National Sample Survey Round 50, July 1993-June 1994. New Delhi, India: Ministry of Statistics and Programme Implementation.
Ministry of Statistics and Programme Implementation, Government of India. Consumer Expenditure Survey, National Sample Survey Round 50, July 1994-June 1995. New Delhi, India: Ministry of Statistics and Programme Implementation.
Ministry of Statistics and Programme Implementation, Government of India. Consumer Expenditure Survey, National Sample Survey Round 52, July 1995-June 1996. New Delhi, India: Ministry of Statistics and Programme Implementation.
Ministry of Statistics and Programme Implementation, Government of India. Consumer Expenditure Survey, National Sample Survey Round 53, January - December 1997. New Delhi, India: Ministry of Statistics and Programme Implementation.
Ministry of Statistics and Programme Implementation, Government of India. Consumer Expenditure Survey, National Sample Survey Round 54, January - June 1998. New Delhi, India: Ministry of Statistics and Programme Implementation.
Ministry of Statistics and Programme Implementation, Government of India. Consumer Expenditure Survey, National Sample Survey Round 56, July 2000 - June 2001. New Delhi, India: Ministry of Statistics and Programme Implementation.
Ministry of Statistics and Programme Implementation, Government of India. Consumer Expenditure Survey, National Sample Survey Round 57, July 2001 - June 2002. New Delhi, India: Ministry of Statistics and Programme Implementation.
Ministry of Statistics and Programme Implementation, Government of India. Consumer Expenditure Survey, National Sample Survey Round 58, July - December 2002. New Delhi, India: Ministry of Statistics and Programme Implementation.
Ministry of Statistics and Programme Implementation, Government of India. Consumer Expenditure Survey, National Sample Survey Round 59, January - December 2003. New Delhi, India: Ministry of Statistics and Programme Implementation.
Ministry of Statistics and Programme Implementation, Government of India. Consumer Expenditure Survey, National Sample Survey Round 60, January-June 2004. New Delhi, India: Ministry of Statistics and Programme Implementation.
Ministry of Statistics and Programme Implementation, Government of India. Consumer Expenditure Survey, National Sample Survey Round 61, July 2004 - June 2005. New Delhi, India: Ministry of Statistics and Programme Implementation.
Ministry of Statistics and Programme Implementation, Government of India. Consumer Expenditure Survey, National Sample Survey Round 62, July 2005 - June 2006. New Delhi, India: Ministry of Statistics and Programme Implementation.
Ministry of Statistics and Programme Implementation, Government of India. Consumer Expenditure Survey, National Sample Survey Round 63, July 2006- June 2007. New Delhi, India: Ministry of Statistics and Programme Implementation.
Ministry of Statistics and Programme Implementation, Government of India. Consumer Expenditure Survey, National Sample Survey Round 64, July 2007- June 2008. New Delhi, India: Ministry of Statistics and Programme Implementation.

Table 3: GBD 2016 India data inputs
Ministry of Statistics and Programme Implementation, Government of India. Consumer Expenditure Survey, National Sample Survey Round 66, July 2009-June 2010. New Delhi, India: Ministry of Statistics and Programme Implementation.
Ministry of Statistics and Programme Implementation, Government of India. Consumer Expenditure Survey, National Sample Survey Round 68, July 2011- June 2012. New Delhi, India: Ministry of Statistics and Programme Implementation.
Ministry of Statistics and Programme Implementation, Government of India. Drinking Water, Sanitation, Hygiene and Housing Condition Survey, National Sample Survey Round 69, July-December 2012. New Delhi, India: Ministry of Statistics and Programme Implementation.
Ministry of Statistics and Programme Implementation, Government of India. Employment and Unemployment Survey, India National Sample Survey Round 55, July 1999-June 2000. New Delhi, India: Ministry of Statistics and Programme Implementation.
Ministry of Statistics and Programme Implementation, Government of India. Employment and Unemployment Survey, National Sample Survey Round 38, January-December 1983. New Delhi, India: Ministry of Statistics and Programme Implementation.
Ministry of Statistics and Programme Implementation, Government of India. Employment and Unemployment Survey, National Sample Survey Round 43, July 1987- June 1988. New Delhi, India: Ministry of Statistics and Programme Implementation.
Ministry of Statistics and Programme Implementation, Government of India. Employment and Unemployment Survey, National Sample Survey Round 50, July 1993- June 1994. New Delhi, India: Ministry of Statistics and Programme Implementation.
Ministry of Statistics and Programme Implementation, Government of India. Employment and Unemployment Survey, National Sample Survey Round 50, July 1994-June 1995. New Delhi, India: Ministry of Statistics and Programme Implementation.
Ministry of Statistics and Programme Implementation, Government of India. Employment and Unemployment Survey, National Sample Survey Round 61, July 2004- June 2005. New Delhi, India: Ministry of Statistics and Programme Implementation.
Ministry of Statistics and Programme Implementation, Government of India. Employment and Unemployment Survey, National Sample Survey Round 62, July 2005- June 2006. New Delhi, India: Ministry of Statistics and Programme Implementation.
Ministry of Statistics and Programme Implementation, Government of India. Employment and Unemployment Survey, National Sample Survey Round 64, July 2007- June 2008. New Delhi, India: Ministry of Statistics and Programme Implementation.
Ministry of Statistics and Programme Implementation, Government of India. Employment and Unemployment Survey, National Sample Survey Round 66, July 2009-June 2010. New Delhi, India: Ministry of Statistics and Programme Implementation.
Ministry of Statistics and Programme Implementation, Government of India. Employment and Unemployment Survey, National Sample Survey Round 68, July 2011- June 2012. New Delhi, India: Ministry of Statistics and Programme Implementation.
Ministry of Statistics and Programme Implementation, Government of India. Employment and Unemployment surveys, National Sample Survey Round 60, January-June 2004. New Delhi, India: Ministry of Statistics and Programme Implementation.

Table 3: GBD 2016 India data inputs
Ministry of Statistics and Programme Implementation, Government of India. Household expenditure on services and durable goods survey, National Sample Survey Round 72, July 2014 - June 2015. New Delhi, India: Ministry of Statistics and Programme Implementation.
Ministry of Statistics and Programme Implementation, Government of India. Housing Condition Survey, National Sample Survey Round 58, July - December 2002. New Delhi, India: Ministry of Statistics and Programme Implementation.
Ministry of Statistics and Programme Implementation, Government of India. Housing Condition Survey, National Sample Survey Round 65, July 2008- June 2009. New Delhi, India: Ministry of Statistics and Programme Implementation.
Ministry of Statistics and Programme Implementation, Government of India. India State Series: Gross State Domestic Product at Factor Cost by Industry of Origin Tables 1980-81 to 1993-94. New Delhi, India: Ministry of Statistics and Programme Implementation.
Ministry of Statistics and Programme Implementation, Government of India. India State Series: Gross State Domestic Product at Factor Cost by Industry of Origin Tables 1993-94 to 2004-05. New Delhi, India: Ministry of Statistics and Programme Implementation.
Ministry of Statistics and Programme Implementation, Government of India. India State Series: Gross State Domestic Product at Factor Cost by Industry of Origin Tables 1999-2000 to 2009-10. New Delhi, India: Ministry of Statistics and Programme Implementation.
Ministry of Statistics and Programme Implementation, Government of India. India State Series: Gross State Domestic Product at Factor Cost by Industry of Origin Tables 2004-05 to 2013-14. New Delhi, India: Ministry of Statistics and Programme Implementation.
Ministry of Statistics and Programme Implementation, Government of India. Participation & Expenditure in Education Survey, National Sample Survey Round 64, July 2007- June 2008. New Delhi, India: Ministry of Statistics and Programme Implementation.
Ministry of Statistics and Programme Implementation, Government of India. Participation in Education Survey, National Sample Survey Round 52, July 1995-June 1996. New Delhi, India: Ministry of Statistics and Programme Implementation.
Ministry of Statistics and Programme Implementation, Government of India. Social Consumption Survey on Health and Education, National Sample Survey Round 71, January 2014 - June 2014. New Delhi, India: Ministry of Statistics and Programme Implementation.
Ministry of Statistics and Programme Implementation, Government of India. Survey on Healthcare, National Sample Survey Round 52, July 1995-June 1996. New Delhi, India: Ministry of Statistics and Programme Implementation.
Ministry of Statistics and Programme Implementation, Government of India. Survey on Literacy and Culture, National Sample Survey Round 47, July - December 1991. New Delhi, India: Ministry of Statistics and Programme Implementation.
Ministry of Statistics and Programme Implementation, Government of India. Survey on Morbidity and Healthcare, National Sample Survey Round 60, January-June 2004. New Delhi, India: Ministry of Statistics and Programme Implementation.
Ministry of Statistics and Programme Implementation, Government of India. Survey on Participation in Education, National Sample Survey Round 42, July 1986- June 1987. New Delhi, India: Ministry of Statistics and Programme Implementation.
Ministry of Statistics and Programme Implementation, Government of India. Urban Slums Survey, National Sample Survey Round 58, July - December 2002. New Delhi, India: Ministry of Statistics and Programme Implementation.
Ministry of Statistics and Programme Implementation, Government of India. Urban Slums Survey, National Sample Survey Round 65, July 2008- June 2009. New Delhi, India: Ministry of Statistics and Programme Implementation.

Table 3: GBD 2016 India data inputs
Ministry of Statistics and Programme Implementation, Government of India. Urban Slums Survey, National Sample Survey Round 69, July - December 2012. New Delhi, India: Ministry of Statistics and Programme Implementation.
Ministry of Women and Child Development, Government of India, United Nations Children's Fund, Save the Children, Prayas Juvenile Aid Centre Society. India National Study on Child Abuse 2007. New Delhi, India: Ministry of Women and Child Development; 2007.
Ministry of Women and Child Development, Government of India, United Nations Children's Fund. India Rapid Survey on Children 2013-2014. New Delhi, India: Ministry of Women and Child Development.
Minz S, Balraj V, Lalitha MK, Murali N, Cherian T, Manoharan G, Kadirvan S, Joseph A, Steinhoff MC. Incidence of Haemophilus influenzae type b meningitis in India. Indian J Med Res. 2008; 128(1): 57-64.
Mir M, Newcombe R. The relationship of dietary salt and blood pressure in three farming communities in Kashmir. J Hum Hypertens. 1988; 2(4): 241-6.
Mir MA, Mir F, Khosla T, Newcombe R. The relationship of salt intake and arterial blood pressure in salted-tea drinking Kashmiris. Int J Cardiol. 1986; 13(3): 279-88.
Mirkazemi R, Kar A. A population-based study on road traffic injuries in Pune City, India. Traffic Inj Prev. 2014; 15(4): 379-85.
Mirkazemi R, Kar A. Population-based approach to study unintentional injury occurrences in Pune city, India. Int J Inj Contr Saf Promot. 2014;21(1):9–16.
Mirkazemi R, Kar A. Pune Household Unintentional Injuries Study 2008-09. [Data shared for this analysis]
Mishra A, Mishra S, Jain P, Bhadoriya R, Mishra R, Lahariya C. Measles related complications and the role of vitamin A supplementation. Indian J Pediatr. 2008; 75(9): 887-90.
Mishra OP, Dhawan T, Singla PN, Dixit VK, Arya NC, Nath G. Endoscopic and histopathological evaluation of preschool children with chronic diarrhoea. J Trop Pediatr. 2001; 47(2): 77-80.
Misra M, Mittal M, Singh R, Verma A, Rai R, Chandra G, Singh D, Chauhan R, Chowdhary V, Singh R, Mall A, Khan MJ, Khare S, Yadav K. Prevalence of rheumatic heart disease in school-going children of Eastern Uttar Pradesh. Indian Heart J. 2007; 59(1): 42-3.
Misra S, Kantharia SL, Damor JR. Prevalence of goitre in 6 -12 years school-going children of Panchmahal district in Gujarat, India. Indian J Med Res. 2007; 126(5): 475-9.
Mittal M, Tandon M, Raghuvanshi RS. Iodine status of children and use of iodized salt in Tarai region of North India. J Trop Pediatr. 2000; 46(5): 300-2.
Mittal RL, Sekhon AS, Singh G, Thakral H. The prevalence of congenital orthopaedic anomalies in a rural community. Int Orthop. 1993; 17(1): 11-2.
Mittal SK, Rastogi A, Rastogi A, Kumar N, Talukdar B, Kar P. Seroprevalence of hepatitis A in children--implications for hepatitis A vaccine. Trop Gastroenterol. 1998; 19(3): 120-1.
Mohamed S, Janakiram C. Periodontal status among tobacco users in Karnataka, India. Indian J Public Health. 2013; 57(2): 105-8.
Mohan D, Chopra A, Sethi H. A rapid assessment study on prevalence of substance abuse disorders in metropolis Delhi. Indian J Med Res. 2001; 114: 107-14.

Table 3: GBD 2016 India data inputs
Mohan D, Chopra A, Sethi H. Incidence estimates of substance use disorders in a cohort from Delhi, India. Indian J Med Res. 2002; 115: 128-35
Mohan D, Tsimhoni O, Sivak M, Flannagan MJ. Road safety in India: Challenges and opportunities. Ann Arbor, United States: University of Michigan Transportation Research Institute; 2009. Report No. UMTRI-2009-1; 1-57.
Mohan V, Deepa M, Anjana RM, Lanthorn H, Deepa R. Incidence of diabetes and pre-diabetes in a selected urban south Indian population (CUPS - 19). J Assoc Physicians India. 2008; 56: 152-7.
Mohan V, Deepa M, Farooq S, Datta M, Deepa R. Prevalence, awareness and control of hypertension in Chennai--The Chennai Urban Rural Epidemiology Study (CURES-52). J Assoc Physicians India. 2007; 55: 326-32.
Mohan V, Mathur P, Deepa R, Deepa M, Shukla DK, Menon GR, Anand K, Desai NG, Joshi PP, Mahanta J, Thankappan KR, Shah B. Urban rural differences in prevalence of self-reported diabetes in India--the WHO-ICMR Indian NCD risk factor surveillance. Diabetes Res Clin Pract. 2008; 80(1): 159-68.
Mohan V, Vijayaprabha R, Rema M. Vascular complications in long-term south Indian NIDDM of over 25 years' duration. Diabetes Res Clin Pract. 1996; 31(1-3): 133–40.
Mohan VR, Sharma S, Ramanujam K, Babji S, Koshy B, Bondu JD, John SM, Kang G. Effects of elevated blood lead levels in preschool children in urban Vellore. Indian Pediatr. 2014; 51(8): 621-5.
Mohanan PP, Mathew R, Harikrishnan S, Krishnan MN, Zachariah G, Joseph J, Eapen K, Abraham M, Menon J, Thomas M, Jacob S, Huffman MD, Prabhakaran D, Kerala ACS Registry Investigators. Presentation, management, and outcomes of 25 748 acute coronary syndrome admissions in Kerala, India: results from the Kerala ACS Registry. Eur Heart J. 2013; 34(2): 121–9.
Mohanty D, Colah R B, Gorakshakar A C, Patel R Z, Matsre D C, Mahanta J, Sharma S K, Chaudhari U, Ghosh M, Das S, Britt R P, Singh S, Ross C, Jagannathan L, Kaul R, Shukla D K, Muthuswamy V. Prevalence of ß-thalassemia and other haemoglobinopathies in six cities in India: a multicentre study. J Community Genet. 2013; 4(1): 33–42.
Mohanty MK, Panigrahi MK, Mohanty S, Das SK. Victimiologic study of female homicide. Leg Med (Tokyo). 2004; 6(3): 151-6.
Mohapatra PK, Narain K, Prakash A, Bhattacharyya DR, Mahanta J. Risk factors of malaria in the fringes of an evergreen monsoon forest of Arunachal Pradesh. Natl Med J India. 2001; 14(3): 139-42.
Mohapatra PK, Prakash A, Bhattacharyya DR, Mahanta J. Epidemiological importance of younger age group during malaria epidemic in PHC Tamulpur, Assam. J Commun Dis. 1998; 30(4): 229-32.
Montgomery AL, Ram U, Kumar R, Jha P, Bhutta Z. Maternal Mortality in India: Causes and Healthcare Service Use Based on a Nationally Representative Survey. PLoS One. 2014; 9(1): e83331.
Mony PK, Varghese B, Thomas T. Estimation of perinatal mortality rate for institutional births in Rajasthan state, India, using capture-recapture technique. BMJ Open. 2015; 5(3): e005966.
Moorthy D, Patro BK, Das BC, Sankar R, Karmakar MG, Pandav CS. Tracking progress towards sustainable elimination of iodine deficiency disorders in Orissa. Indian J Public Health. 2007; 51(4): 211-5.
Morgan C, John S, Esan O, Hibben M, Patel V, Weiss HA, Murray R, Hutchinson G, Gureje O, Thara R, Cohen A. The incidence of psychoses in diverse settings, INTREPID (2): a feasibility study in India, Nigeria, and Trinidad. Psychol Med. 2016; 46(9): 1923-33.
Muir CS, Waterhouse J, Mack T, Powell J, Whelan SL, eds. Cancer Incidence in Five Continents, Vol. V. International Agency for Research on Cancer (IARC) Scientific Publications, No. 88. Lyon, France: IARC; 1987.
Mukherjee B, Dutta A, Roychoudhury S, Ray MR. Chronic inhalation of biomass smoke is associated with DNA damage in airway cells: involvement of particulate pollutants and benzene. Journal of Applied Toxicology. 2013; 33(4): 281-289.

Table 3: GBD 2016 India data inputs
Mukherjee P, Ramamurthy T, Bhattacharya MK, Rajendran K, Mukhopadhyay AK. Campylobacter jejuni in hospitalized patients with diarrhea, Kolkata, India. Emerg Infect Dis. 2013; 19(7): 1155–6.
Mukhopadhyay AK, Hati AK, Dey P. Malariogenic situations in areas of Aiodhya hills of the district Purulia West Bengal and its present status. Indian J Public Health. 2001; 45(1): 31-2.
Mukhopadhyay AK, Patnaik SK, Babu PS. Status of lymphatic filariasis in parts of east Godavari district of Andhra Pradesh, India. J Vector Borne Dis. 2007; 44(1): 72-4.
Mukhopadhyay AK, Patnaik SK. Effect of mass drug administration programme on microfilaria carriers in East Godavari district of Andhra Pradesh. J Vector Borne Dis. 2007; 44(4): 277-80.
Mukhopadhyay AK. Lymphatic filariasis in Andhra Pradesh Paper Mill Colony, Rajahmundry, India after nine rounds of MDA programme. J Vector Borne Dis. 2010; 47(1): 55-7.
Muninarayana C, Balachandra G, Hiremath SG, Iyengar K, Anil NS. Prevalence and awareness regarding diabetes mellitus in rural Tamaka, Kolar. Int J Diabetes Dev Ctries. 2010; 30(1): 18-21.
Munro HL, Pradeep BS, Jayachandran AA, Lowndes CM, Mahapatra B, Ramesh BM, Washington R, Jagannathan L, Mendonca K, Moses S, Blanchard JF, Alary M. Prevalence and determinants of HIV and sexually transmitted infections in a general population-based sample in Mysore district, Karnataka state, southern India. AIDS. 2008; 22(Suppl 5): S117-125.
Murhekar KM, Murhekar MV, Mukherjee MB, Gorakshakar AC, Surve R, Wadia M, Phanasgaonkar S, Shridevi S, Colah RB, Mohanty D. Red cell genetic abnormalities, beta-globin gene haplotypes, and APOB polymorphism in the Great Andamanese, a primitive Negrito tribe of Andaman and Nicobar Islands, India. Hum Biol. 2001; 73(5): 739-44.
Murhekar MV, Ahmad M, Shukla H, Abhishek K, Perry RT, Bose AS, Shimpi R, Kumar A, Kaliaperumal K, Sethi R, Selvaraj V, Kamaraj P, Routray S, Das VN, Menabde N, Bahl S. Measles case fatality rate in Bihar, India, 2011-12. PLoS One. 2014; 9(5): e96668.
Murhekar MV, Hutin YJ, Ramakrishnan R, Ramachandran V, Biswas AK, Das PK, Gupta SN, Maji D, Martolia HCS, Mohan A, Gupte MD. The heterogeneity of measles epidemiology in India: implications for improving control measures. J Infect Dis. 2011; 204(Suppl 1): S421-426.
Murhekar MV, Murhekar KM, Sehgal SC. Age-specific prevalence of hepatitis B infection among the Karen in the Andaman and Nicobar Islands, India. Trop Doct. 2004; 34(2): 117-8.
Murhekar MV, Murhekar KM, Sehgal SC. Seroepidemiology of hepatitis B infection among tribal school children in Andaman and Nicobar Islands, India. Ann Trop Paediatr. 2004; 24(1): 85-8.
Murthy AK, Pramila M, Ranganath S. Prevalence of clinical consequences of untreated dental caries and its relation to dental fear among 12-15-year-old schoolchildren in Bangalore city, India. Eur Arch Paediatr Dent. 2014; 15(1): 45-9.
Murthy GV, Gupta S, Ellwein LB, Munoz SR, Bachani D, Dada VK. A population-based eye survey of older adults in a rural district of Rajasthan: I. Central vision impairment, blindness, and cataract surgery. Ophthalmology. 2001; 108(4): 679-85.
Murthy GVS, Gupta SK, Bachani D, Jose R, John N. Current estimates of blindness in India. [Data shared for this analysis]
Murthy GVS, Gupta SK, Bachani D, Jose R, John N. Current estimates of blindness in India. Br J Ophthalmol. 2005; 89(3): 257-60.
Murthy GVS. Rapid Assessment of Avoidable Blindness in India, 1990-2001. [Data shared for this analysis]
Murthy J, Vijay S, Ravi Raju C, Thomas J. Acute symptomatic seizures associated with neurocysticercoses: A community based prevalence study and comprehensive rural epilepsy study in South India (CRESSI). Neurology Asia. 2004; 9(Suppl 1): 86.

Table 3: GBD 2016 India data inputs
Murty US, Praveen B, Kumar DVRS, Sriram K, Rao KM, Sai KSK. A baseline study of rural Bancroftian filariasis in southern India. Southeast Asian J Trop Med Public Health. 2004; 35(3): 583-6.
Mutheneni SR, Upadhyayula SM, Kumaraswamy S, Kadiri MR, Nagalla B. Impact of socioeconomic factors on the prevalence of lymphatic filariasis in Andhra Pradesh, India. J Public Health. 2015; 23(4): 231-40.
Muwonge R, Ramadas K, Sankila R, Thara S, Thomas G, Vinoda J, Sankaranarayanan R. Role of tobacco smoking, chewing and alcohol drinking in the risk of oral cancer in Trivandrum, India: a nested case-control design using incident cancer cases. Oral Oncol. 2008; 44(5): 446-54.
Nadiger HA, Krishnamachari KA, Naidu AN, Rao BS, Srikantia SG. The use of common salt (sodium chloride) fortified with iron to control anaemia: results of a preliminary study. Br J Nutr. 1980; 43(1): 45-51.
Nadkarni A, Dean K, Weiss HA, Patel V. Prevalence and Correlates of Perpetration of Violence Among Young People?: A Population-Based Survey From Goa, India. Asia Pac J Public Health. 2011; 27(2): NP2512–20
Nadkarni A, Weiss HA, Naik A, Bhat B, Patel V. The six-year outcome of alcohol use disorders in men: A population based study from India. Drug Alcohol Depend. 2016; 162: 107–15.
Nagamani K, Pavuluri PRR, Gyaneshwari M, Prasanthi K, Rao MIS, Saxena NK. Molecular characterisation of Cryptosporidium: an emerging parasite. Indian J Med Microbiol. 2007; 25(2): 133-6.
Nagar R, Raman R. Diversity of sickle cell trait in Jharkhand state in India: Is it the zone of contact between two geographically and ethnically distinct populations in India?. J Biosci. 2015; 40(3): 539-47.
Nagel G, Weinmayr G, Flohr C, Kleiner A, Strachan DP. Association of pertussis and measles infections and immunizations with asthma and allergic sensitization in ISAAC Phase Two. Pediatr Allergy Immunol. 2012; 23(8): 737-46.
Nahar R, Kotecha U, Puri RD, Pandey RM, Verma IC. Survival analysis of Down syndrome cohort in a tertiary health care center in India. Indian J Pediatr. 2013; 80(2): 118-23.
Nair GB, Ramamurthy T, Bhattacharya MK, Krishnan T, Ganguly S, Saha DR, Rajendran K, Manna B, Ghosh M, Okamoto K, Takeda Y. Emerging trends in the etiology of enteric pathogens as evidenced from an active surveillance of hospitalized diarrhoeal patients in Kolkata, India. Gut Pathog. 2010; 2(1): 4.
Nair MKC, George B, Jeyaseelan L. Pyritinol for post asphyxial encephalopathy in term babies – a randomized double-blind controlled trial. Indian Pediatr. 2009; 46(Suppl): S37-42.
Nair MKC, Russell PSS, Mammen P, Abhiram Chandran R, Krishnan R, Nazeema S, Chembagam N, Peter D. ADad 3: the epidemiology of Anxiety Disorders among adolescents in a rural community population in India. Indian J Pediatr. 2013; 80 Suppl 2: S144-148.
Naish S, McCarthy J, Williams GM. Prevalence, intensity and risk factors for soil-transmitted helminth infection in a South Indian fishing village. Acta Trop. 2004; 91(2): 177–87.
Nallam NR, Paul I, Gnanamani G. Anemia and hypoalbuminia as an adjunct to soil-transmitted helminthiasis among slum school children in Visakhapatnam, South India. Asia Pac J Clin Nutr. 1998; 7(2): 164–9.
Nandi J, Bhawalkar V, Mody H, Elavia A, Desai PK, Banerjee K. Detection of HIV-1, HBV and HCV antibodies in blood donors from Surat, western India. Vox Sang. 1994; 67(4): 406-7.
Nangia V, Jonas JB, Gupta R, Khare A, Sinha A. Visual impairment and blindness in rural central India: the Central India Eye and Medical Study. Acta Ophthalmol. 2013; 91(5.0): 483-6.
Nangia V, Jonas JB, Kulkarni M, Matin A. Prevalence of age-related macular degeneration in rural central India: the Central India Eye and Medical Study. Retina. 2011; 31(6): 1179-85.

Table 3: GBD 2016 India data inputs
Nangia V, Jonas JB, Sinha A, Bhojwani K, Matin A. Visual impairment among school children in urban Central India: The Central India Children Eye Study. Acta Ophthalmol (Copenh). 2012; 90(4): e329-e331.
Nangia V, Jonas JB, Sinha A, Matin A, Kulkarni M, Panda-Jonas S. Ocular axial length and its associations in an adult population of Central Rural India. The Central India Eye and Medical Study. Ophthalmology 2010;117(7):1360-6.
Nangia V, Jonas JB, Sinha A, Matin A, Kulkarni M, Panda-Jonas S. Ocular axial length and its associations in an adult population of Central Rural India: The Central India Eye and Medical Study. Ophthalmology 2010;117(7):1360-6. [Data shared for this analysis]
Narain J, Khare S, Rana S, Banerjee K. Epidemic measles in an isolated unvaccinated population, India. Int J Epidemiol. 1989; 18(4): 952-8.
Narain K, Medhi GK, Rajguru SK, Mahanta J. Cure and reinfection patterns of geohelminthic infections after treatment in communities inhabiting the tropical rainforest of Assam, India. Southeast Asian J Trop Med Public Health. 2004; 35(3): 512–7.
Narain K, Rajguru SK, Mahanta J. Prevalence of Trichuris trichiura in relation to socio-economic & behavioural determinants of exposure to infection in rural Assam. Indian J Med Res. 2000; 112: 140–6.
Narain R, Anantharaman DS, Diwakara AM. Prevalence of nonspecific tuberculin sensitivity in certain parts of India. Bull World Health Organ. 1974; 51(3): 273.
Narain R, Geser A, Jambunathan MV, Subramanian M. Some aspects of a tuberculosis prevalence survey in a South Indian district. Bull World Health Organ. 1963; 29(5): 641-64.
Narain R, Geser A, Jambunathan MV, Subramanian M. Tuberculosis prevalence survey in Tumkur district. Indian J Tuberc. 1963; 10(3): 85–116.
Narang P, Mendiratta DK, Tyagi NK, Jajoo UN, Tayade AT, Parihar PH, et al. Prevalence of pulmonary tuberculosis in Wardha district of Maharashtra, Central India. J Epidemiol Glob Health. 2015 Dec ;5(4):S11–8.
Narayana P, Prasanna M, Narahari S, Guruprasad A. Prevalence of asthma in school children in rural India. Ann Thorac Med. 2010;5(2):118.
Nasrin D, Wu Y, Blackwelder WC, Farag TH, Saha D, Sow SO, Alonso PL, Breiman RF, Sur D, Faruque ASG, Zaidi AKM, Biswas K, Van Eijk AM, Walker DG, Levine MM, Kotloff KL. Health care seeking for childhood diarrhea in developing countries: evidence from seven sites in Africa and Asia. Am J Trop Med Hyg. 2013; 89(1 Suppl): 3-12.
Nath G, Choudhury A, Shukla BN, Singh TB, Reddy DC. Significance of Cryptosporidium in acute diarrhoea in North-Eastern India. J Med Microbiol. 1999; 48(6): 523-6.
Nath J, Banyal N, Gautam DS, Ghosh SK, Singha B, Paul J. Systematic detection and association of Entamoeba species in stool samples from selected sites in India. Epidemiol Infect. 2015; 143(1): 108-19.
National Aeronautics and Space Administration (NASA), Center for International Earth Science Information Network, Columbia University. Gridded Population of the World, Version 4 (GPWv4): Population Count Adjusted to Match 2015 Revision of UN WPP Country Totals. Palisades, United States: NASA SEDAC; 2016. Available from: http://sedac.ciesin.columbia.edu/data/set/gpw-v4-population-count-adjusted-to-2015-unwpp-country-totals.
National Aeronautics and Space Administration. Aerosol Optical Depth data from Aerosol Robotic Network. Available from: https://aeronet.gsfc.nasa.gov/new_web/data.html
National Aeronautics and Space Administration. Aerosol Optical Depth data from Moderate Resolution Imaging Spectroradiometer-Dark Target Aerosol Retrieval Algorithm. Avaialble from: https://darktarget.gsfc.nasa.gov/algorithm

Table 3: GBD 2016 India data inputs
National Aeronautics and Space Administration. Aerosol Optical Depth data from Moderate Resolution Imaging Spectroradiometer-Multi-Angle Implementation of Atmospheric Correction. Available from: https://ladsweb.modaps.eosdis.nasa.gov/api/v1/productGroupPage/name=maiac
National Aeronautics and Space Administration. Aerosol Optical Depth data from Moderate Resolution Imaging Spectroradiometer. Available from: https://modis.gsfc.nasa.gov/data/
National Aeronautics and Space Administration. SeaWiFS Deep Blue Aerosol Optical Depth and Angstrom Exponent Monthly Level 3 Data Gridded at 1.0 Degrees. Avaialble from: https://data.nasa.gov/Earth-Science/SeaWiFS-Deep-Blue-Aerosol-Optical-Depth-and-Angstr/h7mm-kenx
National AIDS Control Organization (NACO), Ministry of Health and Family Welfare, Government of India. HIV Sentinel Surveillance data 2014-2015: HIV Prevalence among Antenatal Clinic Attendees by State. New Delhi, India: NACO.
National AIDS Control Organization (NACO), Ministry of Health and Family Welfare, Government of India. HIV Sentinel Surveillance: HIV Adult Prevalence data, Andhra Pradesh, 1998-2013. New Delhi, India: NACO.
National AIDS Control Organization (NACO), Ministry of Health and Family Welfare, Government of India. HIV Sentinel Surveillance: HIV Adult Prevalence data, Chhattisgarh, 2001-2013. New Delhi, India: NACO.
National AIDS Control Organization (NACO), Ministry of Health and Family Welfare, Government of India. HIV Sentinel Surveillance: HIV Adult Prevalence data, Goa, 1998-2013. New Delhi, India: NACO.
National AIDS Control Organization (NACO), Ministry of Health and Family Welfare, Government of India. HIV Sentinel Surveillance: HIV Adult Prevalence data, Karnataka, 1998-2013. New Delhi, India: NACO.
National AIDS Control Organization (NACO), Ministry of Health and Family Welfare, Government of India. Antiretroviral Therapy Coverage Data by State 2004. New Delhi, India: NACO.
National AIDS Control Organization (NACO), Ministry of Health and Family Welfare, Government of India. Antiretroviral Therapy Coverage Data by State 2015-2016. New Delhi, India: NACO.
National AIDS Control Organization (NACO), Ministry of Health and Family Welfare, Government of India. HIV Sentinal Surveillance Report 2014-2015. New Delhi, India: NACO.
National AIDS Control Organization (NACO), Ministry of Health and Family Welfare, Government of India. HIV Sentinel Surveillance: HIV Adult Prevalence data, Arunachal Pradesh, 1999-2013. New Delhi, India: NACO.
National AIDS Control Organization (NACO), Ministry of Health and Family Welfare, Government of India. HIV Sentinel Surveillance: HIV Adult Prevalence data, Assam, 1998-2013. New Delhi, India: NACO.
National AIDS Control Organization (NACO), Ministry of Health and Family Welfare, Government of India. HIV Sentinel Surveillance: HIV Adult Prevalence data, Bihar, 1998-2013. New Delhi, India: NACO.
National AIDS Control Organization (NACO), Ministry of Health and Family Welfare, Government of India. HIV Sentinel Surveillance: HIV Adult Prevalence data, Delhi, 1998-2013. New Delhi, India: NACO.
National AIDS Control Organization (NACO), Ministry of Health and Family Welfare, Government of India. HIV Sentinel Surveillance: HIV Adult Prevalence data, Gujarat, 1998-2013. New Delhi, India: NACO.
National AIDS Control Organization (NACO), Ministry of Health and Family Welfare, Government of India. HIV Sentinel Surveillance: HIV Adult Prevalence data, Haryana, 1998-2013. New Delhi, India: NACO.
National AIDS Control Organization (NACO), Ministry of Health and Family Welfare, Government of India. HIV Sentinel Surveillance: HIV Adult Prevalence data, Himachal Pradesh, 1998-2013. New Delhi, India: NACO.

Table 3: GBD 2016 India data inputs
National AIDS Control Organization (NACO), Ministry of Health and Family Welfare, Government of India. HIV Sentinel Surveillance: HIV Adult Prevalence data, Jammu and Kashmir, 1998-2013. New Delhi, India: NACO.
National AIDS Control Organization (NACO), Ministry of Health and Family Welfare, Government of India. HIV Sentinel Surveillance: HIV Adult Prevalence data, Jharkhand, 2001-2013. New Delhi, India: NACO.
National AIDS Control Organization (NACO), Ministry of Health and Family Welfare, Government of India. HIV Sentinel Surveillance: HIV Adult Prevalence data, Kerala, 1998-2013. New Delhi, India: NACO.
National AIDS Control Organization (NACO), Ministry of Health and Family Welfare, Government of India. HIV Sentinel Surveillance: HIV Adult Prevalence data, Madhya Pradesh 1998-2013. New Delhi, India: NACO.
National AIDS Control Organization (NACO), Ministry of Health and Family Welfare, Government of India. HIV Sentinel Surveillance: HIV Adult Prevalence data, Maharashtra, 1998-2013. New Delhi, India: NACO.
National AIDS Control Organization (NACO), Ministry of Health and Family Welfare, Government of India. HIV Sentinel Surveillance: HIV Adult Prevalence data, Manipur, 1998-2013. New Delhi, India: NACO.
National AIDS Control Organization (NACO), Ministry of Health and Family Welfare, Government of India. HIV Sentinel Surveillance: HIV Adult Prevalence data, Meghalaya, 1998-2013. New Delhi, India: NACO.
National AIDS Control Organization (NACO), Ministry of Health and Family Welfare, Government of India. HIV Sentinel Surveillance: HIV Adult Prevalence data, Mizoram, 1998-2013. New Delhi, India: NACO.
National AIDS Control Organization (NACO), Ministry of Health and Family Welfare, Government of India. HIV Sentinel Surveillance: HIV Adult Prevalence data, Nagaland, 1999-2013. New Delhi, India: NACO.
National AIDS Control Organization (NACO), Ministry of Health and Family Welfare, Government of India. HIV Sentinel Surveillance: HIV Adult Prevalence data, Odisha, 1998-2013. New Delhi, India: NACO.
National AIDS Control Organization (NACO), Ministry of Health and Family Welfare, Government of India. HIV Sentinel Surveillance: HIV Adult Prevalence data, Punjab, 1998-2013. New Delhi, India: NACO.
National AIDS Control Organization (NACO), Ministry of Health and Family Welfare, Government of India. HIV Sentinel Surveillance: HIV Adult Prevalence data, Rajasthan, 1998-2013. New Delhi, India: NACO.
National AIDS Control Organization (NACO), Ministry of Health and Family Welfare, Government of India. HIV Sentinel Surveillance: HIV Adult Prevalence data, Sikkim, 1998-2013. New Delhi, India: NACO.
National AIDS Control Organization (NACO), Ministry of Health and Family Welfare, Government of India. HIV Sentinel Surveillance: HIV Adult Prevalence data, Tamil Nadu, 1998-2013. New Delhi, India: NACO.
National AIDS Control Organization (NACO), Ministry of Health and Family Welfare, Government of India. HIV Sentinel Surveillance: HIV Adult Prevalence data, Tripura, 1999-2013. New Delhi, India: NACO.
National AIDS Control Organization (NACO), Ministry of Health and Family Welfare, Government of India. HIV Sentinel Surveillance: HIV Adult Prevalence data, Uttar Pradesh, 1998-2013. New Delhi, India: NACO.
National AIDS Control Organization (NACO), Ministry of Health and Family Welfare, Government of India. HIV Sentinel Surveillance: HIV Adult Prevalence data, Uttarkhand, 2001-2013. New Delhi, India: NACO.
National AIDS Control Organization (NACO), Ministry of Health and Family Welfare, Government of India. HIV Sentinel Surveillance: HIV Adult Prevalence data, West Bengal, 1998-2013. New Delhi, India: NACO.
National AIDS Control Organization (NACO), Ministry of Health and Family Welfare, Government of India. National Integrated Biological and Behavioural Surveillance (IBBS) data, Andhra Pradesh, India 2014-15. New Delhi, India: NACO.

Table 3: GBD 2016 India data inputs
National AIDS Control Organization (NACO), Ministry of Health and Family Welfare, Government of India. National Integrated Biological and Behavioural Surveillance (IBBS) data, Haryana, India 2014-15. New Delhi, India: NACO.
National AIDS Control Organization (NACO), Ministry of Health and Family Welfare, Government of India. National Integrated Biological and Behavioural Surveillance (IBBS) data, Karnataka, India 2014-15. New Delhi, India: NACO.
National AIDS Control Organization (NACO), Ministry of Health and Family Welfare, Government of India. National Integrated Biological and Behavioural Surveillance (IBBS) data, Maharashtra, India 2014-15. New Delhi, India: NACO.
National AIDS Control Organization (NACO), Ministry of Health and Family Welfare, Government of India. National Integrated Biological and Behavioural Surveillance (IBBS) data, Manipur, India 2014-15. New Delhi, India: NACO.
National AIDS Control Organization (NACO), Ministry of Health and Family Welfare, Government of India. National Integrated Biological and Behavioural Surveillance (IBBS) data, Mizoram, India 2014-15. New Delhi, India: NACO.
National AIDS Control Organization (NACO), Ministry of Health and Family Welfare, Government of India. National Integrated Biological and Behavioural Surveillance (IBBS) data, Nagaland, India 2014-15. New Delhi, India: NACO.
National Cancer Registry Programme, National Centre for Disease Informatics and Research, Indian Council of Medical Research (ICMR). District Cancer Registry Kollam, Kerala 1990-2014. New Delhi, India: ICMR; 2016.
National Cancer Registry Programme, National Centre for Disease Informatics and Research, Indian Council of Medical Research (ICMR). Barshi Expanded Registry (Osmanabad & Beed District) Data 2007-2012. New Delhi, India: ICMR; 2016.
National Cancer Registry Programme, National Centre for Disease Informatics and Research, Indian Council of Medical Research (ICMR). India Consolidated Report of Population Based Cancer Registries 2004-2005. New Delhi, India: ICMR; 2008.
National Cancer Registry Programme, National Centre for Disease Informatics and Research, Indian Council of Medical Research (ICMR). India Three-Year Report of Population Based Cancer Registries 2006-2008. New Delhi, India: ICMR; 2010.
National Cancer Registry Programme, National Centre for Disease Informatics and Research, Indian Council of Medical Research (ICMR). India Three-year Report of Population Based Cancer Registries 2009-2011. New Delhi, India: ICMR; 2013.
National Cancer Registry Programme, National Centre for Disease Informatics and Research, Indian Council of Medical Research (ICMR). India Three-year Report of Population Based Cancer Registries 2012-2014. New Delhi, India: ICMR; 2016.
National Cancer Registry Programme, National Centre for Disease Informatics and Research, Indian Council of Medical Research (ICMR). North East Population Based Cancer Registries Report 2005-2006. New Delhi, India: ICMR; 2008.
National Cancer Registry Programme, National Centre for Disease Informatics and Research, Indian Council of Medical Research (ICMR). Population Based Cancer Registry of Bhopal 1986-2013. New Delhi, India: ICMR; 2016.
National Cancer Registry Programme, National Centre for Disease Informatics and Research, Indian Council of Medical Research (ICMR). Population Based Cancer Registry, Ahmedabad Urban Agglomeration Area 2007-2013. New Delhi, India: ICMR; 2016.
National Cancer Registry Programme, National Centre for Disease Informatics and Research, Indian Council of Medical Research (ICMR). Population Based Cancer Registry, Arunachal West 2011-2014. New Delhi, India: ICMR; 2016.
National Cancer Registry Programme, National Centre for Disease Informatics and Research, Indian Council of Medical Research (ICMR). Population Based Cancer Registry, Assam Medical College & Hospital, Dibrugarh 2003-2014. New Delhi, India: ICMR; 2016.
National Cancer Registry Programme, National Centre for Disease Informatics and Research, Indian Council of Medical Research (ICMR). Population Based Cancer Registry, Aurangabad 1982-2014. New Delhi, India: ICMR; 2016.

Table 3: GBD 2016 India data inputs
National Cancer Registry Programme, National Centre for Disease Informatics and Research, Indian Council of Medical Research (ICMR). Population Based Cancer Registry, Bangalore 1982-2012. New Delhi, India: ICMR; 2016.
National Cancer Registry Programme, National Centre for Disease Informatics and Research, Indian Council of Medical Research (ICMR). Population Based Cancer Registry, Cachar District 2007-2014. New Delhi, India: ICMR; 2016.
National Cancer Registry Programme, National Centre for Disease Informatics and Research, Indian Council of Medical Research (ICMR). Population Based Cancer Registry, Chennai 1982-2013. New Delhi, India: ICMR; 2016.
National Cancer Registry Programme, National Centre for Disease Informatics and Research, Indian Council of Medical Research (ICMR). Population Based Cancer Registry, Delhi 1986-2012. New Delhi, India: ICMR; 2016.
National Cancer Registry Programme, National Centre for Disease Informatics and Research, Indian Council of Medical Research (ICMR). Population Based Cancer Registry, Guwahati Kamrup Urban District 2003-2014. New Delhi, India: ICMR; 2016.
National Cancer Registry Programme, National Centre for Disease Informatics and Research, Indian Council of Medical Research (ICMR). Population Based Cancer Registry, Kolkata 2005-2012. New Delhi, India: ICMR; 2016.
National Cancer Registry Programme, National Centre for Disease Informatics and Research, Indian Council of Medical Research (ICMR). Population Based Cancer Registry, Manipur State 2003-2014. New Delhi, India: ICMR; 2016.
National Cancer Registry Programme, National Centre for Disease Informatics and Research, Indian Council of Medical Research (ICMR). Population Based Cancer Registry, Meghalaya 2009-2014. New Delhi, India: ICMR; 2016.
National Cancer Registry Programme, National Centre for Disease Informatics and Research, Indian Council of Medical Research (ICMR). Population Based Cancer Registry, Mizoram State 2003-2014. New Delhi, India: ICMR; 2016.
National Cancer Registry Programme, National Centre for Disease Informatics and Research, Indian Council of Medical Research (ICMR). Population Based Cancer Registry, Mumbai 1982-2012. New Delhi, India: ICMR; 2016.
National Cancer Registry Programme, National Centre for Disease Informatics and Research, Indian Council of Medical Research (ICMR). Population Based Cancer Registry, Nagaland 2009-2014. New Delhi, India: ICMR; 2016.
National Cancer Registry Programme, National Centre for Disease Informatics and Research, Indian Council of Medical Research (ICMR). Population Based Cancer Registry, Nagpur 1982-2013. New Delhi, India: ICMR; 2016.
National Cancer Registry Programme, National Centre for Disease Informatics and Research, Indian Council of Medical Research (ICMR). Population Based Cancer Registry, Pasighat 2011-2014. New Delhi, India: ICMR; 2016.
National Cancer Registry Programme, National Centre for Disease Informatics and Research, Indian Council of Medical Research (ICMR). Population Based Cancer Registry, Patiala District 2011-2014. New Delhi, India: ICMR; 2016.
National Cancer Registry Programme, National Centre for Disease Informatics and Research, Indian Council of Medical Research (ICMR). Population Based Cancer Registry, Pune 1982-2013. New Delhi, India: ICMR; 2016.
National Cancer Registry Programme, National Centre for Disease Informatics and Research, Indian Council of Medical Research (ICMR). Population Based Cancer Registry, Sikkim State 2003-2014. New Delhi, India: ICMR; 2016.
National Cancer Registry Programme, National Centre for Disease Informatics and Research, Indian Council of Medical Research (ICMR). Population Based Cancer Registry, Thiruvananthapuram 2006-2014. New Delhi, India: ICMR; 2016.
National Cancer Registry Programme, National Centre for Disease Informatics and Research, Indian Council of Medical Research (ICMR). Population Based Cancer Registry, Tripura 2009-2014. New Delhi, India: ICMR; 2016.

Table 3: GBD 2016 India data inputs
National Cancer Registry Programme, National Centre for Disease Informatics and Research, Indian Council of Medical Research (ICMR). Population Based Cancer Registry, Wardha 2010-2014. New Delhi, India: ICMR; 2016.
National Cancer Registry Programme, National Centre for Disease Informatics and Research, Indian Council of Medical Research (ICMR). Population Based Rural Cancer Registry, Barshi (Barshi, Paranda and Bhum) 1987-2014. New Delhi, India: ICMR; 2016.
National Cancer Registry Programme, National Centre for Disease Informatics and Research, Indian Council of Medical Research (ICMR). Rural Cancer Registry, Ahmedabad District 2004-2014. New Delhi, India: ICMR; 2016.
National Centre for Disease Control (NCDC), Directorate General of Health Services, Ministry of Health and Family Welfare, Government of India. District-wise Guinea worm cases for Andhra Pradesh and Telangana 1984-1998. [Data shared for this analysis]
National Centre for Disease Control (NCDC), Directorate General of Health Services, Ministry of Health and Family Welfare, Government of India. Guinea Worm Eradication Programme Data. New Delhi, India: NCDC.
National Commission for Protection of Child Rights (NCPCR), Government of India, National Drug Dependence Treatment Centre, All India Institute of Medical Sciences. Report on assessment of pattern, profile and correlates of substance use among children in India 2012-2013. New Delhi, India: NCPCR; 2013.
National Geophysical Data Center, National Oceanic and Atmosphere Administration. Global Land One-kilometer Base Elevation. Boulder, United States: National Geophysical Data Center, National Oceanic and Atmosphere Administration; 1999.
National Institute for Research in Reproductive Health, Indian Council of Medical Research (ICMR). India National Institute for Research in Reproductive Health Annual Report 2007-2008. New Delhi, India: ICMR.
National Institute of Medical Statistics, Indian Council of Medical Research (ICMR), Integrated Disease Surveillance Programme. Non-Communicable Disease Risk Factors Survey Data 2007-2008. New Delhi, India: ICMR. [Data shared for this analysis]
National Institute of Medical Statistics, Indian Council of Medical Research (ICMR), Integrated Disease Surveillance Programme. Non-Communicable Disease Risk Factors Survey Report 2007-2008. New Delhi, India: ICMR.
National Institute of Mental Health and Neurosciences, World Health Organization (WHO). Report on WHO Collaborative Project on Unrecorded Consumption of Alcohol in Karnataka, India 2001-2002. Available from: http://nimhans.ac.in/cam/sites/default/files/Publications/25.pdf
National Institute of Nutrition, Indian Council of Medical Research. India rural and urban survey data of processed and non-processed foods conducted in five regions 2012-2013. [Data shared for this analysis]
National Institute of Nutrition, Indian Council of Medical Research. Urban dietary survey data 2015-2016. [Data shared for this analysis]
National Nutrition Monitoring Bureau, National Institute of Nutrition (NIN), Indian Council of Medical Research. India Linked Diet and Nutritional Status and Consumer Expenditure Survey Data 1983-1984. [Data shared for this analysis]
National Nutrition Monitoring Bureau, National Institute of Nutrition (NIN), Indian Council of Medical Research. India Linked Diet and Nutritional Status and Consumer Expenditure Survey Report 1983-1984. Hyderabad, India: NIN.
National Nutrition Monitoring Bureau, National Institute of Nutrition (NIN), Indian Council of Medical Research. India Micronutrient Deficiency Survey Data 2003-2006. [Data shared for this analysis]
National Nutrition Monitoring Bureau, National Institute of Nutrition (NIN), Indian Council of Medical Research. India Micronutrient Deficiency Survey Report 2003-2006. Hyderabad, India: NIN.
National Nutrition Monitoring Bureau, National Institute of Nutrition (NIN), Indian Council of Medical Research. India Rural First Repeat Survey of Diet and Nutritional Status Data 1988-1990. [Data shared for this analysis]

Table 3: GBD 2016 India data inputs
National Nutrition Monitoring Bureau, National Institute of Nutrition (NIN), Indian Council of Medical Research. India Rural First Repeat Survey of Diet and Nutritional Status Report 1988-1990. Hyderabad, India: NIN.
National Nutrition Monitoring Bureau, National Institute of Nutrition (NIN), Indian Council of Medical Research. India Rural Second Repeat Survey of Diet and Nutritional Status Data 1996-1997. [Data shared for this analysis]
National Nutrition Monitoring Bureau, National Institute of Nutrition (NIN), Indian Council of Medical Research. India Rural Second Repeat Survey of Diet and Nutritional Status Report 1996-1997. Hyderabad, India: NIN.
National Nutrition Monitoring Bureau, National Institute of Nutrition (NIN), Indian Council of Medical Research. India Rural Survey of Diet and Nutritional Status Data 1994-1995. [Data shared for this analysis]
National Nutrition Monitoring Bureau, National Institute of Nutrition (NIN), Indian Council of Medical Research. India Rural Survey of Diet and Nutritional Status Data 2000-2001. [Data shared for this analysis]
National Nutrition Monitoring Bureau, National Institute of Nutrition (NIN), Indian Council of Medical Research. India Rural Survey of Diet and Nutritional Status Data 2004-2006. [Data shared for this analysis]
National Nutrition Monitoring Bureau, National Institute of Nutrition (NIN), Indian Council of Medical Research. India Rural Survey of Diet and Nutritional Status Report 1994-1995. Hyderabad, India: NIN.
National Nutrition Monitoring Bureau, National Institute of Nutrition (NIN), Indian Council of Medical Research. India Rural Survey of Diet and Nutritional Status Report 2000-2001. Hyderabad, India: NIN.
National Nutrition Monitoring Bureau, National Institute of Nutrition (NIN), Indian Council of Medical Research. India Rural Survey of Diet and Nutritional Status Report 2004-2006. Hyderabad, India: NIN.
National Nutrition Monitoring Bureau, National Institute of Nutrition (NIN), Indian Council of Medical Research. India Rural Third Repeat Survey of Diet and Nutritional Status Report 2011-2012. Hyderabad, India: NIN.
National Nutrition Monitoring Bureau, National Institute of Nutrition (NIN), Indian Council of Medical Research. India Rural Third Repeat Survey of Diet and Nutritional Status Data 2011-2012. [Data shared for this analysis]
National Nutrition Monitoring Bureau, National Institute of Nutrition (NIN), Indian Council of Medical Research. India Survey of Diet and Nutritional Status Data 1981. [Data shared for this analysis]
National Nutrition Monitoring Bureau, National Institute of Nutrition (NIN), Indian Council of Medical Research. India Survey of Diet and Nutritional Status Data 1990-1992. [Data shared for this analysis]
National Nutrition Monitoring Bureau, National Institute of Nutrition (NIN), Indian Council of Medical Research. India Survey of Diet and Nutritional Status Data 1991-1992. [Data shared for this analysis]
National Nutrition Monitoring Bureau, National Institute of Nutrition (NIN), Indian Council of Medical Research. India Survey of Diet and Nutritional Status Report 1981. Hyderabad, India: NIN.
National Nutrition Monitoring Bureau, National Institute of Nutrition (NIN), Indian Council of Medical Research. India Survey of Diet and Nutritional Status Report 1990-1992. Hyderabad, India: NIN.
National Nutrition Monitoring Bureau, National Institute of Nutrition (NIN), Indian Council of Medical Research. India Survey of Diet and Nutritional Status Report 1991-1992. Hyderabad, India: NIN.
National Nutrition Monitoring Bureau, National Institute of Nutrition (NIN), Indian Council of Medical Research. India Tribal First Repeat Survey of Diet and Nutritional Status Data 1998-1999. [Data shared for this analysis]
National Nutrition Monitoring Bureau, National Institute of Nutrition (NIN), Indian Council of Medical Research. India Tribal First Repeat Survey of Diet and Nutritional Status Report 1998-1999. Hyderabad, India: NIN.

Table 3: GBD 2016 India data inputs
National Nutrition Monitoring Bureau, National Institute of Nutrition (NIN), Indian Council of Medical Research. India Tribal Second Repeat Survey of Diet and Nutritional Status Data 2007-2008. [Data shared for this analysis]
National Nutrition Monitoring Bureau, National Institute of Nutrition (NIN), Indian Council of Medical Research. India Tribal Second Repeat Survey of Diet and Nutritional Status Data 2008-2009. [Data shared for this analysis]
National Nutrition Monitoring Bureau, National Institute of Nutrition (NIN), Indian Council of Medical Research. India Tribal Second Repeat Survey of Diet and Nutritional Status Report 2007-2008. Hyderabad, India: NIN.
National Nutrition Monitoring Bureau, National Institute of Nutrition (NIN), Indian Council of Medical Research. India Tribal Second Repeat Survey of Diet and Nutritional Status Report 2008-2009. Hyderabad, India: NIN.
National Nutrition Monitoring Bureau, National Institute of Nutrition (NIN), Indian Council of Medical Research. India Urban Slums Survey of Diet and Nutritional Status Data 1993-1994. [Data shared for this analysis]
National Nutrition Monitoring Bureau, National Institute of Nutrition (NIN), Indian Council of Medical Research. India Urban Slums Survey of Diet and Nutritional Status Report 1993-1994. Hyderabad, India: NIN.
National Nutrition Monitoring Bureau, National Institute of Nutrition, Indian Council of Medical Research. India National Nutrition Monitoring Bureau Eight States Pooled Data 1991-1992. [Data shared for this analysis]
National Rural Drinking Water Programme, Ministry of Drinking water and Sanitation, Government of India. State Quality Profile For Lab Testing 2014-16. New Delhi, India: Ministry of Drinking water and Sanitation; 2017. Available from: http://indiawater.gov.in/imisreports/Reports/WaterQuality/rpt_wqm_districtProfile_S.aspx?Rep=0
National Tuberculosis Institute, Directorate General of Health Services, Second Zonal Level Tuberculin Surveys in India for Estimating the Annual Risk of Tuberculous Infection among Children 2009-2010. [Data shared for this analysis]
National Tuberculosis Institute-Bangalore, Tuberculosis Research Center-Chennai, Directorate General of Health Services, Ministry of Health and Family Welfare, Government of India. Annual Risk of Tuberculous Infection in Different Zones of India: A National Sample Survey 2000-2003. [Data shared for this analysis].
National Tuberculosis Institute-Bangalore, Tuberculosis Research Center-Chennai, Directorate General of Health Services, Ministry of Health and Family Welfare, Government of India. Report of Annual Risk of Tuberculous Infection in Different Zones of India: A National Sample Survey 2000-2003. Bangalore, India: National Tuberculosis Institute; 2004.
National Vector Borne Disease Control Programme (NVBDCP), Directorate General of Health Services, Ministry of Health and Family Welfare, Government of India. India Dengue Cases and Deaths 2007-2014. New Delhi, India: NVBDCP.
National Vector Borne Disease Control Programme (NVBDCP), Directorate General of Health Services, Ministry of Health and Family Welfare, Government of India. India Visceral Leishmaniasis Cases and Deaths 2007-2014. New Delhi, India: NVBDCP.
National Vector Borne Disease Control Programme (NVBDCP), Directorate General of Health Services, Ministry of Health and Family Welfare, Government of India. National Roadmap for the Elimination of Lymphatic Filariasis 2002. New Delhi, India: NVBDCP.
National Vector Borne Disease Control Programme (NVBDCP), Directorate General of Health Services, Ministry of Health and Family Welfare, Government of India. State-wise Lymphatic Filariasis Programme Data 2004-2014. New Delhi, India: NVBDCP.
National Vector Borne Disease Control Programme (NVBDCP), Directorate General of Health Services, Ministry of Health and Family Welfare, Government of India. State-wise Malaria Programme Data 2013-2017. New Delhi, India: NVBDCP; 2017. [Data shared for this analysis]
Natural Earth, North American Cartographic Information Society (NACIS), Washington Post. Natural Earth Vector and Raster Map Data. Milwaukee, United States: Natural Earth.

Table 3: GBD 2016 India data inputs
Natural Environment Research Council (NERC), US Department of Energy, UK National Centre for Atmospheric Science. Climatic Research Unit-Time Series v.3.24 High Resolution Gridded Time-Series Dataset 1901-2015. Swindon, United Kingdom: NERC. Available from: http://crudata.uea.ac.uk/cru/data/hrg
Nazareth Hospital. Inpatient and Discharge Data 2014. Shillong, Meghalaya: Nazareth Hospital. [Data shared for this analysis]
Nazir A, Papita R, Anbalagan VP, Anjana RM, Deepa M, Mohan V. Prevalence of diabetes in Asian Indians based on glycated hemoglobin and fasting and 2-H post-load (75-g) plasma glucose (CURES-120). Diabetes Technol Ther. 2012; 14(8): 665–8.
Neena J, Rachel J, Praveen V, Murthy GVS. Rapid Assessment of Avoidable Blindness in India. PLoS One. 2008; 3(8): e2867.
Negi PC, Kanwar A, Chauhan R, Asotra S, Thakur JS, Bhardwaj AK. Epidemiological trends of RF/RHD in school children of Shimla in north India. Indian J Med Res. 2013; 137(6.0): 1121-7.
Nelson DI, Concha-Barrientos M, Driscoll T, Steenland K, Fingerhut M, Punnett L, Prüss-Ustün A, Leigh J, Corvalan C. The global burden of selected occupational diseases and injury risks: Methodology and summary. Am J Ind Med. 2005; 48(6): 400-18.
New Delhi Tuberculosis Centre. Study of Epidemiology of Tuberculosis in an Urban Population of Delhi: Report on 30 year Follow-Up. Indian J Tuberc. 1999; 46:113–124
Nichani V, Li W-I, Smith MA, Noonan G, Kulkarni M, Kodavor M, Naeher LP. Blood lead levels in children after phase-out of leaded gasoline in Bombay, India. Sci Total Environ. 2006; 363(1-3): 95-106.
Nidhi R, Padmalatha V, Nagarathna R, Amritanshu R. Prevalence of polycystic ovarian syndrome in Indian adolescents. J Pediatr Adolesc Gynecol. 2011; 24(4): 223-7.
Ningombam S, Hutin Y, Murhekar MV. Prevalence and pattern of substance use among the higher secondary school students of Imphal, Manipur, India. Natl Med J India. 2011; 24(1): 11-5.
Nirmalan PK, Katz J, Robin AL, Tielsch JM, Namperumalsamy P, Kim R, Narendran V, Ramakrishnan R, Krishnadas R, Thulasiraj RD, Suan E. Prevalence of vitreoretinal disorders in a rural population of southern India: the Aravind Comprehensive Eye Study. Arch Ophthalmol. 2004; 122(4): 581-6.
Nirmalan PK, Thulasiraj RD, Maneksha V, Rahmathullah R, Ramakrishnan R, Padmavathi A, Munoz SR, Ellwein LB. A population based eye survey of older adults in Tirunelveli district of south India: blindness, cataract surgery, and visual outcomes. Br J Ophthalmol. 2002; 86(5): 505-12.
Nirmalan PK, Tielsch JM, Katz J, Thulasiraj RD, Krishnadas R, Ramakrishnan R, Robin AL. Relationship between vision impairment and eye disease to vision-specific quality of life and function in rural India: the Aravind Comprehensive Eye Survey. Invest Ophthalmol Vis Sci. 2005; 46(7): 2308-12.
Nirmalan PK, Vijayalakshmi P, Sheeladevi S, Kothari MB, Sundaresan K, Rahmathullah L. The Kariapatti pediatric eye evaluation project: baseline ophthalmic data of children aged 15 years or younger in Southern India. Am J Ophthalmol. 2003; 136(4): 703-9.
Nisarga R, Premalatha R, Shivananda , Ravikumar KL, Shivappa U, Gopi A, Chikkadasarahalli SB, Batuwanthudawe R, Kilgore PE, Kim SA, Balter I, Jouve S, Ye J, Moscariello M. Hospital-based surveillance of invasive pneumococcal disease and pneumonia in South Bangalore, India. Indian Pediatr. 2015; 52(3): 205-11.
Nishank SS, Chhotray GP, Kar SK, Ranjit MR. Molecular variants of G6PD deficiency among certain tribal communities of Orissa, India. Ann Hum Biol. 2008; 35(3): 355-61.

Table 3: GBD 2016 India data inputs
Nitin S, Venkatesh V, Husain N, Masood J, Agarwal GG. Overview of intestinal parasitic prevalence in rural and urban population in Lucknow, north India. J Commun Dis. 2007; 39(4): 217–23.
Nizamie SH, Akthar S, Banerjee I, Goyal N. Health care delivery model in epilepsy to reduce treatment gap: World Health Organization study from a rural tribal population of India. Epilepsy Res. 2009; 84(2–3): 146–52.
Norboo T, Stobdan T, Tsering N, Angchuk N, Tsering P, Ahmed I, Chorol T, Kumar Sharma V, Reddy P, Singh SB, Kimura Y, Sakamoto R, Fukutomi E, Ishikawa M, Suwa K, Kosaka Y, Nose M, Yamaguchi T, Tsukihara T, Matsubayashi K, Otsuka K, Okumiya K. Prevalence of hypertension at high altitude: cross-sectional survey in Ladakh, Northern India 2007-2011. BMJ Open. 2015; 5(4): e007026.
Norboo T, Stobdan T, Tsering N, Angchuk N, Tsering P, Ahmed I, Chorol T, Kumar Sharma V, Reddy P, Singh SB, Kimura Y, Sakamoto R, Fukutomi E, Ishikawa M, Suwa K, Kosaka Y, Nose M, Yamaguchi T, Tsukihara T, Matsubayashi K, Otsuka K, Okumiya K. Prevalence of hypertension at high altitude: cross-sectional survey in Ladakh, Northern India 2007-2011. BMJ Open. 2015; 5(4): e007026.
Nujum ZT, KR LIA, Haran JC, Vijayakumar K, Prabhakaran ST, Noushad SA. Need for a differential criteria to stop mass drug administration, based on an epidemiological perspective of lymphatic filariasis in Thiruvananthapuram, Kerala, India. Asian Pac J Trop Dis. 2014; 4(S1): S186-S193.
O'Brien D, Spelman T, Greig J, McMahon J, Ssonko C, Casas E, Mesic A, Du Cros P, Ford N. Risk factors for mortality during antiretroviral therapy in older populations in resource-limited settings. J Int AIDS Soc. 2016; 19(1): 20665.
Ochiai RL, Acosta CJ, Danovaro-Holliday MC, Baiqing D, Bhattacharya SK, Agtini MD, Bhutta ZA, Canh DG, Ali M, Shin S, Wain J, Page A-L, Albert MJ, Farrar J, Abu-Elyazeed R, Pang T, Galindo CM, von Seidlein L, Clemens JD, Domi Typhoid Study Group. A study of typhoid fever in five Asian countries: disease burden and implications for controls. Bull World Health Organ. 2008; 86(4): 260-8.
Ochiai RL, Wang X, von Seidlein L, Yang J, Bhutta ZA, Bhattacharya SK, Agtini M, Deen JL, Wain J, Kim DR, Ali M, Acosta CJ, Jodar L, Clemens JD. Salmonella Paratyphi A Rates, Asia. Emerg Infect Dis. 2005; 11(11): 1764-6.
Odhiambo JA, Williams HC, Clayton TO, Robertson CF, Asher MI, ISAAC Phase Three Study Group. Global variations in prevalence of eczema symptoms in children from ISAAC Phase Three. J Allergy Clin Immunol. 2009; 124(6): 1251-8.
Office of the Registrar General & Census Commissioner, Centre for Global Health Research - University of Toronto. India SRS Maternal Mortality: Trends, Causes and Risk Factors 1997-2003. New Delhi, India: Office of the Registrar General & Census Commissioner; 2006.
Office of the Registrar General & Census Commissioner, Centre for Global Health Research - University of Toronto. India SRS Special Fertility and Mortality Survey 1998. New Delhi, India: Office of the Registrar General & Census Commissioner; 2005.
Office of the Registrar General & Census Commissioner, Ministry of Home Affairs, Government of India. India Annual Health Survey Data 2010-2011. New Delhi, India: Office of the Registrar General & Census Commissioner.
Office of the Registrar General & Census Commissioner, Ministry of Home Affairs, Government of India. India Annual Health Survey Data 2010-2013. New Delhi, India: Office of the Registrar General & Census Commissioner.
Office of the Registrar General & Census Commissioner, Ministry of Home Affairs, Government of India. India Annual Health Survey Data 2011-2012. New Delhi, India: Office of the Registrar General & Census Commissioner.
Office of the Registrar General & Census Commissioner, Ministry of Home Affairs, Government of India. India Annual Health Survey Data 2012-2013. New Delhi, India: Office of the Registrar General & Census Commissioner.

Table 3: GBD 2016 India data inputs
Office of the Registrar General & Census Commissioner, Ministry of Home Affairs, Government of India. India Annual Health Survey Report 2010-2011. New Delhi, India: Office of the Registrar General & Census Commissioner.
Office of the Registrar General & Census Commissioner, Ministry of Home Affairs, Government of India. India Annual Health Survey Report 2010-2013. New Delhi, India: Office of the Registrar General & Census Commissioner.
Office of the Registrar General & Census Commissioner, Ministry of Home Affairs, Government of India. India Annual Health Survey Report 2011-2012. New Delhi, India: Office of the Registrar General & Census Commissioner.
Office of the Registrar General & Census Commissioner, Ministry of Home Affairs, Government of India. India Annual Health Survey Report 2012-2013. New Delhi, India: Office of the Registrar General & Census Commissioner.
Office of the Registrar General & Census Commissioner, Ministry of Home Affairs, Government of India. India Census 1981. New Delhi, India: Office of the Registrar General & Census Commissioner.
Office of the Registrar General & Census Commissioner, Ministry of Home Affairs, Government of India. India Medical Certification of Cause of Death Report 1997. New Delhi, India: Office of the Registrar General & Census Commissioner.
Office of the Registrar General & Census Commissioner, Ministry of Home Affairs, Government of India. India Medical Certification of Cause of Death Report 2005. New Delhi, India: Office of the Registrar General & Census Commissioner.
Office of the Registrar General & Census Commissioner, Ministry of Home Affairs, Government of India. India Medical Certification of Cause of Death Report 2006. New Delhi, India: Office of the Registrar General & Census Commissioner.
Office of the Registrar General & Census Commissioner, Ministry of Home Affairs, Government of India. India Medical Certification of Cause of Death Report 2008. New Delhi, India: Office of the Registrar General & Census Commissioner.
Office of the Registrar General & Census Commissioner, Ministry of Home Affairs, Government of India. India Medical Certification of Cause of Death Report 2009. New Delhi, India: Office of the Registrar General & Census Commissioner; 2014.
Office of the Registrar General & Census Commissioner, Ministry of Home Affairs, Government of India. India Medical Certification of Cause of Death Report 2010. New Delhi, India: Office of the Registrar General & Census Commissioner; 2014.
Office of the Registrar General & Census Commissioner, Ministry of Home Affairs, Government of India. India Medical Certification of Cause of Death Report 2011. New Delhi, India: Office of the Registrar General & Census Commissioner.
Office of the Registrar General & Census Commissioner, Ministry of Home Affairs, Government of India. India Medical Certification of Cause of Death Report 2012. New Delhi, India: Office of the Registrar General & Census Commissioner; 2015.
Office of the Registrar General & Census Commissioner, Ministry of Home Affairs, Government of India. India Medical Certification of Cause of Death State-Level Tabulations 1990. New Delhi, India: Office of the Registrar General & Census Commissioner.
Office of the Registrar General & Census Commissioner, Ministry of Home Affairs, Government of India. India Medical Certification of Cause of Death State-Level Tabulations 1991. New Delhi, India: Office of the Registrar General & Census Commissioner.

Table 3: GBD 2016 India data inputs
Office of the Registrar General & Census Commissioner, Ministry of Home Affairs, Government of India. India Medical Certification of Cause of Death State-Level Tabulations 1992. New Delhi, India: Office of the Registrar General & Census Commissioner.
Office of the Registrar General & Census Commissioner, Ministry of Home Affairs, Government of India. India Medical Certification of Cause of Death State-Level Tabulations 1993. New Delhi, India: Office of the Registrar General & Census Commissioner.
Office of the Registrar General & Census Commissioner, Ministry of Home Affairs, Government of India. India Medical Certification of Cause of Death State-Level Tabulations 1994. New Delhi, India: Office of the Registrar General & Census Commissioner.
Office of the Registrar General & Census Commissioner, Ministry of Home Affairs, Government of India. India Medical Certification of Cause of Death State-Level Tabulations 1995. New Delhi, India: Office of the Registrar General & Census Commissioner.
Office of the Registrar General & Census Commissioner, Ministry of Home Affairs, Government of India. India Medical Certification of Cause of Death State-Level Tabulations 1996. New Delhi, India: Office of the Registrar General & Census Commissioner.
Office of the Registrar General & Census Commissioner, Ministry of Home Affairs, Government of India. India Medical Certification of Cause of Death State-Level Tabulations 1998. New Delhi, India: Office of the Registrar General & Census Commissioner.
Office of the Registrar General & Census Commissioner, Ministry of Home Affairs, Government of India. India Medical Certification of Cause of Death State-Level Tabulations 1999. New Delhi, India: Office of the Registrar General & Census Commissioner.
Office of the Registrar General & Census Commissioner, Ministry of Home Affairs, Government of India. India Medical Certification of Cause of Death State-Level Tabulations 2000. New Delhi, India: Office of the Registrar General & Census Commissioner.
Office of the Registrar General & Census Commissioner, Ministry of Home Affairs, Government of India. India Medical Certification of Cause of Death State-Level Tabulations 2001. New Delhi, India: Office of the Registrar General & Census Commissioner.
Office of the Registrar General & Census Commissioner, Ministry of Home Affairs, Government of India. India Medical Certification of Cause of Death State-Level Tabulations 2002. New Delhi, India: Office of the Registrar General & Census Commissioner.
Office of the Registrar General & Census Commissioner, Ministry of Home Affairs, Government of India. India Medical Certification of Cause of Death State-Level Tabulations 2003. New Delhi, India: Office of the Registrar General & Census Commissioner.
Office of the Registrar General & Census Commissioner, Ministry of Home Affairs, Government of India. India Medical Certification of Cause of Death State-Level Tabulations 2004. New Delhi, India: Office of the Registrar General & Census Commissioner.
Office of the Registrar General & Census Commissioner, Ministry of Home Affairs, Government of India. India Population and Housing Census 1981. New Delhi, India: Office of the Registrar General & Census Commissioner.
Office of the Registrar General & Census Commissioner, Ministry of Home Affairs, Government of India. India Population and Housing Census 1991. New Delhi, India: Office of the Registrar General & Census Commissioner.
Office of the Registrar General & Census Commissioner, Ministry of Home Affairs, Government of India. India Population and Housing Census 2001. New Delhi, India: Office of the Registrar General & Census Commissioner.
Office of the Registrar General & Census Commissioner, Ministry of Home Affairs, Government of India. India Population and Housing Census 2011. New Delhi, India: Office of the Registrar General & Census Commissioner; 2012.

Table 3: GBD 2016 India data inputs
Office of the Registrar General & Census Commissioner, Ministry of Home Affairs, Government of India. India Sample Registration System Age-Specific Death Rates 1970-2006. New Delhi, India: Office of the Registrar General & Census Commissioner.
Office of the Registrar General & Census Commissioner, Ministry of Home Affairs, Government of India. India Sample Registration System Baseline Survey 2014. New Delhi, India: Office of the Registrar General & Census Commissioner.
Office of the Registrar General & Census Commissioner, Ministry of Home Affairs, Government of India. India SRS Bulletin 2010. New Delhi, India: Office of the Registrar General & Census Commissioner; 2011.
Office of the Registrar General & Census Commissioner, Ministry of Home Affairs, Government of India. India SRS Maternal Mortality Ratio Bulletin 2011-2013. New Delhi, India: Office of the Registrar General & Census Commissioner.
Office of the Registrar General & Census Commissioner, Ministry of Home Affairs, Government of India. India SRS Maternal Mortality Tables 2001-2009. New Delhi, India: Office of the Registrar General & Census Commissioner.
Office of the Registrar General & Census Commissioner, Ministry of Home Affairs, Government of India. India SRS Special Bulletin on Maternal Mortality 2010-2012. New Delhi, India: Office of the Registrar General & Census Commissioner; 2013.
Office of the Registrar General & Census Commissioner, Ministry of Home Affairs, Government of India. India SRS Statistical Report 1995. New Delhi, India: Office of the Registrar General & Census Commissioner.
Office of the Registrar General & Census Commissioner, Ministry of Home Affairs, Government of India. India SRS Statistical Report 1996. New Delhi, India: Office of the Registrar General & Census Commissioner.
Office of the Registrar General & Census Commissioner, Ministry of Home Affairs, Government of India. India SRS Statistical Report 1996. New Delhi, India: Office of the Registrar General & Census Commissioner; 1998.
Office of the Registrar General & Census Commissioner, Ministry of Home Affairs, Government of India. India SRS Statistical Report 1997. New Delhi, India: Office of the Registrar General & Census Commissioner; 2001.
Office of the Registrar General & Census Commissioner, Ministry of Home Affairs, Government of India. India SRS Statistical Report 1998. New Delhi, India: Office of the Registrar General & Census Commissioner; 2000.
Office of the Registrar General & Census Commissioner, Ministry of Home Affairs, Government of India. India SRS Statistical Report 1999. New Delhi, India: Office of the Registrar General & Census Commissioner.
Office of the Registrar General & Census Commissioner, Ministry of Home Affairs, Government of India. India SRS Statistical Report 2000. New Delhi, India: Office of the Registrar General & Census Commissioner.
Office of the Registrar General & Census Commissioner, Ministry of Home Affairs, Government of India. India SRS Statistical Report 2001. New Delhi, India: Office of the Registrar General & Census Commissioner; 2005.
Office of the Registrar General & Census Commissioner, Ministry of Home Affairs, Government of India. India SRS Statistical Report 2002. New Delhi, India: Office of the Registrar General & Census Commissioner; 2005.
Office of the Registrar General & Census Commissioner, Ministry of Home Affairs, Government of India. India SRS Statistical Report 2003. New Delhi, India: Office of the Registrar General and Census Commissioner; 2005.

Table 3: GBD 2016 India data inputs
Office of the Registrar General & Census Commissioner, Ministry of Home Affairs, Government of India. India SRS Statistical Report 2004. New Delhi, India: Office of the Registrar General and Census Commissioner; 2006.
Office of the Registrar General & Census Commissioner, Ministry of Home Affairs, Government of India. India SRS Statistical Report 2005. New Delhi, India: Office of the Registrar General and Census Commissioner; 2006.
Office of the Registrar General & Census Commissioner, Ministry of Home Affairs, Government of India. India SRS Statistical Report 2006. New Delhi, India: Office of the Registrar General and Census Commissioner; 2008.
Office of the Registrar General & Census Commissioner, Ministry of Home Affairs, Government of India. India SRS Statistical Report 2007. New Delhi, India: Office of the Registrar General and Census Commissioner; 2008.
Office of the Registrar General & Census Commissioner, Ministry of Home Affairs, Government of India. India SRS Statistical Report 2008. New Delhi, India: Office of the Registrar General and Census Commissioner; 2010.
Office of the Registrar General & Census Commissioner, Ministry of Home Affairs, Government of India. India SRS Statistical Report 2009. New Delhi, India: Office of the Registrar General and Census Commissioner.
Office of the Registrar General & Census Commissioner, Ministry of Home Affairs, Government of India. India SRS Statistical Report 2010. New Delhi, India: Office of the Registrar General and Census Commissioner; 2012.
Office of the Registrar General & Census Commissioner, Ministry of Home Affairs, Government of India. India SRS Statistical Report 2011. New Delhi, India: Office of the Registrar General and Census Commissioner; 2013.
Office of the Registrar General & Census Commissioner, Ministry of Home Affairs, Government of India. India SRS Statistical Report 2012. New Delhi, India: Office of the Registrar General and Census Commissioner; 2013.
Office of the Registrar General & Census Commissioner, Ministry of Home Affairs, Government of India. India SRS Statistical Report 2013. New Delhi, India: Office of the Registrar General and Census Commissioner; 2014.
Office of the Registrar General & Census Commissioner, Ministry of Home Affairs, Government of India. India SRS Statistical Report 2014. New Delhi, India: Office of the Registrar General and Census Commissioner.
Office of the Registrar General & Census Commissioner, Ministry of Home Affairs, Government of India. India SRS Statistical Report 2015. New Delhi, India: Office of the Registrar General and Census Commissioner.
Office of the Registrar General & Census Commissioner, Ministry of Home Affairs, Government of India. India SRS Verbal Autopsy 2004-2006. New Delhi, India: Office of the Registrar General and Census Commissioner. [Data shared for this analysis]
Office of the Registrar General & Census Commissioner, Ministry of Home Affairs, Government of India. India SRS Verbal Autopsy 2007-2009. New Delhi, India: Office of the Registrar General and Census Commissioner. [Data shared for this analysis]
Office of the Registrar General & Census Commissioner, Ministry of Home Affairs, Government of India. India SRS Verbal Autopsy 2010-2013. New Delhi, India: Office of the Registrar General and Census Commissioner. [Data shared for this analysis]

Table 3: GBD 2016 India data inputs
Office of the Registrar General & Census Commissioner, Ministry of Home Affairs, Government of India. India SRS Verbal Autopsy Report 2004-2006. New Delhi, India: Office of the Registrar General and Census Commissioner.
Office of the Registrar General & Census Commissioner, Ministry of Home Affairs, Government of India. India SRS Verbal Autopsy Report 2007-2009. New Delhi, India: Office of the Registrar General and Census Commissioner.
Office of the Registrar General & Census Commissioner, Ministry of Home Affairs, Government of India. India SRS Verbal Autopsy Report 2010-2013. New Delhi, India: Office of the Registrar General and Census Commissioner.
Office of the Registrar General & Census Commissioner, Ministry of Home Affairs, Government of India. India Survey of Causes of Death 1980. New Delhi, India: Office of the Registrar General and Census Commissioner.
Office of the Registrar General & Census Commissioner, Ministry of Home Affairs, Government of India. India Survey of Causes of Death 1981. New Delhi, India: Office of the Registrar General and Census Commissioner.
Office of the Registrar General & Census Commissioner, Ministry of Home Affairs, Government of India. India Survey of Causes of Death 1982. New Delhi, India: Office of the Registrar General and Census Commissioner.
Office of the Registrar General & Census Commissioner, Ministry of Home Affairs, Government of India. India Survey of Causes of Death 1983. New Delhi, India: Office of the Registrar General and Census Commissioner.
Office of the Registrar General & Census Commissioner, Ministry of Home Affairs, Government of India. India Survey of Causes of Death 1984. New Delhi, India: Office of the Registrar General and Census Commissioner.
Office of the Registrar General & Census Commissioner, Ministry of Home Affairs, Government of India. India Survey of Causes of Death 1985. New Delhi, India: Office of the Registrar General and Census Commissioner.
Office of the Registrar General & Census Commissioner, Ministry of Home Affairs, Government of India. India Survey of Causes of Death 1986. New Delhi, India: Office of the Registrar General and Census Commissioner.
Office of the Registrar General & Census Commissioner, Ministry of Home Affairs, Government of India. India Vital Statistics 1980. New Delhi, India: Office of the Registrar General and Census Commissioner.
Office of the Registrar General & Census Commissioner, Ministry of Home Affairs, Government of India. India Vital Statistics 1981. New Delhi, India: Office of the Registrar General and Census Commissioner.
Office of the Registrar General & Census Commissioner, Ministry of Home Affairs, Government of India. India Vital Statistics 1982. New Delhi, India: Office of the Registrar General and Census Commissioner.
Office of the Registrar General & Census Commissioner, Ministry of Home Affairs, Government of India. India Vital Statistics 1983. New Delhi, India: Office of the Registrar General and Census Commissioner.
Office of the Registrar General & Census Commissioner, Ministry of Home Affairs, Government of India. India Vital Statistics 1984. New Delhi, India: Office of the Registrar General and Census Commissioner.
Office of the Registrar General & Census Commissioner, Ministry of Home Affairs, Government of India. India Vital Statistics 1986. New Delhi, India: Office of the Registrar General and Census Commissioner.

Table 3: GBD 2016 India data inputs
Office of the Registrar General & Census Commissioner, Ministry of Home Affairs, Government of India. India Vital Statistics 1987. New Delhi, India: Office of the Registrar General and Census Commissioner.
Office of the Registrar General & Census Commissioner, Ministry of Home Affairs, Government of India. India Vital Statistics 1989. New Delhi, India: Office of the Registrar General and Census Commissioner.
Office of the Registrar General & Census Commissioner, Ministry of Home Affairs, Government of India. India Vital Statistics 1990. New Delhi, India: Office of the Registrar General and Census Commissioner.
Office of the Registrar General & Census Commissioner, Ministry of Home Affairs, Government of India. India Vital Statistics 1991. New Delhi, India: Office of the Registrar General and Census Commissioner.
Office of the Registrar General & Census Commissioner, Ministry of Home Affairs, Government of India. India Vital Statistics 1992. New Delhi, India: Office of the Registrar General and Census Commissioner.
Office of the Registrar General & Census Commissioner, Ministry of Home Affairs, Government of India. India Vital Statistics 1993. New Delhi, India: Office of the Registrar General and Census Commissioner.
Office of the Registrar General & Census Commissioner, Ministry of Home Affairs, Government of India. India Vital Statistics 1994. New Delhi, India: Office of the Registrar General and Census Commissioner.
Office of the Registrar General & Census Commissioner, Ministry of Home Affairs, Government of India. India Vital Statistics 1995. New Delhi, India: Office of the Registrar General and Census Commissioner.
Office of the Registrar General and Census Commissioner, Ministry of Health and Family Welfare, Government of India, National Institute of Health and Family Welfare, Nutrition Foundation of India, National Institute of Nutrition, Indian Council of Medical Research. India Clinical, Anthropometric and Bio-chemical (CAB) Survey Data 2014 [Biomarker Component of Annual Health Survey]. New Delhi, India: Office of the Registrar General and Census Commissioner.
Office of the Registrar General and Census Commissioner, Ministry of Health and Family Welfare, Government of India, National Institute of Health and Family Welfare, Nutrition Foundation of India, National Institute of Nutrition, Indian Council of Medical Research. India Clinical, Anthropometric and Bio-chemical (CAB) Survey Report 2014 [Biomarker Component of Annual Health Survey]. New Delhi, India: Office of the Registrar General and Census Commissioner.
Olofin I, McDonald CM, Ezzati M, Flaxman S, Black RE, Fawzi WW, Caulfield LE, Danaei G; Nutrition Impact Model Study (anthropometry cohort pooling). Associations of suboptimal growth with all-cause and cause-specific mortality in children under five years: a pooled analysis of ten prospective studies. PLoS One. 2013; 8(5): e64636.
Oommen A.M, Abraham V.J, George K, Jose J.J. Prevalence of Risk Factors for Non-Communicable Diseases in Rural and Urban Tamil Nadu 2010-2012. Indian J Med Res. 2016 Sep; 144(3): 460–71.
Oommen AM, Abraham VJ, George K, Jose VJ. Prevalence of coronary heart disease in rural and urban Vellore: A repeat cross-sectional survey. Indian Heart J. 2016; 68(4): 473-9.
Padmavathi R, Rajkumar S, Kumar N, Manoharan A, Kamath S. Prevalence of schizophrenia in an urban community in madras. Indian J Psychiatry. 1988; 30(3): 233-9.

Table 3: GBD 2016 India data inputs
Pagidipati NJ, Huffman MD, Jeemon P, Gupta R, Negi P, Jaison TM, Sharma S, Sinha N, Mohanan P, Muralidhara BG, Bijulal S, Sivasankaran S, Puri VK, Jose J, Reddy KS, Prabhakaran D. Association between gender, process of care measures, and outcomes in ACS in India: results from the detection and management of coronary heart disease (DEMAT) registry. PLoS One. 2013; 8(4): e62061.
Pahuja S, Sharma M, Baitha B, Jain M. Prevalence and trends of markers of hepatitis C virus, hepatitis B virus and human immunodeficiency virus in Delhi blood donors: a hospital based study. Jpn J Infect Dis. 2007; 60(6): 389-91.
Pal A, Bhattacharyya R, Adhikari S, Roy A, Chakrabarty D, Ghosh P, Banerjee C. Eclampsia-scenario in a hospital--a ten years study. Bangladesh Med Res Counc Bull. 2011; 37(2): 66-70.
Pal DK, Das T, Sengupta S. Comparison of key informant and survey methods for ascertainment of childhood epilepsy in West Bengal, India. Int J Epidemiol. 1998; 27(4): 672–6.
Pal H, Srivastava A, Dwivedi SN, Pandey A, Nath J. Prevalence of drug abuse in India through a national household survey. Int J Curr Sci. 2015;15:e103–13.
Palaniappan K, Roy A, Balakrishnan K, Gopalakrishnan L, Mukherjee B, Hu H, Bellinger DC. Lead exposure and visual-motor abilities in children from Chennai, India. Neurotoxicology. 2011; 32(4): 465-70.
Palimkar A, Khandekar R, Venkataraman V. Prevalence and distribution of glaucoma in central India (Glaucoma Survey 2001). Indian J Ophthalmol. 2008; 56(1): 57-62.
Panchanadeswaran S, Johnson SC, Mayer KH, Srikrishnan AK, Sivaran S, Zelaya CE, Go VF, Solomon S, Bentley ME, Celentano DD. Gender differences in the prevalence of sexually transmitted infections and genital symptoms in an urban setting in southern India. Sex Transm Infect. 2006; 82(6): 491–5.
Pandav CS, Krishnamurthy P, Sankar R, Yadav K, Palanivel C, Karmarkar MG. A review of tracking progress towards elimination of iodine deficiency disorders in Tamil Nadu, India. Indian J Public Health. 2010; 54(3): 120-5.
Pandav CS, Mallik A, Anand K, Pandav S, Karmarkar MG. Prevalence of iodine deficiency disorders among school children of Delhi. Natl Med J India. 1997; 10(3): 112-4.
Pandey GK, Dutt D, Banerjee B. Partner and relationship factors in domestic violence: perspectives of women from a slum in Calcutta, India. J Interpers Violence. 2009; 24(7): 1175-91.
Pandey S, Singhi P, Bharti B. Prevalence and treatment gap in childhood epilepsy in a north Indian city: a community-based study. J Trop Pediatr. 2014; 60(2): 118-23.
Pandian JD, Singh G, Kaur P, Bansal R, Paul BS, Singla M, Singh S, Samuel CJ, Verma SJ, Moodbidri P, Mehmi G, Sharma A, Arora OP, Dhanuka AK, Sobti MK, Sehgal H, Kaur M, Grewal SS, Jhawar SS, Shadangi TN, Arora T, Saxena A, Sachdeva G, Gill JS, Brar RS, Gill A, Bakshi SS, Pawar SS, Singh G, Sikka P, Litoria PK, Sharma M. Incidence, short-term outcome, and spatial distribution of stroke patients in Ludhiana, India. Neurology. 2016; 86(5): 425-33.
Pandian JD. Ludhiana Population Based Stroke Registry 2012-2013 [Data shared for this analysis]
Pandit DP, Pagaro M P, Nabamita C. Prevalence of antibodies to hepatitis C virus in voluntary blood donors: are women better donors?. J Clin Diagn Res. 2014; 8.0(4): DC20-3.
Pandit L, Kundapur R. Prevalence and patterns of demyelinating central nervous system disorders in urban Mangalore, South India. Mult Scler. 2014; 20(12): 1651-3.
Pani SP, Balakrishnan N, Srividya A, Bundy DA, Grenfell BT. Clinical epidemiology of bancroftian filariasis: effect of age and gender. Trans R Soc Trop Med Hyg. 1991; 85(2): 260-4.

Table 3: GBD 2016 India data inputs
Pani SP, Krishnamoorthy K, Rao AS, Prathiba J. Clinical manifestations in malayan filariasis infection with special reference to lymphoedema grading. Indian J Med Res. 1990; 91: 200-7.
Panicker PV, Gadkari AS, Kulkarni SW, Handa BK, Joshi MW. Prevalence of hookworm in some villages around Nagpur. J Commun Dis. 1980; 12(4): 192–6.
Panigrahi D, Agarwal KC, Kaur T, Ayyagari A, Walia BN. A study of rotavirus diarrhoea in children in a north Indian community. J Diarrhoeal Dis Res. 1985; 3(1): 20-3.
Panigrahi S, Patra PK, Khodiar PK. Neonatal screening of sickle cell anemia: a preliminary report. Indian J Pediatr. 2012; 79(6): 747–50.
Paniker CK, Mathew S, Mathan M. Rotavirus and acute diarrhoeal disease in children in a southern Indian coastal town. Bull World Health Organ. 1982; 60(1): 123-7.
Pao M, Kulkarni A, Gupta V, Kaul S, Balan S. Neonatal screening for glucose-6-phosphate dehydrogenase deficiency. Indian J Pediatr. 2005; 72(10): 835-7.
Parasuramalu BG, Huliraj N, Rudraprasad BM, Prashanth Kumar SP, Gangaboraiah, Ramesh Masthi NR. Prevalence of bronchial asthma and its association with smoking habits among adult population in rural area. Indian J Public Health. 2010; 54(3): 165-8.
Parasuramalu BG, Vastrad SA, Shivaram C. Prevalence of anaemia in the aged population in selected slums of Hubli City. Indian J Public Health. 1990; 34(2): 117-8.
Parihar YS, Patnaik JP, Nema BK, Sahoo GB, Misra IB, Adhikary S. Coal workers' pneumoconiosis: a study of prevalence in coal mines of eastern Madhya Pradesh and Orissa states of India. Ind Health. 1997; 35(4): 467-73.
Parikha J, Balakrishnanb K, Laxmia V, Biswasa H. Exposure from cooking with biofuels: pollution monitoring and analysis for rural Tamil Nadu, India. Energy. 2001; 26(10): 949–62.
Parkin DM, Muir CS, Whelan SL, Gao YT, Ferlay J, Powell J, eds. Cancer Incidence in Five Continents, Vol. VI. International Agency for Research on Cancer (IARC) Scientific Publications, No. 120. Lyon, France: IARC; 1992.
Parkin DM, Whelan SL, Ferlay J, Raymond L, Young J, eds. Cancer Incidence in Five Continents, Vol. VII International Agency for Research on Cancer (IARC) Scientific Publications, No. 143, Lyon, France: IARC; 1997.
Parkin DM, Whelan SL, Ferlay J, Teppo L, Thomas DB, eds. Cancer Incidence in Five Continents, Vol. VIII International Agency for Research on Cancer (IARC) Scientific Publications, No. 155. Lyon, France: IARC; 2002.
Pasricha S-R, Black J, Muthayya S, Shet A, Bhat V, Nagaraj S, Prashanth NS, Sudarshan H, Biggs B-A, Shet AS. Determinants of anemia among young children in rural India. Pediatrics. 2010; 126(1): e140-9.
Patandin S, Bots ML, Abel R, Valkenburg HA. Impaired glucose tolerance and diabetes mellitus in a rural population in south India. Diabetes Res Clin Pract. 1994; 24(1): 47-53.
Patel AB, Mamtani MR, Thakre TP, Kulkarni H. Association of umbilical cord blood lead with neonatal behavior at varying levels of exposure. Behav Brain Funct . 2006; 2(1):22.
Patel AB, Prabhu AS. Determinants of lead level in umbilical cord blood. Indian Pediatr. 2009; 46(9): 791–3.
Patel AB, Williams SV, Frumkin H, Kondawar VK, Glick H, Ganju AK. Blood lead in children and its determinants in Nagpur, India. Int J Occup Environ Health. 2001; 7(2): 119-26.
Patel J, Patel B, Gamit N, Serjeant GR. Screening for the sickle cell gene in Gujarat, India: a village-based model. J Community Genet. 2013; 4(1): 43–7.

Table 3: GBD 2016 India data inputs
Patel JK, Vyas AP, Berman B, Vierra M. Incidence of childhood dermatosis in India. Skinmed. 2010; 8(3): 136-42.
Patel V, Andrew G. Gender, sexual abuse and risk behaviours in adolescents: a cross-sectional survey in schools in Goa. Natl Med J India. 2001; 14(5): 263-7.
Pathak P, Singh P, Kapil U, Raghuvanshi RS. Prevalence of iron, vitamin A, and iodine deficiencies amongst adolescent pregnant mothers. Indian J Pediatr. 2003; 70(4): 299-301.
Pathak P, Tandon M, Kapil U, Singh C. Prevalence of iron deficiency anemia amongst pregnant women in urban slum communities of Delhi. Indian Pediatr. 1999; 36(3): 322-3.
Pathak S, Chandrashekhar M. Transfusion transmittable infections - Seroprevalence among blood donors in a tertiary care hospital of Delhi. Asian J Transfus Sci. 2013; 7(2): 116–8.
Patil AJ, Bhagwat VR, Patil JA, Dongre NN, Ambekar JG, Das KK. Biochemical aspects of lead exposure in silver jewelry workers in western Maharashtra (India). J Basic Clin Physiol Pharmacol. 2006; 17(4): 213-29.
Patil RN, Nagaonkar SN, Shah NB, Bhat TS. A Cross-sectional Study of Common Psychiatric Morbidity in Children Aged 5 to 14 Years in an Urban Slum. J Fam Med Prim Care. 2013; 2(2): 164-8.
Patil S, Bhosale R, Sambarey P, Gupte N, Suryavanshi N, Sastry J, Bollinger RC, Gupta A, Shankar A. Impact of maternal human immunodeficiency virus infection on pregnancy and birth outcomes in Pune, India. AIDS Care. 2011; 23(12): 1562-9.
Patil S, Gogate P, Vora S, Ainapure S, Hingane RN, Kulkarni AN, Shammanna BR. Prevalence, causes of blindness, visual impairment and cataract surgical services in Sindhudurg district on the western coastal strip of India. Indian J Ophthalmol. 2014; 62(2): 240-245.
Patil YP, Shinde RL. Undernutrition among Indian men: a study based on NFHS-3. Am J Men Health. 2014; 8(6): 492–502.
Patnaik SK, Mukhopadhyay AK. Prevalence of lymphatic filariasis and economic impact of chronic forms of the disease in a group of weavers in the Godavari Districts of Andhra Pradesh. Indian J Public Health Res Dev. 2016; 7(2): 103-7.
Patowary AC, Kumar S, Patowary S, Dhar P. Iodine deficiency disorders (IDD) and iodised salt in Assam: a few observations. Indian J Public Health. 1995; 39(4): 135-40.
Patra PK, Chauhan VS, Khodiar PK, Dalla AR, Serjeant GR. Screening for the sickle cell gene in Chhattisgarh state, India: an approach to a major public health problem. J Community Genet. 2011; 2(3): 147–51.
Pattanayak, Ipsa. Distribution of A1A2BO and Rh Blood Groups Among the Rajputs of Uttaranchal. Anthropologist. 2006; 8(2): 139-40.
Patwari AK, Bisht S, Srinivasan A, Deb M, Chattopadhya D. Aetiology of pneumonia in hospitalized children. J Trop Pediatr. 1996; 42(1): 15–20.
Paul I, Gnanamani G, Nallam NR. Intestinal helminth infections among school children in Visakhapatnam. Indian J Pediatr. 1999; 66(5): 669–73.
Paul SB, Chalamalasetty SB, Vishnubhatla S, Madan K, Gamanagatti SR, Batra Y, Gupta SD, Panda SK, Acharya SK. Clinical Profile, Etiology and Therapeutic Outcome in 324 Hepatocellular Carcinoma Patients at a Tertiary Care Center in India. Oncology. 2009; 77(3-4): 162-71.
Pazhanivel M, Jayanthi V. Diabetes mellitus and cirrhosis liver. Minerva Gastroenterol Dietol. 2010; 56(1): 7-11.
Pearce N, Ait-Khaled N, Beasley R, Mallol J, Keil U, Mitchell E, et al. Worldwide trends in the prevalence of asthma symptoms: phase III of the International Study of Asthma and Allergies in Childhood (ISAAC). Thorax. 2007;62(9):758–66.

Table 3: GBD 2016 India data inputs
Pemminati S, Prabha Adhikari MR, Pathak R, Pai MRSM. Prevalence of metabolic syndrome (METS) using IDF 2005 guidelines in a semi urban south Indian (Boloor Diabetes Study) population of Mangalore. J Assoc Physicians India. 2010; 58: 674–7.
Pereira CE, Vaidyan VK, Chougaonkar MP, Mayya YS, Sahoo BK, Jojo PJ. Indoor radon and thoron levels in Neendakara and Chavara regions of southern coastal Kerala, India. Radiat Prot Dosimetry. 2012; 150(3): 385-90.
Periwal KL, Gupta BK, Panwar RB, Khatri PC, Raja S, Gupta R. Prevalence of rheumatic heart disease in school children in Bikaner: an echocardiographic study. J Assoc Physicians India. 2006; 54: 279-82.
Pew Research Center. The Future of the Global Muslim Population. Washington D.C., United States: Pew Research Center; 2011.
Phaneendra Rao RS, Kumari J, Krishna Rao TS, Narasimham VL. Measles in a rural community. J Commun Dis. 1988; 20(2): 131-5.
Phukan RK, Mahanta J. A study of neonatal deaths in the tea gardens of Dibrugarh district of upper Assam. J Indian Med Assoc. 1998; 96(11): 333-4, 337.
Pigott DM, Bhatt S, Golding N, Duda KA, Battle KE, Brady OJ, Messina JP, Balard Y, Bastien P, Pratlong F, Brownstein JS, Freifeld CC, Mekaru SR, Gething PW, George DB, Myers MF, Reithinger R, Hay SI. Global distribution maps of the leishmaniases. eLife 2014;3:e02851
Pillai A, Nayak MB, Greenfield TK, Bond JC, Hasin DS, Patel V. Adolescent drinking onset and its adult consequences among men: A population based study from India. J Epidemiol Community Health. 2014; 68(10): 922–7.
Pillai A, Nayak MB, Greenfield TK, Bond JC, Nadkarni A, Patel V. Patterns of alcohol use, their correlates, and impact in male drinkers: a population-based survey from Goa, India. Soc Psychiatry Psychiatr Epidemiol. 2013; 48(2): 275–82.
Pillai A, Patel V, Cardozo P, Goodman R, Weiss HA, Andrew G. Non-traditional lifestyles and prevalence of mental disorders in adolescents in Goa, India. Br J Psychiatry. 2008; 192(1): 45-51.
Poongothai S, Pradeepa R, Ganesan A, Mohan V. Prevalence of depression in a large urban South Indian population – the Chennai Urban Rural Epidemiology Study (CURES-70). PLoS One. 2009; 4(9): e7185.
Poovathinal SA, Anitha A, Thomas R, Kaniamattam M, Melempatt N, Anilkumar A, Meena M. Prevalence of autism spectrum disorders in a semiurban community in south India. Ann Epidemiol. 2016; 26(9): 663–665e8.
Postgraduate Institute of Medical Education & Research (PGIMER) Chandigarh, Tata Memorial Center. Cancer Incidence and Mortality in S.A.S. Nagar District 2013. Chandigarh, India: PGIMER Chandigarh.
Postgraduate Institute of Medical Education & Research (PGIMER), Tata Memorial Center, Civil Hospital-Mansa District. Cancer Incidence and Mortality in Mansa District. Chandigarh, India: PGIMER; 2013.
Postgraduate Institute of Medical Education & Research (PGIMER), Tata Memorial Center, Civil Hospital-Sangur District. Cancer Incidence and Mortality in Sangrur District. Chandigarh, India: PGIMER; 2013.
Pradeepa R, Rema M, Vignesh J, Deepa M, Deepa R, Mohan V. Prevalence and risk factors for diabetic neuropathy in an urban south Indian population: the Chennai Urban Rural Epidemiology Study (CURES-55). Diabet Med. 2008; 25(4): 407–12.
Prakash A, Bhattacharyya DR, Mohapatra PK, Mahanta J. Role of the prevalent Anopheles species in the transmission of Plasmodium falciparum and P. vivax in Assam state, north-eastern India. Ann Trop Med Parasitol. 2004; 98(6): 559-68.
Prakash A, Mohapatra PK, Bhattacharyya D, Sharma C, Goswami B, Hazarika NC, Mahanta J. Epidemiology of malaria outbreak (April/May, 1999) in Titabor Primary Health Centre, District Jorhat (Assam). Indian J Med Res. 2000; 111: 121–6.

Table 3: GBD 2016 India data inputs
Prakash A, Mohapatra PK, Bhattacharyya DR, Doloi P, Mahanta J. Changing malaria endemicity – a village based study in Sonitpur, Assam. J Commun Dis. 1997; 29(2): 175-8.
Prasad DS, Kabir Z, Dash AK, Das BC. Prevalence and risk factors for diabetes and impaired glucose tolerance in Asian Indians: a community survey from urban eastern India. Diabetes Metab Syndr. 2012; 6(2): 96-101.
Prasad G, Prasad Y, Gusain GS, Ramola RC. Measurement of radon and thoron levels in soil, water and indoor atmosphere of Budhakedar in Garhwal Himalaya, India. Radiat Meas. 2008; S375-S379.
Prasad KN, Prasad A, Gupta RK, Nath K, Pradhan S, Tripathi M, Pandey CM. Neurocysticercosis in patients with active epilepsy from the pig farming community of Lucknow district, north India. Trans R Soc Trop Med Hyg. 2009; 103(2): 144-50.
Prasad RN, Das MK, Sharma T, Dutta GD. Prevalence of filariasis in rural areas of Shahjahanpur district (Uttar Pradesh). Indian J Med Res. 1993; 97: 112-4.
Pratinidhi SA, Patil AJ, Behera M, Patil M, Ghadage DP, Pratinidhi AK. Effects of blood lead level on biochemical and hematological parameters in children with neurological diseases of Western Maharashtra, India. J Basic Clin Physiol Pharmacol. 2014; 25(2): 229-33.
Premarajan KC, Danabalan M, Chandrasekar R, Srinivasa DK. Prevalence of psychiatry morbidity in an urban community of Pondicherry. Indian J Psychiatry. 1993; 35(2): 99-102.
Prince M, Acosta D, Ferri CP, Guerra M, Huang Y, Llibre Rodriguez JJ, Salas A, Sosa AL, Williams JD, Dewey ME, Acosta I, Jotheeswaran AT, Liu Z. Dementia incidence and mortality in middle-income countries, and associations with indicators of cognitive reserve: a 10/66 Dementia Research Group population-based cohort study. Lancet. 2012; 380(9836): 50-8.
Prinja S, Thakur JS, Bhatia SS. Pilot testing of WHO child growth standards in Chandigarh: implications for India?s child health programmes. Bull World Health Organ. 2009; 87(2): 116-22.
PS R, Verma S, Rai L, Kumar P, Pai MV, Shetty J. "Near miss" obstetric events and maternal deaths in a tertiary care hospital: an audit. J Pregnancy. 2013.
Public Health Foundation of India, Institute for Health Metrices and Evaluation. Cost effectiveness of ART and its determinants in Andhra Pradesh and Rajasthan in India Data 2011-14. [Data shared for this analysis]
Public Health Foundation of India. India Cause of Death Estimation Study in Bihar 2011-2014. New Delhi, India: Public Health Foundation of India. [Data shared for this analysis]
Pulickal AS, Fernandez GVJ. Comparison of the prevalence of tuberculosis infection in BCG vaccinated versus non-vaccinated school age children. Indian Pediatr. 2007; 44(5):344–347.
Pullan RL, Smith JL, Jasrasaria R, Brooker SJ. Global numbers of infection and disease burden of soil transmitted helminth infections in 2010. Parasit Vectors. 2014; 7:37. [Data shared for this analysis by the Global Burden of Disease 2010 Soil Transmitted Helminths Expert Group].
Puri A, Yadav I, Jain N. Maternal mortality in an urban tertiary care hospital of north India. J Obstet Gynaecol India. 2011; 61(3): 280-5.
Purohit CK, Sharma R. A study of general health status of persons aged 60 years and above in the Rural Health Training Centre Area, Naila. Indian J Med Res. 1976; 64(2): 202-10.

Table 3: GBD 2016 India data inputs
Purwar M, Ughade S, Bhagat B, Agarwal V, Kulkarni H. Bacterial vaginosis in early pregnancy and adverse pregnancy outcome. J Obstet Gynaecol Res. 2001; 27(4): 175-81.
Qamer S, Shahab T, Alam S, Malik A, Afzal K. Age-specific prevalence of hepatitis B surface antigen in pediatric population of Aligarh, North India. Indian J Pediatr. 2004; 71(11): 965-7.
Radhakrishna S, Frieden TR, Subramani R, Narayanan PR. Value of dual testing for identifying tuberculous infection. Tuberculosis. 2006; 86(1): 47–53.
Radhakrishnan K, Pandian JD, Santhoshkumar T, Thomas SV, Deetha TD, Sarma PS, Jayachandran D, Mohamed E. Prevalence, knowledge, attitude, and practice of epilepsy in Kerala, South India. Epilepsia. 2000; 41(8): 1027-35.
Radhakrishnan S, Balamurugan S. Prevalence of diabetes and hypertension among geriatric population in a rural community of Tamilnadu. Indian J Med Sci. 2013; 67(5-6): 130-8.
Raghunath R, Tripathi RM, Suseela B, Bhalke S, Shukla VK, Puranik VD. Dietary intake of metals by Mumbai adult population. Sci Total Environ. 2006; 356(1-3): 62-8.
Ragunathan L, Kalivaradhan SK, Ramadass S, Nagaraj M, Ramesh K. Helminthic infections in school children in Puducherry, South India. J Microbiol Immunol Infect. 2010; 43(3): 228–32.
Rahmathullah L, Underwood BA, Thulasiraj RD, Milton RC, Ramaswamy K, Rahmathullah R, Babu G. Reduced mortality among children in southern India receiving a small weekly dose of vitamin A. N Engl J Med. 1990; 323(14): 929-35.
Rahmathullah L, Underwood BA, Thulasiraj RD, Milton RC, Ramaswamy K, Rahmathullah R, Babu G. Reduced mortality among children in southern India receiving a small weekly dose of vitamin A. N Engl J Med. 1990; 323(14): 929-35. as it appears in Imdad A, Herzer K, Mayo-Wilson E, Yakoob MY, Bhutta ZA. Vitamin A supplementation for preventing morbidity and mortality in children from 6 months to 5 years of age. Cochrane Database Syst Rev. 2010; CD008524.
Rai S, Mahapatra B, Sircar S, Raj PY, Venkatesh S, Shaukat M, Rewari BB. Adherence to Antiretroviral Therapy and Its Effect on Survival of HIV-Infected Individuals in Jharkhand, India. PLoS One. 2013; 8(6): e66860.
Raina SK, Kashyap V, Bhardwaj AK, Kumar D, Chander V. Prevalence of autism spectrum disorders among children (1-10 years of age) - findings of a mid-term report from Northwest India. J Postgrad Med. 2015; 61(4): 243–6.
Raina SK, Razdan S, Nanda R. Prevalence of neurological disorders in children less than 10 years of age in RS Pura town of Jammu and Kashmir. J Pediatr Neurosci. 2011; 6(2): 103–5.
Raina SK, Razdan S, Pandita KK, Sharma R, Gupta VP, Razdan S. Active epilepsy as indicator of neurocysticercosis in rural northwest India. Epilepsy Res Treat. 2012; 2012: 802747.
Raina VK, Joshi MC, Joshi RD, Singh S, Yadava RL, Kumar A. Filaria situation in the Union Territory of Dadra & Nagar Haveli. J Commun Dis. 1991; 23(3): 182-4.
Raina VK, Joshi MC, Singh S, Joshi RD, Bhattacharjee KK, Kumar A, Verghese T. Epidemiology of Brugia malayi infection and its co-existence with Wuchereria bancrofti in and around Sillaberia PHC, District Midnapur, West Bengal. J Commun Dis. 1990; 22(3): 205-8.
Raina VK, Tripathi VC, Ram R, Kumar A, Verghese T. Status of lymphatic filariasis in some select slum clusters of Delhi. J Commun Dis. 1992; 24(2): 92-6.
Raisler J, Kumari S, Walia I, Singh A. Self-Reported Uterine Prolapse in a Resettlement Colony of North India. J Midwifery Womens Health. 2000; 45(4): 343-50.
Rajagopalan P, Pani S, Das P, Jambulingam P. Malaria in Koraput district of Orissa. Indian J Pediatr. 1989; 56(3): 355–64.

Table 3: GBD 2016 India data inputs
Rajagopalan PK, Das PK, Subramanian S, Vanamail P, Ramaiah KD. Bancroftian filariasis in Pondicherry, south India: 1. Pre-control epidemiological observations. Epidemiol Infect. 1989; 103(3): 685-92.
Rajagopalan PK, Panicker KN, Pani SP. Impact of 50 years of vector control on the prevalence of Brugia malayi in Shertallai area of Kerala state. Indian J Med Res. 1989; 89: 418-25.
Rajapure V, Tirwa R, Poudyal H, Thakur N. Prevalence and risk factors associated with sexually transmitted diseases (STDs) in Sikkim. J Community Health. 2013; 38(1): 156-62.
Rajaratnam J, Abel R, Asokan JS, Jonathan P. Prevalence of anemia among adolescent girls of rural Tamilnadu. Indian Pediatr. 2000; 37(5): 532-6.
Rajaratnam J, Abel R, Ganesan C, Jayaseelan SA. Maternal anaemia: a persistent problem in rural Tamil Nadu. Natl Med J India. 2000; 13(5): 242-5.
Rajasekariah GR, Parab PB, Chandrashekar R, Deshpande L, Subrahmanyam D. Pattern of Wuchereria bancrofti microfilaraemia in young and adolescent school children in Bassein, India, an endemic area for lymphatic filariasis. Ann Trop Med Parasitol. 1991; 85(6): 663-5.
Rajendran G, Panicker KN, Krishnamoorthy K, Sabesan S, Snehlatha, Radhakrishnan R. Current status of filariasis in Chavakad taluk, Trichur district, Kerala. J Commun Dis. 1997; 29(4): 333-43.
Rajendran R, Sunish IP, Mani TR, Munirathinam A, Abdullah SM, Arunachalam N, Satyanarayana K. Impact of two annual single-dose mass drug administrations with diethylcarbamazine alone or in combination with albendazole on Wuchereria bancrofti microfilaraemia and antigenaemia in South India. Trans R Soc Trop Med Hyg. 2004; 98(3): 174-81.
Rajendran R, Sunish IP, Mani TR, Munirathinam A, Abdullah SM, Augustin DJ, Satyanarayana K. The influence of the mass administration of diethylcarbamazine, alone or with albendazole, on the prevalence of filarial antigenaemia. Ann Trop Med Parasitol. 2002; 96(6): 595-602.
Rajendran R, Sunish IP, Mani TR, Munirathinam A, Arunachalam N, Satyanarayana K, Dash AP. Community-based study to assess the efficacy of DEC plus ALB against DEC alone on bancroftian filarial infection in endemic areas in Tamil Nadu, south India. Trop Med Int Health. 2006; 11(6): 851-61.
Rajkumar AP, Thangadurai P, Senthilkumar P, Gayathri K, Prince M, Jacob KS. Nature, prevalence and factors associated with depression among the elderly in a rural south Indian community. Int Psychogeriatr. 2009; 21(2): 372-8.
Rajkumar S, Kumar S, Thara R. Prevalence of dementia in a rural setting: A report from India. Int J Geriatr Psychiatry. 1997; 12(7): 702-7.
Rajkumar S, Padmavathi R, Thara R, Menon MS. Incidence of schizophrenia in an urban community in Madras. Indian J Psychiatry. 1993; 35(1): 18-21.
Rajput R, Rajput M, Singh J, Bairwa M. Prevalence of diabetes mellitus among the adult population in rural blocks of Haryana, India: a community-based study. Metab Syndr Relat Disord. 2012; 10(6): 443-6.
Rajshekhar V, Raghava MV, Prabhakaran V, Oommen A, Muliyil J. Active epilepsy as an index of burden of neurocysticercosis in Vellore district, India. Neurology. 2006; 67(12): 2135-9.
Rama Kumari N, Bhaskara Raju I, Patnaik AN, Barik R, Singh A, Pushpanjali A, Laxmi V, Satya Ramakrishna L. Prevalence of rheumatic and congenital heart disease in school children of Andhra Pradesh, South India. J Cardiovasc Dis Res. 2013; 4(1): 11-4.
Ramachandan TV, Sathish LA. Nationwide indoor 222Rn and 220Rn map for India: a review. J Environ Radioact. 2011; 102(11): 975-86.

Table 3: GBD 2016 India data inputs
Ramachandran A, Mary S, Yamuna A, Murugesan N, Snehalatha C. High prevalence of diabetes and cardiovascular risk factors associated with urbanization in India. Diabetes Care. 2008; 31(5): 893-8.
Ramachandran A, Snehalatha C, Abdul Khader OM, Joseph TA, Viswanathan M. Prevalence of childhood diabetes in an urban population in south India. Diabetes Res Clin Pract. 1992; 17(3): 227-31.
Ramachandran A, Snehalatha C, Kapur A, Vijay V, Mohan V, Das AK, Rao PV, Yajnik CS, Prasanna Kumar KM, Nair JD. High prevalence of diabetes and impaired glucose tolerance in India: National Urban Diabetes Survey. Diabetologia. 2001; 44(9): 1094-101.
Ramachandran A, Snehalatha C, Latha E, Manoharan M, Vijay V. Impacts of urbanisation on the lifestyle and on the prevalence of diabetes in native Asian Indian population. Diabetes Res Clin Pract. 1999; 44(3): 207-13.
Ramachandran A, Snehalatha C, Satyavani K, Latha E, Sasikala R, Vijay V. Prevalence of vascular complications and their risk factors in type 2 diabetes. J Assoc Physicians India. 1999; 47(12): 1152–6.
Ramachandran A, Snehalatha C, Vinitha R, Thayyil M, Kumar CKS, Sheeba L, Joseph S, Vijay V. Prevalence of overweight in urban Indian adolescent school children. Diabetes Res Clin Pract. 2002; 57(3): 185-90.
Ramadevi R, Savithri HS, Devi AR, Bittles AH, Rao NA. An unusual distribution of glucose-6-phosphate dehydrogenase deficiency of south Indian newborn population. Indian J Biochem Biophys. 1994; 31(4): 358–60.
Ramage CS. Monsoon Meteorology. New York: Academic Press, 1971 (International geophysics series ; v. 15).
Ramaiah KD, Das PK, Dhanda V. Estimation of permissible levels of transmission of bancroftian filariasis based on some entomological and parasitological results of a 5-year vector control programme. Acta Trop. 1994; 56(1): 89-96.
Ramaiah KD, Das PK, Vanamail P, Pani SP. Impact of 10 years of diethylcarbamazine and ivermectin mass administration on infection and transmission of lymphatic filariasis. Trans R Soc Trop Med Hyg. 2007; 101(6): 555-63.
Ramaiah KD, Pani SP, Balakrishnan N, Sadanandane C, Das LK, Mariappan T, Rajavel AR, Vanamail P, Subramanian S. Prevalence of bancroftian filariasis & its control by single course of diethyl carbamazine in a rural area in Tamil Nadu. Indian J Med Res. 1989; 89: 184-91.
Ramaiah KD, Ramu K, Kumar KNV, Guyatt H. Epidemiology of acute filarial episodes caused by Wuchereria bancrofti infection in two rural villages in Tamil Nadu, south India. Trans R Soc Trop Med Hyg. 1996; 90(6): 639-43.
Ramaiah KD, Thiruvengadam B, Vanamail P, Subramanian S, Gunasekaran S, Nilamani N, Das PK. Prolonged persistence of residual Wuchereria bancrofti infection after cessation of diethylcarbamazine-fortified salt programme. Trop Med Int Health. 2009; 14(8): 870-6.
Ramaiah KD, Vanamail P, Das PK. Changes in Wuchereria bancrofti infection in a highly endemic community following 10 rounds of mass administration of diethylcarbamazine. Trans R Soc Trop Med Hyg. 2007; 101(3): 250-5.
Ramaiah KD, Vanamail P, Pani SP, Das PK. The prevalences of Wuchereria bancrofti antigenaemia in communities given six rounds of treatment with diethylcarbamazine, ivermectin or placebo tablets. Ann Trop Med Parasitol. 2003; 97(7): 737-41.
Ramaiah KD, Vanamail P, Yuvaraj J, Das PK. Effect of annual mass administration of diethylcarbamazine and albendazole on bancroftian filariasis in five villages in south India. Trans R Soc Trop Med Hyg. 2011; 105(8): 431-7.
Ramaiah KD, Vanamail P. Surveillance of lymphatic filariasis after stopping ten years of mass drug administration in rural communities in south India. Trans R Soc Trop Med Hyg. 2013; 107(5): 293-300.
Ramakrishnan R, Nirmalan PK, Krishnadas R, Thulasiraj RD, Tielsch JM, Katz J, Friedman DS, Robin AL. Glaucoma in a rural population of southern India: the Aravind comprehensive eye survey. Ophthalmology. 2003; 110(8): 1484-90.

Table 3: GBD 2016 India data inputs
Ramakrishnan U, Latham MC, Abel R, Frongillo EA Jr. Vitamin A supplementation and morbidity among preschool children in south India. Am J Clin Nutr. 1995; 61(6): 1295-303.
Raman Kutty V, Joseph A, Soman CR. High prevalence of type 2 diabetes in an urban settlement in Kerala, India. Ethn Health. 1999; 4(4): 231-9.
Raman L, Subbalaxmi P, Vasumathi N, Rawal A, Vasanthi G, Parvathi C, Adinarayana K, Pawashe A, Rao K. Iron and folic acid nutritional status of women in slum. Nutr Report Int. 1989; 73-81.
Ramesh R, Munshi A, Panda SK. Prevalence of hepatitis C virus antibodies in chronic liver disease and hepatocellular carcinoma patients in India . J Gastroenterol Hepatol. 1992; 7(4): 393-5.
Ramola RC. Survey of radon and thoron in homes of Indian Himalaya. Radiat Prot Dosimetry. 2011; 146(1-3): 11-3.
Randomised trial to assess benefits and safety of vitamin A supplementation linked to immunisation in early infancy. WHO/CHD Immunisation-Linked Vitamin A Supplementation Study Group.Lancet. 1998; 352(9136): 1257-63.
Rani A, Singh S, Duggal V. Indoor radon measurements in the dwellings of Punjab and Himachal Pradesh, India. Radiat Prot Dosimetry. 2013; 156(1): 118-24.
Rani PK, Raman R, Gella L, Kulothungan V, Sharma T. Prevalence of Visual Impairment and Associated Risk Factors in Subjects with Type II Diabetes Mellitus: Sankara Nethralaya Diabetic Retinopathy Epidemiology and Molecular Genetics Study (SN-DREAMS, Report 16). Middle East Afr J Ophthalmol. 2012; 19(1): 129-34.
Rani PK, Raman R, Rachapalli SR, Pal SS, Kulothungan V, Sharma T. Prevalence and risk factors for severity of diabetic neuropathy in type 2 diabetes mellitus. Indian J Med Sci. 2010; 64(2): 51–7.
Ranjan S, Passi SJ, Singh SN. Prevalence and risk factors associated with the presence of Soil-Transmitted Helminths in children studying in Municipal Corporation of Delhi Schools of Delhi, India. J Parasit Dis. 2015;39(3): 377–84.
Rao CR, Kamath VG, Shetty A, Kamath A. A study on the prevalence of type 2 diabetes in coastal Karnataka. Int J Diabetes Dev Ctries. 2010; 30(2): 80-5.
Rao GN, Kulkarni GB, Gururaj G, Rajesh K, Subbakrishna DK, Steiner TJ, Stovner LJ. The burden of headache disorders in India: methodology and questionnaire validation for a community-based survey in Karnataka State. J Headache Pain. 2012; 13(7): 543-50.
Rao KV, Radhaiah G, Raju SV. Association of growth status and the prevalence of anaemia in preschool children. Indian J Med Res. 1980;71: 237-46.
Rao PS, Amalraj A. Maternal mortality in southern India. Trop Geogr Med. 1994; 46(5): 302-4.
Rao RSP, Kamath R, Das A, Nair NS, Keshavamurthy. Prevalence of goitre among school children in coastal Karnataka. Indian J Pediatr. 2002; 69(6): 477-9.
Rao VG, Bhat J, Yadav R, Gopalan GP, Nagamiah S, Bhondeley MK, et al. Prevalence of Pulmonary Tuberculosis - A Baseline Survey In Central India. Hill PC, editor. PLoS ONE. 2012;7(8):e43225.
Raoot A, Dewan DK, Dubey AP, Batra RK, Seth S. Measles outbreak in high risk areas of Delhi: epidemiological investigation and laboratory confirmation. Indian J Pediatr. 2016; 83(3): 200-8.
Rapid Assessment of Avoidable Blindness (RAAB) Repository, Health Information Services. India Rapid Assessment of Avoidable Blindness Repository 1995-2016. Available from: http://raabdata.info/repository/
Rath AD, Pett I, eds. Approaches to Increasing the Use of Insecticide Treated Mosquito Nets in Orissa, India. New Delhi, India: British Council Division; 1997.

Table 3: GBD 2016 India data inputs
Rath CP, Akki A, Patil SV, Kalyanshettar SS. Seroprevalence of hepatitis A virus antibody in Bijapur, Karnataka. Indian Pediatr. 2011; 48(1): 71-3.
Rath RN, Mohapatra BN, Das B. Detection of a new focus of Brugia malayi infection in Orissa. J Commun Dis. 1989; 21(1): 39-40.
Ratho R, Mishra B, Singh T, Rao P, Kumar R. Measles outbreak in a migrant population. Indian J Pediatr. 2005; 72(10): 893-4.
Rathod SD, De Silva MJ, Ssebunnya J, Breuer E, Murhar V, Luitel NP, et al. Treatment Contact Coverage for Probable Depressive and Probable Alcohol Use Disorders in Four Low- and Middle-Income Country Districts: The PRIME Cross-Sectional Community Surveys. Fischer G, editor. PLOS One. 2016;11(9):e0162038.
Rathod SD, Nadkarni A, Bhana A, Shidhaye R. Epidemiological features of alcohol use in rural India: a population-based cross-sectional study. BMJ Open. 2015; 5(12): e009802.
Rathore S, Jamwal A, Gupta V. Herpes simplex virus type 2: Seroprevalence in antenatal women. Indian J Sex Transm Dis. 2010; 31(1): 11–5.
Ravikumar P, Bhansali A, Ravikiran M, Bhansali S, Walia R, Shanmugasundar G, Thakur JS, Kumar Bhadada S, Dutta P. Prevalence and risk factors of diabetes in a community-based study in North India: the Chandigarh Urban Diabetes Study (CUDS). Diabetes Metab. 2011; 37(3): 216-21.
Ravindran B, Sahoo PK, Dash AP. Lymphatic filariasis and malaria: concomitant parasitism in Orissa, India. Trans R Soc Trop Med Hyg. 1998; 92(1): 21-3.
Rawat CM, Garg SK, Singh JV, Bhatnagar M, Chopra H, Bajpai SK. Prevalence of anaemia among adolescent girls in rural area of District Meerut, U.P. Indian J Public Health. 2001; 45(1): 24-6.
Ray K, Bala M, Bhattacharya M, Muralidhar S, Kumari M, Salhan S. Prevalence of RTI/STI agents and HIV infection in symptomatic and asymptomatic women attending peripheral health set-ups in Delhi, India. Epidemiol Infect. 2008; 136(10): 1432-40.
Ray S, Mallik S, Munsi A, Mitra S, Baur B, Kumar S. Epidemiological study of measles in slum areas of Kolkata. Indian J Pediatr. 2004; 71(7): 583-6.
Ray SK, Mallick S, Kumar S, Biswas B. A rapid assessment of anaemia in pregnancy in West Bengal with special reference to care seeking behaviour of mothers. Indian J Public Health. 2000; 44(2): 58-64.
Razdan S, Kaul RL, Motta A, Kaul S, Bhatt RK. Prevalence and pattern of major neurological disorders in rural Kashmir (India) in 1986. Neuroepidemiology. 1994; 13(3): 113-9.
Razdan S, Koul RL, Motta A, Kaul S. Cerebrovascular disease in rural Kashmir, India. Stroke. 1989; 20(12): 1691–3.
Reddaiah VP, Kapoor SK. Socio-biological factors in underfive deaths in a rural area. Indian J Pediatr. 1992; 59(5): 567-71.
Reddaiah VP, Raj PP, Ramachandran K, Nath LM, Sood SK, Madan N, Rusia U. Supplementary iron dose in pregnancy anemia prophylaxis. Indian J Pediatr. 1989; 56(1): 109-14.
Reddy KK, Rao AP, Reddy TP. Socioeconomic status and the prevalence of coronary heart disease risk factors. Asia Pac J Clin Nutr. 2002; 11(2): 98-103.
Reddy SG, Reddy RR, Bronkhorst EM, Prasad R, Ettema AM, Sailer HF, Bergé SJ. Incidence of cleft Lip and palate in the state of Andhra Pradesh, South India. Indian J Plast Surg. 2010; 43(2): 184-9.

Table 3: GBD 2016 India data inputs
Reddy YS, Y A, Ramalaksmi BA, Kumar BD. Lead and trace element levels in placenta, maternal and cord blood: a cross-sectional pilot study. J Obstet Gynaecol Res. 2014; 40(12): 2184-90.
Reesu R, Bhattacharya D, Chaaithanya IK, Muruganandam N, Bharadwaj AP, Singhania M, Sugunan AP, Vijayachari P. Emergence of an unusual genotype of rotavirus in andaman and nicobar islands, India. Intervirology. 2013; 56(2): 134-9.
Regu K, Rajendran R, Ali MKS, Koya SM, Dhariwal AC, Lal S. Decline of brugian filariasis in Cherthala taluk, Alappuzha district, Kerala. J Commun Dis. 2005; 37(3): 209-18.
Regu K, Showkath Ali MK, Rajendran R, Koya SM, Ganesh B, Dhariwal AC, Lal S. Mass drug administration against lymphatic filariasis: Experiences from Kozhikode district of Kerala State. J Commun Dis. 2006; 38(4): 333-8.
Reuben R, Rajendran R, Sunish IP, Mani TR, Tewari SC, Hiriyan J, Gajanana A. Annual single-dose diethylcarbamazine plus ivermectin for control of bancroftian filariasis: comparative efficacy with and without vector control. Ann Trop Med Parasitol. 2001; 95(4): 361-78.
Reynolds SJ, Risbud AR, Shepherd ME, Zenilman JM, Brookmeyer RS, Paranjape RS, Divekar AD, Gangakhedkar RR, Ghate MV, Bollinger RC, Mehendale SM. Recent herpes simplex virus type 2 infection and the risk of human immunodeficiency virus type 1 acquisition in India. J Infect Dis. 2003; 187(10): 1513-21.
Risbud AR, Prasad SR, Mehendale SM, Mawar N, Shaikh N, Umrani UB, Bedekar SS, Banerjee K. Measles outbreak in a tribal population of Thane district, Maharashtra. Indian Pediatr. 1994; 31(5): 543-51.
Ritchie GE, Kengne AP, Joshi R, Chow C, Neal B, Patel A, Zoungas S. Comparison of near-patient capillary glucose measurement and a risk assessment questionnaire in screening for type 2 diabetes in a high-risk population in rural India. Diabetes Care. 2011; 34(1): 44–9.
Rogawski ET, Westreich D, Becker-Dreps S, Adair LS, Sandler RS, Sarkar R, Kattula D, Ward HD, Meshnick SR, Kang G. The effect of early life antibiotic exposures on diarrheal rates among young children in Vellore, India. Pediatr Infect Dis J. 2015; 34(6): 583-8.
Roy A, Ansari MA, Biswas S, Kabilan L. Comparison of parasitological and serological data in evaluating malaria. J Commun Dis. 1997; 29(1): 63-5.
Roy A, Ansari MA, Kabilan L. A longitudinal study of sero-reactivity to Plasmodium falciparum antigen in children and adult living in an endemic area of U.P. Indian J Malariol. 1998; 35(2): 48-56.
Roy A, Bellinger D, Hu H, Schwartz J, Ettinger AS, Wright RO, Bouchard M, Palaniappan K, Balakrishnan K. Lead exposure and behavior among young children in Chennai, India. Environ Health Perspect. 2009; 117(10): 1607-11.
Roy A, Ettinger AS, Hu H, Bellinger D, Schwartz J, Modali R, Wright RO, Palaniappan K, Balakrishnan K. Effect modification by transferrin C2 polymorphism on lead exposure, hemoglobin levels, and IQ. Neurotoxicology. 2013; 38: 17-22.
Roy A, Tyagi P, Sharma SK. Serological appraisal of malaria status in tribal area of Orissa, India. Indian J Malariol. 2001; 38(3-4): 84-90.
Roy KK, Baruah J, Kumar S, Malhotra N, Deorari AK, Sharma JB. Maternal antenatal profile and immediate neonatal outcome in VLBW and ELBW babies. Indian J Pediatr. 2006; 73(8): 669-73.
Rudra SK, Chandra G. Comparative epidemiological studies on lymphatic filariasis, between tribal and non-tribal populations of Bankura district, West Bengal, India. Ann Trop Med Parasitol. 2000; 94(4): 365-72.
Rudra SK, Sukul NC. Prevalence and treatment of bancroftian filariasis in the rural population of Bankura district, West Bengal, India. Int J Child Adolesc Health. 2014; 415–26.

Table 3: GBD 2016 India data inputs
Ruikar MM, Pratinidhi AK. Physical wife abuse in an urban slum of Pune, Maharastra. Indian J Public Health. 2008; 52(4): 215-7.
Rusia U, Gupta S, Agarwal N, Singh K, Sikka M, Madan N. Efficacy of the new program of iron supplementation in pregnancy in India. Indian J Hematol Blood Transfus. 1999; 17(4): 87-91.
Rusia U, Madan N, Agarwal N, Sikka M, Sood SK. Effect of maternal iron deficiency anaemia on foetal outcome. Indian J Pathol Microbiol. 1995; 38(3): 273-9.
Rustagi N, Uppal Y, Taneja DK. Screening for visual impairment: outcome among schoolchildren in a rural area of Delhi. Indian J Ophthalmol. 2012; 60(3): 203-6.
S Mayurnath, DS Anantharaman, GVJ Baily, MP Radhamani, RS Vallishayee, P Venkataraman, SP Tripathy. Tuberculosis prevalence survey in Kashmir valley. Indian J Med Res. 1984; 80(2): 129-40.
S Singh, VK Chadha, RK Srivastava, Lakshminarayana, V Magesh, P Suganthi, G Umadevi, J Gupta, J Ahmed. Prevalence & annual risk of tuberculous infection among school children in Bangalore rural district. NTI Bulletin. 2006; 42(3 & 4): 68-73.
Sachdev HPS, Osmond C, Fall CHD, Lakshmy R, Ramji S, Dey Biswas SK, Prabhakaran D, Tandon N, Reddy KS, Barker DJP, Bhargava SK. Predicting adult metabolic syndrome from childhood body mass index: follow-up of the New Delhi birth cohort. Arch Dis Child. 2009; 94(10): 768-74.
Sachdeva JS, Singh S, Sidhu BS, Goyal RK, Singh J. An epidemiological study of psychiatric disorders in rural Faridkot (Punjab). Indian J Psychiatry. 1986; 28(4): 317-23.
Sachdeva S, Alam S, Beig FK, Khan Z, Khalique N. Determinants of vitamin A deficiency amongst children in Aligarh District, Uttar Pradesh. Indian Pediatr. 2011; 48(11): 861-6.
Sachdeva S, Nanda S, Bhalla K, Sachdeva R. Gross congenital malformation at birth in a government hospital. Indian J Public Health. 2014; 58(1): 54-6.
Sadikot SM, Nigam A, Das S, Bajaj S, Zargar AH, Prasannakumar KM, Sosale A, Munichoodappa C, Seshiah V, Singh SK, Jamal A, Sai K, Sadasivrao Y, Murthy SS, Hazra DK, Jain S, Mukherjee S, Bandyopadhay S, Sinha NK, Mishra R, Dora M, Jena B, Patra P, Goenka K. The burden of diabetes and impaired glucose tolerance in India using the WHO 1999 criteria: prevalence of diabetes in India study (PODIS). Diabetes Res Clin Pract. 2004; 66(3): 301-7.
Sagili H, Pramya N, Prabhu K, Mascarenhas M, Reddi Rani P. Are teenage pregnancies at high risk? A comparison study in a developing country. Arch Gynecol Obstet. 2012; 285(3): 573-7.
Saha MR, Sen D, Datta P, Datta D, Pal SC. Role of rotavirus as the cause of acute paediatric diarrhoea in Calcutta. Trans R Soc Trop Med Hyg. 1984; 78(6): 818-20.
Saha S P, Bhattachrya S, Roy B K, Basu A, Roy T, Maity B, Das S K. A prospective incidence study of epilepsy in a rural community of West-Bengal, India. Neurology Asia 2008; 13: 41-8.
Saha SP, Bhattacharya S, Das SK, Maity B, Roy T, Raut DK. Epidemiological study of neurological disorders in a rural population of Eastern India. J Indian Med Assoc. 2003; 101(5): 299-304.
Sahoo PK, Geddam JJ, Satapathy AK, Mohanty MC, Ravindran B. Bancroftian filariasis: prevalence of antigenaemia and endemic normals in Orissa, India. Trans R Soc Trop Med Hyg. 2000; 94(5): 515-7.
Sahu SS, Jambulingam P, Vijayakumar T, Subramanian S, Kalyanasundaram M. Impact of alphacypermethrin treated bed nets on malaria in villages of Malkangiri district, Orissa, India. Acta Trop. 2003; 89(1): 55-66.

Table 3: GBD 2016 India data inputs
Sahu SS, Vijayakumar T, Kalyanasundaram M, Subramanian S, Jambulingam P. Impact of lambdacyhalothrin capsule suspension treated bed nets on malaria in tribal villages of Malkangiri district, Orissa, India. Indian J Med Res. 2008; 128(3): 262-70.
Saikia L, Nath R, Saikia NJ, Choudhury G, Sarkar M. A diphtheria outbreak in Assam, India. Southeast Asian J Trop Med Public Health. 2010; 41(3): 647-52.
Saini N, Srinivasan R, Chawla Y, Sharma S, Chakraborti A, Rajwanshi A. Telomerase activity, telomere length and human telomerase reverse transcriptase expression in hepatocellular carcinoma is independent of hepatitis virus status . Liver Int. 2009; 29(8): 1162-70.
Saleem S, McClure EM, Goudar SS, Patel A, Esamai F, Garces A, Chomba E, Althabe F, Moore J, Kodkany B, Pasha O, Belizan J, Mayansyan A, Derman RJ, Hibberd PL, Liechty EA, Krebs NF, Hambidge KM, Buekens P, Carlo WA, Wright LL, Koso-Thomas M, Jobe AH, Goldenberg RL, on behalf of the Global Network Maternal Newborn Health Registry Study Investigators. A prospective study of maternal, fetal and neonatal deaths in low- and middle-income countries. Bull World Health Organ. 2014; 92(8): 605-12.
Saluja T, Sharma SD, Gupta M, Kundu R, Kar S, Dutta A, et al. A multicenter prospective hospital-based surveillance to estimate the burden of rotavirus gastroenteritis in children less than five years of age in India. Vaccine. 2014;32 Suppl 1:A13-19.
Salve H, Gupta V, Palanivel C, Yadav K, Singh B. Prevalence of knee osteoarthritis amongst perimenopausal women in an urban resettlement colony in South Delhi. Indian J Public Health. 2010; 54(3): 155-7.
Salve H, Kumar R, Sinha S, Krishnan A. Suicide an emerging public health problem: evidence from rural Haryana, India. Indian J Public Health. 2013; 57(1): 40-2.
Samal SK, Khuntia HK, Nanda PK, Satapathy CS, Nayak SR, Sarangi AK, Sahoo N, Pattnaik SK, Chhotray GP, Pal BB. Incidence of bacterial enteropathogens among hospitalized diarrhea patients from Orissa, India. Jpn J Infect Dis. 2008; 61(5): 350-5.
Samuel P, Antonisamy B, Raghupathy P, Richard J, Fall CHD. Socio-economic status and cardiovascular risk factors in rural and urban areas of Vellore, Tamilnadu, South India. Int J Epidemiol. 2012; 41(5): 1315-27.
Sandesh K, Varghese T, Harikumar R, Beena P, Sasidharan VP, Bindu CS, Tony J, Harish K, Sunilkumar K, Ramachandran TM. Prevalence of Hepatitis B and C in the normal population and high risk groups in north Kerala. Trop Gastroenterol. 2006; 27(2): 80-3.
Sangath, Columbia University, Alcohol Research Group, Public Health Institute, University of California San Francisco, London School of Hygiene and Tropical Medicine. Soryacher Asar Ani Hacher Amcho Shodh (SAAHAS) Goa Alcohol Use Study 2004-2008. Goa, India: Sangath.
Sankar J, Singhi P, Bansal A, Ray P, Singhi S. Role of dexamethasone and oral glycerol in reducing hearing and neurological sequelae in children with bacterial meningitis. Indian Pediatr. 2007; 44(9): 649-56.
Sankar R, Pulger T, Rai B, Gomathi S, Gyatso TR, Pandav CS. Epidemiology of endemic cretinism in Sikkim, India. Indian J Pediatr. 1998; 65(2): 303-9.
Sankar R, Pulger T, Rai TB, Gomathi S, Pandav CS. Iodine deficiency disorders in school children of Sikkim. Indian J Pediatr. 1994; 61(4): 407-14.
Santha T, Garg R, Frieden T, Chandrasekaran V, Subramani R, Gopi P, Selvakumar N, Ganapathy S, Charles N, Rajamma J, others. Risk factors associated with default, failure and death among tuberculosis patients treated in a DOTS programme in Tiruvallur District, South India, 2000. Int J Tuberc Lung Dis. 2002; 6(9): 780–788.

Table 3: GBD 2016 India data inputs
Sanwalka N, Khadilkar A, Chiplonkar S, Khatod K, Phadke N, Khadilkar V. Vitamin D receptor gene polymorphisms and bone mass indices in post-menarchal Indian adolescent girls. J Bone Miner Metab. 2013; 31(1): 108-15.
Saravanan S, Kalyani V, Vijayarani MP, Jayakodi P, Felix J, Arunmozhi P, Krishnan V, Sampath Kumar P. Caries prevalence and treatment needs of rural school children in Chidambaram Taluk, Tamil Nadu, South India. Indian J Dent Res. 2008; 19(3): 186-90.
Saravanan S, Velu V, Kumarasamy N, Shankar EM, Nandakumar S, Murugavel KG, Balakrishnan P, Solomon SS, Solomon S, Thyagarajan SP. The prevalence of hepatitis B virus and hepatitis C virus infection among patients with chronic liver disease in South India. Int J Infect Dis. 2008; 12(5): 513-8.
Saravanan S, Velu V, Nandakumar S, Madhavan V, Shanmugasundaram U, Murugavel KG, Balakrishnan P, Kumarasamy N, Solomon S, Thyagarajan SP. Hepatitis B virus and hepatitis C virus dual infection among patients with chronic liver disease. J Microbiol Immunol Infect. 2009; 42(2): 122-8.
Sarin A. Severe anemia of pregnancy, recent experience. Int J Gynaecol Obstet. 1995; 50(Suppl 2): 45-49.
Sarin AR, Singla P, Kaur H. Maternal mortality -- aetiological factors: analytic study from a teaching hospital of Punjab. Indian J Matern Child Health. 1992; 3(3): 69-73.
Sarin SK, Thakur V, Guptan RC, Saigal S, Malhotra V, Thyagarajan SP, Das BC. Profile of hepatocellular carcinoma in India: an insight into the possible etiologic associations. J Gastroenterol Hepatol. 2001; 16(6): 666-73.
Sarkar S, Mohanty B, Basu S. Iodine deficiency in school going children of Pondicherry. Indian J Pediatr. 2007; 74(8): 731-4.
Sarkar S, Patra C, Dasgupta MK, Nayek K, Karmakar PR. Prevalence of congenital anomalies in neonates and associated risk factors in a tertiary care hospital in eastern India. J Clin Neonatol. 2013; 2(3): 131-4.
Sarkar S, Sinha VK, Praharaj SK. Depressive disorders in school children of suburban India: an epidemiological study. Soc Psychiatry Psychiatr Epidemiol. 2012; 47(5): 783-8.
Sarkhel S, Sinha VK, Arora M, Desarkar P. Prevalence of conduct disorder in schoolchildren of Kanke. Indian J Psychiatry. 2006; 48(3): 159-64.
Sarma KV, Naidu AN. Anemia in children. Indian Pediatr. 1984; 21(4): 295-8.
Sarma RV, Vallishayee RS, Mayurnath S, Narayanan PR, Radhamani MP, Tripathy SP. Prevalence survey of filariasis in two villages in Chingleput district of Tamil Nadu. Indian J Med Res. 1987; 85: 522-30.
Sartorius N, Jablensky A, Korten A, Ernberg G, Anker M, Cooper JE, Day R. Early manifestations and first-contact incidence of schizophrenia in different cultures: a preliminary report on the initial evaluation phase of the WHO Collaborative Study on determinants of outcome of severe mental disorders. Psychol Med. 1986; 16(04): 909–28.
Sathyanarayana Rao TS, Darshan MS, Tandon A, Raman R, Karthik KN, Saraswathi N, Das K, Harsha GT, Krishna VST, Ashok NC. Suttur study: An epidemiological study of psychiatric disorders in south Indian rural population. Indian J Psychiatry. 2014; 56(3): 238–45.
Satija A, Hu FB, Bowen L, Bharathi AV, Vaz M, Prabhakaran D, et al. Dietary patterns in India and their association with obesity and central obesity. Public Health Nutr. 2015;18(16):3031-41.
Satoskar A, Ray V. Prevalence of hepatitis B surface antigen (HBsAg) in blood donors from Bombay. Trop Geogr Med. 1992; 44(1-2): 119-21.
Satpathy SK, Chakraborty AK. Epidemiological study of measles in Singur, West Bengal. J Commun Dis. 1990; 22(1): 23-6.
Satyanarayana K, Pradhan DR, Ramnath T, Rao NP. Anemia and physical fitness of school children of rural Hyderabad. Indian Pediatr. 1990; 27(7): 715-21.

Table 3: GBD 2016 India data inputs
Sauvaget C, Ramadas K, Thomas G, Thara S, Sankaranarayanan R. Prognosis criteria of casual systolic and diastolic blood pressure values in a prospective study in India. J Epidemiol Community Health. 2010; 64(4): 366–72.
Sawaithul VK, Ukey PM, Bobhate SK. Prevalence of HIV infection among persons attending voluntary counseling and testing center, Nagpur. Biomed Res. 2006; 17: 201–4.
Sawant P, Rathi PM, Upadhyaya A. Hepatitis B subtypes and hepatitis C genotypes in cirrhosis in western India: results of a pilot study. J Assoc Physicians India. 1999; 47(6): 580-3.
Saxena DDK, Singh C, Murthy RC, Mathur N, Chandra SV. Blood and placental lead levels in an Indian city: a preliminary report. Arch Environ Health. 1994; 49(2): 106-10.
Saxena N, Nayar D, Kapil U. Prevalence of underweight, stunting and wasting. Indian Pediatr. 1997; 34(7): 627-31.
Sazawal S, Dhingra U, Dhingra P, Hiremath G, Kumar J, Sarkar A, Menon VP, Black RE. Effects of fortified milk on morbidity in young children in north India: community based, randomised, double masked placebo controlled trial. BMJ. 2007; 334(7585): 140.
Schensul SL, Hawkes S, Saggurti N, Verma RK, Narvekar SS, Nastasi BK, Burleson JA, Risbud A. Sexually transmitted infections in men in Mumbai slum communities: the relationship of prevalence to risk behavior. Sex Transm Dis. 2007; 34(7): 444–50.
Schneider JA, Lakshmi V, Dandona R, Kumar GA, Sudha T, Dandona L. Population-based seroprevalence of HSV-2 and syphilis in Andhra Pradesh state of India. BMC Infect Dis. 2010; 10:59.
Scott BE, Curtis V, Rabie T. What Motivates Handwashing in Kerala? A Re-analysis of the Formative Research Data. Mumbai, India: Indian Market Research Bureau; 2003.
Scott W, Mathew NT. A Development Monitoring Service at the Local Level: Monitoring Change in Kerala: The First Five Years. Geneva, Switzerland: United Nations Research Institute for Social Development; 1985.
Seby K, Chaudhury S, Chakraborty R. Prevalence of psychiatric and physical morbidity in an urban geriatric population. Indian J Psychiatry. 2011; 53(2): 121-7.
Sehgal A, Telang S, Passah SM, Jyothi MC. Maternal and Neonatal Profile and Immediate Outcome in Extremely Low Birth Weight Babies in Delhi. Trop Doct. 2004; 34(3): 165-8.
Sen B, Nandi DN, Mukherjee SP, Mishra DC, Banerjee G, Sarkar S. Psychiatric morbidity in an urban slum-dwelling community. Indian J Psychiatry. 1984; 26(3): 185–93.
Sethi S, Sharma K, Dhaliwal LK, Banga SS, Sharma M. Declining trends in syphilis prevalence among antenatal women in northern India: a 10-year analysis from a tertiary healthcare centre. Sex Transm Infect. 2007; 83(7): 592.
Sethi V, Goindi G, Kapil U. Prevalence of anemia amongst primary school age children (6-11 years) in National Capital Territory of Delhi. Indian J Pediatr. 2003; 70(6): 519-20.
Sgaier SK, Mony P, Jayakumar S, McLaughlin C, Arora P, Kumar R, Bhatia P, Jha P. Prevalence and correlates of Herpes Simplex Virus-2 and syphilis infections in the general population in India. Sex Transm Infect. 2011; 87(2): 94–100.
Shah A, Afzal M. Prevalence of diabetes and hypertension and association with various risk factors among different Muslim populations of Manipur, India. J Diabetes Metab Disord. 2013; 12(1): 52.
Shah AS, Nisarga R, Ravi Kumar KL, Hubler R, Herrera G, Kilgore PE. Establishment of population-based surveillance for invasive pneumococcal disease in Bangalore, India. Indian J Med Sci. 2009; 63(11): 498–507.
Shah BD, Dwivedi LK. Causes of neonatal deaths among tribal women in Gujarat, India. Popul Res Policy Rev. 2011; 30(4): 517-36.

Table 3: GBD 2016 India data inputs
Shah C, Sheth NR, Solanki B, Shah N. To assess the prevalence of impaired glucose tolerance and impaired fasting glucose in Western Indian population. J Assoc Physicians India. 2013; 61(3): 179–84.
Shah MS, Khalique N, Khan Z. Determinants of childhood mortality. Indian J Prev Soc Med. 2011; 42(2): 118-22.
Shah P, Shah S, Kutty RV, Modi D. Changing epidemiology of maternal mortality in rural India: time to reset strategies for MDG-5. Trop Med Int Health. 2014; 19(5): 568-75.
Shah PA, Shapoo SF, Koul RK, Khan MA. Prevalence of epilepsy in school-going children (6-18 years) in Kashmir Valley of North-west India. J Indian Med Assoc. 2009; 107(4): 216-8.
Shah SN, Bakash A, Rauf A, Muzzafar A, Zuthshi ML. Incidence of iron deficiency anaemia in rural population of Kashmir. Indian J Public Health. 1982; 26(3): 144-54.
Shailee F, Girish MS, Kapil RS, Nidhi P. Oral health status and treatment needs among 12- and 15-year-old government and private school children in Shimla city, Himachal Pradesh, India. J Int Soc Prev Community Dent. 2013; 3(1): 44-50.
Shaji S, Bose S, Verghese A. Prevalence of dementia in an urban population in Kerala, India. Br J Psychiatry. 2005; 186: 136-40.
Shaji S, Promodu K, Abraham T, Roy KJ, Verghese A. An epidemiological study of dementia in a rural community in Kerala, India. Br J Psychiatry. 1996; 168(6): 745-9.
Shaji S, Verghese A, Promodu K, George B, Shibu VP. Prevalence of priority psychiatric disorders in a rural area in kerala. Indian J Psychiatry. 1995; 37(2): 91–6.
Shakir Khan M, Naqvi AH, Azam A. Study of indoor radon and its progeny levels in rural areas of North India using LR-115 plastic track detectors. Radiat Meas. 2008; 43: S385-8.
Sharda AJ, Sharda J, Mathur LK. Oral Health Behavior and its Relationship with Dental Caries Status and Periodontal Status among 12-13 Year Old School Children in Udaipur, India. Oral Health Dent Manag. 2013; 12(4): 237-42.
Sharma A, Prasad K, Rao KV. Identification of an appropriate strategy to control anemia in adolescent girls of poor communities. Indian Pediatr. 2000; 37(3): 261-7.
Sharma BS, Kumar MG, Chandel R. Prevalence of asthma in urban school children in Jaipur, Rajasthan. Indian Pediatr. 2012; 49(10): 835-6.
Sharma GK, Rao CK, Sharma SP, Sundaram RM, Ghosh TK, Raina VK, Rao PK, Das M. Relative impact of integrated vector control strategy vis-a-vis conventional control strategy on bancroftian filariasis in Pondicherry. J Commun Dis. 1986; 18(4): 267-75.
Sharma JB, Soni D, Murthy NS, Malhotra M. Effect of dietary habits on prevalence of anemia in pregnant women of Delhi. J Obstet Gynaecol Res. 2003; 29(2): 73-8.
Sharma MK, Bhatia V, Swami H. Outbreak of measles amongst vaccinated children in a slum of Chandigarh. Indian J Med Sci. 2004; 58(2): 47-53.
Sharma NC, Banavaliker JN, Ranjan R, Kumar R. Bacteriological and epidemiological characteristics of diphtheria cases in and around Delhi – a retrospective study. Indian J Med Res. 2007; 126(6): 545-52.
Sharma NC, Mandal PK, Dhillon R, Jain M. Changing profile of Vibrio cholerae O1, O139 in Delhi & its periphery (2003-2005). Indian J Med Res. 2007; 125(5): 633-40.
Sharma P, Malhotra C, Taneja DK, Saha R. Problems related to menstruation amongst adolescent girls. Indian J Pediatr. 2008; 75(2): 125-9.

Table 3: GBD 2016 India data inputs
Sharma P, Sharma A, Sehgal R, Malla N, Khurana S. Genetic diversity of Cryptosporidium isolates from patients in North India. Int J Infect Dis. 2013; 17(8): e601-5.
Sharma R, ed. Epidemiology of Musculoskeletal Conditions in India 2007-2010. New Delhi, India: Indian Council of Medical Research; 2012.
Sharma R. An epidemiological study of measles epidemic in district Bhilwara, Rajasthan. J Commun Dis. 1988; 20(4): 301-11.
Sharma RR, Cheema R, Vajpayee M, Rao U, Kumar S, Marwaha N, Agnihotri SK. Prevalence of markers of transfusion transmissible diseases in voluntary and replacement blood donors. Natl Med J India. 2004; 17(1): 19-21.
Sharma RS, Kaushic VK, Johri SP, Ray SN. An epidemiological investigation of measles outbreak in Alwar--Rajasthan. J Commun Dis. 1984; 16(4): 299-303.
Sharma S, Sharma M, Rathaur S. Bancroftian filariasis in the Varanasi region of north India: an epidemiological study. Ann Trop Med Parasitol. 1999; 93(4): 379-87.
Sharma S, Singh MM. Prevalence of mental disorders: An epidemiological study in Goa. Indian J Psychiatry. 2001; 43(2): 118–26.
Sharma SK, Chattopadhyay R, Chakrabarti K, Pati SS, Srivastava VK, Tyagi PK, Mahanty S, Misra SK, Adak T, Das BS, Chitnis CE. Epidemiology of malaria transmission and development of natural immunity in a malaria-endemic village, San Dulakudar, in Orissa state, India. Am J Trop Med Hyg. 2004; 71(4): 457-65.
Sharma SK, Goel A, Gupta SK, Mohan K, Sreenivas V, Rai SK, et al. Prevalence of tuberculosis in Faridabad district, Haryana State, India. Indian J Med Res. 2015; 141(2):228-35.
Sharma SK, Tyagi PK, Padhan K, Adak T, Subbarao SK. Malarial morbidity in tribal communities living in the forest and plain ecotypes of Orissa, India. Ann Trop Med Parasitol. 2004; 98(5): 459-68.
Sharma SK, Tyagi PK, Padhan K, Upadhyay AK, Haque MA, Nanda N, Joshi H, Biswas S, Adak T, Das BS, Chauhan VS, Chitnis CE, Subbarao SK. Epidemiology of malaria transmission in forest and plain ecotype villages in Sundargarh District, Orissa, India. Trans R Soc Trop Med Hyg. 2006; 100(10): 917-25.
Sharma SK, Tyagi PK, Upadhyay AK, Haque MA, Adak T, Dash AP. Building small dams can decrease malaria: a comparative study from Sundargarh District, Orissa, India. Acta Trop. 2008; 107(2): 174-8.
Sharma SK, Upadhyay AK, Haque MA, Padhan K, Tyagi PK, Batra CP, Adak T, Dash AP, Subbarao SK. Effectiveness of mosquito nets treated with a tablet formulation of deltamethrin for malaria control in a hyperendemic tribal area of Sundargarh District, Orissa, India. J Am Mosq Control Assoc. 2006; 22(1): 111-8.
Sharma VK, Khandpur S. Changing patterns of sexually transmitted infections in India. Natl Med J India. 2004; 17(6): 310-9.
Sharma VP, Dev V, Phookan S. Neglected Plasmodium vivax malaria in northeastern States of India. Indian J Med Res. 2015; 141(5): 546-555.
Shatrugna V, Kulkarni B, Kumar PA, Rani KU, Balakrishna N. Bone status of Indian women from a low-income group and its relationship to the nutritional status. Osteoporos Int. 2005; 16(12): 1827-35.
Shekar C, Cheluvaiah MB, Namile D. Prevalence of dental caries and dental fluorosis among 12 and 15 years old school children in relation to fluoride concentration in drinking water in an endemic fluoride belt of Andhra Pradesh. Indian J Public Health. 2012; 56(2): 122-8.

Table 3: GBD 2016 India data inputs
Shekeeb Shahab M, Kumar P, Sharma N, Narang A, Prasad R. Evaluation of oxidant and antioxidant status in term neonates: a plausible protective role of bilirubin. Mol Cell Biochem. 2008; 317(1-2): 51-9.
Shenoi RP, Khandekar RN, Jaykar AV, Raghunath R. Sources of lead exposure in urban slum school children. Indian Pediatr. 1991; 28(9): 1021-7.
Shenoy RK, Rahmah N, Suma TK, Kumaraswami V, Thaslim AX, Gopu RB. Relevance of anti-BmR1 IgG4 antibodies in children from an area endemic for Brugia malayi infection in Kerala, India. J Commun Dis. 2009; 41(2): 63-70.
Shenoy RK, Suma TK, Kumaraswami V, Rahmah N, Dhananjayan G, Padma S, Abhilash G, Ramesh C. Preliminary findings from a cross-sectional study on lymphatic filariasis in children, in an area of India endemic for Brugia malayi infection. Ann Trop Med Parasitol. 2007; 101(3): 205-13.
Sher-i-Kashmir Institute of Medical Sciences-Srinagar, Population Health and Occupational Disease, National Heart & Lung Institute, Imperial College London. India - Srinagar Burden of Obstructive Lung Disease Intiative Survey (BOLD) 2010-2011. [Data shared for this analysis]
Shidhaye R, Gangale S, Patel V. Prevalence and treatment coverage for depression: a population-based survey in Vidarbha, India. Soc Psychiatry Psychiatr Epidemiol. 2016; 51(7): 993–1003.
Shikha B, Harsh S, Narayan G. Infant deaths' audit: Contextual factors contributing to Infant deaths in tribal district-Valsad, Gujarat (India). J Res Med Den Sci. 2015; 3(3): 171-5.
Shivaswamy KN, Thappa DM, Jaisankar TJ, Sujatha S. High seroprevalence of HSV-1 and HSV-2 in STD clinic attendees and non-high risk controls: a case control study at a referral hospital in south India. Indian J Dermatol Venereol Leprol. 2005; 71(1): 26-30.
Shivpuri D, Rajesh MS, Jain D. Prevalence and characteristics of migraine among adolescents: a questionnaire survey. Indian Pediatr. 2003; 40(7): 665-9.
Shobha S, Sharada D. Efficacy of twice weekly iron supplementation in anemic adolescent girls. Indian Pediatr. 2003; 40(12): 1186-90.
Shridhar K, Dhillon PK, Bowen L, Kinra S, Bharathi AV, Prabhakaran D, et al. Nutritional profile of Indian vegetarian diets – the Indian Migration Study (IMS). Nutr J. 2014 ;13(1):55.
Shrikhande SN, Joshi SG, Zodpey SP, Saoji AM. Chlamydia trachomatis in pelvic inflammatory disease. Indian J Pathol Microbiol. 1995; 38(2): 181-4.
Shriram AN, Krishnamoorthy K, Sivan A, Saha BP, Kumaraswami V, Vijayachari P. Impact of MDA and the prospects of elimination of the lone focus of diurnally sub periodic lymphatic filariasis in Nicobar Islands, India. Acta Trop. 2014; 133: 93–7.
Shriram AN, Murhekar MV, Ramaiah KD, Sehgal SC. Prevalence of diurnally subperiodic bancroftian filariasis among the Nicobarese in Andaman and Nicobar Islands, India: effect of age and gender. Trop Med Int Health. 2002; 7(11): 949-54.
Shriram AN, Sugunan AP, Murhekar MV, Sehgal SC. Little Andaman Island, a new focus of infection with nocturnally periodic Wuchereria bancrofti. Indian J Med Res. 1996; 166-70.
Shrivastava SR, Ghorpade AG. High prevalence of type 2 diabetes melitus and its risk factors among the rural population of Pondicherry, South India. J Res Health Sci. 2014; 14(4): 258-63.
Shukla RP, Sharma SN, Bhat SK. Malaria outbreak in Bhojpur PHC of district Moradabad, Uttar Pradesh, India. J Commun Dis. 2002; 34(2): 118-23.

Table 3: GBD 2016 India data inputs
Shukla V, Karoli R, Chandra A. A study of newly diagnosed type 2 diabetes mellitus patients from rural areas. J Assoc Physicians India. 2014; 62(8): 682–4.
Siddiqi D, Ghose S, Krishnamurthy MS, Sashidhara AN. Tuberculosis infection rate in a rural population of Bikaner district. Indian J Tuberc. 1996; 43: 91–98.
Sidhu PK, Kaur K, Kumar I, Lata S. Glucose-6-phosphate dehydrogenase deficiency in blood donors: screening by micromethaemoglobin reduction test. Indian J Pathol Microbiol. 2001; 44(1): 23–5.
Sidhu S, Kumari K, Uppal M. Prevalence of anemia in Schedule Caste preschool children of Punjab. Indian J Med Sci. 2002; 56(5): 218-21.
Sidhu S. Incidence of anaemia among scheduled caste pre-school children of Punjab. Indian J Matern Child Health. 1996; 7(3): 76-7.
Silvanus V, Subramanian P. Epidemiological study of mental morbidity in an urban slum community in India for the development of a community mental health programme. Nepal Med Coll J. 2012; 14(1): 13-7.
Sinclair S, Mittal SK, Basu N, Ghai OP, Bhide NK. Hazard from lead to children in Delhi. Indian Pediatr. 1973; 10(1): 13-8.
Singaravelu G, Mahalingam S, Sumathy S. Estimation of different degrees of provocation by DEC (diethyl carbamazine citrate) medication in bancroftian filariasis in Vellore, Tamilnadu. Indian J Exp Biol. 1999; 37(11): 1142-3.
Singh A, Kaur A. Epilepsy in rural Haryana – prevalence and treatment seeking behaviour. J Indian Med Assoc. 1997; 95(2): 37-47.
Singh A. Flooding in Asia Continues to Displace Millions, Death Toll Rising Rapidly. Available from: http://edition.cnn.com/2015/08/07/asia/asia-flooding/index.html
Singh AK, Farag YMK, Mittal BV, Subramanian KK, Reddy SRK, Acharya VN, Almeida AF, Channakeshavamurthy A, Ballal HS, P G, Issacs R, Jasuja S, Kirpalani AL, Kher V, Modi GK, Nainan G, Prakash J, Rana DS, Sreedhara R, Sinha DK, V SB, Sunder S, Sharma RK, Seetharam S, Raju TR, Rajapurkar MM. Epidemiology and risk factors of chronic kidney disease in India - results from the SEEK (Screening and Early Evaluation of Kidney Disease) study. BMC Nephrol. 2013; 14:114.
Singh AK, Jain A, Jain B, Singh KP, Dangi T, Mohan M, Dwivedi M, Kumar R, Kushwaha R a. S, Singh JV, Mishra AC, Chhaddha MS. Viral aetiology of acute lower respiratory tract illness in hospitalised paediatric patients of a tertiary hospital: one year prospective study. Indian J Med Microbiol. 2014; 32(1): 13–8.
Singh AK, Mani K, Krishnan A, Aggarwal P, Gupta SK. Prevalence, awareness, treatment and control of diabetes among elderly persons in an urban slum of delhi. Indian J Community Med. 2012; 37(4): 236-9.
Singh AK, Singh M. Lead decline in the Indian environment resulting from the petrol-lead phase-out programme. Sci Total Environ. 2006; 368(2-3): 686-94.
Singh B, Chandran V, Bandhu HK, Mittal BR, Bhattacharya A, Jindal SK, Varma S. Impact of lead exposure on pituitary-thyroid axis in humans. Biometals. 2000; 13(2): 187–92.
Singh G, Bawa J, Chinna D, Chaudhary A, Saggar K, Modi M, Sander JW. Association between epilepsy and cysticercosis and toxocariasis: a population-based case-control study in a slum in India. Epilepsia. 2012; 53(12): 2203-8.
Singh G, Singh P, Singh I, Rani A, Kaushal S, Avasthi G. Epidemiologic classification of seizures associated with neurocysticercosis: observations from a sample of seizure disorders in neurologic care in India. Acta Neurol Scand. 2006; 113(4): 233–40.
Singh H, Aggarwal R, Singh RL, Naik SR, Naik S. Frequency of infection by hepatitis B virus and its surface mutants in a northern Indian population. Indian J Gastroenterol. 2003; 22(4): 132-7.

Table 3: GBD 2016 India data inputs
Singh J, Bora D, Sachdeva V, Sharma R, Verghese T. Vibrio cholerae O1 and O139 in Less Than Five Years Old Children Hospitalised for Watery Diarrhoea in Delhi, 1993. J Diarrhoeal Dis Res. 1997; 15(1): 3–6.
Singh J, Harit AK, Jain DC, Panda RC, Tewari KN, Bhatia R, Sokhey J. Diphtheria is declining but continues to kill many children: analysis of data from a sentinel centre in Delhi, 1997. Epidemiol Infect. 1999; 123(2): 209-15.
Singh J, Kumar A, Rai RN, Khare S, Jain DC, Bhatia R, Datta KK. Widespread outbreaks of measles in rural Uttar Pradesh, India, 1996: high risk areas and groups. Indian Pediatr. 1999; 36(3): 249-56.
Singh J, Sachdeva V, Bhatia R, Bora D, Jain D, Sokhey J. Endemic Cholera in Delhi, 1995: Analysis of Data from a Sentinel Centre. J Diarrhoeal Dis Res. 1998; 16(2): 66–73.
Singh MP, Nayar S. Magnitude of acute respiratory infections in under five children. J Commun Dis. 1996; 4(28): 273-8.
Singh N, Mishra SS, Singh MP, Sharma VP. Seasonality of Plasmodium vivax and P. falciparum in tribal villages in central India (1987-1995). Ann Trop Med Parasitol. 2000; 94(2): 101-12.
Singh N, Shukla MM, Chand G, Bharti PK, Singh MP, Shukla MK, Mehra RK, Sharma RK, Dash AP. Epidemic of Plasmodium falciparum malaria in Central India, an area where chloroquine has been replaced by artemisinin-based combination therapy. Trans R Soc Trop Med Hyg. 2011; 105(3): 133-9.
Singh NP, Ingle GK, Saini VK, Jami A, Beniwal P, Lal M, Meena GS. Prevalence of low glomerular filtration rate, proteinuria and associated risk factors in North India using Cockcroft-Gault and Modification of Diet in Renal Disease equation: an observational, cross-sectional study. BMC Nephrol. 2009; 10: 4.
Singh PN, Hasan B, Ahmed J et al. Goitre survey in a north Indian village. In: Nagataki S et al., eds. The Thyroid 1988: Proceedings of the International Thyroid Symposium, Tokyo, 13-15 July 1988. Amsterdam, Excerpta Medica, 1988 : 297-300.
Singh RB, Bajaj S, Niaz MA, Rastogi SS, Moshiri M. Prevalence of type 2 diabetes mellitus and risk of hypertension and coronary artery disease in rural and urban population with low rates of obesity. Int J Cardiol. 1998; 66(1): 65-72.
Singh RB, Fedacko J, Pella D, Macejova Z, Ghosh S, de Amit K, Begom R, Tumbis ZA, Haque M, Vajpeyee SK, de Meester F, Sergey C, Agarwalo R, Muthusamy VV, Five City Study Group, Gupta AK. Prevalence and risk factors for prehypertension and hypertension in five Indian cities. Acta Cardiol. 2011; 66(1): 29-37.
Singh S, Bora D, Dhariwal AC, Singh R, Lal S. Lymphatic filariasis in rural areas of Patna District, Bihar. A challenge ahead. J Commun Dis. 2006; 38(2): 160-3.
Singh S, Bora D, Lal S. Lymphatic filariasis in East District, Sikkim. J Commun Dis. 2010; 42(1): 33-7.
Singh S, Bora D, Sharma RC. A study of filarial transmission in a non-endemic area of Pathankot (Punjab). J Commun Dis. 2000; 32(1): 61-4.
Singh S, Dhariwal AC, Bora D, Lal S. Status of lymphatic filariasis in Lucknow District, Uttar Pradesh. J Commun Dis. 2009; 41(1): 39-44.
Singh S, Murthy GV, Thippaiah A, Upadhyaya S, Krishna M, Shukla R, Srikrishna SR. Community based maternal death review: lessons learned from ten districts in Andhra Pradesh, India. Matern Child Health J. 2015; 19(7): 1447-54.
Singh S, Raina VK, Bora D, Dhariwal AC, Lal S. Lymphatic filariasis in Bilaspur district, Chhattisgarh. J Commun Dis. 2005; 37(2): 125-30.
Singh S, Sharma BB, Salvi S, Chhatwal J, Jain KC, Kumar L, Joshi MK, Pandramajal SB, Awasthi S, Bhave S, Rego S, Sukumaran TU, Khatav VA, Singh V, Sharma SK, Sabir M. Allergic rhinitis, rhinoconjunctivitis, and eczema: prevalence and associated factors in children. Clin Respir J. 2016. [Epub ahead of print]

Table 3: GBD 2016 India data inputs
Singh S, Sharma BB, Sharma SK, Sabir M, Singh V, investigators I collaborating. Prevalence and severity of asthma among Indian school children aged between 6 and 14 years: associations with parental smoking and traffic pollution. J Asthma. 2016; 53(3): 238–44.
Singh SP, Picado A, Boelaert M, Gidwani K, Andersen EW, Ostyn B, Meheus F, Rai M, Chappuis F, Davies C, Sundar S. The epidemiology of Leishmania donovani infection in high transmission foci in India. Trop Med Int Health. 2010; 15: 12-20.
Singh V, Sharma BB, Patel V, Poonia S. Clinical profile of pneumonia and its association with rain wetting in patients admitted at a tertiary care institute during pandemic of influenza A (H1N1) pdm09 virus infection. Indian J Chest Dis Allied Sci. 2014; 56(1): 21-6.
Singh VP, Ranjan A, Topno RK, Verma RB, Siddique NA, Ravidas VN, Kumar N, Pandey K, Das P. Estimation of under-reporting of visceral leishmaniasis cases in Bihar, India. Am J Trop Med Hyg. 2010; 82(1): 9-11.
Singh Z, Chadha P. Assessment of DNA damage as an index of genetic toxicity in welding microenvironments among iron-based industries. Toxicol Ind Health. 2016; 32(10): 1817-24.
Singhal PK, Mathur GP, Mathur S, Singh YD. Mortality patterns in under six children in I.C.D.S. urban slum. Indian Pediatr. 1986; 23(8): 617-22.
Singhal PK, Mathur GP, Mathur S, Singh YD. Neonatal morbidity and mortality in ICDS Urban Slums. Indian Pediatr. 1990; 27(5): 485-8.
Singhvi A, Pulimood RB, John TJ, Babu PG, Samuel BU, Padankatti T, Carman RH. The prevalence of markers for hepatitis B and human immunodeficiency viruses, malarial parasites and microfilaria in blood donors in a large hospital in south India. J Trop Med Hyg. 1990; 93(3): 178-82.
Singla PN, Agarwal KN. Studies on normal hemoglobin and hematocrit values in healthy children based on hematinic supplementation. Indian Pediatr. 1981; 18(11): 821-5.
Sinha A, Sazawal S, Kumar R, Sood S, Reddaiah VP, Singh B, Rao M, Naficy A, Clemens JD, Bhan MK. Typhoid fever in children aged less than 5 years. Lancet. 1999; 354(9180): 734-7.
Sinha A, SenGupta S, Guin S, Dutta S, Ghosh S, Mukherjee P, Mukhopadhyay AK, Ramamurthy T, Takeda Y, Kurakawa T, Nomoto K, Nair GB, Nandy RK. Culture-independent real-time PCR reveals extensive polymicrobial infections in hospitalized diarrhoea cases in Kolkata, India. Clin Microbiol Infect. 2013; 19(2): 173–80.
Sinha B, Singla R, Chowdhury R. An epidemiological profile of chronic obstructive pulmonary disease: A community-based study in Delhi. J Postgrad Med. 2016; 63(1):29.
Sinha B, Singla R, Chowdhury R. Community-based study on chronic obstructive pulmonary diseasey in Delhi dataset 2016. [Data shared for this analysis]
Sinharay K, Paul UK, Bhattacharyya AK, Pal SK. Prevalence of diabetic foot ulcers in newly diagnosed diabetes mellitus patients. J Indian Med Assoc. 2012; 110(9): 608-11.
Sivakumar B, Brahmam GN, Madhavan Nair K, Ranganathan S, Vishnuvardhan Rao M, Vijayaraghavan K, Krishnaswamy K. Prospects of fortification of salt with iron and iodine. Br J Nutr. 2001; 167-173.
Sloan N, Durocher J, Aldrich T, Blum J, Winikoff B. What measured blood loss tells us about postpartum bleeding: a systematic review. BJOG. 2010; 117(7): 788-800.
Smith JL, Brooker S. Impact of hookworm infection and deworming on anaemia in non-pregnant populations: a systematic review. Trop Med Int Health. 2010; 15(7): 776–95.

Table 3: GBD 2016 India data inputs
Sohal KS, Sharma TD, Kapil U, Tandon M. Assessment of iodine deficiency disorders in district Hamirpur, Himachal Pradesh. Indian Pediatr. 1998; 35(10): 1008-11.
Sohal KS, Sharma TD, Kapil U, Tandon M. Current status of prevalence of goiter and iodine content of salt consumed in District Solan, Himachal Pradesh. Indian Pediatr. 1999; 36(12): 1253-6.
Sohi D, Walia I, Singh A. Prevalence and treatment of epilepsy in a Chandigarh slum. Bull PGI. 1993; 27: 175-8.
Sonwane BR, Birare SD, Kulkarni PV. Prevalence of seroreactivity among blood donors in rural population. Indian J Med Sci. 2003; 57(9): 405-7.
Sood A, Midha V, Goyal O, Goyal P, Sood N, Sharma SK. Profile of hepatocellular carcinoma in a tertiary care hospital in Punjab in northern India. Indian J Gastroenterol. 2014; 33(1): 35-40.
Sood A, Midha V, Sood N, Bhatia AS, Avasthi G. Incidence and prevalence of ulcerative colitis in Punjab, North India. Gut. 2003; 52(11): 1587-90.
Sood A, Sidhu SS, Midha V, Jyoti D. High seroprevalence of hepatitis C virus and dual infection (hepatitis B and C virus) in non-alcoholic chronic liver disease in north India. J Assoc Physicians India. 1999; 47(2): 205-8.
Sood SK, Ramachandran K, Rani K, Ramalingaswami V, Mathan VI, Ponniah J, Baker SJ. WHO sponsored collaborative studies on nutritional anaemia in India. The effect of parenteral iron administration in the control of anaemia of pregnancy. Br J Nutr. 1979; 42(3): 399-406.
Sophia A, Isaac R, Rebekah G, Brahmadathan K, Rupa V. Risk factors for otitis media among preschool, rural Indian children. Int J Pediatr Otorhinolaryngol. 2010; 74(6): 677-83.
Sosale A, Prasanna Kumar KM, Sadikot SM, Nigam A, Bajaj S, Zargar AH, Singh SK. Chronic complications in newly diagnosed patients with Type 2 diabetes mellitus in India. Indian J Endocrinol Metab. 2014; 18(3): 355–60.
Spector JM, Agrawal P, Kodkany B, Lipsitz S, Lashoher A, Dziekan G, Bahl R, Merialdi M, Mathai M, Lemer C, Gawande A. Improving Quality of Care for Maternal and Newborn Health: Prospective Pilot Study of the WHO Safe Childbirth Checklist Program. PLoS One. 2012; 7(5): e35151.
Sree Chitra Tirunal Institute for Medical Sciences and Technology. India Sree Chitra Tirunal Institute for Medical Sciences and Technology Heart Failure Registry 2001-16. [Data shared for this analysis]
Sreehari U, Razdan RK, Mittal PK, Ansari MA, Rizvi MMA, Dash AP. Impact of Olyset nets on malaria transmission in India. J Vector Borne Dis. 2007; 44(2): 137-44.
Sreenath Reddy M, Yadagiri Reddy P, Rama Reddy K, Eappen KP, Ramachandran TV, Mayya YS. Indoor radon levels in urban Hyderabad area, Andhra Pradesh, India. Radiat Prot Dosimetry. 2008; 132(4): 403-8.
Sridharan SE, Unnikrishnan JP, Sukumaran S, Sylaja PN, Nayak SD, Sarma PS, Radhakrishnan K. Incidence, Types, Risk Factors, and Outcome of Stroke in a Developing Country. Stroke. 2009; 40(4): 1212-8.
Sriharibabu M, Himabindu Y, Kabir Z. Rheumatic heart disease in rural south India: A clinico-observational study. J Cardiovasc Dis Res. 2013; 4(1.0): 25-9.
Srinath S, Girimaji SC, Gururaj G, Seshadri S, Subbakrishna DK, Bhola P, Kumar N. Epidemiological study of child and adolescent psychiatric disorders in urban and rural areas of Bangalore, India. Indian J Med Res. 2005; 122(1): 67-79.
Srinivasa Reddy Y, Pullakhandam R, Radha Krishna KV, Uday Kumar P, Dinesh Kumar B. Lead and essential trace element levels in school children: a cross-sectional study. Ann Hum Biol. 2011; 38(3): 372-7.
Srivastava HC, Chandrashekar P, Kurien G, Sreehari U, Yadav RS. Malaria in seasonal migrant population in Southern Gujarat, India. Trop Biomed. 2011; 28(3): 638-45.

Table 3: GBD 2016 India data inputs
Srivastava HC, Yadav RS. Malaria outbreak in a tribal area of Gujarat state, India. Southeast Asian J Trop Med Public Health. 2000; 31(2): 219-24.
Srivastava PK, Krishnamoorthy K, Govenkar S, Perni S, Dalvi S, Subramanain S, Dhariwal AC, Bhattacharjee J, Dash AP. Elimination of lymphatic filariasis in goa: First successful transmission assessment survey in India. J Commun Dis. 2014; 46(2): 7-16.
Srivastava R, Gupta SK, Mathur VP, Goswami A, Nongkynrih B. Prevalence of dental caries and periodontal diseases, and their association with socio-demographic risk factors among older persons in Delhi, India: a community-based study. Southeast Asian J Trop Med Public Health. 2013; 44(3): 523-33.
St. John's Medical College and Research Institute, Population Health Research Institute, All India Institute of Health Sciences, Monilek Hospital and Research Centre, Government Medical College-Nagpur, KEM Hospital-Mumbai, Sri Ramachandra Medical College-Chennai, Amrita Institute of Medical Sciences, Christian Medical College-Ludhiana, Mediciti Hospital-Hyderabad, SSKM Hospital-Kolkata. India CREATE Registry Data 2001-2005. [Data shared for this analysis]
St. John's Medical College and Research Institute, Population Health Research Institute, Mahatma Gandhi Institute of Medical Sciences, Fortis Escort Hospital. INdian Stroke ProspectIve REgistry (INSPIRE) Data 2009-2014. [Data shared for this analysis]
Stalin P, Senthilvel V, Kanimozhy K, Singh Z, Rajkamal R, Purty AJ. Burden, distribution and impact of domestic accidents in a semi-urban area of coastal Tamil Nadu, India. Int J Inj Contr Saf Promot. 2015; 22(1): 11–5.
State Bureau of Health Intelligence & Vital Statistics, Directorate of Health Services, Department of Health & Family Welfare, Government of Odisha . India - Odisha Medical Certification of Cause of Death Data 2009. Odisha, India: Department of Health & Family Welfare, Government of Odisha.
State Bureau of Health Intelligence & Vital Statistics, Directorate of Health Services, Department of Health & Family Welfare, Government of Odisha . India - Odisha Medical Certification of Cause of Death Data 2010. Odisha, India: Department of Health & Family Welfare, Government of Odisha
State Bureau of Health Intelligence & Vital Statistics, Directorate of Health Services, Department of Health & Family Welfare, Government of Odisha . India - Odisha Medical Certification of Cause of Death Data 2011. Odisha, India: Department of Health & Family Welfare, Government of Odisha.
State Bureau of Health Intelligence & Vital Statistics, Directorate of Health Services, Department of Health & Family Welfare, Government of Odisha . India - Odisha Medical Certification of Cause of Death Data 2012. Odisha, India: Department of Health & Family Welfare, Government of Odisha.
State Bureau of Health Intelligence & Vital Statistics, Directorate of Health Services, Department of Health & Family Welfare, Government of Odisha . India - Odisha Medical Certification of Cause of Death Data 2013. Odisha, India: Department of Health & Family Welfare, Government of Odisha.
State Bureau of Health Intelligence & Vital Statistics, Public Health Department, Government of Maharashtra. Annual Report on Maharashtra Survey of Causes of Deaths Scheme (Rural) 2011. [Report shared for this analysis]
State TB Cell, Commissionerate of Health, Medical Services & Medical Education, Department of Health & Family Welfare, Government of Gujarat, State TB Training & Demonstration Center, Civil Hospital Campus – Ahmedabad. Report on population-based survey to assess prevalence of pulmonary tuberculosis cases in the state of Gujarat, India 2011-2012. Gandhinagar, India: State TB Cell, Department of Health & Family Welfare, Government of Gujarat; 2013.
Stein Z, Belmont L, Durkin M. Mild mental retardation and severe mental retardation compared: experiences in eight less developed countries. Ups J Med Sci Suppl. 1987; 44: 89-96.
Straughn HK, Goldenberg RL, Tolosa JE, Daly S, de Codes J, Festin MR, Limpongsanurak S, Lumbiganon P, Paul VK, Peedicayil A, Purwar M, Sabogal JC, Shenoy S. Birthweight-specific neonatal mortality in developing countries and obstetric practices. Int J Gynaecol Obstet. 2003; 80(1): 71-8.

Table 3: GBD 2016 India data inputs
Subba Ramu MC, Shaikh AN, Muraleedharan TS, Ramachandran TV, Nambi KSV. Environmental Radon Monitoring in India: A Plea for National Effort. In: Kumar S, Reddy AR, editors. Proceedings of the National Conference on Particle Tracks in Solids; 1991 Oct 9-11; Jodhpur, India: Defence Laboratory Jodhpur, Defence Research & Development Organisation; 1991. p 11.
Subbarao SK, Vasantha K, Raghavendra K, Sharma VP, Sharma GK. Anopheles culicifacies: siblings species composition and its relationship to malaria incidence. J Am Mosq Control Assoc. 1988; 4(1): 29-33.
Subramanian S, Manoharan A, Sahu S, Jambulingam P, Govardhini P, Mohapatra SS, Das PK. Living conditions and occurrence of malaria in a rural community. Indian J Malariol. 1991; 28(1): 29-37.
Subramanian SV, Smith GD. Patterns, distribution, and determinants of under- and overnutrition: a population-based study of women in India. Am J Clin Nutr. 2006; 84(3): 633-40.
Sudha P, Bhasin S, Anegundi RT. Prevalence of dental caries among 5-13-year-old children of Mangalore city. J Indian Soc Pedod Prev Dent. 2005; 23(2): 74-9.
Sugunan AP, Murhekar MV, Sehgal SC. Intestinal parasitic infestation among different population groups of Andaman and Nicobar islands. J Commun Dis. 1996; 28(4): 253–9.
Sukhvir S, Bora D, Dhariwal AC, Pawan D, Shiv L. Epidemiological, clinical and entomological observations on lymphatic filariasis in urban Puri, Orissa. J Commun Dis. 2008; 40(2): 161-5.
Sukumar S, Mukherjee MB, Colah RB, Mohanty D. Molecular basis of G6PD deficiency in India. Blood Cells Mol Dis. 2004; 33(2): 141-5.
Sunish IP, Kalimuthu M, Kumar VA, Munirathinam A, Nagaraj J, Tyagi BK, White GB, Arunachalam N. Can community-based integrated vector control hasten the process of LF elimination?. Parasitol Res. 2016; 115(6): 2353-62.
Sunish IP, Munirathinam A, Kalimuthu M, Kumar VA, Tyagi BK. Persistence of Lymphatic Filarial Infection in the Paediatric Population of Rural Community, after Six Rounds of Annual Mass Drug Administrations. J Trop Pediatr. 2014; 60(3): 245-8.
Sunish IP, Rajendran R, Mani TR, Munirathinam A, Dash AP, Tyagi BK. Vector control complements mass drug administration against bancroftian filariasis in Tirukoilur, India. Bull World Health Organ. 2007; 85(2): 138-45.
Sunish IP, Rajendran R, Mani TR, Munirathinam A, Reuben R, Dash AP. Impact of single dose of diethylcarbamazine and other antifilarial drug combinations on bancroftian filarial infection variables: assessment after 2 years. Parasitol Int. 2006; 55(3): 233-6.
Sunish IP, Rajendran R, Satyanarayana K, Munirathinam A, Gajanana A. Immunochromatographic test (ICT) for estimation of true prevalence of bancroftian filariasis in an endemic area in southern India. Trans R Soc Trop Med Hyg. 2001; 95(6): 607-9.
Suprabha BS, Rao A, Shenoy R, Khanal S. Utility of knowledge, attitude, and practice survey, and prevalence of dental caries among 11- to 13-year-old children in an urban community in India. Glob Health Action. 2013; 6: 20750.
Sur D, Ali M, von Seidlein L, Manna B, Deen JL, Acosta CJ, Clemens JD, Bhattacharya SK. Comparisons of predictors for typhoid and paratyphoid fever in Kolkata, India. BMC Public Health. 2007; 7: 289.
Sur D, Ochiai RL, Bhattacharya SK, Ganguly NK, Ali M, Manna B, Dutta S, Donner A, Kanungo S, Park JK, Puri MK, Kim DR, Dutta D, Bhaduri B, Acosta CJ, Clemens JD. A cluster-randomized effectiveness trial of Vi typhoid vaccine in India. N Engl J Med. 2009; 361(4): 335-44.
Sureka RK, Sureka R. Prevalence of epilepsy in rural Rajasthan--a door-to-door survey. J Assoc Physicians India. 2007; 55: 741-2.
Surendran K, Pani SP, Soudarssanane MB, Srinivasa DK, Bordolai PC, Subramanian S. Natural history, trend of prevalence and spectrum of manifestations of Bancroftian filarial disease in Pondicherry (South India). Acta Trop. 1996; 61(1): 9-18.

Table 3: GBD 2016 India data inputs
Surya NC, Gupta SP, Gopalakrishna R, Sundaram D, Kutty J. Mental morbidity in Pondicherry (1962-1963). Trans All India Inst Ment Health. 1964; 4: 50-61.
Suvarna BS, Kamath A. Prevalence of attention deficit disorder among preschool age children. Nepal Med Coll J. 2009; 11(1): 1-4.
Swain S, Singh S, Bhatia BD, Pandey S, Krishna M. Maternal hemoglobin and serum albumin and fetal growth. Indian Pediatr. 1994; 31(7): 777-82.
Swain S, Agrawal A, Bhatia BD. Congenital malformations at birth. Indian Pediatr. 1994; 31(10): 1187-91.
Swami SS, Chandra S, Dudani IU, Sharma R, Mathur MM. Epidemiology of measles in western Rajasthan. J Commun Dis. 1987; 19(4): 370-2.
Swaminathan M, Apte S, Someswara Rao K. Nutrition of the people of Ankola Taluk N. Kanara. Indian J Med Res. 1960; 762-74.
Swaminathan S, Perumal V, Adinarayanan S, Kaliannagounder K, Rengachari R, Purushothaman J. Epidemiological assessment of eight rounds of mass drug administration for lymphatic filariasis in India: implications for monitoring and evaluation. PLoS Negl Trop Dis. 2012; 6(11): e1926.
Talsania NJ, Lala MK. Evaluation of antenatal risk scoring in a preterm birth prevention and perinatal loss. Indian J Matern Child Health. 1994; 5(1): 5-9.
Talukdar A, Khandokar MR, Bandopadhyay SK, Detels R. Risk of HIV infection but not other sexually transmitted diseases is lower among homeless Muslim men in Kolkata. AIDS. 2007; 21(16): 2231–5.
Tamil Nadu Kidney Research Foundation, Kidney Disease Data Center (KDDC), Chennai International Society of Nephrology (ISN). Chronic Kidney Disease and Cardiovascular Risk Survey 2014-2015. Chennai, India; ISN-KDDC.
Tatte VS, Chothe NS, Chitambar SD. Characterisation of rotavirus strains identified in adolescents and adults with acute gastroenteritis highlights circulation of non-typeable strains: 2008-2012. Vaccine. 2014; A68-74.
Tatte VS, Gentsch JR, Chitambar SD. Characterization of group A rotavirus infections in adolescents and adults from Pune, India: 1993-1996 and 2004-2007. J Med Virol. 2010; 82(3): 519–27.
TB Trials Study Group, Uppada DR, Selvam S, Jesuraj N, Lau EL, Doherty TM, Grewal HMS, Vaz M, Lindtjørn B. Incidence of tuberculosis among school-going adolescents in South India. BMC Public Health. 2016; 16(1): 641.
Tennant PWG, Pearce MS, Bythell M, Rankin J. 20-year survival of children born with congenital anomalies: a population-based study. Lancet. 2010; 375(9715): 649-56.
Tewari SC, Hiriyan J, Reuben R. Epidemiology of subperiodic Wuchereria bancrofti infection in the Nicobar Islands, India. Trans R Soc Trop Med Hyg. 1995; 89(2): 163-6.
Thakur JS, Jeet G, Pal A, Singh S, Singh A, Deepti SS, et al. Profile of risk factors for non-communicable diseases in Punjab, Northern India: Results of a state-wide STEPS Survey. PloS one. 2016 11(7):e0157705.
Thakur JS, Negi PC, Ahluwalia SK, Vaidya NK. Epidemiological survey of rheumatic heart disease among school children in the Shimla Hills of northern India: prevalence and risk factors. J Epidemiol Community Health. 1996; 50(1): 62-7.
Thakur JS, Ratho RK, Bhatia SP, Grover R, Issaivanan M, Ahmed B, Parmar V, Swami HM. Measles outbreak in a periurban area of Chandigarh: need for improving vaccine coverage and strengthening surveillance. Indian J Pediatr. 2002; 69(1): 33-7.
Thakur TS, Goyal A, Sharma V, Gupta ML, Singh S. Incidence of australia antigen (HBs Ag) in Himachal Pradesh. J Commun Dis. 1990; 22(3): 173-7.

Table 3: GBD 2016 India data inputs
Thakur TS, Sharma V, Goyal A, Gupta ML. Seroprevalence of HIV antibodies, Australia antigen and VDRL reactivity in Himachal Pradesh. Indian J Med Sci. 1991; 45(12): 332-5.
Thangaleela T, Vijayalakshmi P. Prevalence of Anaemia in Pregnancy. Indian J Nutr Diet.1994 ; 31(2):26–9.
Thapa BR, Singh K, Singh V, Broor S, Singh V, Nain CK. Pattern of hepatitis A and hepatitis B virus markers in cases of acute sporadic hepatitis and in healthy school children from north west India. J Trop Pediatr. 1995; 41(6): 328-9.
Thara R. Twenty-year course of schizophrenia: the Madras Longitudinal Study. Can J Psychiatry. 2004; 49(8): 564-9.
The INTERSALT Co-operative Research Group. Appendix tables. Centre-specific results by age and sex. J Hum Hypertens 1989; 3(5):331-407.
Thilothammal N, Sujaritha R, Banu K, Ratnam S, Ezhlarasi. The nutritional status of south Indian women in child bearing age and its influence on birth weight and maturity of offspring. J Obstet Gynaecol India. 1993; 871-7.
Thomas K, Mukkai Kesavan L, Veeraraghavan B, Jasmine S, Jude J, Shubankar M, Kulkarni P, Steinhoff M. Invasive pneumococcal disease associated with high case fatality in India. J Clin Epidemiol. 2013; 66(1): 36-43.
Thomas K, Thyagarajan SP, Jeyaseelan L, Varghese JC, Krishnamurthy P, Bai L, Hira S, Sudhakar K, Peedicayil A, George S, George R, Rajendran P, Joyee AG, Hari D, Balakrishnan, Sethuraman N, Gharpure H, Srinivasan V. Community prevalence of sexually transmitted diseases and human immunodeficiency virus infection in Tamil Nadu, India: a probability proportional to size cluster survey. Natl Med J India. 2002; 15(3): 135-40.
Thomas SV, Sindhu K, Ajaykumar B, Sulekha Devi PB, Sujamol J. Maternal and obstetric outcome of women with epilepsy. Seizure. 2009; 18(3): 163-6.
Thulasiraj RD, Nirmalan PK, Ramakrishnan R, Krishnadas R, Manimekalai TK, Baburajan NP, Katz J, Tielsch JM, Robin AL. Blindness and vision impairment in a rural south Indian population: the Aravind Comprehensive Eye Survey. Ophthalmology. 2003; 110(8): 1491-8.
Thulasiraj RD, Rahamathulla R, Saraswati A, Selvaraj S, Ellwein LB. The Sivaganga eye survey: I. Blindness and cataract surgery. Ophthalmic Epidemiol. 2002; 9(5): 299-312.
Thun MJ, Hannan LM, Adams-Campbell LL, Boffetta P, Buring JE, Feskanich D, Flanders WD, Jee SH, Katanoda K, Kolonel LN, Lee IM, Marugame T, Palmer JR, Riboli E, Sobue T, Avila-Tang E, Wilkens LR, Samet JM. Lung cancer occurrence in never-smokers: an analysis of 13 cohorts and 22 cancer registry studies. PLoS Med. 2008; 5(9): e185.
Tielsch JM, Rahmathullah L, Thulasiraj RD, Katz J, Coles C, Sheeladevi S, John R, Prakash K. Newborn vitamin A dosing reduces the case fatality but not incidence of common childhood morbidities in South India. J Nutr. 2007; 137(11): 2470-4.
Tiwari RV, Gupta A, Agrawal A, Gandhi A, Gupta M, Das M. Women and Tobacco Use: Discrepancy in the Knowledge, Belief and Behavior towards Tobacco Consumption among Urban and Rural Women in Chhattisgarh, Central India. Asian Pac J Cancer Prev. 2015; 16(15): 6365-73.
Tiwari S, Hopke PK, Pipal AS, Srivastava AK, Bishta DS, Tiwari S, Singh AK, Sonie VK, Attrie SD. Intra-urban variability of particulate matter (PM2.5 and PM10) and its relationship with optical properties of aerosols over Delhi, India. Atmos Res. 2015; 166: 223-32.
Tripathi RM, Khandekar RN, Raghunath R, Mishra UC. Assessment of atmospheric pollution from toxic heavy metals in two cities in India. Atmos Environ. 1989; 23(4): 879–83.
Tripathi RM, Raghunath R, Kumar AV, Sastry VN, Sadasivan S. Atmospheric and children’s blood lead as indicators of vehicular traffic and other emission sources in Mumbai, India. Sci Total Environ. 2001; 267(1–3): 101–8.
Tripathi RM, Raghunath R, Mahapatra S, Sadasivan S. Blood lead and its effect on Cd, Cu, Zn, Fe and hemoglobin levels of children. Sci Total Environ. 2001; 277(1-3): 161-8.
Tripathy V, Satapathy KC, Gupta R. ABO and Rh D polymorphism among Tibetans in India. Hum Biol. 2006; 78(2): 229-33.

Table 3: GBD 2016 India data inputs
Trivedi H, Vanikar A, Patel H, Kanodia K, Kute V, Nigam L, Suthar K, Thakkar U, Sutariya H, Gandhi S. High prevalence of chronic kidney disease in a semi-urban population of Western India. Clin Kidney J. 2016; 9(3): 438-43.
Trivedi SS, Pasrija S. Teenage pregnancies and their obstetric outcomes. Trop Doct. 2007; 37(2): 85-8.
TRL Limited-United Kingdom. The Involvement and Impact of Road Crashes on the Poor: Bangladesh and India Case Studies. Berkshire, England: TRL Limited United Kingdom; 2004.
Tyagi S, Tyagi A. Possible Correlation of Transfusion Transmitted Diseases with Rh type and ABO Blood Group System. J Clin Diagn Res. 2013; 7.0(9): 1930-1.
Tyrovolas S, Koyanagi A, Garin N, Olaya B, Ayuso-Mateos JL, Miret M, Chatterji S, Tobiasz-Adamczyk B, Koskinen S, Leonardi M, Haro JM. Determinants of the components of arterial pressure among older adults--the role of anthropometric and clinical factors: a multi-continent study. Atherosclerosis. 2015; 238(2): 240–9.
U.S. Department of Agriculture (USDA). USDA Global Tobacco Database 1960-2005. Washington DC , United States: USDA.
Uberoi IS, DeSweemer C, Taylor CE. A study of anemia among rural Punjabi children. Indian J Med Res. 1972; 60(5): 793-9.
Uma S, Balakrishnan P, Murugavel KG, Srikrishnan AK, Kumarasamy N, Anand S, Cecelia JA, Celentano D, Mayer KH, Thyagarajan SP, Solomon S. Bacterial vaginosis in women of low socioeconomic status living in slum areas in Chennai, India. Sex Health. 2006; 3(4): 297–8.
United Nations Children's Fund (UNICEF). The State of the World's Children 1996. Oxford, United Kingdom and New York, United States: Oxford University Press for UNICEF.
United Nations Children's Fund (UNICEF). The State of the World's Children 1997. Oxford, United Kingdom and New York, United States: Oxford University Press for UNICEF.
United Nations Children's Fund (UNICEF). The State of the World's Children 2000. New York, United States: UNICEF.
United Nations Children's Fund (UNICEF). The State of the World's Children 2001. New York, United States: UNICEF.
United Nations Children's Fund (UNICEF). The State of the World's Children 2005. New York, United States: UNICEF.
United Nations Children's Fund (UNICEF). The State of the World's Children 2007. New York, United States: UNICEF.
United Nations Children's Fund (UNICEF). The State of the World's Children 2009. New York, United States: UNICEF.
United Nations Children's Fund (UNICEF). The State of the World's Children 2011. New York, United States: UNICEF.
United Nations Children's Fund (UNICEF). The State of the World's Children 2012. New York, United States: UNICEF.
United Nations Office on Drugs and Crime (UNODC) Regional Office for South Asia. Rapid Situation and Response Assessment of Drugs and HIV in Bangladesh, Bhutan, India, Nepal and Sri Lanka - A Regional Report 2008. New Delhi, India: UNODC Regional Office for South Asia.
United Nations Office on Drugs and Crime (UNODC). UNODC Global Study on Homicide 2011. Vienna, Austria: UNODC; 2011.
United Nations Office on Drugs and Crime (UNODC). World Drug Report 2012. Vienna, Austria: UNODC; 2012.
United Nations Population Division, Department of Economic and Social Affairs, United Nations (UN). Abortion Policies: A Global Review. New York, United States: UN; 2002.
United Nations Population Division, Department of Economic and Social Affairs, United Nations (UN). World Abortion Policies 2007. New York, United States: UN; 2007.
United Nations Population Division, Department of Economic and Social Affairs, United Nations (UN). World Abortion Policies 2013. New York, United States: UN; 2013.

Table 3: GBD 2016 India data inputs
United Nations Population Division, Department of Economic and Social Affairs, United Nations. Trends in International Migrant Stock: Migrants by Destination and Origin - 2013 Revision. New York City, United States: United Nations Population Division.
United Nations Statistics Division (UNSD). United Nations Demographic Yearbook. New York City, United States: UNSD.
United States Geological Survey (USGS). USGS Mineral Industry Surveys: World Asbestos Consumption 2003-2007. Reston, United States: USGS; 2009.
United States Geological Survey (USGS). USGS Minerals Yearbook 2013. Reston, United States: USGS.
Unnikrishnan JP, Sylaja S, Nayak SD, Radhakrishnan K. India - Trivandrum Stroke Registry 2005. [Data shared for this analysis]
Upadhyay RP, Rai SK, Krishnan A. Using three delays model to understand the social factors responsible for neonatal deaths in rural Haryana, India. J Trop Pediatr. 2013; 59(2): 100-5.
Upadhyaya S, Shettyb S, Kumarc SS, Dongred A, Deshmukhe P. Institutionalizing district level infant death review: an experience from southern India. Southeast Asia J Public Health. 2012; 1(4): 446-56.
Upadhyayula SM, Mutheneni SR, Kumaraswamy S, Kadiri MR, Pabbisetty SK, Yellepeddi VSM. Filaria monitoring visualization system: a geographical information system-based application to manage lymphatic filariasis in Andhra Pradesh, India. Vector Borne Zoonotic Dis. 2012; 12(5): 418-27.
Uppada DR, Selvam S, Jesuraj N, Bennett S, Verver S, Grewal HM, Vaz M. The tuberculin skin test in school going adolescents in South India: associations of socio-demographic and clinical characteristics with TST positivity and non-response. BMC Infect Dis. 2014; 14(1): 1.
Uppsala Conflict Data Program (UCDP), Department of Peace and Conflict Research, Uppsala University, Centre for the Study of Civil War, International Peace Research Institute (PRIO). UCDP/PRIO Armed Conflict Database Version 4, 2015 - UCDP. Uppsala, Sweden: Uppsala University; 2014.
Uppsala Conflict Data Program (UCDP), Department of Peace and Conflict Research, Uppsala University. UCDP Battle-Related Deaths Database, Version 5, 2016. Uppsala, Sweden: Uppsala University; 2015.
Uppsala Conflict Data Program (UCDP), Department of Peace and Conflict Research, Uppsala University. UCDP Nonstate Conflict Dataset, Version 2.5, 2016. Uppsala, Sweden: Uppsala University; 2013.
Uppsala Conflict Data Program (UCDP), Department of Peace and Conflict Research, Uppsala University. UCDP One-Sided Violence Dataset, Version 1.4, 2015. Uppsala, Sweden: Uppsala University; 2015.
Usha Menon V, Sundaram KR, Unnikrishnan AG, Jayakumar RV, Nair V, Kumar H. High prevalence of undetected thyroid disorders in an iodine sufficient adult south Indian population. J Indian Med Assoc. 2009; 107(2): 72-7.
V M Vashishtha, A Kalra, K Kalra, V K Jain. Prevalence of congenital heart disease in school children. Indian Pediatr. 1993; 30(11): 1337-40.
Vadivoo S, Gupte MD, Adhikary R, Kohli A, Kangusamy B, Joshua V, Mathai AK, Kumar K, Mainkar M, Goswami P, IBBA Study Team. Appropriateness and execution challenges of three formal size estimation methods for high-risk populations in India. AIDS. 2008; 22 Suppl 5: S137-148.
Vaid A, Mammen A, Primrose B, Kang G. Infant mortality in an urban slum. Indian J Pediatr. 2007; 74(5): 449-53.
Vaishnav KG, Patel IC. Independent assessment of Mass Drug Administration in filariasis affected Surat city. J Commun Dis. 2006; 38(2): 149-54.
Vas CJ, Pinto C, Panikker D, Noronha S, Deshpande N, Kulkarni L, Sachdeva S. Prevalence of Dementia in an Urban Indian Population. Int Psychogeriatr. 2001; 13(04): 439-50.

Table 3: GBD 2016 India data inputs
Vashishtha VM, John TJ. Prevalence of Mycobacterium tuberculosis infection in children in western Uttar Pradesh. Indian Pediatr. 2010; 47(1): 97–100.
Vashistha VM, Kalra A, Kalra K, Jain VK. Prevalence of rheumatic heart disease in school children. Indian Pediatr. 1993; 30(1): 53-6.
Vasudev JP, Nandan D, Chandra R, Srivastava BC. Post measles complications in a rural population. J Commun Dis. 1983; 15(4): 249-52.
Vaz NC, Ferreira AM, Kulkarni MS, Vaz FS. Prevalence of diabetes mellitus in a rural population of Goa, India. Natl Med J India. 2011; 24(1): 16-8.
Venkatarao T, Ramakrishnan R, Nair NG, Radhakrishnan S, Sundaramoorthy L, Koya PK, Kumar SK. Effect of vitamin A supplementation to mother and infant on morbidity in infancy. Indian Pediatr. 1996; 33(4): 279-86. as it appears in Imdad A, Herzer K, Mayo-Wilson E, Yakoob MY, Bhutta ZA. Vitamin A supplementation for preventing morbidity and mortality in children from 6 months to 5 years of age. Cochrane Database Syst Rev. 2010; CD008524.
Venkitaraman AR, Seigneurin JM, Baccard M, Lenoir GM, John TJ. Measurement of antibodies to varicella-zoster virus in a tropical population by enzyme-linked immunosorbent assay. J Clin Microbiol. 1984; 20(3): 582-3.
Venkitaraman AR, Seigneurin JM, Lenoir GM, John TJ. Infections due to the human herpesviruses in southern India: a seroepidemiological survey. Int J Epidemiol. 1986; 15(4): 561-6.
Verma A, Shrimali L. Maternal body mass index and pregnancy outcome. J Clin Diagn Res. 2012; 6(9): 1531-3.
Verma M, Chhatwal J, Kaur G. Prevalence of anemia among urban school children of Punjab. Indian Pediatr. 1998; 35(12): 1181-6.
Verma M, Singla D, Crowell SB. G6PD deficiency in neonates: a prospective study. Indian J Pediatr. 1990; 57(3): 385–8.
Verma PK, Tewari KN. Epidemiology of Road Traffic Injuries in Delhi Result of a Survey. Regional Health Forum. 2004; 8(1): 1-10.
Vethanayagam RR, Ananda Babu M, Nagalaxmi KS, Maiya PP, Venkatesh HA, Purohit S, Behl R, Bhan MK, Ward RL, Greenberg HB, Durga Rao C. Possible role of neonatal infection with the asymptomatic reassortant rotavirus (RV) strain I321 in the decrease in hospital admissions for RV diarrhea, Bangalore, India, 1988-1999. J Infect Dis. 2004; 189(12): 2282-9.
Victora CG, Huttly SR, Fuchs SC, Barros FC, Garenne M, Leroy O, Fontaine O, Beau JP, Fauveau V, Chowdhury HR. International differences in clinical patterns of diarrhoeal deaths: a comparison of children from Brazil, Senegal, Bangladesh, and India. J Diarrhoeal Dis Res. 1993; 11(1): 25-9.
Vidwan NK, Regi A, Steinhoff M, Huppert JS, Staat MA, Dodd C, Nongrum R, Anandan S, Verghese V. Low prevalence of Chlamydia trachomatis infection in non-urban pregnant women in Vellore, S. India. PLoS One. 2012; 7(5): e34794.
Vidyalakshmi K, Yashavanth R, Chakrapani M, Shrikala B, Bharathi B, Suchitra U, Dhanashree B, Dominic RMS. Epidemiological shift, seasonal variation and antimicrobial susceptibility patterns among enteric fever pathogens in South India. Trop Doct. 2008; 38(2): 89-91.
Vignesh SS, Jaya V, Sasireka BI, Sarathy K, Vanthana M. Prevalence and referral rates in neonatal hearing screening program using two step hearing screening protocol in Chennai - A prospective study. Int J Pediatr Otorhinolaryngol. 2015; 79(10): 1745–7.
Vijaya L, Asokan R, Panday M, Choudhari NS, Ramesh SV, Velumuri L, Boddupalli SD, Sunil GT, George R. Baseline risk factors for incidence of blindness in a South Indian population: the chennai eye disease incidence study. Invest Ophthalmol Vis Sci. 2014; 55(9): 5545-50.
Vijaya L, George R, Arvind H, Baskaran M, Raju P, Ramesh SV, Paul PG, Kumaramanickavel G, McCarty C. Prevalence and causes of blindness in the rural population of the Chennai Glaucoma Study. Br J Ophthalmol. 2006; 90(4): 407-10.

Table 3: GBD 2016 India data inputs
Vijaya L, George R, Asokan R, Velumuri L, Ramesh SV. Prevalence and causes of low vision and blindness in an urban population: The Chennai Glaucoma Study. Indian J Ophthalmol. 2014; 62(4):477-81.
Vijaya L, George R, Baskaran M, Arvind H, Raju P, Ramesh SV, Kumaramanickavel G, McCarty C. Prevalence of primary open-angle glaucoma in an urban south Indian population and comparison with a rural population. The Chennai Glaucoma Study. Ophthalmology. 2008; 115(4): 648-54.
Vijaya Mn D, Umashankar K, Sudha null, Nagure AG, Kavitha G. Prevalence of the trichomonas vaginalis infection in a tertiary care hospital in rural bangalore, southern India. J Clin Diagn Res. 2013; 7(7): 1401–3.
Vijayakumar G, Arun R, Kutty VR. High prevalence of type 2 diabetes mellitus and other metabolic disorders in rural Central Kerala. J Assoc Physicians India. 2009; 57: 563-7.
Vijayaraghavan K, Rao DH. Diet & nutrition situation in rural India. Indian J Med Res. 1998; 108:243-53.
Vijesh S. Prevalence of cholera in pediatric patients with acute dehydrating diarrhea. Indian J Pediatr. 2010; 77(1): 67-71.
Virta RL, United States Geological Survey (USGS). Worldwide Asbestos Supply and Consumption Trends from 1990 through 2003: USGS Circular 1298. Reston, United States: USGS; 2006.
Virus Research And Diagnostic Laboratory Network, National Institute of Epidemiology, Indian Council of Medical Research. Laboratory-wise data on Dengue 2014-2016. [Data shared for this analysis]
Viswanathan R, Singh AK, Ghosh C, Dasgupta S, Mukherjee S, Basu S. Profile of neonatal septicaemia at a district-level sick newborn care unit. J Health Popul Nutr. 2012; 30(1): 41-8.
Viswanathan V, Kumpatla S. Pattern and causes of amputation in diabetic patients--a multicentric study from India. J Assoc Physicians India. 2011; 59:148–51.
Viswanathan V, Thomas N, Tandon N, Asirvatham A, Rajasekar S, Ramachandran A, Senthilvasan K, Murugan VS, Muthulakshmi. Profile of diabetic foot complications and its associated complications – a multicentric study from India. J Assoc Physicians India. 2005; 53: 933-6.
Vivek R, Chandy GM, Brown DW, Kang G. Seroprevalence of IgG antibodies to hepatitis E in urban and rural southern India. Trans R Soc Trop Med Hyg. 2010; 104(4): 307-9.
Vogel JP, Lee ACC, Souza JP. Maternal morbidity and preterm birth in 22 low- and middle-income countries: a secondary analysis of the WHO Global Survey dataset. BMC Pregnancy Childbirth. 2014; 14: 56.
Vogel JP, Souza JP, Mori R, Morisaki N, Lumbiganon P, Laopaiboon M, Ortiz-Panozo E, Hernandez B, Pérez-Cuevas R, Roy M, Mittal S, Cecatti JG, Tunçalp Ö, Gülmezoglu AM; WHO Multicountry Survey on Maternal and Newborn Health Research Network. Maternal complications and perinatal mortality: findings of the World Health Organization Multicountry Survey on Maternal and Newborn Health. BJOG. 2014; 121(Supp 1): 76-88.
Wadia NH, Bhatia K. Multiple sclerosis is prevalent in the Zoroastrians (Parsis) of India. Ann Neurol. 1990; 28(2): 177-9.
Walia R, Bhansali A, Ravikiran M, Ravikumar P, Bhadada SK, Shanmugasundar G, Dutta P, Sachdeva N. High prevalence of cardiovascular risk factors in Asian Indians: a community survey - Chandigarh Urban Diabetes Study (CUDS). Indian J Med Res. 2014; 139(2): 252–9.
Wall LL, Arrowsmith SD, Briggs ND, Browning A, Lassey A. The Obstetric Vesicovaginal Fistula in the Developing World (Chapter 22). Bristol, United Kingdom: The International Continence Society; 2005. p.1403-54. Available from: https://www.ics.org/publications/ici_3/v2.pdf/chap22.pdf
Wang B-E, Ma W-M, Sulaiman A, Noer S, Sumoharjo S, Sumarsidi D, Tandon BN, Nakao K, Mishiro S, Miyakawa Y, Akahane Y, Suzuki H. Demographic, clinical, and virological characteristics of hepatocellular carcinoma in Asia: survey of 414 patients from four countries. J Med Virol. 2002; 67(3): 394-400.

Table 3: GBD 2016 India data inputs
Wang H-Y, Leena KB, Plymoth A, Hergens M-P, Yin L, Shenoy KT, Ye W. Prevalence of gastro-esophageal reflux disease and its risk factors in a community-based population in southern India. BMC Gastroenterol. 2016; 16: 36.
Wani SA, Ahmad F, Zargar SA, Ahmad Z, Ahmad P, Tak H. Prevalence of intestinal parasites and associated risk factors among schoolchildren in Srinagar City, Kashmir, India. J Parasitol. 2007; 93(6): 1541–3.
Wani SA, Ahmad F, Zargar SA, Dar PA, Dar ZA, Jan TR. Intestinal helminths in a population of children from the Kashmir valley, India. J Helminthol. 2008; 82(4): 313–7.
Waterhouse J, Muir CS, Correa P, Powell J, eds. Cancer Incidence in Five Continents, Vol. III. International Agency for Research on Cancer (IARC) Scientific Publications, No. 15. Lyon, France: IARC; 1976.
Waterhouse J, Muir CS, Shanmugaratnam K,Powell J, eds. Cancer Incidence in Five Continents, Vol. IV. International Agency for Research on Cancer (IARC) Scientific Publications, No. 42. Lyon, France: IARC; 1982.
Weatherall D. Sickle Cell and Thalassemias Prevalence Data, Personal Correspondence with David Weatherall. [Data shared for this analysis]
Werner GT, Frosner GG, Sareen DK. Prevalence of hepatitis A, B and HIV markers in Punjab. J Indian Med Assoc. 1990; 88(10): 293-4.
Whooping Cough Case Fatality Rate Estimates as provided by the Global Burden of Disease 2010 Pertussis Expert Group. [Data shared for this analysis]
Wikipedia. List of Terrorist Incidents in 2016. San Francisco, United States: Wikipedia.
Williams H, Stewart A, Von Mutius E, Cookson W, Anderson HR. Is eczema really on the increase worldwide. J Allergy Clin Immunol. 2008; 121(4): 947-954.
Working Group on Fortification of Salt with Iron. Use of common salt fortified with iron in the control and prevention of anemia--a collaborative study. Report of the Working Group on Fortification of Salt with Iron. Am J Clin Nutr. 1982; 35(6): 1442-51.
World Bank. Survey of Living Conditions 1997-1998, Uttar Pradesh and Bihar. Washington D.C., United States: World Bank.
World Bank. World Development Indicators - Hospital Beds (per 1,000 People). Washington D.C., United States: World Bank.
World Bank. World Development Indicators - Vitamin A Supplementation Coverage Rate. Washington D.C., United States: World Bank.
World Energy Council. Survey of Energy Resources 2010. London, United Kingdom: World Energy Council; 2010.
World Health Organization (WHO), United Nations Children's Fund (UNICEF). WHO and UNICEF Reported Administrative Data for Immunization Coverage Time Series. Available from: http://www.who.int/immunization/monitoring_surveillance/data/en/
World Health Organization (WHO), United Nations Children's Fund (UNICEF). WHO and UNICEF Reported Disease Incidence Time Series 1980-2016. Geneva, Switzerland: WHO .
World Health Organization (WHO), United Nations Children's Fund (UNICEF). WHO and UNICEF Reported Estimates of Immunization Coverage Time Series 1980-2013. Geneva, Switzerland: WHO.
World Health Organization (WHO), United Nations Children's Fund (UNICEF). WHO and UNICEF Survey Data for Immunization Coverage Time Series 1978-2013. Geneva, Switzerland: WHO.
World Health Organization (WHO). Global Database on Child Growth and Malnutrition - Historical. Geneva, Switzerland: WHO.
World Health Organization (WHO). Global Status Report on Road Safety 2009. Geneva, Switzerland: WHO; 2009.

Table 3: GBD 2016 India data inputs
World Health Organization (WHO). India WHO Leishmaniasis Country Profile 1990-2010. Geneva, Switzerland: WHO.
World Health Organization (WHO). Mental Illness in General Health Care: An International Study. Geneva, Switzerland: WHO; 1995.
World Health Organization (WHO). WHO DengueNet 1955-2016. Geneva, Switzerland: WHO.
World Health Organization (WHO). WHO Global Database on Anemia, Nutrition Landscape Information System. Geneva, Switzerland: WHO.
World Health Organization (WHO). WHO Global Database on Iodine Deficiency. Geneva, Switzerland: WHO.
World Health Organization (WHO). WHO Global Database on Vitamin A Deficiency. Geneva, Switzerland: WHO.
World Health Organization (WHO). WHO Global Health Observatory - Cholera: Number of Reported Cases by Country. Geneva, Switzerland: WHO.
World Health Organization (WHO). WHO Global Health Observatory - Population Living in Trachoma Endemic Areas. Geneva, Switzerland: WHO.
World Health Organization (WHO). WHO Global Health Observatory Interactive Graph - Number of Cases of Cutaneous Leishmaniasis Reported 2003. Geneva, Switzerland: WHO.
World Health Organization (WHO). WHO Global Health Observatory Interactive Graph - Number of Cases of Cutaneous Leishmaniasis Reported 2004. Geneva, Switzerland: WHO.
World Health Organization (WHO). WHO Global Health Observatory Interactive Graph - Number of Cases of Cutaneous Leishmaniasis Reported 2005. Geneva, Switzerland: WHO.
World Health Organization (WHO). WHO Global Health Observatory Interactive Graph - Number of Cases of Cutaneous Leishmaniasis Reported 2006. Geneva, Switzerland: WHO.
World Health Organization (WHO). WHO Global Health Observatory Interactive Graph - Number of Cases of Cutaneous Leishmaniasis Reported 2007. Geneva, Switzerland: WHO.
World Health Organization (WHO). WHO Global Health Observatory Interactive Graph - Number of Cases of Cutaneous Leishmaniasis Reported 2008. Geneva, Switzerland: WHO.
World Health Organization (WHO). WHO Global Health Observatory Interactive Graph - Number of Cases of Cutaneous Leishmaniasis Reported 2009. Geneva, Switzerland: WHO.
World Health Organization (WHO). WHO Global Health Observatory Interactive Graph - Number of Cases of Cutaneous Leishmaniasis Reported 2011. Geneva, Switzerland: WHO.
World Health Organization (WHO). WHO Global Health Observatory Interactive Graph - Number of Cases of Cutaneous Leishmaniasis Reported 2012. Geneva, Switzerland: WHO.
World Health Organization (WHO). WHO Global Health Observatory Interactive Graph - Number of Cases of Cutaneous Leishmaniasis Reported 2013. Geneva, Switzerland: WHO.
World Health Organization (WHO). WHO Global Health Observatory Interactive Graph - Number of Cases of Visceral Leishmaniasis Reported 1998. Geneva, Switzerland: WHO.
World Health Organization (WHO). WHO Global Health Observatory Interactive Graph - Number of Cases of Visceral Leishmaniasis Reported 1999. Geneva, Switzerland: WHO.
World Health Organization (WHO). WHO Global Health Observatory Interactive Graph - Number of Cases of Visceral Leishmaniasis Reported 2000. Geneva, Switzerland: WHO.
World Health Organization (WHO). WHO Global Health Observatory Interactive Graph - Number of Cases of Visceral Leishmaniasis Reported 2001. Geneva, Switzerland: WHO.

Table 3: GBD 2016 India data inputs
World Health Organization (WHO). WHO Global Health Observatory Interactive Graph - Number of Cases of Visceral Leishmaniasis Reported 2003. Geneva, Switzerland: WHO.
World Health Organization (WHO). WHO Global Health Observatory Interactive Graph - Number of Cases of Visceral Leishmaniasis Reported 2004. Geneva, Switzerland: WHO.
World Health Organization (WHO). WHO Global Health Observatory Interactive Graph - Number of Cases of Visceral Leishmaniasis Reported 2005. Geneva, Switzerland: WHO.
World Health Organization (WHO). WHO Global Health Observatory Interactive Graph - Number of Cases of Visceral Leishmaniasis Reported 2006. Geneva, Switzerland: WHO.
World Health Organization (WHO). WHO Global Health Observatory Interactive Graph - Number of Cases of Visceral Leishmaniasis Reported 2007. Geneva, Switzerland: WHO.
World Health Organization (WHO). WHO Global Health Observatory Interactive Graph - Number of Cases of Visceral Leishmaniasis Reported 2008. Geneva, Switzerland: WHO.
World Health Organization (WHO). WHO Global Health Observatory Interactive Graph - Number of Cases of Visceral Leishmaniasis Reported 2009. Geneva, Switzerland: WHO.
World Health Organization (WHO). WHO Global Health Observatory Interactive Graph - Number of Cases of Visceral Leishmaniasis Reported 2010. Geneva, Switzerland: WHO.
World Health Organization (WHO). WHO Global Health Observatory Interactive Graph - Number of Cases of Visceral Leishmaniasis Reported 2011. Geneva, Switzerland: WHO.
World Health Organization (WHO). WHO Global Health Observatory Interactive Graph - Number of Cases of Visceral Leishmaniasis Reported 2012. Geneva, Switzerland: WHO.
World Health Organization (WHO). WHO Global Health Observatory Interactive Graph - Number of Cases of Visceral Leishmaniasis Reported 2013. Geneva, Switzerland: WHO.
World Health Organization (WHO). WHO Global Health Observatory Interactive Graph - Number of Cases of Visceral Leishmaniasis Reported 2014. Geneva, Switzerland: WHO.
World Health Organization (WHO). WHO Global Health Observatory Interactive Graph - Number of Cases of Visceral Leishmaniasis Reported 2015. Geneva, Switzerland: WHO.
World Health Organization (WHO). WHO Global Infobase - Tobacco Use Prevalence. Geneva, Switzerland: WHO.
World Health Organization (WHO). WHO Global Project on Anti-Tuberculosis Drug Resistance Surveillance Data 1988-2015. Geneva, Switzerland: WHO.
World Health Organization (WHO). WHO Global Survey on Maternal and Perinatal Health 2004-2008. Geneva, Switzerland: WHO.
World Health Organization (WHO). WHO Household Energy Database. Geneva, Switzerland: WHO; 2010.
World Health Organization (WHO). WHO PCT Databank - Lymphatic Filariasis. Geneva, Switzerland: WHO.
World Health Organization (WHO). WHO PCT Databank - Soil-transmitted Helminthiases. Geneva, Switzerland: WHO.
World Health Organization (WHO). WHO Tuberculosis Case Notifications 2006-2015. Geneva, Switzerland: WHO.
World Health Organization (WHO). WHO Urban Ambient Air Pollution Database Draft 2016. Geneva, Switzerland: WHO.
World Health Organization (WHO). WHO World Mental Health Surveys: Global Perspectives on the Epidemiology of Mental Disorders. Cambridge, United Kingdom: Cambridge University Press; 2008.

Table 3: GBD 2016 India data inputs
World Health Organization (WHO). World Malaria Report 2013. Geneva, Switzerland: WHO.
World Health Organization (WHO). World Malaria Report 2016. Geneva, Switzerland: WHO; 2016.
World Health Organization Regional Office for South-East Asia (SEARO). Iodine-deficiency Disorders in South-East Asia. New Delhi, India: SEARO; 1985.
World Health Organization Regional Office for South-East Asia (SEARO). Reported Cases of DF/DHF in Selected Countries in SEA Region. New Delhi, India: SEARO.
World Health Organization Regional Office for South-East Asia (SEARO). Situation Update of Dengue in the SEA Region, 2010. New Delhi, India: SEARO.
World Health Organization Regional Office for South-East Asia (SEARO). WHO South-East Asia Region: Reported Cases and Deaths of Dengue from 2003 to 2012. New Delhi, India: SEARO; 2013.
World Health Organization. Global leprosy situation, 2005. Wkly Epidemiol Rec. 2005: 80(34): 289-95.
World Health Organization. Global leprosy situation, 2007. Wkly Epidemiol Rec. 2007: 82(25): 225-32.
World Health Organization. Global leprosy situation, 2009. Wkly Epidemiol Rec. 2009: 84(33): 333-40.
World Health Organization. Global leprosy situation, 2010. Wkly Epidemiol Rec. 2010: 85(35): 337-48.
World Health Organization. Global leprosy situation, 2012. Wkly Epidemiol Rec. 2012: 87(34): 317-28.
World Health Organization. Global leprosy situation, beginning of 2008. Wkly Epidemiol Rec. 2008: 83(33): 293-300.
World Health Organization. Global leprosy situation, September 1999. Wkly Epidemiol Rec. 1999: 74(38): 313-6.
World Health Organization. Global leprosy: update on the 2012 situation. Wkly Epidemiol Rec. 2013: 88(35): 365-79.
World Health Organization. Leprosy - Global situation. Wkly Epidemiol Rec. 2000: 75(28): 226-31.
World Health Organization. Leprosy update 2011. Wkly Epidemiol Rec. 2011: 86(36): 389-99.
World Health Organization. Leprosy. Wkly Epidemiol Rec. 2001: 76(23): 173-9.
World Health Organization. Leprosy: Global situation. Wkly Epidemiol Rec. 2002: 77(1): 1-8.
World Health Organization. Progress towards eliminating leprosy as a public health problem. Part I. Wkly Epidemiol Rec. 1994: 69(20): 145-51.
World Health Organization. Progress towards leprosy elimination. Wkly Epidemiol Rec. 1997: 72(23): 165-72.
World Health Organization. Progress towards the elimination of leprosy as a public health problem. Part I. Wkly Epidemiol Rec. 1995: 70(25): 177-82.
World Health Organization. Progress towards the elimination of leprosy as a public health problem. Wkly Epidemiol Rec. 1993: 68(25): 181-6.
World Health Organization. Progress towards the elimination of leprosy as a public health problem. Wkly Epidemiol Rec. 1996: 71(20): 149-56.
World Health Organization. Study on Global Ageing and Adult Health (SAGE) Pilot Study 2005 Data from the Data Archive of Social Research on Aging. Los Altos, United States: Sociometrics Corporation. Available from: http://home.socio.com/age2728.php
Worldwide variations in the prevalence of asthma symptoms: the International Study of Asthma and Allergies in Childhood (ISAAC). Eur Respir J. 1998; 12(2): 315-35.
Xavier D, Pais P, Devereaux PJ, Xie C, Prabhakaran D, Reddy KS, et al. Treatment and outcomes of acute coronary syndromes in India (CREATE): a prospective analysis of registry data. The Lancet. 2008;371(9622):1435–1442.

Table 3: GBD 2016 India data inputs
Yachha SK, Singh V, Kanwar SS, Mehta S. Epidemiology, subgroups and serotypes of rotavirus diarrhea in north Indian communities. Indian Pediatr. 1994; 31(1): 27-33.
Yadav R, Rao VG, Bhat J, Gopi PG, Selvakumar N, Wares DF. Prevalence of pulmonary tuberculosis amongst the Baigas--a primitive tribe of Madhya Pradesh, Central India. Indian J Tuberc. 2010; 57(2): 114-6.
Yadav RS, Sampath TR, Sharma VP, Adak T, Ghosh SK. Evaluation of lambdacyhalothrin-impregnated bednets in a malaria endemic area of India. Part 3. Effects on malaria incidence and clinical measures. J Am Mosq Control Assoc. 1998; 14(4): 444-50.
Yadav RS, Sharma VP, Ghosh SK, Kumar A. Quartan malaria--an investigation on the incidence of Plasmodium malariae in Bisra PHC, District Sundargarh, Orissa. Indian J Malariol. 1990; 27(2): 85-94.
Yusufji, Mathan VI, Baker SJ. Iron, folate, and vitamin B 12 nutrition in pregnancy: a study of 1000 women from southern India. Bull World Health Organ. 1973; 48(1): 15-22.
Yuvaraj J, Pani SP, Vanamail P, Ramaiah KD, Das PK. Impact of seven rounds of mass administration of diethylcarbamazine and ivermectin on prevalence of chronic lymphatic filariasis in south India. Trop Med Int Health. 2008; 13(5): 737-42.
Zachariah ES, Naidu M, Seshadri L. Oral misoprostol in the third stage of labor. Int J Gynaecol Obstet. 2006; 92(1): 23-6.
Zaman FA, Borang A. Prevalence of diabetes mellitus amongst rural hilly population of North Eastern India and its relationship with associated risk factors and related co-morbidities. J Nat Sci Biol Med. 2014; 5(2): 383–8.
Zaman FA, Pal R, Zaman GS, Swati IA, Kayyum A. Glucose indices, frank and undetected diabetes in relation to hypertension and anthropometry in a South Indian rural population. Indian J Public Health. 2011; 55(1): 34–7.
Zargar AH, Khan AK, Masoodi SR, Laway BA, Wani AI, Bashir MI, Dar FA. Prevalence of type 2 diabetes mellitus and impaired glucose tolerance in the Kashmir Valley of the Indian subcontinent. Diabetes Res Clin Pract. 2000; 47(2): 135-46.
Zargar AH, Shah JA, Masoodi SR, Laway BA, Shah NA, Mir MM. Prevalence of goitre in school children in Baramulla (Kashmir valley). Indian J Pediatr. 1997; 64(2): 225-30.
Zargar AH, Shah JA, Mir MM, Laway BA, Masoodi SR, Shah NA. Prevalence of goiter in schoolchildren in Kashmir Valley, India. Am J Clin Nutr. 1995; 62(5): 1020-1.
Zargar AH, Wani AI, Masoodi SR, Laway BA, Salahuddin M. Epidemiologic and etiologic aspects of primary infertility in the Kashmir region of India. Fertil Steril. 1997; 68(4): 637-43.

Table 4: Change in Epidemiological transition ratio from 1990 to 2016				
	Epidemiological transition ratio 1990	Epidemiological transition ratio 2016	Change from 1990 to 2016	Percent change from 1990 to 2016
Kerala	0.49	0.16	-0.33	-67.6
Goa	0.84	0.21	-0.62	-74.5
Tamil Nadu	1.02	0.26	-0.76	-75.0
Punjab	1.05	0.29	-0.76	-72.7
Himachal Pradesh	1.14	0.30	-0.84	-73.7
UTs other than Delhi	0.85	0.32	-0.53	-62.8
Maharashtra	1.10	0.33	-0.77	-70.3
West Bengal	1.34	0.33	-1.01	-75.4
Karnataka	1.16	0.34	-0.82	-71.2
Jammu and Kashmir	1.14	0.34	-0.80	-70.4
Andhra Pradesh	1.50	0.37	-1.13	-75.3
Telangana	1.33	0.38	-0.95	-71.4
Delhi	1.16	0.38	-0.78	-66.7
Haryana	1.34	0.40	-0.94	-70.2
Manipur	1.37	0.42	-0.95	-69.4
Sikkim	1.44	0.45	-0.99	-68.9
Tripura	1.38	0.45	-0.93	-67.4
Gujarat	1.47	0.46	-1.01	-68.5
Uttarakhand	1.40	0.46	-0.94	-66.9
Nagaland	1.52	0.47	-1.05	-68.7
Mizoram	1.18	0.53	-0.65	-55.2
Arunachal Pradesh	1.96	0.55	-1.41	-72.0
Odisha	2.00	0.58	-1.42	-70.9
Madhya Pradesh	2.05	0.60	-1.45	-70.8
Chhattisgarh	1.97	0.60	-1.37	-69.3
Assam	1.66	0.62	-1.04	-62.4
Meghalaya	1.98	0.64	-1.34	-67.6
Rajasthan	2.05	0.66	-1.39	-67.7
Uttar Pradesh	2.08	0.68	-1.40	-67.3
Jharkhand	2.04	0.69	-1.35	-66.2
Bihar	2.06	0.74	-1.32	-63.9

The sequence of states is from the lowest to highest ratio in 2016

Table 5: Change in all-age and age-standardised death rates in the states of India from 1990 to 2016

	1990						2016						Percent Change					
	All Age			Age Standardised			All Age			Age Standardised			All age			Age Standardised		
	Men	Women	Both sexes	Men	Women	Both sexes	Men	Women	Both sexes	Men	Women	Both sexes	Men	Women	Both sexes	Men	Women	Both sexes
**India**	**1043 (1017 to 1067)**	**1005 (977 to 1034)**	**1025 (1003 to 1045)**	**1733 (1688 to 1776)**	**1572 (1525 to 1621)**	**1653 (1617 to 1686)**	**794 (770 to 819)**	**691 (668 to 716)**	**744 (726 to 763)**	**1265 (1230 to 1303)**	**1017 (985 to 1053)**	**1139 (1112 to 1166)**	**-23.9 (-26.8 to -20.6)**	**-31.3 (-34.3 to -28.2)**	**-27.4 (-29.7 to -25.0)**	**-27.0 (-29.7 to -24.0)**	**-35.3 (-38.1 to -32.5)**	**-31.1 (-33.2 to -28.9)**
**Low ETL Group**	**1139 (1094 to 1183)**	**1164 (1110 to 1217)**	**1151 (1112 to 1186)**	**1834 (1757 to 1910)**	**1768 (1682 to 1852)**	**1802 (1742 to 1862)**	**810 (771 to 853)**	**733 (695 to 776)**	**773 (743 to 805)**	**1366 (1304 to 1437)**	**1179 (1117 to 1247)**	**1273 (1226 to 1323)**	**-28.8 (-33.1 to -24.3)**	**-36.9 (-41.3 to -32.4)**	**-32.8 (-36.2 to -29.4)**	**-25.5 (-29.9 to -20.8)**	**-33.3 (-38.2 to -28.3)**	**-29.3 (-32.7 to -25.9)**
Bihar	986 (879 to 1095)	1051 (920 to 1189)	1017 (914 to 1128)	1623 (1461 to 1793)	1623 (1433 to 1819)	1623 (1494 to 1761)	687 (602 to 779)	691 (604 to 792)	689 (624 to 759)	1183 (1037 to 1340)	1202 (1051 to 1377)	1191 (1087 to 1313)	-30.4 (-40.8 to -18.3)	-34.2 (-45.0 to -19.8)	-32.3 (-41.2 to -21.8)	-27.1 (-37.6 to -15.3)	-25.9 (-37.6 to -11.9)	-26.6 (-35.0 to -16.8)
Jharkhand	1089 (957 to 1227)	1097 (960 to 1233)	1093 (983 to 1205)	1774 (1573 to 2003)	1756 (1539 to 1978)	1765 (1608 to 1926)	708 (620 to 803)	675 (580 to 772)	692 (626 to 758)	1240 (1090 to 1399)	1212 (1043 to 1380)	1226 (1110 to 1339)	-35.0 (-45.4 to -21.6)	-38.5 (-48.4 to -27.1)	-36.7 (-44.2 to -28.0)	-30.1 (-41.0 to -16.5)	-31.0 (-42.0 to -17.9)	-30.5 (-38.4 to -21.9)
Uttar Pradesh	1206 (1109 to 1308)	1291 (1170 to 1406)	1246 (1162 to 1324)	1884 (1722 to 2060)	1907 (1696 to 2108)	1894 (1752 to 2021)	854 (768 to 955)	780 (692 to 871)	819 (755 to 885)	1443 (1300 to 1607)	1268 (1127 to 1418)	1357 (1255 to 1463)	-29.2 (-38.3 to -18.9)	-39.6 (-47.3 to -30.1)	-34.3 (-40.5 to -27.2)	-23.4 (-32.9 to -12.6)	-33.5 (-42.6 to -22.6)	-28.4 (-35.1 to -20.8)
Rajasthan	1027 (943 to 1119)	979 (898 to 1065)	1004 (944 to 1067)	1786 (1616 to 1971)	1489 (1340 to 1649)	1632 (1523 to 1749)	781 (688 to 882)	671 (592 to 755)	728 (667 to 793)	1377 (1218 to 1546)	1006 (886 to 1133)	1179 (1077 to 1281)	-23.9 (-34.1 to -11.0)	-31.4 (-40.5 to -21.2)	-27.4 (-34.3 to -19.5)	-22.9 (-33.7 to -9.5)	-32.5 (-42.4 to -22.1)	-27.8 (-35.1 to -19.6)
Meghalaya	861 (768 to 955)	701 (623 to 784)	783 (703 to 864)	1612 (1431 to 1808)	1425 (1254 to 1606)	1523 (1358 to 1694)	571 (498 to 653)	432 (370 to 495)	503 (444 to 570)	1263 (1114 to 1429)	917 (792 to 1047)	1082 (960 to 1214)	-33.6 (-43.9 to -21.0)	-38.3 (-49.3 to -26.1)	-35.8 (-44.8 to -24.5)	-21.6 (-32.7 to -8.0)	-35.7 (-46.8 to -22.8)	-29.0 (-38.4 to -16.8)
Assam	1120 (1018 to 1232)	1057 (948 to 1172)	1090 (1015 to 1172)	2010 (1810 to 2224)	1982 (1753 to 2213)	1998 (1851 to 2161)	882 (768 to 1007)	727 (629 to 831)	807 (728 to 893)	1505 (1319 to 1701)	1254 (1093 to 1426)	1382 (1256 to 1523)	-21.3 (-32.8 to -7.6)	-31.2 (-41.9 to -18.1)	-26.0 (-34.7 to -16.2)	-25.1 (-35.8 to -12.9)	-36.7 (-46.9 to -24.6)	-30.8 (-39.0 to -21.8)
Chhattisgarh	1130 (995 to 1269)	1014 (906 to 1136)	1073 (973 to 1176)	1862 (1664 to 2078)	1645 (1462 to 1859)	1750 (1608 to 1898)	848 (745 to 971)	748 (654 to 858)	798 (725 to 880)	1447 (1284 to 1639)	1155 (1019 to 1312)	1292 (1182 to 1413)	-25.0 (-36.5 to -10.8)	-26.2 (-38.0 to -12.2)	-25.6 (-34.3 to -14.7)	-22.3 (-33.4 to -9.0)	-29.8 (-40.8 to -16.9)	-26.2 (-34.0 to -16.8)
Madhya Pradesh	1169 (1077 to 1272)	1212 (1114 to 1313)	1190 (1121 to 1259)	1825 (1666 to 2007)	1716 (1545 to 1888)	1769 (1650 to 1899)	833 (741 to 931)	715 (630 to 808)	776 (715 to 846)	1383 (1231 to 1540)	1080 (953 to 1220)	1229 (1133 to 1334)	-28.7 (-38.1 to -17.8)	-41.1 (-49.1 to -31.9)	-34.7 (-41.0 to -27.7)	-24.2 (-34.9 to -12.4)	-37.1 (-46.1 to -26.6)	-30.5 (-37.4 to -22.7)
Odisha	1339 (1213 to 1466)	1285 (1155 to 1422)	1312 (1210 to 1415)	2041 (1850 to 2238)	1943 (1740 to 2147)	1993 (1834 to 2142)	922 (815 to 1037)	812 (708 to 942)	868 (790 to 952)	1328 (1186 to 1483)	1179 (1039 to 1356)	1255 (1148 to 1367)	-31.2 (-40.5 to -20.8)	-36.8 (-46.1 to -25.0)	-33.8 (-40.7 to -26.0)	-34.9 (-43.7 to -25.6)	-39.3 (-48.4 to -29.2)	-37.0 (-43.5 to -29.5)
**Lower-middle ETL Group**	**940 (882 to 1001)**	**891 (830 to 951)**	**916 (870 to 959)**	**1663 (1545 to 1785)**	**1436 (1329 to 1547)**	**1546 (1458 to 1625)**	**744 (681 to 808)**	**637 (576 to 700)**	**693 (648 to 736)**	**1254 (1155 to 1355)**	**945 (858 to 1034)**	**1092 (1026 to 1155)**	**-20.8 (-28.5 to -11.4)**	**-28.3 (-36.3 to -20.1)**	**-24.3 (-29.7 to -18.2)**	**-24.5 (-31.9 to -15.5)**	**-34.1 (-41.5 to -26.1)**	**-29.3 (-34.6 to -23.3)**
Arunachal Pradesh	844 (759 to 944)	817 (739 to 899)	832 (755 to 912)	1596 (1402 to 1802)	1497 (1311 to 1684)	1552 (1380 to 1729)	551 (474 to 630)	390 (332 to 449)	474 (409 to 540)	1180 (1015 to 1357)	900 (766 to 1045)	1048 (907 to 1194)	-34.7 (-45.6 to -22.3)	-52.3 (-59.9 to -43.2)	-43.0 (-51.0 to -34.1)	-26.1 (-38.2 to -11.5)	-39.9 (-50.5 to -27.4)	-32.5 (-42.6 to -20.7)
Mizoram	651 (585 to 723)	595 (533 to 660)	624 (564 to 686)	1416 (1259 to 1579)	1223 (1084 to 1369)	1321 (1183 to 1464)	652 (559 to 751)	468 (398 to 548)	562 (489 to 646)	1172 (1012 to 1344)	828 (707 to 965)	997 (871 to 1140)	0.1 (-15.3 to 18.5)	-21.3 (-34.0 to -6.0)	-10.0 (-22.8 to 5.2)	-17.2 (-30.0 to -2.4)	-32.3 (-43.0 to -18.7)	-24.6 (-35.1 to -11.6)
Nagaland	780 (680 to 888)	702 (608 to 801)	743 (654 to 839)	1428 (1255 to 1609)	1279 (1117 to 1456)	1361 (1207 to 1523)	541 (461 to 626)	345 (293 to 405)	448 (387 to 512)	1119 (966 to 1282)	789 (669 to 924)	967 (844 to 1099)	-30.7 (-43.0 to -16.5)	-50.9 (-59.6 to -38.8)	-39.8 (-49.5 to -28.3)	-21.7 (-34.5 to -6.4)	-38.3 (-48.4 to -24.4)	-28.9 (-39.3 to -16.4)
Uttarakhand	1109 (1003 to 1221)	959 (854 to 1068)	1035 (956 to 1123)	1962 (1757 to 2161)	1743 (1542 to 1953)	1853 (1705 to 2007)	870 (767 to 986)	671 (574 to 780)	773 (696 to 846)	1354 (1203 to 1522)	957 (823 to 1108)	1147 (1036 to 1250)	-21.6 (-32.9 to -7.9)	-30.0 (-41.4 to -15.9)	-25.3 (-33.9 to -15.5)	-31.0 (-40.7 to -19.2)	-45.1 (-53.9 to -34.2)	-38.1 (-45.2 to -30.3)
Gujarat	925 (849 to 1005)	895 (818 to 974)	910 (853 to 968)	1634 (1482 to 1796)	1398 (1263 to 1544)	1510 (1400 to 1618)	735 (653 to 823)	656 (581 to 738)	698 (642 to 756)	1245 (1114 to 1383)	951 (845 to 1064)	1089 (1004 to 1173)	-20.5 (-30.6 to -8.5)	-26.6 (-36.8 to -15.4)	-23.4 (-30.7 to -15.0)	-23.8 (-34.1 to -12.0)	-32.0 (-41.5 to -21.3)	-27.9 (-35.0 to -19.8)
Tripura	1003 (899 to 1118)	939 (842 to 1055)	972 (879 to 1081)	1633 (1455 to 1838)	1478 (1313 to 1672)	1558 (1404 to 1746)	814 (705 to 938)	672 (572 to 783)	745 (648 to 854)	1282 (1121 to 1467)	963 (824 to 1117)	1119 (978 to 1277)	-18.9 (-31.8 to -3.0)	-28.5 (-40.3 to -14.2)	-23.4 (-34.7 to -9.5)	-21.5 (-33.9 to -6.5)	-34.8 (-45.6 to -21.9)	-28.2 (-38.6 to -16.1)
Sikkim	805 (712 to 897)	738 (653 to 834)	773 (688 to 862)	1528 (1352 to 1725)	1415 (1247 to 1605)	1478 (1313 to 1658)	561 (484 to 656)	346 (300 to 403)	461 (406 to 531)	1043 (904 to 1207)	728 (635 to 851)	905 (795 to 1033)	-30.2 (-41.7 to -16.7)	-53.1 (-60.7 to -43.7)	-40.3 (-49.0 to -29.8)	-31.8 (-42.8 to -18.2)	-48.6 (-56.9 to -37.8)	-38.8 (-47.7 to -28.3)
Manipur	821 (738 to 911)	757 (669 to 851)	789 (710 to 873)	1454 (1303 to 1631)	1353 (1185 to 1536)	1405 (1255 to 1564)	729 (632 to 841)	541 (465 to 626)	636 (556 to 731)	1215 (1067 to 1390)	915 (791 to 1051)	1065 (936 to 1213)	-11.2 (-24.4 to 4.5)	-28.6 (-40.4 to -13.1)	-19.4 (-31.0 to -5.5)	-16.4 (-28.1 to -2.7)	-32.4 (-43.4 to -18.2)	-24.2 (-34.8 to -11.4)
**Higher-middle ETL Group**	**981 (938 to 1022)**	**917 (880 to 955)**	**950 (921 to 979)**	**1702 (1625 to 1778)**	**1513 (1444 to 1579)**	**1608 (1555 to 1658)**	**773 (731 to 815)**	**655 (616 to 693)**	**716 (684 to 745)**	**1203 (1143 to 1263)**	**934 (882 to 985)**	**1064 (1019 to 1105)**	**-21.1 (-26.2 to -15.4)**	**-28.5 (-33.1 to -23.5)**	**-24.6 (-28.6 to -20.4)**	**-29.3 (-33.9 to -24.2)**	**-38.2 (-42.4 to -33.9)**	**-33.8 (-37.2 to -30.2)**
Haryana	990 (893 to 1082)	912 (826 to 1005)	954 (884 to 1022)	1670 (1486 to 1846)	1410 (1260 to 1579)	1543 (1419 to 1659)	893 (791 to 1007)	658 (580 to 743)	784 (712 to 860)	1470 (1313 to 1643)	966 (856 to 1087)	1213 (1108 to 1325)	-9.8 (-22.1 to 4.6)	-27.9 (-38.9 to -15.8)	-17.8 (-26.4 to -8.4)	-12.0 (-23.9 to 2.2)	-31.5 (-42.1 to -19.2)	-21.4 (-29.6 to -12.3)
Delhi	685 (612 to 769)	653 (575 to 733)	671 (614 to 731)	1391 (1241 to 1564)	1239 (1077 to 1406)	1320 (1205 to 1448)	507 (432 to 588)	414 (350 to 482)	465 (408 to 517)	991 (855 to 1133)	763 (646 to 880)	878 (778 to 968)	-26.0 (-38.4 to -11.9)	-36.5 (-48.4 to -24.6)	-30.7 (-40.1 to -20.8)	-28.8 (-40.5 to -16.1)	-38.4 (-49.9 to -26.1)	-33.5 (-42.4 to -24.8)
Telangana	923 (819 to 1031)	887 (781 to 1002)	905 (820 to 1000)	1563 (1374 to 1762)	1509 (1310 to 1715)	1538 (1376 to 1705)	678 (579 to 790)	601 (507 to 713)	640 (555 to 735)	1074 (927 to 1235)	870 (741 to 1020)	967 (846 to 1098)	-26.5 (-38.4 to -11.9)	-32.3 (-44.4 to -17.7)	-29.3 (-39.5 to -17.0)	-31.3 (-42.1 to -17.8)	-42.4 (-52.5 to -29.6)	-37.2 (-45.8 to -25.9)
Andhra Pradesh	1032 (929 to 1139)	1006 (905 to 1118)	1019 (930 to 1116)	1702 (1519 to 1896)	1621 (1454 to 1821)	1662 (1516 to 1835)	795 (702 to 910)	657 (567 to 766)	727 (644 to 825)	1236 (1101 to 1400)	941 (819 to 1088)	1080 (965 to 1213)	-23.0 (-34.1 to -9.7)	-34.7 (-44.8 to -21.2)	-28.7 (-38.1 to -17.7)	-27.4 (-37.0 to -15.4)	-42.0 (-50.6 to -30.2)	-35.0 (-43.1 to -24.8)
Jammu and Kashmir	931 (841 to 1031)	897 (805 to 999)	915 (849 to 991)	1695 (1521 to 1886)	1616 (1430 to 1816)	1660 (1533 to 1800)	699 (611 to 801)	587 (503 to 683)	647 (584 to 713)	1178 (1039 to 1340)	951 (819 to 1100)	1068 (970 to 1171)	-25.0 (-36.8 to -13.0)	-34.5 (-45.3 to -22.0)	-29.3 (-37.4 to -20.0)	-30.5 (-41.2 to -18.7)	-41.2 (-50.8 to -30.0)	-35.7 (-43.3 to -27.2)
Karnataka	1020 (926 to 1111)	889 (811 to 980)	955 (891 to 1020)	1713 (1545 to 1882)	1427 (1280 to 1596)	1568 (1458 to 1691)	827 (722 to 940)	736 (641 to 838)	782 (709 to 855)	1268 (1119 to 1426)	1006 (882 to 1140)	1131 (1032 to 1229)	-18.8 (-31.2 to -5.1)	-17.2 (-29.3 to -2.8)	-18.1 (-26.9 to -8.0)	-25.9 (-37.4 to -14.1)	-29.5 (-39.8 to -16.9)	-27.9 (-35.6 to -19.0)
West Bengal	995 (903 to 1085)	989 (897 to 1078)	992 (925 to 1058)	1758 (1588 to 1930)	1671 (1512 to 1833)	1717 (1593 to 1834)	779 (690 to 889)	671 (586 to 762)	727 (664 to 794)	1197 (1070 to 1350)	995 (875 to 1121)	1096 (1007 to 1188)	-21.7 (-32.4 to -8.5)	-32.1 (-41.9 to -20.4)	-26.8 (-34.5 to -18.2)	-31.9 (-40.8 to -20.7)	-40.5 (-48.9 to -30.1)	-36.2 (-42.6 to -28.4)
Maharashtra	989 (903 to 1077)	876 (795 to 959)	934 (874 to 995)	1743 (1588 to 1902)	1435 (1297 to 1581)	1586 (1474 to 1686)	792 (697 to 894)	666 (585 to 748)	731 (668 to 795)	1174 (1045 to 1315)	881 (780 to 984)	1023 (939 to 1107)	-19.9 (-31.1 to -6.6)	-23.9 (-34.7 to -12.0)	-21.7 (-29.5 to -13.1)	-32.6 (-41.3 to -21.9)	-38.6 (-47.2 to -28.9)	-35.5 (-41.8 to -28.5)
Union Territories other than Delhi	601 (532 to 671)	595 (524 to 659)	598 (534 to 665)	1237 (1095 to 1377)	1096 (962 to 1224)	1166 (1041 to 1289)	535 (453 to 635)	421 (349 to 510)	482 (411 to 570)	1010 (868 to 1180)	710 (596 to 853)	856 (736 to 999)	-11.0 (-27.3 to 5.6)	-29.3 (-42.0 to -12.3)	-19.4 (-32.1 to -3.8)	-18.3 (-31.7 to -4.1)	-35.2 (-46.2 to -20.9)	-26.6 (-37.7 to -13.5)
**High ETL Group**	**934 (873 to 1001)**	**781 (724 to 840)**	**859 (812 to 907)**	**1528 (1426 to 1630)**	**1221 (1134 to 1306)**	**1371 (1305 to 1440)**	**820 (761 to 885)**	**655 (606 to 708)**	**738 (698 to 782)**	**1088 (1016 to 1165)**	**780 (725 to 838)**	**924 (877 to 973)**	**-12.1 (-21.3 to -2.7)**	**-16.1 (-24.8 to -6.5)**	**-14.0 (-20.6 to -6.9)**	**-28.7 (-35.7 to -21.9)**	**-36.0 (-42.4 to -29.4)**	**-32.6 (-37.4 to -27.8)**
Himachal Pradesh	867 (779 to 970)	819 (730 to 905)	844 (778 to 912)	1349 (1205 to 1526)	1292 (1141 to 1438)	1322 (1213 to 1434)	728 (622 to 836)	534 (497 to 612)	633 (573 to 694)	993 (857 to 1132)	644 (600 to 732)	808 (735 to 881)	-16.1 (-30.3 to -0.8)	-34.8 (-43.9 to -22.7)	-25.0 (-34.1 to -15.8)	-26.4 (-38.7 to -13.8)	-50.1 (-57.3 to -40.7)	-38.9 (-46.1 to -31.5)
Punjab	931 (839 to 1031)	848 (766 to 938)	892 (822 to 962)	1537 (1377 to 1706)	1324 (1187 to 1474)	1434 (1320 to 1543)	878 (770 to 991)	725 (618 to 837)	806 (722 to 887)	1218 (1078 to 1364)	916 (787 to 1049)	1066 (959 to 1167)	-5.7 (-19.4 to 11.0)	-14.5 (-29.6 to 2.4)	-9.6 (-20.3 to 2.7)	-20.8 (-32.2 to -6.9)	-30.8 (-42.6 to -17.9)	-25.7 (-34.0 to -16.4)
Tamil Nadu	1071 (959 to 1185)	919 (816 to 1023)	996 (915 to 1085)	1733 (1556 to 1910)	1493 (1335 to 1651)	1614 (1495 to 1742)	865 (762 to 978)	670 (580 to 763)	768 (697 to 842)	1176 (1047 to 1316)	872 (761 to 986)	1018 (930 to 1110)	-19.2 (-31.5 to -4.9)	-27.1 (-38.9 to -12.2)	-22.8 (-32.5 to -12.4)	-32.2 (-41.8 to -20.5)	-41.6 (-50.6 to -30.6)	-36.9 (-43.9 to -29.1)
Goa	631 (548 to 718)	605 (530 to 686)	618 (545 to 701)	1225 (1086 to 1374)	1040 (907 to 1171)	1124 (998 to 1257)	605 (531 to 707)	502 (476 to 566)	554 (507 to 631)	886 (789 to 1022)	604 (574 to 677)	730 (674 to 823)	-4.1 (-20.2 to 15.7)	-17.1 (-28.3 to -1.1)	-10.3 (-22.8 to 6.2)	-27.7 (-38.9 to -14.7)	-41.9 (-49.3 to -31.0)	-35.1 (-43.2 to -24.1)
Kerala	690 (621 to 767)	483 (429 to 540)	585 (541 to 633)	1184 (1070 to 1318)	751 (665 to 839)	945 (878 to 1022)	702 (633 to 784)	603 (585 to 641)	651 (615 to 691)	851 (774 to 942)	592 (574 to 628)	703 (667 to 744)	1.7 (-12.7 to 19.3)	25.0 (10.6 to 41.6)	11.3 (1.0 to 23.5)	-28.1 (-38.0 to -16.8)	-21.2 (-30.2 to -10.9)	-25.6 (-32.4 to -18.0)

Table 6: Percent contribution of disease categories to total DALYs in states grouped by epidemiological transition level, 1990 and 2016

Disease categories	Percent of DALYS in 1990 95% UI					Percent of DALYS in 2016 95% UI				
	Low ETL group	Lower-middle ETL group	Higher-middle ETL group	High ETL group	India	Low ETL group	Lower-middle ETL group	Higher-middle ETL group	High ETL group	India
**Communicable, maternal, neonatal, and nutritional diseases**	**67.0 (64.7 to 69.6)**	**59.3 (56.8 to 62.0)**	**55.7 (53.2 to 58.5)**	**47.7 (44.5 to 51.2)**	**60.9 (58.6 to 63.5)**	**39.9 (37.7 to 42.7)**	**31.6 (29.9 to 33.8)**	**25.7 (24.3 to 27.4)**	**19.5 (18.0 to 21.3)**	**32.7 (31.0 to 34.8)**
HIV/AIDS and tuberculosis	5.3 (4.8 to 5.8)	5.9 (5.3 to 6.6)	4.8 (4.3 to 5.3)	4.3 (3.9 to 4.9)	5.1 (4.6 to 5.5)	4.8 (4.4 to 5.2)	5.3 (4.8 to 5.9)	3.6 (3.2 to 3.9)	2.8 (2.5 to 3.2)	4.2 (3.9 to 4.6)
Diarrhea, lower respiratory, and other common infectious diseases	38.6 (35.8 to 42.0)	30.3 (27.7 to 33.3)	28.1 (25.6 to 31.1)	23.3 (20.5 to 26.5)	33.2 (30.7 to 36.2)	16.5 (14.4 to 19.4)	10.6 (9.4 to 12.1)	8.8 (7.7 to 10.7)	7.0 (5.8 to 8.8)	12.7 (11.1 to 15.0)
Neglected tropical diseases and malaria	2.8 (1.7 to 4.0)	1.9 (1.2 to 3.0)	1.4 (1.0 to 1.8)	0.9 (0.7 to 1.3)	2.1 (1.4 to 2.8)	2.0 (1.3 to 2.5)	1.4 (0.8 to 1.9)	1.1 (0.7 to 1.3)	0.8 (0.6 to 1.0)	1.5 (1.0 to 1.9)
Maternal disorders	1.5 (1.3 to 1.7)	1.2 (1.0 to 1.4)	1.4 (1.2 to 1.6)	1.0 (0.8 to 1.2)	1.4 (1.2 to 1.5)	0.8 (0.7 to 1.0)	0.4 (0.4 to 0.5)	0.4 (0.3 to 0.4)	0.3 (0.2 to 0.3)	0.6 (0.5 to 0.7)
Neonatal disorders	13.0 (11.5 to 15.2)	15.0 (13.5 to 16.5)	15.1 (13.9 to 16.1)	14.1 (12.4 to 15.9)	13.9 (12.7 to 15.4)	9.5 (8.5 to 10.6)	8.4 (7.1 to 9.9)	6.5 (5.8 to 7.3)	4.2 (3.5 to 5.0)	7.9 (7.2 to 8.8)
Nutritional deficiencies	4.2 (3.4 to 5.1)	3.5 (2.7 to 4.4)	3.4 (2.7 to 4.3)	3.1 (2.3 to 4.0)	3.8 (3.0 to 4.6)	5.0 (4.0 to 6.3)	4.4 (3.4 to 5.7)	4.3 (3.3 to 5.5)	3.8 (2.9 to 4.8)	4.6 (3.6 to 5.8)
Other communicable, maternal, neonatal, and nutritional diseases	1.6 (1.3 to 2.0)	1.5 (1.2 to 1.9)	1.5 (1.2 to 1.8)	0.9 (0.7 to 1.2)	1.5 (1.2 to 1.8)	1.3 (1.2 to 1.4)	1.1 (1.0 to 1.2)	1.0 (0.9 to 1.1)	0.6 (0.5 to 0.7)	1.1 (1.0 to 1.2)
**Non-communicable diseases**	**25.6 (23.3 to 27.7)**	**32.3 (29.9 to 34.6)**	**34.6 (32.2 to 36.7)**	**41.3 (38.5 to 44.1)**	**30.5 (28.4 to 32.6)**	**49.0 (46.4 to 51.0)**	**56.5 (54.5 to 58.4)**	**61.7 (59.8 to 63.2)**	**67.4 (65.5 to 68.9)**	**55.4 (53.3 to 57.2)**
Neoplasms	2.0 (1.8 to 2.2)	2.3 (2.1 to 2.5)	2.5 (2.3 to 2.7)	2.9 (2.6 to 3.2)	2.3 (2.1 to 2.5)	4.7 (4.2 to 5.1)	5.0 (4.5 to 5.6)	5.4 (4.9 to 5.9)	5.6 (5.1 to 6.3)	5.0 (4.6 to 5.5)
Cardiovascular diseases	4.9 (4.4 to 5.3)	7.1 (6.4 to 7.7)	8.7 (7.9 to 9.3)	10.8 (9.7 to 12.0)	6.9 (6.3 to 7.4)	10.9 (10.0 to 11.9)	14.3 (12.9 to 15.8)	17.4 (15.9 to 19.1)	19.5 (17.5 to 21.6)	14.1 (12.9 to 15.3)
Chronic respiratory diseases	4.4 (3.8 to 4.8)	5.0 (4.3 to 5.5)	4.7 (4.2 to 5.1)	4.3 (3.9 to 4.7)	4.5 (4.0 to 4.9)	6.6 (6.0 to 7.3)	6.7 (6.1 to 7.3)	6.2 (5.7 to 6.8)	5.3 (4.8 to 6.2)	6.4 (5.8 to 7.0)
Cirrhosis and other chronic liver diseases	0.7 (0.6 to 0.9)	0.9 (0.8 to 1.1)	1.2 (1.1 to 1.3)	0.9 (0.8 to 1.3)	0.9 (0.8 to 1.0)	1.3 (1.2 to 1.7)	1.6 (1.4 to 1.9)	2.0 (1.8 to 2.4)	1.4 (1.2 to 2.0)	1.6 (1.4 to 1.8)
Digestive diseases	1.4 (1.0 to 1.7)	1.1 (1.0 to 1.2)	1.1 (1.0 to 1.3)	1.1 (1.0 to 1.4)	1.3 (1.1 to 1.4)	1.8 (1.6 to 2.0)	1.3 (1.1 to 1.6)	1.2 (1.1 to 1.6)	1.1 (1.0 to 1.6)	1.5 (1.4 to 1.7)
Neurological disorders	1.7 (1.3 to 2.1)	2.3 (1.7 to 2.8)	2.2 (1.7 to 2.7)	2.6 (2.0 to 3.2)	2.0 (1.5 to 2.5)	3.2 (2.5 to 3.9)	3.9 (3.2 to 4.8)	4.0 (3.2 to 4.9)	4.4 (3.5 to 5.3)	3.6 (2.9 to 4.4)
Mental and substance use disorders	2.4 (1.9 to 3.0)	3.1 (2.4 to 3.9)	3.4 (2.6 to 4.2)	3.9 (3.0 to 4.9)	2.9 (2.3 to 3.7)	4.8 (3.8 to 5.8)	5.7 (4.5 to 6.8)	6.4 (5.1 to 7.6)	6.8 (5.5 to 8.1)	5.6 (4.5 to 6.7)
Diabetes, urogenital, blood, and endocrine diseases	2.1 (1.9 to 2.4)	2.7 (2.4 to 3.1)	2.9 (2.6 to 3.2)	4.1 (3.7 to 4.6)	2.6 (2.4 to 2.9)	4.6 (4.3 to 5.1)	5.6 (5.2 to 6.0)	6.1 (5.7 to 6.5)	8.9 (8.2 to 9.3)	5.6 (5.2 to 6.0)
Musculoskeletal disorders	1.9 (1.4 to 2.4)	2.5 (1.9 to 3.1)	2.6 (1.9 to 3.2)	3.1 (2.4 to 3.9)	2.3 (1.7 to 2.9)	3.9 (3.0 to 4.8)	4.9 (3.8 to 6.0)	5.3 (4.2 to 6.5)	6.0 (4.9 to 7.3)	4.6 (3.6 to 5.7)
Other non-communicable diseases	4.1 (3.1 to 5.3)	5.4 (4.2 to 6.6)	5.3 (4.3 to 6.6)	7.6 (5.8 to 9.1)	4.9 (4.0 to 6.1)	7.1 (5.9 to 8.7)	7.6 (6.2 to 9.5)	7.6 (6.1 to 9.5)	8.2 (6.6 to 10.3)	7.4 (6.1 to 9.2)
**Injuries**	**7.4 (6.6 to 8.1)**	**8.3 (7.7 to 9.0)**	**9.7 (8.6 to 10.6)**	**11.0 (9.6 to 12.0)**	**8.6 (7.8 to 9.3)**	**11.1 (10.2 to 11.9)**	**11.8 (11.0 to 12.6)**	**12.7 (11.6 to 13.5)**	**13.1 (11.8 to 14.1)**	**11.9 (10.9 to 12.7)**
Transport injuries	1.5 (1.4 to 1.6)	1.7 (1.6 to 1.9)	2.0 (1.9 to 2.2)	2.1 (1.9 to 2.4)	1.7 (1.6 to 1.9)	3.1 (2.9 to 3.4)	3.3 (3.0 to 3.6)	3.4 (3.2 to 3.7)	3.8 (3.4 to 4.1)	3.3 (3.1 to 3.5)
Unintentional injuries	4.4 (3.7 to 4.9)	4.6 (4.0 to 5.1)	4.8 (4.1 to 5.4)	5.3 (4.4 to 5.9)	4.6 (4.0 to 5.2)	5.4 (4.6 to 5.9)	5.4 (4.8 to 5.8)	5.3 (4.7 to 5.8)	5.4 (4.7 to 5.9)	5.4 (4.7 to 5.8)
Self-harm and interpersonal violence	1.4 (1.3 to 1.6)	1.9 (1.7 to 2.2)	2.8 (2.3 to 3.1)	3.5 (2.8 to 4.0)	2.1 (1.8 to 2.3)	2.5 (2.3 to 2.8)	3.1 (2.8 to 3.5)	3.9 (3.2 to 4.4)	3.9 (3.1 to 4.5)	3.1 (2.7 to 3.5)
Forces of nature, conflict and terrorism, and executions and police conflict	0.0 (0.0 to 0.1)	0.1 (0.1 to 0.1)	0.1 (0.1 to 0.1)	0.1 (0.1 to 0.1)	0.1 (0.1 to 0.1)	0.0 (0.0 to 0.0)	0.0 (0.0 to 0.1)	0.0 (0.0 to 0.0)	0.0 (0.0 to 0.0)	0.0 (0.0 to 0.0)

Table 7: Percent change of DALY number and rates for major disease groups in states grouped by epidemiological transition level from 1990 to 2016			
	Percent change from 1990 to 2016 (95% UI)		
	Communicable, maternal, neonatal, and nutritional diseases	Non-communicable diseases	Injuries
**DALY numbers**			
Low ETL group	-48.7<br>(-52.9 to -44.2)	64.9<br>(55.3 to 76.1)	29.5<br>(18.0 to 45.5)
Lower-middle ETL group	-48.6<br>(-53.6 to -43.5)	68.5<br>(58.2 to 79.5)	36.6<br>(23.9 to 51.3)
Higher-middle ETL group	-59.9<br>(-63.3 to -56.3)	55.0<br>(47.6 to 62.1)	13.5<br>(4.4 to 23.8)
High ETL group	-65.8<br>(-70.5 to -60.9)	36.4<br>(28.5 to 45.8)	-0.2<br>(-9.9 to 10.1)
**All-ages DALY rate**			
Low ETL group	-68.4<br>(-71.0 to -65.7)	1.5<br>(-4.4 to 8.4)	-20.3<br>(-27.3 to -10.4)
Lower-middle ETL group	-67.6<br>(-70.7 to -64.4)	6.2<br>(-0.3 to 13.2)	-13.9<br>(-21.9 to -4.6)
Higher-middle ETL group	-72.8<br>(-75.2 to -70.4)	5.0<br>(-0.0 to 9.8)	-23.1<br>(-29.3 to -16.1)
High ETL group	-73.4<br>(-77.0 to -69.5)	6.2<br>(-0.0 to 13.4)	-22.3<br>(-29.9 to -14.3)
**Age-standardised DALY rate**			
Low ETL group	-59.7<br>(-62.8 to -56.4)	-9.4<br>(-14.2 to -4.0)	-17.3<br>(-23.3 to -9.9)
Lower-middle ETL group	-56.2<br>(-60.1 to -51.9)	-12.0<br>(-17.8 to -6.1)	-14.3<br>(-21.3 to -6.3)
Higher-middle ETL group	-61.7<br>(-64.7 to -58.6)	-16.3<br>(-20.4 to -11.9)	-25.6<br>(-30.9 to -19.7)
High ETL group	-62.1<br>(-66.7 to -57.2)	-17.3<br>(-21.8 to -12.0)	-25.9<br>(-33.1 to -18.8)

Table 8: Comparison of the percent change in prevalence of NCDs and incidence rate of injuries with the percent change in their DALY rate from 1990 to 2016, India

Non-communicable diseases	Percent change in all-ages prevalence from 1990 to 2016 (95% UI)	Percent change in all-ages DALY rate from 1990 to 2016 (95% UI)	Percent change in age-standardised prevalence from 1990 to 2016 (95% UI)	Percent change in age-standardised DALY rate from 1990 to 2016 (95% UI)
Ischemic heart disease	53.0 (50.2 to 55.4)	33.9 (24.7 to 43.6)	9.4 (7.3 to 11.2)	2.2 (-4.8 to 9.7)
COPD	29.2 (27.9 to 30.4)	-10.5 (-20.5 to 2.9)	-5.0 (-5.9 to -4.2)	-35.9 (-42.7 to -26.1)
Cerebrovascular disease	53.9 (47.2 to 60.3)	0.4 (-7.9 to 9.4)	12.2 (7.1 to 17.0)	-25.7 (-32.0 to -18.8)
Sense organ diseases	21.2 (20.4 to 22.0)	21.7 (20.1 to 23.3)	-0.9 (-1.2 to -0.6)	-4.4 (-5.3 to -3.5)
Low back & neck pain	9.3 (7.1 to 11.6)	9.0 (6.3 to 11.4)	-11.2 (-12.5 to -10.0)	-11.6 (-12.8 to -10.3)
Diabetes	64.3 (60.1 to 68.8)	80.0 (71.6 to 88.5)	29.3 (26.2 to 32.2)	39.6 (32.1 to 46.7)
Migraine	8.5 (7.7 to 9.3)	11.0 (9.6 to 12.3)	-2.7 (-3.2 to -2.3)	-0.7 (-1.6 to 0.1)
Skin diseases	4.9 (3.9 to 5.7)	1.7 (-1.4 to 4.9)	4.5 (4.1 to 4.9)	5.3 (2.1 to 8.6)
Congenital defects	-13.6 (-17.4 to -9.3)	-48.1 (-65.6 to -26.8)	-8.1 (-12.5 to -3.3)	-20.3 (-46.8 to 10.1)
Other musculoskeletal	17.8 (15.0 to 20.7)	18.0 (15.1 to 21.0)	-2.0 (-3.5 to -0.5)	-1.3 (-2.9 to 0.3)
Chronic kidney disease	26.1 (23.4 to 28.9)	12.2 (2.3 to 23.3)	-4.3 (-6.0 to -2.7)	-8.3 (-16.4 to 0.4)
Depressive disorders	11.1 (8.2 to 14.1)	8.4 (5.4 to 11.3)	-6.2 (-8.3 to -4.0)	-7.9 (-9.8 to -5.9)
Asthma	8.6 (6.1 to 11.4)	-44.3 (-55.2 to -33.5)	-3.8 (-5.3 to -2.1)	-53.6 (-64.1 to -44.0)
Anxiety disorders	6.4 (3.5 to 9.5)	6.2 (3.2 to 9.4)	-4.0 (-6.3 to -1.3)	-3.6 (-6.1 to -0.9)
Rheumatic heart disease	-1.1 (-3.4 to 1.3)	-32.7 (-43.9 to -22.0)	-10.8 (-13.0 to -8.7)	-39.8 (-50.3 to -28.7)
**Injuries**	**Percent change in all-ages incidence rate from 1990 to 2016 (95% UL)**	**Percent change in all-ages DALY rate from 1990 to 2016 (95% UL)**	**Percent change in age-standardised incidence rate from 1990 to 2016 (95% UL)**	**Percent change in age-standardised DALY rate from 1990 to 2016 (95% UL)**
Road injuries	55.8 (51.4 to 59.7)	8.3 (0.7 to 15.9)	43.4 (40.3 to 46.0)	3.9 (-2.9 to 10.6)
Self-harm	4.8 (1.3 to 8.1)	-14.8 (-24.4 to 0.1)	-3.2 (-5.5 to -1.2)	-19.5 (-28.2 to -5.7)
Falls	6.3 (2.2 to 10.5)	-7.2 (-23.0 to 4.7)	-0.4 (-2.1 to 1.4)	-12.6 (-25.1 to -4.2)
Drowning	-27.3 (-30.8 to -23.6)	-58.0 (-65.2 to -44.4)	-20.4 (-22.5 to -18.2)	-48.2 (-56.3 to -33.9)

Table 9: Ratio of the observed to expected DALY rate based on SDI for the top 30 causes in states grouped by epidemiological transition level, 2016						
		Observed versus expected ratio of DALY in 2016				
Rank	Causes	Low ETL group	Lower-middle ETL group	Higher-middle ETL group	High ETL group	India
1	Ischemic heart disease	1.00	1.27	1.36	1.65	1.22
2	Chronic obstructive pulmonary disease	2.47	2.34	2.17	1.84	2.28
3	Diarrheal diseases	1.44	2.15	2.25	2.69	2.47
4	Lower respiratory infections	0.71	1.02	0.73	0.70	0.90
5	Stroke	0.66	0.56	0.95	0.61	0.74
6	Iron-deficiency anemia	2.44	3.22	3.27	3.47	3.00
7	Neonatal preterm birth complications	0.73	1.12	0.92	0.73	0.87
8	Tuberculosis	2.53	5.62	3.23	3.82	3.61
9	Sense organ diseases	1.26	1.24	1.33	1.48	1.30
10	Road injuries	0.66	0.70	0.71	0.83	0.69
11	Self-harm	1.52	1.63	2.05	1.93	1.78
12	Low back and neck pain	0.84	0.79	0.81	0.84	0.83
13	Diabetes mellitus	0.79	0.89	1.00	1.76	0.95
14	Other neonatal disorders	2.31	2.48	1.76	1.07	2.41
15	Migraine	1.26	1.21	1.24	1.23	1.24
16	Skin and subcutaneous diseases	0.89	0.86	0.84	0.81	0.87
17	Falls	1.77	1.76	1.66	1.94	1.81
18	Congenital birth defects	0.61	0.65	0.51	0.54	0.59
19	Other musculoskeletal disorders	1.47	1.49	1.63	1.68	1.50
20	Chronic kidney disease	0.84	1.15	1.41	2.18	1.17
21	Depressive disorders	0.94	0.94	1.17	1.25	1.05
22	Neonatal encephalopathy due to birth asphyxia	0.62	1.11	0.82	0.76	0.80
23	Asthma	1.62	1.56	1.37	1.06	1.55
24	Intestinal infectious diseases	26.94	78.59	54.78	78.02	61.75
25	HIV/AIDS	4.04	5.43	5.31	3.86	4.19
26	Anxiety disorders	0.85	0.79	0.86	0.86	0.84
27	Meningitis	1.03	1.94	1.46	1.49	1.46
28	Rheumatic heart disease	1.78	2.11	2.25	2.14	2.10
29	Protein-energy malnutrition	1.02	2.49	1.88	1.91	1.96
30	Drowning	0.46	0.59	0.61	0.58	0.54

Table 10: Change in all-age and age-standardised DALY rates in each state of India from 1990 to 2016

	1990						2016						Percent Change					
	All Age			Age Standardised			All Age			Age Standardised			All age			Age Standardised		
	Men	Women	Both sexes	Men	Women	Both sexes	Men	Women	Both sexes	Men	Women	Both sexes	Men	Women	Both sexes	Men	Women	Both sexes
**India**	**61725 (58784 to 64698)**	**62752 (59384 to 66432)**	**62221 (59050 to 65459)**	**65648 (62026 to 69464)**	**63997 (60156 to 68240)**	**64810 (61303 to 68653)**	**36745 (34006 to 39832)**	**34025 (30706 to 37678)**	**35435 (32540 to 38775)**	**43794 (40714 to 47200)**	**38966 (35395 to 42807)**	**41362 (38221 to 44965)**	**-40.5 (-43.2 to -37.7)**	**-45.8 (-49.0 to -42.7)**	**-43.1 (-45.9 to -40.3)**	**-33.3 (-35.8 to -30.8)**	**-39.1 (-42.0 to -36.3)**	**-36.2 (-38.6 to -33.8)**
**Low ETL Group**	**69405 (65932 to 73114)**	**74875 (70856 to 79154)**	**72020 (68601 to 75714)**	**71940 (67918 to 76236)**	**74059 (69664 to 78749)**	**72935 (68955 to 76996)**	**38805 (35878 to 42079)**	**37524 (34048 to 41232)**	**38194 (35013 to 41547)**	**47104 (43627 to 50916)**	**43786 (39813 to 47988)**	**45460 (41888 to 49149)**	**-44.1 (-47.4 to -40.7)**	**-49.9 (-53.5 to -46.3)**	**-47.0 (-50.1 to -43.9)**	**-34.5 (-37.9 to -31.1)**	**-40.9 (-44.3 to -37.3)**	**-37.7 (-40.8 to -34.7)**
Bihar	62009 (54618 to 69674)	71031 (61157 to 81177)	66328 (58145 to 75003)	64222 (57667 to 71143)	69352 (61280 to 78086)	66680 (60350 to 73706)	35937 (32014 to 40340)	38328 (33812 to 43045)	37074 (33027 to 41420)	42462 (37914 to 47703)	44481 (39148 to 50076)	43400 (39063 to 48044)	-41.9 (-50.2 to -33.1)	-45.8 (-54.4 to -36.2)	-43.9 (-51.9 to -34.7)	-33.8 (-42.2 to -25.2)	-35.7 (-44.1 to -26.0)	-34.8 (-42.0 to -27.5)
Jharkhand	67282 (58797 to 76025)	71091 (61938 to 80737)	69110 (60890 to 76967)	68388 (60734 to 76366)	70197 (61948 to 78361)	69258 (62328 to 75783)	34807 (30960 to 39311)	35413 (30864 to 40253)	35095 (31408 to 39082)	43738 (38975 to 49202)	44041 (38444 to 49821)	43858 (39522 to 48623)	-48.1 (-55.1 to -40.3)	-50.1 (-56.7 to -42.4)	-49.1 (-55.0 to -42.8)	-35.9 (-43.8 to -26.9)	-37.2 (-44.8 to -28.9)	-36.6 (-42.8 to -30.2)
Uttar Pradesh	71999 (66849 to 77375)	82453 (75697 to 88820)	76900 (71739 to 81895)	75133 (69507 to 80919)	81746 (74248 to 88545)	78216 (72969 to 83295)	39983 (35792 to 44250)	39141 (34884 to 43818)	39585 (35741 to 43500)	49261 (44057 to 54365)	46209 (41264 to 51492)	47754 (43248 to 52359)	-44.4 (-50.4 to -37.9)	-52.5 (-57.6 to -46.9)	-48.5 (-53.3 to -43.3)	-34.4 (-41.3 to -27.1)	-43.4 (-49.3 to -36.9)	-38.9 (-43.9 to -33.4)
Rajasthan	63281 (58860 to 68455)	65255 (60554 to 70417)	64223 (60104 to 68583)	68440 (62821 to 74818)	64901 (59633 to 70753)	66556 (61940 to 71686)	38076 (34029 to 42491)	34903 (30810 to 38847)	36556 (33110 to 40428)	46800 (41722 to 52296)	39522 (34774 to 43941)	43020 (39077 to 47459)	-39.8 (-45.9 to -33.3)	-46.5 (-51.7 to -41.0)	-43.1 (-47.7 to -38.2)	-31.5 (-38.9 to -23.1)	-39.1 (-45.2 to -32.9)	-35.3 (-40.1 to -30.2)
Meghalaya	56356 (50163 to 62432)	48429 (43016 to 53642)	52477 (47057 to 57915)	61384 (55280 to 67571)	54804 (48612 to 61192)	58242 (52577 to 63878)	32186 (28226 to 37039)	26471 (22617 to 30610)	29367 (25550 to 33850)	44235 (39024 to 50403)	35147 (30331 to 40509)	39653 (34848 to 45097)	-42.7 (-50.8 to -32.1)	-45.2 (-53.4 to -35.6)	-43.9 (-51.8 to -34.2)	-27.8 (-37.1 to -17.1)	-35.8 (-44.2 to -26.4)	-31.8 (-39.4 to -22.5)
Assam	66062 (61119 to 71726)	65547 (59642 to 71948)	65814 (61436 to 70831)	73846 (67515 to 80986)	73222 (65321 to 81481)	73611 (68211 to 79627)	41824 (37045 to 47113)	37889 (33112 to 42958)	39915 (35734 to 44542)	51379 (45730 to 57572)	46011 (40417 to 51851)	48791 (43870 to 53933)	-36.6 (-43.9 to -28.7)	-42.1 (-48.7 to -35.1)	-39.3 (-45.2 to -33.3)	-30.3 (-38.6 to -21.9)	-37.2 (-44.6 to -28.8)	-33.7 (-40.0 to -27.3)
Chhattisgarh	72199 (63528 to 81458)	64557 (58011 to 71510)	68427 (61675 to 76022)	72612 (64950 to 80691)	65829 (59129 to 73061)	69184 (62787 to 75435)	40372 (35955 to 45134)	37212 (32687 to 42337)	38810 (34812 to 43314)	49205 (43994 to 54737)	43068 (38022 to 48711)	46020 (41560 to 51055)	-43.9 (-52.3 to -34.8)	-42.3 (-49.8 to -34.2)	-43.2 (-50.0 to -35.8)	-32.1 (-40.9 to -22.2)	-34.5 (-42.4 to -25.5)	-33.4 (-39.9 to -26.4)
Madhya Pradesh	73053 (67700 to 78556)	78428 (72989 to 84131)	75649 (70999 to 80346)	73103 (67293 to 79538)	74182 (67980 to 80515)	73562 (68575 to 78866)	39370 (35228 to 43883)	35833 (31693 to 40417)	37678 (33707 to 41493)	47557 (42654 to 53015)	41048 (36484 to 46121)	44310 (39730 to 48609)	-46.1 (-51.5 to -40.1)	-54.3 (-58.9 to -49.6)	-50.2 (-54.3 to -45.9)	-34.9 (-41.6 to -27.6)	-44.6 (-50.4 to -38.4)	-39.8 (-44.4 to -34.8)
Odisha	79486 (72405 to 87259)	76452 (69563 to 83667)	77985 (71580 to 84744)	79040 (71864 to 86063)	76430 (69131 to 83647)	77759 (71588 to 83730)	40566 (36027 to 45485)	37538 (32629 to 42897)	39091 (34852 to 43580)	45771 (40772 to 51044)	42185 (36896 to 47968)	44027 (39488 to 48870)	-48.9 (-54.8 to -42.8)	-50.9 (-56.8 to -44.4)	-49.8 (-54.7 to -44.4)	-42.0 (-48.4 to -35.3)	-44.7 (-51.2 to -37.9)	-43.4 (-48.6 to -37.9)
**Lower-middle ETL Group**	**56290 (52502 to 60216)**	**55204 (51097 to 59314)**	**55764 (52149 to 59536)**	**61275 (56891 to 66134)**	**57214 (52484 to 61674)**	**59201 (55027 to 63528)**	**35661 (32036 to 39343)**	**31900 (28328 to 35978)**	**33860 (30571 to 37392)**	**43236 (39070 to 47350)**	**36606 (32691 to 41098)**	**39878 (36213 to 43704)**	**-36.6 (-41.8 to -31.2)**	**-42.2 (-47.2 to -37.4)**	**-39.3 (-43.6 to -35.2)**	**-29.4 (-35.1 to -23.6)**	**-36.0 (-41.2 to -31.0)**	**-32.6 (-36.8 to -28.4)**
Arunachal Pradesh	56499 (51186 to 62287)	57057 (51941 to 62119)	56757 (51963 to 61753)	62405 (56000 to 69085)	60266 (54149 to 66431)	61414 (55418 to 67325)	30489 (26502 to 34523)	25200 (21738 to 28892)	27964 (24452 to 31652)	40851 (35794 to 46087)	34040 (29433 to 39007)	37698 (33063 to 42581)	-46.0 (-53.2 to -38.6)	-55.8 (-61.2 to -49.9)	-50.7 (-56.4 to -44.8)	-34.4 (-42.7 to -25.9)	-43.5 (-50.1 to -36.3)	-38.6 (-45.5 to -31.8)
Mizoram	39890 (36308 to 43725)	38349 (34488 to 42181)	39152 (35702 to 42758)	50188 (45449 to 55460)	45830 (40945 to 50981)	48090 (43590 to 52920)	32697 (28717 to 36967)	26455 (22935 to 30300)	29636 (25950 to 33399)	40629 (35766 to 45847)	32083 (27969 to 36620)	36377 (31984 to 40895)	-18.0 (-26.6 to -8.0)	-31.0 (-38.7 to -22.5)	-24.3 (-32.0 to -15.7)	-19.0 (-28.1 to -8.7)	-29.9 (-37.5 to -21.1)	-24.3 (-31.9 to -15.6)
Nagaland	47020 (40969 to 53938)	46784 (40402 to 53595)	46909 (40999 to 53567)	53446 (47578 to 60029)	50991 (45083 to 57475)	52342 (46520 to 58714)	28041 (24225 to 32248)	22472 (19115 to 26300)	25398 (21947 to 29260)	38932 (33698 to 44687)	31215 (26784 to 36515)	35386 (30710 to 40835)	-40.1 (-49.7 to -29.0)	-51.8 (-59.7 to -42.3)	-45.7 (-54.2 to -35.2)	-27.0 (-37.5 to -15.2)	-38.6 (-47.3 to -28.3)	-32.3 (-41.5 to -21.5)
Uttarakhand	59667 (53597 to 65505)	53969 (49045 to 59357)	56864 (52010 to 61900)	67347 (60683 to 73960)	61316 (54802 to 67906)	64368 (58810 to 70083)	38917 (34596 to 43325)	32164 (28076 to 36027)	35622 (31880 to 39208)	47144 (42014 to 52434)	37365 (32707 to 41531)	42190 (37922 to 46186)	-34.7 (-42.5 to -26.1)	-40.4 (-47.0 to -33.5)	-37.3 (-43.1 to -31.2)	-29.9 (-37.7 to -20.9)	-39.0 (-45.8 to -31.2)	-34.4 (-40.2 to -28.5)
Gujarat	56504 (52264 to 60970)	56461 (51883 to 60953)	56483 (52517 to 60700)	61090 (55999 to 66745)	57392 (52195 to 62486)	59141 (54598 to 63954)	35650 (31649 to 39825)	32799 (28801 to 37341)	34291 (30648 to 38232)	42932 (38163 to 47633)	37036 (32763 to 41894)	39909 (35925 to 44241)	-36.9 (-43.4 to -30.1)	-41.9 (-48.2 to -36.1)	-39.3 (-44.3 to -34.2)	-29.7 (-36.6 to -22.0)	-35.4 (-42.0 to -28.8)	-32.5 (-37.7 to -27.0)
Tripura	58391 (52827 to 64462)	54772 (49389 to 60369)	56629 (51446 to 62105)	63011 (56923 to 69839)	58501 (52596 to 65125)	60836 (55162 to 67176)	37631 (33069 to 42540)	31683 (27277 to 36489)	34745 (30386 to 39540)	44753 (39401 to 50483)	36417 (31436 to 41685)	40642 (35660 to 46068)	-35.4 (-44.1 to -25.5)	-42.1 (-50.0 to -33.7)	-38.6 (-46.3 to -29.7)	-28.9 (-37.9 to -18.4)	-37.7 (-45.8 to -28.8)	-33.1 (-41.1 to -24.4)
Sikkim	50257 (44579 to 56214)	49035 (43427 to 55213)	49683 (44256 to 55199)	55822 (49698 to 62047)	53434 (47328 to 59663)	54750 (48853 to 60652)	28915 (25443 to 33045)	23228 (19954 to 27083)	26277 (23038 to 30094)	36300 (32003 to 41443)	29456 (25399 to 33956)	33289 (29275 to 37899)	-42.4 (-49.8 to -34.3)	-52.6 (-58.5 to -46.2)	-47.0 (-53.2 to -40.3)	-34.9 (-42.9 to -26.2)	-44.8 (-51.3 to -37.4)	-39.1 (-45.9 to -32.1)
Manipur	49159 (44141 to 54872)	44940 (40265 to 49865)	47088 (42279 to 51843)	52533 (47501 to 58310)	49326 (43915 to 55152)	50990 (45934 to 56281)	33085 (29026 to 37395)	26937 (23184 to 31005)	30061 (26292 to 34067)	40795 (36013 to 46040)	32959 (28555 to 37840)	36943 (32551 to 41709)	-32.6 (-41.1 to -23.4)	-40.0 (-46.9 to -31.3)	-36.1 (-43.2 to -27.8)	-22.2 (-31.2 to -12.6)	-33.1 (-41.0 to -23.6)	-27.5 (-35.1 to -18.7)
**Higher-middle ETL Group**	**57078 (53831 to 60486)**	**55285 (51962 to 59203)**	**56211 (52983 to 59731)**	**62722 (58814 to 66860)**	**58799 (54926 to 63186)**	**60781 (57013 to 64859)**	**34960 (31971 to 38330)**	**31208 (27653 to 35065)**	**33146 (30001 to 36679)**	**41255 (37966 to 44926)**	**35349 (31564 to 39358)**	**38282 (34894 to 42027)**	**-38.8 (-42.5 to -35.3)**	**-43.6 (-47.5 to -39.6)**	**-41.1 (-44.6 to -37.8)**	**-34.2 (-37.8 to -30.7)**	**-39.9 (-43.6 to -36.3)**	**-37.0 (-40.2 to -34.0)**
Haryana	53637 (49234 to 58095)	55584 (51182 to 60223)	54539 (50666 to 58887)	60273 (54796 to 66138)	58131 (52809 to 63820)	59095 (54349 to 63899)	39204 (34996 to 43811)	32719 (28441 to 36928)	36191 (32489 to 40082)	48277 (43343 to 53685)	37624 (32901 to 42287)	42940 (38797 to 47307)	-26.9 (-34.5 to -18.9)	-41.1 (-47.8 to -35.1)	-33.6 (-39.3 to -28.2)	-19.8 (-28.4 to -10.7)	-35.2 (-42.8 to -28.2)	-27.3 (-33.4 to -21.5)
Delhi	43865 (39505 to 48314)	44025 (39472 to 48856)	43937 (40169 to 48111)	51778 (46584 to 57446)	49023 (43877 to 55027)	50503 (46183 to 55469)	28214 (24635 to 31952)	25345 (21721 to 29350)	26898 (23583 to 30392)	36344 (31797 to 40856)	31315 (27093 to 35959)	33921 (30053 to 38068)	-35.6 (-44.3 to -26.3)	-42.4 (-49.7 to -34.8)	-38.7 (-45.7 to -31.7)	-29.7 (-38.7 to -20.4)	-36.1 (-44.1 to -28.1)	-32.8 (-39.7 to -25.9)
Telangana	57321 (51613 to 63090)	54010 (48670 to 59903)	55686 (50916 to 60655)	59703 (53539 to 66207)	57653 (51492 to 64439)	58730 (52980 to 64516)	33057 (28887 to 37454)	30196 (26112 to 34868)	31646 (27776 to 35922)	38673 (33998 to 43490)	33682 (29334 to 38732)	36142 (31893 to 40906)	-42.3 (-49.9 to -34.3)	-44.0 (-50.7 to -36.6)	-43.1 (-49.2 to -36.7)	-35.1 (-42.9 to -26.8)	-41.5 (-48.5 to -33.5)	-38.4 (-44.8 to -31.5)
Andhra Pradesh	63846 (57613 to 70219)	62562 (56649 to 68867)	63212 (57482 to 69028)	66760 (60214 to 73349)	65154 (58780 to 72125)	65975 (59932 to 72250)	37004 (32722 to 41735)	32376 (28090 to 37545)	34721 (30564 to 39495)	43041 (38263 to 48279)	36119 (31565 to 41614)	39497 (35011 to 44650)	-42.0 (-48.9 to -34.6)	-48.2 (-54.4 to -41.5)	-45.0 (-50.9 to -38.6)	-35.4 (-42.7 to -27.5)	-44.5 (-50.8 to -37.7)	-40.1 (-46.1 to -33.3)
Jammu and Kashmir	51683 (47321 to 56386)	53352 (48541 to 58778)	52469 (48510 to 56757)	59825 (54497 to 65727)	59622 (53574 to 66062)	59783 (54946 to 65044)	31853 (28162 to 35880)	28660 (24993 to 32526)	30363 (27191 to 33705)	40398 (35901 to 45301)	35500 (31250 to 40038)	38056 (34360 to 41946)	-38.3 (-45.2 to -31.2)	-46.3 (-52.4 to -40.0)	-42.1 (-47.5 to -37.0)	-32.4 (-40.1 to -24.7)	-40.4 (-47.4 to -33.5)	-36.3 (-41.8 to -30.7)
Karnataka	58857 (53704 to 63577)	53416 (48940 to 58160)	56186 (51855 to 60349)	63460 (57367 to 69203)	56122 (50976 to 61948)	59819 (55162 to 64658)	37122 (32699 to 41417)	33363 (29058 to 38097)	35277 (31442 to 39484)	43375 (38372 to 48225)	37240 (32633 to 42225)	40237 (36039 to 44847)	-36.9 (-44.3 to -29.2)	-37.5 (-44.5 to -30.1)	-37.2 (-43.1 to -31.2)	-31.6 (-39.6 to -23.5)	-33.6 (-40.6 to -25.9)	-32.7 (-38.7 to -26.8)
West Bengal	58539 (53656 to 63599)	59752 (54584 to 65065)	59120 (54803 to 63728)	64893 (59351 to 70896)	63965 (58170 to 70035)	64450 (59502 to 69554)	34570 (30473 to 39095)	31517 (27138 to 35958)	33078 (29394 to 37119)	40701 (36095 to 45748)	36539 (31812 to 41336)	38650 (34657 to 43139)	-40.9 (-47.3 to -33.5)	-47.2 (-53.0 to -40.8)	-44.0 (-49.1 to -38.7)	-37.2 (-43.8 to -29.7)	-42.8 (-48.7 to -36.1)	-40.0 (-45.0 to -34.8)
Maharashtra	54787 (50695 to 59680)	50998 (46534 to 55740)	52955 (49253 to 57119)	61953 (56877 to 67731)	54809 (49585 to 59906)	58363 (54045 to 63065)	34576 (30600 to 38821)	30614 (26267 to 35027)	32677 (29214 to 36534)	40067 (35676 to 44824)	33709 (29239 to 38421)	36851 (33087 to 40926)	-36.9 (-43.3 to -30.0)	-40.0 (-46.7 to -33.3)	-38.3 (-43.7 to -33.2)	-35.3 (-41.8 to -28.2)	-38.5 (-45.2 to -32.0)	-36.8 (-42.2 to -31.7)
Union Territories other than Delhi	36218 (31801 to 40528)	37817 (33520 to 42116)	36973 (32849 to 41160)	44251 (39132 to 49313)	42905 (37994 to 47843)	43564 (38861 to 48311)	28219 (24127 to 32623)	25046 (21045 to 29682)	26755 (22753 to 31053)	35533 (30484 to 41013)	29745 (25007 to 35008)	32662 (27888 to 37545)	-22.0 (-32.3 to -11.3)	-33.8 (-42.0 to -24.4)	-27.6 (-36.2 to -18.8)	-19.6 (-29.8 to -8.8)	-30.6 (-39.3 to -21.3)	-25.0 (-33.2 to -15.5)
**High ETL Group**	**50719 (46353 to 54972)**	**46751 (42463 to 51172)**	**48761 (44656 to 52867)**	**54991 (50598 to 59507)**	**49196 (44736 to 53865)**	**52067 (47874 to 56375)**	**33987 (30562 to 38055)**	**29443 (25669 to 33406)**	**31732 (28319 to 35489)**	**36974 (33453 to 41112)**	**30879 (27105 to 34826)**	**33802 (30392 to 37571)**	**-33.0 (-39.4 to -26.4)**	**-37.0 (-43.0 to -30.3)**	**-34.9 (-40.5 to -29.3)**	**-32.7 (-38.4 to -27.2)**	**-37.2 (-42.4 to -31.6)**	**-35.1 (-39.5 to -30.5)**
Himachal Pradesh	46632 (41756 to 51507)	45010 (40484 to 49687)	45830 (41684 to 50242)	49541 (44604 to 54970)	48583 (43342 to 54065)	49087 (44641 to 53814)	30914 (26838 to 34995)	25627 (22194 to 29406)	28320 (24744 to 31890)	35271 (30847 to 39708)	28080 (24554 to 31977)	31556 (27828 to 35283)	-33.6 (-42.0 to -25.3)	-43.0 (-49.4 to -36.2)	-38.2 (-44.4 to -32.3)	-28.7 (-37.7 to -19.9)	-42.2 (-48.5 to -35.3)	-35.7 (-41.8 to -29.7)
Punjab	48958 (44243 to 53885)	49578 (44413 to 54854)	49249 (44431 to 53926)	54498 (49175 to 60226)	52234 (46767 to 57989)	53359 (48601 to 58455)	35538 (31177 to 40028)	31765 (27329 to 36491)	33766 (29822 to 37814)	40839 (36038 to 45870)	35019 (30385 to 39829)	37923 (33767 to 42198)	-27.3 (-36.1 to -18.3)	-35.9 (-44.4 to -27.5)	-31.4 (-38.5 to -24.3)	-25.0 (-34.0 to -15.7)	-32.9 (-40.8 to -24.8)	-28.9 (-35.4 to -22.2)
Tamil Nadu	59156 (52587 to 65566)	55666 (49273 to 62075)	57432 (51624 to 63627)	62514 (56117 to 68651)	58217 (52281 to 64260)	60392 (55219 to 65920)	36061 (31858 to 40881)	30957 (26610 to 35366)	33527 (29744 to 37649)	39314 (34912 to 44277)	33132 (28648 to 37683)	36154 (32302 to 40478)	-38.9 (-47.3 to -29.9)	-44.3 (-52.0 to -36.2)	-41.5 (-48.5 to -34.0)	-37.0 (-44.6 to -28.7)	-43.0 (-50.0 to -35.8)	-40.1 (-46.1 to -33.5)
Goa	40043 (33945 to 46025)	36891 (32064 to 41771)	38494 (33060 to 43588)	45124 (40000 to 50363)	40219 (35470 to 45188)	42572 (37572 to 47693)	28677 (24652 to 33274)	24614 (21088 to 28609)	26687 (23000 to 30855)	31329 (27044 to 36140)	25010 (21459 to 28994)	28024 (24197 to 32251)	-28.1 (-39.7 to -15.2)	-33.2 (-42.3 to -23.2)	-30.5 (-40.3 to -19.2)	-30.4 (-40.2 to -20.2)	-37.8 (-45.2 to -29.9)	-34.1 (-42.0 to -25.1)
Kerala	36476 (32744 to 40299)	28944 (25404 to 32763)	32646 (29103 to 36073)	41801 (37436 to 46426)	31790 (27737 to 35964)	36506 (32604 to 40497)	29044 (25268 to 32868)	25683 (22284 to 29703)	27301 (23764 to 31171)	29714 (25919 to 33593)	24752 (21587 to 28503)	26987 (23611 to 30705)	-20.3 (-29.3 to -11.1)	-11.3 (-17.8 to -4.4)	-16.4 (-23.0 to -9.7)	-28.8 (-36.8 to -20.9)	-22.1 (-27.7 to -16.1)	-26.1 (-31.4 to -20.7)

Table 11: Change in life expectancy in the states of India from 1990 to 2016

	1990			2016			Change from 1990 to 2016		
	Men	Women	Both sexes	Men	Women	Both sexes	Men	Women	Both sexes
**India**	**58.3 (57.7 to 58.9)**	**59.7 (59.0 to 60.3)**	**59.0 (58.5 to 59.4)**	**66.9 (66.2 to 67.6)**	**70.3 (69.6 to 71.0)**	**68.5 (67.9 to 69.1)**	**8.6 (7.8 to 9.5)**	**10.6 (9.7 to 11.6)**	**9.6 (8.8 to 10.3)**
**Low ETL group**	**56.5 (55.4 to 57.5)**	**56.0 (54.8 to 57.2)**	**56.2 (55.4 to 57.2)**	**65.7 (64.5 to 66.7)**	**67.7 (66.6 to 68.8)**	**66.6 (65.7 to 67.5)**	**9.2 (7.7 to 10.7)**	**11.7 (10.1 to 13.3)**	**10.4 (9.1 to 11.5)**
Bihar	58.9 (57.5 to 60.5)	58.0 (56.2 to 59.7)	58.5 (57.3 to 59.6)	67.7 (65.8 to 69.7)	67.7 (65.8 to 69.5)	67.7 (66.3 to 69.0)	8.8 (6.5 to 11.1)	9.7 (7.2 to 12.2)	9.2 (7.4 to 11.0)
Jharkhand	57.3 (55.1 to 59.2)	57.7 (55.7 to 59.7)	57.5 (56.0 to 59.0)	67.1 (65.1 to 68.9)	67.8 (65.9 to 69.8)	67.4 (66.1 to 68.9)	9.8 (6.9 to 12.6)	10.1 (7.4 to 12.7)	9.9 (8.1 to 11.9)
Uttar Pradesh	54.9 (53.3 to 56.4)	53.5 (51.8 to 55.3)	54.2 (53.1 to 55.6)	64.6 (62.8 to 66.2)	66.8 (65.1 to 68.4)	65.6 (64.3 to 66.9)	9.7 (7.5 to 11.9)	13.3 (10.7 to 15.6)	11.4 (9.4 to 13.1)
Rajasthan	57.2 (55.6 to 58.8)	59.5 (57.7 to 61.1)	58.3 (57.0 to 59.5)	65.5 (63.7 to 67.4)	70.2 (68.5 to 71.9)	67.7 (66.3 to 69.1)	8.3 (5.8 to 10.6)	10.7 (8.4 to 13.0)	9.4 (7.8 to 11.0)
Meghalaya	59.8 (57.9 to 61.7)	63.2 (61.3 to 65.1)	61.4 (59.7 to 63.1)	66.8 (64.9 to 68.6)	72.4 (70.6 to 74.1)	69.5 (67.7 to 71.1)	7.0 (4.5 to 9.4)	9.2 (6.6 to 11.7)	8.1 (5.8 to 10.2)
Assam	55.7 (53.9 to 57.5)	56.6 (54.5 to 58.6)	56.1 (54.7 to 57.4)	63.6 (61.3 to 65.8)	67.0 (65.0 to 68.9)	65.1 (63.5 to 66.7)	7.9 (5.3 to 10.5)	10.4 (7.5 to 13.1)	9.0 (7.0 to 11.2)
Chhattisgarh	55.8 (53.9 to 57.6)	59.0 (56.9 to 60.8)	57.3 (55.8 to 58.7)	64.6 (62.5 to 66.5)	68.3 (66.6 to 70.1)	66.4 (65.0 to 67.8)	8.8 (6.3 to 11.5)	9.4 (6.8 to 12.1)	9.1 (7.2 to 11.1)
Madhya Pradesh	55.6 (54.0 to 57.2)	55.9 (54.3 to 57.7)	55.8 (54.5 to 56.9)	65.3 (63.4 to 67.1)	69.3 (67.6 to 71.0)	67.2 (65.8 to 68.5)	9.7 (7.2 to 12.0)	13.4 (10.9 to 15.7)	11.4 (9.6 to 13.1)
Odisha	53.7 (51.4 to 55.9)	55.3 (52.9 to 57.5)	54.5 (52.5 to 56.4)	66.1 (64.2 to 67.9)	68.6 (66.7 to 70.4)	67.3 (65.9 to 68.7)	12.4 (9.7 to 15.1)	13.3 (10.4 to 16.1)	12.8 (10.7 to 15.0)
**Lower-middle ETL group**	**55.2 (54.1 to 56.3)**	**56.5 (55.4 to 57.6)**	**55.8 (55.0 to 56.6)**	**65.1 (64.0 to 66.2)**	**68.6 (67.7 to 69.6)**	**66.8 (65.9 to 67.6)**	**9.9 (8.5 to 11.3)**	**12.2 (10.8 to 13.6)**	**11.0 (9.9 to 12.0)**
Arunachal Pradesh	59.4 (57.5 to 61.3)	60.9 (59.0 to 63.0)	60.0 (58.3 to 61.9)	68.2 (66.2 to 70.3)	72.8 (70.9 to 74.7)	70.2 (68.3 to 72.1)	8.8 (6.2 to 11.4)	11.9 (9.2 to 14.5)	10.2 (7.9 to 12.5)
Mizoram	64.0 (62.3 to 65.8)	66.7 (64.9 to 68.4)	65.2 (63.7 to 66.9)	68.3 (66.1 to 70.4)	73.8 (71.8 to 75.8)	70.9 (68.9 to 72.6)	4.3 (1.8 to 6.7)	7.1 (4.7 to 9.4)	5.6 (3.4 to 7.8)
Nagaland	63.1 (61.1 to 65.2)	65.0 (62.9 to 67.1)	63.9 (62.0 to 65.8)	69.1 (67.1 to 71.1)	74.5 (72.5 to 76.5)	71.4 (69.7 to 73.1)	6.0 (3.2 to 8.7)	9.5 (6.8 to 12.1)	7.5 (5.0 to 9.8)
Uttarakhand	57.8 (56.0 to 59.7)	60.5 (58.7 to 62.4)	59.1 (57.8 to 60.5)	65.4 (63.5 to 67.1)	71.1 (69.1 to 73.1)	68.1 (66.8 to 69.5)	7.5 (5.0 to 10.0)	10.6 (8.1 to 13.0)	8.9 (7.1 to 10.8)
Gujarat	59.8 (58.2 to 61.3)	62.2 (60.6 to 63.8)	61.0 (59.8 to 62.2)	67.3 (65.6 to 69.0)	71.5 (69.8 to 73.2)	69.3 (68.0 to 70.6)	7.5 (5.2 to 9.7)	9.2 (7.1 to 11.5)	8.3 (6.5 to 10.0)
Tripura	59.0 (57.0 to 60.8)	61.6 (59.6 to 63.5)	60.2 (58.5 to 61.8)	66.3 (64.2 to 68.4)	71.5 (69.5 to 73.6)	68.7 (66.8 to 70.7)	7.3 (4.6 to 10.0)	9.9 (7.4 to 12.5)	8.5 (6.2 to 10.9)
Sikkim	61.9 (59.9 to 63.8)	63.6 (61.7 to 65.4)	62.6 (60.8 to 64.4)	70.5 (68.4 to 72.5)	75.8 (73.9 to 77.4)	72.7 (70.9 to 74.3)	8.6 (6.0 to 11.4)	12.2 (9.7 to 14.5)	10.1 (7.8 to 12.3)
Manipur	63.0 (61.1 to 64.5)	65.0 (63.0 to 66.9)	63.9 (62.2 to 65.5)	68.0 (66.0 to 69.9)	72.9 (71.0 to 74.6)	70.3 (68.4 to 72.0)	5.1 (2.8 to 7.5)	7.9 (5.3 to 10.3)	6.3 (4.2 to 8.6)
**Higher-Middle ETL group**	**59.4 (58.7 to 60.2)**	**61.5 (60.7 to 62.3)**	**60.4 (59.8 to 61.0)**	**68.2 (67.2 to 69.1)**	**72.2 (71.4 to 73.0)**	**70.1 (69.4 to 70.8)**	**8.8 (7.5 to 10.0)**	**10.7 (9.5 to 11.7)**	**9.7 (8.7 to 10.5)**
Haryana	60.2 (58.5 to 61.9)	62.1 (60.3 to 63.9)	61.1 (59.8 to 62.4)	65.0 (63.3 to 66.8)	71.3 (69.6 to 73.0)	67.8 (66.3 to 69.1)	4.8 (2.4 to 7.1)	9.2 (6.7 to 11.6)	6.8 (4.9 to 8.5)
Delhi	63.5 (61.5 to 65.2)	65.7 (63.7 to 67.7)	64.4 (63.0 to 65.9)	70.8 (68.8 to 72.8)	74.7 (72.8 to 76.7)	72.6 (71.2 to 74.2)	7.3 (4.7 to 10.1)	9.1 (6.4 to 11.9)	8.1 (6.3 to 10.2)
Telangana	60.2 (58.3 to 62.2)	61.8 (59.8 to 63.9)	61.0 (59.4 to 62.7)	69.5 (67.5 to 71.5)	73.2 (71.2 to 75.2)	71.3 (69.5 to 73.1)	9.2 (6.6 to 11.8)	11.4 (8.6 to 14.0)	10.3 (8.0 to 12.4)
Andhra Pradesh	57.8 (56.0 to 59.6)	58.5 (56.5 to 60.3)	58.1 (56.5 to 59.6)	67.4 (65.5 to 69.0)	71.9 (70.0 to 73.7)	69.6 (67.9 to 71.0)	9.6 (7.0 to 11.9)	13.4 (10.8 to 16.0)	11.5 (9.1 to 13.4)
Jammu and Kashmir	60.3 (58.5 to 62.0)	61.0 (59.1 to 62.9)	60.5 (59.2 to 61.8)	68.4 (66.4 to 70.2)	71.8 (69.8 to 73.7)	69.9 (68.5 to 71.3)	8.1 (5.8 to 10.5)	10.9 (8.4 to 13.4)	9.4 (7.6 to 11.2)
Karnataka	59.0 (57.4 to 60.7)	62.6 (60.6 to 64.2)	60.7 (59.4 to 61.8)	67.1 (65.3 to 69.0)	71.1 (69.4 to 72.8)	69.1 (67.7 to 70.4)	8.1 (5.7 to 10.7)	8.6 (6.1 to 10.9)	8.4 (6.4 to 10.0)
West Bengal	58.4 (56.9 to 60.0)	59.6 (58.1 to 61.3)	59.0 (57.8 to 60.2)	68.2 (66.4 to 69.8)	71.4 (69.7 to 73.0)	69.7 (68.5 to 70.9)	9.8 (7.4 to 11.9)	11.7 (9.5 to 14.0)	10.7 (9.1 to 12.3)
Maharashtra	59.7 (58.3 to 61.2)	63.0 (61.5 to 64.5)	61.3 (60.2 to 62.5)	68.6 (66.8 to 70.4)	73.0 (71.5 to 74.6)	70.7 (69.4 to 71.9)	8.9 (6.4 to 11.0)	10.0 (7.8 to 12.0)	9.4 (7.7 to 11.0)
Union Territories other than Delhi	66.8 (65.0 to 68.6)	68.7 (67.2 to 70.5)	67.7 (66.1 to 69.3)	71.0 (68.8 to 73.1)	76.0 (73.7 to 77.9)	73.3 (71.3 to 75.2)	4.3 (1.8 to 6.9)	7.3 (4.7 to 9.6)	5.6 (3.3 to 7.8)
**High ETL group**	**61.9 (60.6 to 63.3)**	**65.9 (64.6 to 67.3)**	**63.8 (62.9 to 64.8)**	**70.5 (69.2 to 71.8)**	**75.4 (74.2 to 76.5)**	**72.9 (72.0 to 73.8)**	**8.6 (6.6 to 10.4)**	**9.4 (7.6 to 11.2)**	**9.0 (7.8 to 10.4)**
Himachal Pradesh	64.3 (62.4 to 65.9)	65.4 (63.7 to 67.3)	64.8 (63.5 to 66.1)	71.0 (69.2 to 72.9)	76.9 (75.3 to 77.7)	73.8 (72.6 to 75.0)	6.8 (4.3 to 9.3)	11.5 (9.1 to 13.7)	9.0 (7.4 to 10.8)
Punjab	62.2 (60.6 to 63.9)	64.2 (62.4 to 65.8)	63.1 (62.0 to 64.4)	68.0 (66.3 to 69.8)	72.3 (70.4 to 74.2)	70.0 (68.6 to 71.4)	5.8 (3.3 to 8.2)	8.1 (5.6 to 10.6)	6.9 (5.0 to 8.7)
Tamil Nadu	59.4 (57.7 to 61.1)	62.0 (60.3 to 63.7)	60.6 (59.4 to 61.8)	69.0 (67.3 to 70.6)	73.6 (72.0 to 75.2)	71.2 (69.9 to 72.4)	9.6 (7.1 to 11.8)	11.6 (9.2 to 14.0)	10.6 (8.8 to 12.3)
Goa	66.1 (64.4 to 67.9)	69.3 (67.6 to 71.1)	67.7 (66.2 to 69.4)	73.0 (71.1 to 74.6)	78.4 (77.0 to 78.8)	75.7 (74.1 to 76.7)	6.9 (4.6 to 9.2)	9.1 (7.0 to 10.9)	8.0 (5.9 to 9.8)
Kerala	67.6 (65.9 to 69.0)	74.5 (73.1 to 76.1)	71.0 (69.9 to 71.9)	73.8 (72.5 to 75.1)	78.7 (78.0 to 79.0)	76.4 (75.5 to 77.1)	6.2 (4.2 to 8.3)	4.1 (2.6 to 5.7)	5.4 (4.1 to 6.6)

Figure 1: Association between epidemiological transition ratio and socio-demographic index for the states of India, 1990 and 2016

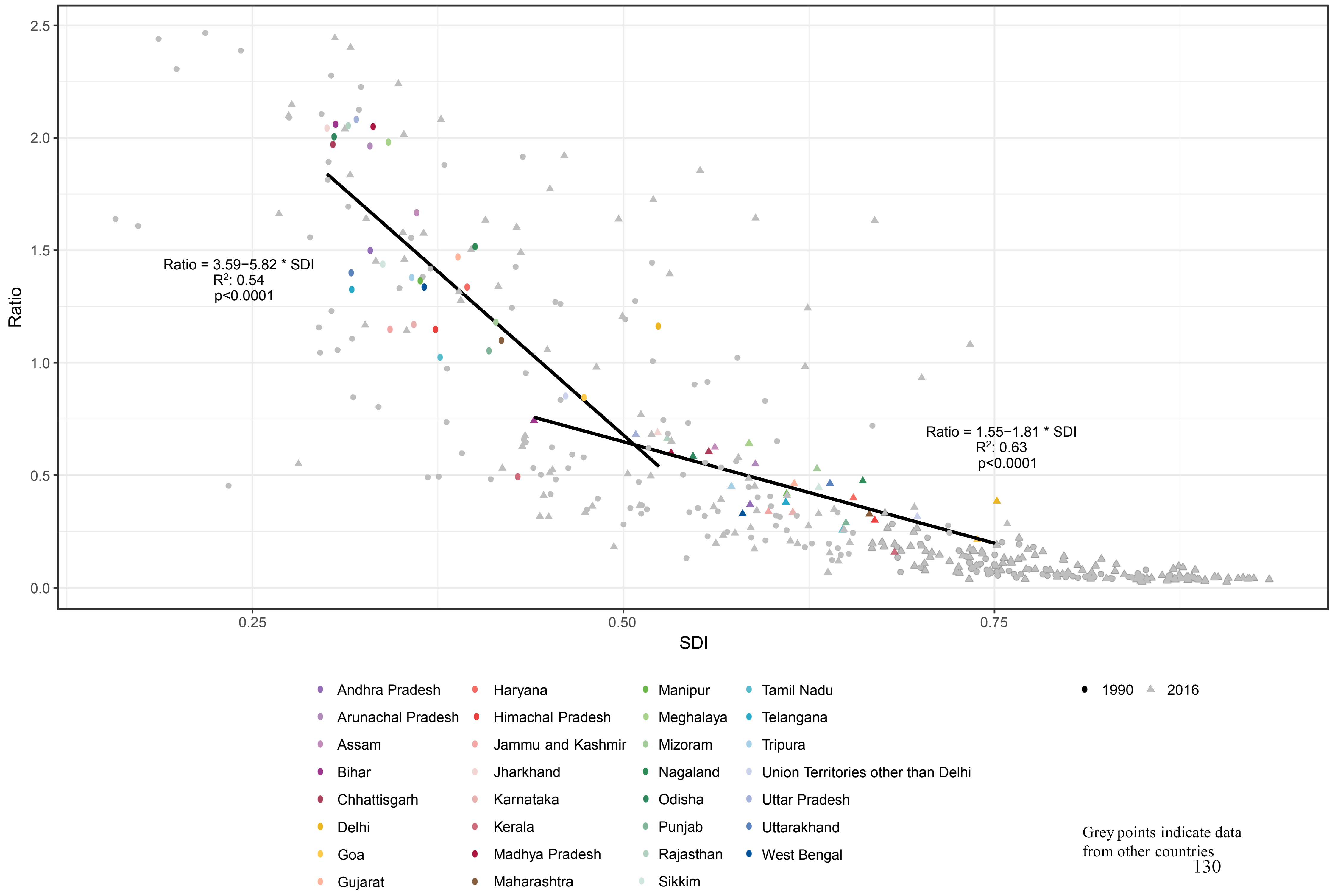

Grey points indicate data from other countries

Figure 2: Change in death number and percent change in rates for the top 30 causes from 1990 to 2016, India

Causes are connected by arrows between time periods. Communicable maternal, neonatal and nutritional disorders are shown in red, non-communicable causes in blue and injuries in green. For the time period 1990 to 2016, three measures of change are shown: percent change in the number of deaths, percent change in the all-ages death rate and percent change in the age-standardized death rate. COPD = Chronic Obstructive Pulmonary Disease.

Leading causes 1990	Leading causes 2016	Mean % change number of Deaths 1990-2016	Mean % change all-ages Death rate 1990-2016	Mean % change age-standardized Death rate 1990-2016
1 Diarrheal diseases	1 Ischemic heart disease	135.4% (119.6 to 153.5%)	54.5% (44.1 to 66.4%)	12.0% (4.5 to 21.3%)
2 Ischemic heart disease	2 COPD	35.9% (19.2 to 63.3%)	-10.8% (-21.7 to 7.2%)	-40.2% (-47.4 to -28.2%)
3 Lower respiratory infect	3 Diarrheal diseases	-38.5% (-51.0 to -22.1%)	-59.6% (-67.8 to -48.9%)	-60.1% (-67.7 to -46.7%)
4 Tuberculosis	4 Cerebrovascular disease	65.0% (49.8 to 81.4%)	8.3% (-1.7 to 19.0%)	-23.7% (-31.2 to -15.3%)
5 COPD	5 Lower respiratory infect	-32.6% (-44.6 to -19.4%)	-55.7% (-63.6 to -47.1%)	-35.6% (-44.6 to -24.4%)
6 Cerebrovascular disease	6 Tuberculosis	-38.5% (-46.8 to -32.1%)	-59.7% (-65.1 to -55.5%)	-68.7% (-73.6 to -65.0%)
7 Neonatal preterm birth	7 Diabetes	251.7% (221.7 to 281.5%)	130.8% (111.1 to 150.4%)	63.7% (48.1 to 79.1%)
8 Measles	8 Road injuries	80.4% (67.3 to 93.6%)	18.4% (9.8 to 27.1%)	8.2% (0.4 to 15.9%)
9 Other neonatal	9 Chronic kidney disease	102.2% (80.3 to 127.5%)	32.7% (18.4 to 49.3%)	1.7% (-10.2 to 14.4%)
10 Asthma	10 Self-harm	40.1% (25.8 to 65.1%)	-8.1% (-17.5 to 8.4%)	-15.2% (-23.7 to -0.3%)
11 Neonatal encephalopathy	11 Falls	87.3% (52.7 to 116.8%)	22.9% (0.2 to 42.3%)	-0.4% (-18.3 to 15.9%)
12 Self-harm	12 Asthma	-10.4% (-31.8 to 13.5%)	-41.2% (-55.3 to -25.5%)	-56.5% (-67.9 to -43.7%)
13 Road injuries	13 Neonatal preterm birth	-55.6% (-62.8 to -47.9%)	-70.9% (-75.6 to -65.8%)	-48.3% (-56.7 to -39.3%)
14 Tetanus	14 Alzheimer disease	232.6% (201.1 to 265.7%)	118.3% (97.6 to 140.0%)	23.4% (10.2 to 36.5%)
15 Intestinal infectious	15 Hypertensive heart disease	89.2% (62.0 to 134.2%)	24.2% (6.3 to 53.7%)	-11.2% (-24.5 to 8.1%)
16 Congenital defects	16 Other neonatal	-50.6% (-61.0 to -36.8%)	-67.6% (-74.4 to -58.5%)	-42.5% (-54.5 to -26.3%)
17 Chronic kidney disease	17 Rheumatic heart disease	22.8% (0.7 to 49.0%)	-19.4% (-33.9 to -2.2%)	-33.3% (-46.0 to -17.1%)
18 Falls	18 HIV/AIDS	1,011.8% (926.7 to 1,101.4%)	629.7% (573.9 to 688.6%)	523.9% (476.5 to 575.3%)
19 Meningitis	19 Congenital defects	-27.0% (-52.9 to 11.5%)	-52.1% (-69.1 to -26.8%)	-23.4% (-50.7 to 16.1%)
20 Rheumatic heart disease	20 Intestinal infectious	-32.0% (-43.0 to -21.2%)	-55.4% (-62.6 to -48.3%)	-47.5% (-55.0 to -40.7%)
21 Diabetes	21 Neonatal encephalopathy	-57.5% (-66.4 to -47.1%)	-72.1% (-77.9 to -65.3%)	-50.4% (-60.8 to -38.3%)
22 Peptic ulcer disease	22 Stomach cancer	50.4% (37.4 to 67.7%)	-1.3% (-9.8 to 10.1%)	-26.6% (-33.3 to -18.0%)
23 Drowning	23 Other neoplasms	377.8% (128.1 to 470.9%)	213.6% (49.7 to 274.7%)	144.4% (15.0 to 202.2%)
24 Malaria	24 Hepatitis	12.8% (0.3 to 26.3%)	-25.9% (-34.2 to -17.1%)	-20.6% (-28.3 to -13.1%)
25 Hypertensive heart disease	25 Lung cancer	154.2% (122.7 to 178.8%)	66.8% (46.1 to 83.0%)	20.9% (5.9 to 32.6%)
26 Encephalitis	26 Urinary diseases	111.1% (75.5 to 129.8%)	38.5% (15.2 to 50.8%)	13.9% (-4.2 to 24.5%)
27 Hepatitis	27 Cirrhosis hepatitis B	90.9% (68.7 to 117.2%)	25.3% (10.7 to 42.6%)	-6.1% (-16.8 to 7.3%)
28 Protein-energy malnutrition	28 Ileus & obstruction	113.3% (79.5 to 172.8%)	40.0% (17.8 to 79.0%)	25.9% (0.3 to 63.2%)
29 Neonatal hemolytic	29 Meningitis	-37.5% (-53.2 to 1.7%)	-59.0% (-69.3 to -33.3%)	-51.7% (-63.4 to -25.3%)
30 Animal contact	30 Drowning	-20.2% (-32.6 to 0.3%)	-47.7% (-55.7 to -34.2%)	-38.6% (-47.1 to -26.7%)
33 Stomach cancer	31 Encephalitis			
35 Alzheimer disease	36 Animal contact			
37 Cirrhosis hepatitis B	38 Peptic ulcer disease			
39 Urinary diseases	41 Malaria			
40 Ileus & obstruction	57 Protein-energy malnutrition			
48 Lung cancer	69 Measles			
66 Other neoplasms	75 Neonatal hemolytic			
80 HIV/AIDS	95 Tetanus			

Legend:

Communicable, maternal, neonatal and nutritional

Non-communicable

Injuries

Figure 3: Change in DALYs number and percent change in rates for the top 30 causes from 1990 to 2016, men and women, India

Causes are connected by arrows between time periods. Communicable maternal, neonatal and nutritional disorders are shown in red, non-communicable causes in blue and injuries in green. For the time period 1990 to 2016, three measures of change are shown: percent change in the number of DALYs, percent change in the all-ages DALY rate and percent change in the age-standardized DALY rate. COPD = Chronic Obstructive Pulmonary Disease.

Men

Leading causes 1990
1 Diarrheal diseases
2 Lower respiratory infect
3 Neonatal preterm birth
4 Tuberculosis
5 Ischemic heart disease
6 Measles
7 Other neonatal
8 COPD
9 Neonatal encephalopathy
10 Road injuries
11 Congenital defects
12 Cerebrovascular disease
13 Tetanus
14 Intestinal infectious
15 Self-harm
16 Iron-deficiency anemia
17 Asthma
18 Meningitis
19 Sense organ diseases
20 Drowning
21 Falls
22 Neonatal hemolytic
23 Skin diseases
24 Protein-energy malnutrition
25 Chronic kidney disease
26 Low back & neck pain
27 Malaria
28 Neonatal sepsis
29 Animal contact
30 Hepatitis
31 Migraine
33 Other musculoskeletal
34 Diabetes
36 Depressive disorders
39 Interpersonal violence
42 Alcohol use disorders
48 Cirrhosis hepatitis B
90 HIV/AIDS

Leading causes 2016	Mean % change number of DALYs 1990-2016	Mean % change all-ages DALY rate 1990-2016	Mean % change age-standardized DALY rate 1990-2016
1 Ischemic heart disease	**112.2% (95.4 to 130.5%)**	**39.1% (28.1 to 51.1%)**	8.1% (-0.4 to 17.8%)
2 COPD	**36.7% (23.1 to 54.0%)**	-10.4% (-19.3 to 0.9%)	**-34.7% (-41.0 to -26.1%)**
3 Road injuries	**73.9% (59.4 to 87.9%)**	**14.0% (4.5 to 23.2%)**	**8.5% (0.1 to 16.6%)**
4 Lower respiratory infect	**-60.9% (-67.6 to -52.1%)**	**-74.4% (-78.8 to -68.6%)**	**-57.9% (-64.8 to -49.6%)**
5 Diarrheal diseases	**-65.4% (-73.3 to -54.4%)**	**-77.3% (-82.5 to -70.1%)**	**-69.5% (-76.2 to -61.8%)**
6 Tuberculosis	**-40.9% (-49.2 to -32.2%)**	**-61.2% (-66.7 to -55.5%)**	**-67.6% (-72.7 to -62.8%)**
7 Cerebrovascular disease	**60.3% (40.9 to 78.4%)**	5.1% (-7.6 to 17.0%)	**-20.8% (-30.0 to -11.6%)**
8 Neonatal preterm birth	**-47.1% (-58.2 to -35.9%)**	**-65.3% (-72.6 to -57.9%)**	**-41.8% (-54.1 to -29.7%)**
9 Self-harm	**54.7% (20.6 to 84.6%)**	1.4% (-20.9 to 21.1%)	-7.3% (-27.8 to 10.7%)
10 Sense organ diseases	**81.0% (78.3 to 84.0%)**	**18.7% (16.9 to 20.6%)**	**-4.8% (-5.9 to -3.8%)**
11 Diabetes	**180.7% (166.5 to 195.8%)**	**84.0% (74.7 to 93.9%)**	**45.0% (37.0 to 53.2%)**
12 Iron-deficiency anemia	**30.8% (27.7 to 34.1%)**	**-14.2% (-16.3 to -12.1%)**	0.6% (-1.4 to 2.3%)
13 Other neonatal	**-54.1% (-65.6 to -40.5%)**	**-69.9% (-77.5 to -61.0%)**	**-47.0% (-60.4 to -31.3%)**
14 Low back & neck pain	**79.7% (75.9 to 83.2%)**	**17.8% (15.3 to 20.1%)**	**-3.0% (-4.1 to -1.8%)**
15 Falls	**45.2% (18.8 to 66.2%)**	-4.8% (-22.1 to 8.9%)	-8.9% (-22.8 to 2.0%)
16 Chronic kidney disease	**76.2% (57.2 to 102.8%)**	**15.5% (3.1 to 33.0%)**	-5.3% (-15.7 to 9.3%)
17 Skin diseases	**57.0% (50.9 to 62.9%)**	2.9% (-1.1 to 6.8%)	**5.8% (1.0 to 10.6%)**
18 Congenital defects	-28.2% (-57.0 to 10.1%)	**-53.0% (-71.8 to -27.8%)**	-28.3% (-56.5 to 7.7%)
19 Neonatal encephalopathy	**-57.4% (-70.6 to -43.9%)**	**-72.1% (-80.7 to -63.2%)**	**-51.1% (-66.2 to -35.7%)**
20 Migraine	**69.4% (66.7 to 71.9%)**	**11.0% (9.3 to 12.7%)**	-0.3% (-1.6 to 0.8%)
21 Other musculoskeletal	**73.5% (68.4 to 78.6%)**	**13.7% (10.4 to 17.1%)**	**-4.3% (-6.3 to -2.4%)**
22 Depressive disorders	**71.4% (66.3 to 76.7%)**	**12.4% (9.0 to 15.9%)**	**-3.9% (-6.2 to -1.5%)**
23 HIV/AIDS	**890.5% (803.4 to 979.4%)**	**549.3% (492.2 to 607.6%)**	**502.9% (450.8 to 556.7%)**
24 Intestinal infectious	**-34.8% (-45.5 to -24.5%)**	**-57.3% (-64.3 to -50.5%)**	**-48.5% (-56.4 to -41.2%)**
25 Asthma	-19.7% (-35.1 to 1.0%)	**-47.3% (-57.5 to -33.8%)**	**-55.1% (-65.9 to -42.2%)**
26 Drowning	**-31.4% (-43.9 to -17.5%)**	**-55.0% (-63.2 to -45.9%)**	**-45.9% (-54.7 to -36.5%)**
27 Alcohol use disorders	**66.7% (47.8 to 83.3%)**	9.3% (-3.1 to 20.2%)	-6.0% (-16.8 to 3.0%)
28 Interpersonal violence	**33.0% (6.8 to 60.8%)**	-12.8% (-30.0 to 5.4%)	**-18.5% (-34.2 to -0.8%)**
29 Meningitis	**-44.2% (-58.7 to -12.9%)**	**-63.4% (-72.9 to -42.9%)**	**-52.3% (-64.6 to -28.7%)**
30 Cirrhosis hepatitis B	**104.5% (75.3 to 137.0%)**	**34.1% (14.9 to 55.3%)**	7.4% (-7.4 to 25.0%)
31 Animal contact			
33 Hepatitis			
37 Protein-energy malnutrition			
47 Malaria			
48 Neonatal sepsis			
61 Neonatal hemolytic			
62 Measles			
100 Tetanus			

Legend:

Communicable, maternal, neonatal and nutritional

Non-communicable

Injuries

Figure 3: Change in DALYs number and percent change in rates for the top 30 causes from 1990 to 2016, men and women, India

Causes are connected by arrows between time periods. Communicable maternal, neonatal and nutritional disorders are shown in red, non-communicable causes in blue and injuries in green. For the time period 1990 to 2016, three measures of change are shown: percent change in the number of deaths, percent change in the all-ages death rate and percent change in the age-standardized death rate. COPD = Chronic Obstructive Pulmonary Disease.

Women

Leading causes 1990	Leading causes 2016	Mean % change number of DALYs 1990-2016	Mean % change all-age DALY rate 1990-2016	Mean % change age-standardized DALY rate 1990-2016
1 Diarrheal diseases	1 Ischemic heart disease	**91.5% (65.1 to 118.7%)**	**25.8% (8.5 to 43.8%)**	-5.5% (-17.6 to 7.9%)
2 Lower respiratory infect	2 Diarrheal diseases	**-69.5% (-76.7 to -57.9%)**	**-80.0% (-84.7 to -72.3%)**	**-73.0% (-77.9 to -65.3%)**
3 Neonatal preterm birth	3 Iron-deficiency anemia	**48.2% (46.2 to 50.1%)**	**-2.6% (-3.9 to -1.3%)**	-0.2% (-1.2 to 0.7%)
4 Measles	4 Lower respiratory infect	**-61.7% (-69.9 to -50.8%)**	**-74.9% (-80.2 to -67.6%)**	**-59.8% (-67.9 to -49.0%)**
5 Tuberculosis	5 COPD	**37.2% (13.8 to 82.9%)**	-9.9% (-25.2 to 20.2%)	**-36.3% (-47.3 to -14.2%)**
6 Other neonatal	6 Cerebrovascular disease	**45.3% (25.0 to 68.8%)**	-4.5% (-17.9 to 10.9%)	**-30.4% (-40.2 to -18.9%)**
7 Ischemic heart disease	7 Neonatal preterm birth	**-44.9% (-57.0 to -30.7%)**	**-63.8% (-71.7 to -54.5%)**	**-38.3% (-51.7 to -22.5%)**
8 Iron-deficiency anemia	8 Sense organ diseases	**89.6% (87.2 to 92.2%)**	**24.6% (23.0 to 26.3%)**	**-4.2% (-5.2 to -3.2%)**
9 COPD	9 Migraine	**69.0% (66.8 to 71.3%)**	**11.1% (9.6 to 12.6%)**	-1.0% (-2.1 to 0.1%)
10 Neonatal encephalopathy	10 Low back & neck pain	**56.6% (51.9 to 61.0%)**	2.9% (-0.2 to 5.8%)	**-17.6% (-19.3 to -15.9%)**
11 Congenital defects	11 Tuberculosis	**-49.7% (-55.0 to -42.9%)**	**-67.0% (-70.4 to -62.5%)**	**-71.4% (-75.0 to -66.6%)**
12 Cerebrovascular disease	12 Self-harm	9.3% (-13.4 to 36.7%)	**-28.2% (-43.1 to -10.2%)**	**-30.1% (-44.3 to -12.6%)**
13 Self-harm	13 Diabetes	**167.1% (147.9 to 185.7%)**	**75.5% (62.9 to 87.8%)**	**34.1% (23.1 to 44.6%)**
14 Intestinal infectious	14 Skin diseases	**53.2% (48.7 to 58.0%)**	0.7% (-2.3 to 3.9%)	**5.0% (2.4 to 7.7%)**
15 Tetanus	15 Other neonatal	**-43.3% (-60.0 to -22.5%)**	**-62.7% (-73.7 to -49.1%)**	**-33.4% (-53.1 to -9.0%)**
16 Low back & neck pain	16 Congenital defects	-7.7% (-49.3 to 27.0%)	**-39.4% (-66.7 to -16.6%)**	-6.6% (-48.2 to 27.7%)
17 Sense organ diseases	17 Depressive disorders	**60.7% (55.7 to 65.8%)**	**5.6% (2.3 to 9.0%)**	**-10.9% (-13.3 to -8.7%)**
18 Meningitis	18 Other musculoskeletal	**85.5% (80.0 to 91.1%)**	**21.9% (18.3 to 25.6%)**	1.4% (-0.9 to 3.9%)
19 Migraine	19 Falls	**37.5% (9.7 to 58.5%)**	-9.6% (-27.9 to 4.1%)	**-16.4% (-30.0 to -6.3%)**
20 Protein-energy malnutrition	20 Chronic kidney disease	**64.2% (45.9 to 83.1%)**	7.9% (-4.1 to 20.3%)	**-11.3% (-21.1 to -1.1%)**
21 Asthma	21 Neonatal encephalopathy	**-53.6% (-65.0 to -39.8%)**	**-69.5% (-77.0 to -60.5%)**	**-45.8% (-59.1 to -29.9%)**
22 Malaria	22 Asthma	-8.6% (-34.4 to 23.7%)	**-39.9% (-56.9 to -18.7%)**	**-50.7% (-66.5 to -31.3%)**
23 Skin diseases	23 Road injuries	**36.2% (19.8 to 51.4%)**	**-10.5% (-21.3 to -0.5%)**	**-10.2% (-20.1 to -1.2%)**
24 Falls	24 Intestinal infectious	**-39.5% (-52.0 to -27.6%)**	**-60.3% (-68.5 to -52.4%)**	**-50.5% (-60.2 to -41.5%)**
25 Maternal hemorrhage	25 Anxiety disorders	**61.6% (55.8 to 67.6%)**	**6.2% (2.4 to 10.2%)**	**-4.2% (-7.5 to -0.8%)**
26 Depressive disorders	26 Rheumatic heart disease	6.7% (-22.4 to 37.1%)	**-29.9% (-49.0 to -9.9%)**	**-37.4% (-55.4 to -17.5%)**
27 Whooping cough	27 Protein-energy malnutrition	**-43.4% (-58.2 to -27.5%)**	**-62.8% (-72.5 to -52.4%)**	**-41.1% (-56.1 to -24.5%)**
28 Neonatal sepsis	28 Gynecological diseases	**62.7% (58.8 to 66.4%)**	**6.9% (4.4 to 9.4%)**	**-10.6% (-13.2 to -8.1%)**
29 Other musculoskeletal	29 Breast cancer	**118.1% (55.0 to 177.8%)**	**43.3% (1.9 to 82.6%)**	9.0% (-22.1 to 36.4%)
30 Encephalitis	30 Malaria	-31.0% (-72.8 to 22.6%)	**-54.6% (-82.1 to -19.4%)**	-35.5% (-74.0 to 9.5%)
31 Rheumatic heart disease	31 Meningitis			
34 Road injuries	32 HIV/AIDS			
35 Chronic kidney disease	34 Encephalitis			
36 Diabetes	35 Neonatal sepsis			
40 Anxiety disorders	48 Maternal hemorrhage			
45 Gynecological diseases	54 Measles			
52 Breast cancer	81 Whooping cough			
112 HIV/AIDS	111 Tetanus			

Legend:
Communicable, maternal, neonatal and nutritional
Non-communicable
Injuries

Figure 4: Age-standardised DALY rates of the top 30 causes in the states of India, 2016

	Ischemic heart disease	COPD	Diarrheal diseases	Lower respiratory infect	Cerebrovascular disease	Sense organ diseases	Neonatal preterm birth	Tuberculosis	Iron-deficiency anemia	Diabetes	Road injuries	Low back & neck pain	Falls	Self-harm	Other neonatal	Chronic kidney disease	Migraine	Congenital defects	Skin diseases	Depressive disorders	Other musculoskeletal	Neonatal encephalopathy	Asthma	Intestinal infectious	HIV/AIDS	Rheumatic heart disease	Hypertensive heart disease	Anxiety disorders	Alzheimer disease	Meningitis
**India**	**4111**	**2432**	**2163**	**1868**	**1694**	**1315**	**1308**	**1282**	**1231**	**1059**	**1018**	**909**	**839**	**836**	**834**	**728**	**723**	**685**	**674**	**627**	**626**	**600**	**566**	**390**	**362**	**326**	**312**	**311**	**304**	**303**
**Low ETL Group**	**3474**	**3043**	**3164**	**2415**	**1715**	**1359**	**1329**	**1866**	**1338**	**957**	**1088**	**895**	**855**	**691**	**1028**	**629**	**720**	**731**	**675**	**567**	**627**	**614**	**735**	**518**	**336**	**405**	**343**	**305**	**309**	**342**
Bihar	3939	2507	3582	2187	1632	1388	1030	1128	1498	972	863	908	800	398	894	645	716	979	634	521	622	758	611	324	453	505	387	313	298	275
Jharkhand	3593	1865	4961	1939	1449	1327	1241	1697	1384	965	1116	911	758	477	910	697	716	599	660	582	626	688	473	295	450	376	314	337	288	266
Uttar Pradesh	3560	3912	3152	2556	1142	1367	1363	2546	1270	990	1258	912	981	768	1078	560	717	786	682	543	622	557	950	607	194	394	345	306	312	434
Rajasthan	3417	4208	1959	2782	1180	1356	1476	1737	1363	642	1228	823	676	604	1041	490	736	480	660	549	633	475	961	735	288	322	268	342	315	315
Meghalaya	1926	1629	2169	2039	1644	1299	1001	1857	1163	921	644	915	679	417	476	730	725	538	672	715	646	712	442	522	184	290	283	316	357	311
Assam	2479	2806	3225	2530	3451	1311	1639	1838	1450	1187	918	811	634	797	1051	842	719	672	690	629	663	905	693	336	344	424	411	314	310	260
Chhattisgarh	3222	1822	3117	2399	3069	1453	1742	1612	1191	1075	939	916	859	953	1217	688	725	537	706	517	627	723	440	483	393	412	354	251	296	254
Madhya Pradesh	4125	2452	2479	2351	1920	1336	1535	1563	1307	1044	1040	913	798	872	1186	582	719	682	684	557	621	418	600	637	376	383	343	249	309	355
Odisha	2162	1540	3926	1866	2860	1314	1058	1522	1253	893	913	912	999	724	857	921	721	512	715	767	631	755	388	328	594	418	332	315	318	244
**Lower-middle ETL Group**	**4526**	**2551**	**1347**	**1698**	**1292**	**1286**	**1427**	**1525**	**1154**	**1003**	**977**	**919**	**860**	**775**	**718**	**677**	**721**	**697**	**673**	**572**	**619**	**679**	**572**	**336**	**446**	**288**	**266**	**298**	**310**	**332**
Arunachal Pradesh	1896	1751	1967	1610	1551	1284	1015	1348	843	1033	796	907	779	799	544	666	715	460	677	741	622	458	410	207	577	220	264	314	351	237
Mizoram	1063	2808	1408	1647	741	1280	922	806	664	803	932	920	778	314	636	674	727	489	675	529	621	485	528	713	573	118	194	317	353	274
Nagaland	2330	1354	919	1812	2051	1259	940	1102	520	822	793	909	716	235	394	713	717	400	683	603	620	499	385	455	772	256	300	315	337	212
Uttarakhand	3618	3383	1394	2579	1064	1261	1305	1488	1009	1042	1491	921	888	610	824	639	727	661	717	548	612	611	695	580	413	304	292	317	327	408
Gujarat	5042	2495	1274	1530	1147	1289	1495	1640	1246	990	916	919	909	794	739	666	720	751	668	570	619	728	565	297	426	288	257	288	304	345
Tripura	3188	2470	1951	2227	3126	1303	1517	626	1214	972	804	912	484	1399	634	806	721	521	661	551	640	500	661	180	270	348	269	315	300	212
Sikkim	2710	1834	788	1895	890	1243	809	875	930	931	751	907	1005	582	448	680	709	335	645	589	599	283	432	352	199	207	264	312	330	215
Manipur	2318	1590	1997	1677	2217	1294	963	1282	462	1410	1149	921	422	602	425	843	728	388	659	657	635	474	313	379	967	274	387	411	336	196
**Higher-middle ETL Group**	**4497**	**2112**	**1430**	**1303**	**1987**	**1287**	**1336**	**796**	**1161**	**1018**	**941**	**918**	**793**	**978**	**604**	**760**	**724**	**613**	**675**	**685**	**634**	**586**	**468**	**251**	**409**	**283**	**274**	**318**	**297**	**258**
Haryana	5661	3103	1512	1670	1142	1246	1072	1348	1309	1116	1448	915	752	724	892	737	714	567	680	756	623	511	627	497	302	280	301	313	317	315
Delhi	4118	1520	709	1183	929	1195	1143	968	959	1295	813	831	738	346	973	741	714	551	684	508	619	626	254	419	208	295	225	313	275	269
Telangana	3982	1911	1915	1048	1334	1292	1445	639	1090	899	906	919	910	1010	386	575	725	576	660	788	592	588	451	298	658	266	279	316	314	224
Andhra Pradesh	4857	2086	1779	1256	1531	1284	1443	662	1334	1038	1027	919	946	1127	422	672	723	691	655	793	611	731	509	364	530	315	311	315	325	261
Jammu and Kashmir	4689	3064	1160	1834	1349	1287	1063	710	943	835	1618	915	693	427	728	898	717	650	645	547	615	442	541	131	161	276	272	314	291	239
Karnataka	4805	2256	1652	1012	1681	1355	1526	785	1143	1529	911	920	847	1369	605	789	741	852	671	623	619	817	523	173	437	313	259	316	296	298
West Bengal	4195	1892	1348	1550	3586	1310	1220	710	1196	756	823	916	612	1081	635	837	723	505	687	583	629	458	452	142	396	310	268	328	287	245
Maharashtra	4403	2082	1207	1244	1648	1254	1377	842	1116	939	875	932	823	833	590	766	721	559	680	749	677	520	434	249	361	236	264	315	291	240
Union Territories other than Delhi	3531	1293	658	901	988	1226	1081	803	1039	1115	836	914	1113	763	311	936	712	585	683	665	570	496	292	155	322	283	559	313	307	281
**High ETL Group**	**4797**	**1443**	**950**	**889**	**1093**	**1269**	**927**	**596**	**1038**	**1479**	**1000**	**924**	**871**	**983**	**337**	**958**	**727**	**631**	**671**	**695**	**602**	**478**	**312**	**213**	**268**	**211**	**334**	**320**	**303**	**206**
Himachal Pradesh	2780	2640	924	1160	762	1236	992	698	847	570	872	926	910	572	641	562	728	533	676	586	594	610	499	271	307	184	215	318	305	188
Punjab	6646	1617	1149	1237	1166	1237	908	698	1141	1526	1357	924	685	427	690	947	719	628	668	502	628	594	326	456	349	245	243	285	298	255
Tamil Nadu	5225	1431	1165	955	1059	1307	1016	764	1260	1825	1046	918	1083	1303	259	1086	724	640	670	820	598	539	313	202	239	233	411	297	286	220
Goa	3265	1228	485	760	1182	1192	640	345	687	1190	705	930	811	516	387	641	728	453	691	585	585	296	262	23	381	101	176	259	283	121
Kerala	3190	1159	461	460	1136	1231	748	209	557	1033	657	934	629	869	147	811	740	643	676	622	593	226	273	17	248	153	289	394	329	145

Lower than national mean
Indistinguishable from national mean
Higher than national mean

Figure 5: Range of all-ages DALY rates across the states of India for the top 10 causes, 2016

The number in each vertical column is the ratio of the highest to lowest rate across the states

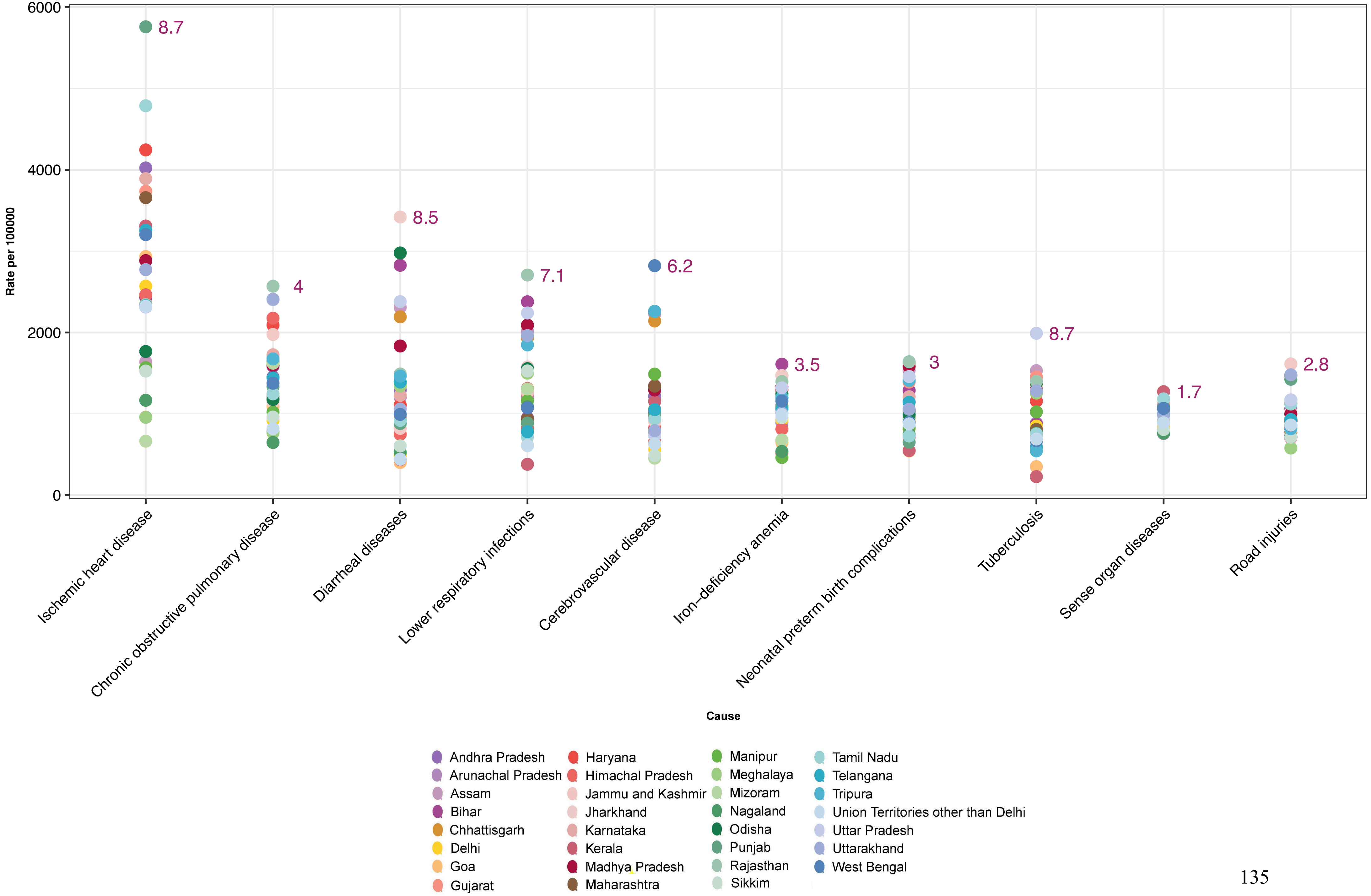

Figure 6: Percent of total DALYs by age group in India, 1990

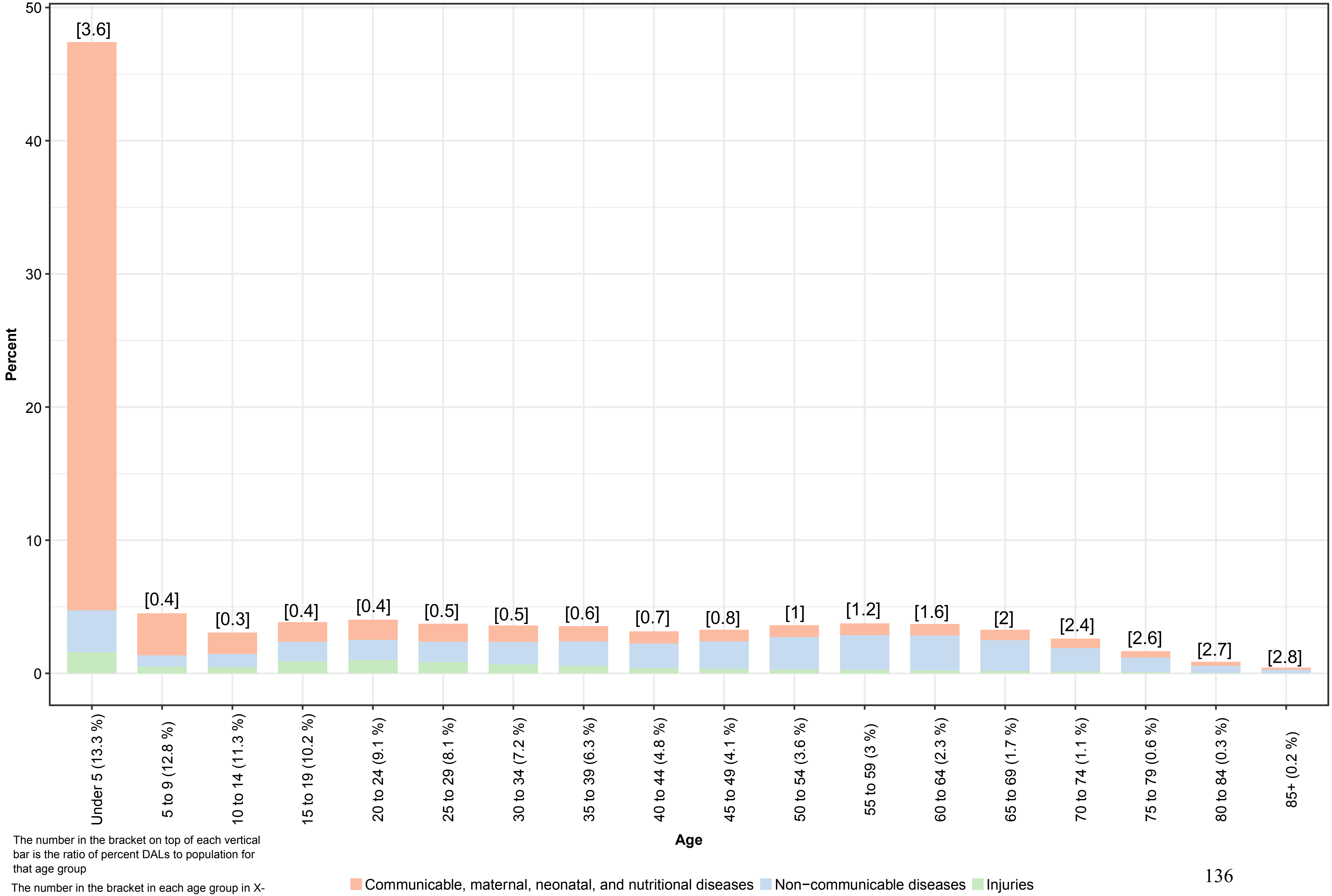

The number in the bracket on top of each vertical bar is the ratio of percent DALs to population for that age group

The number in the bracket in each age group in X-axis is the percent of population in that age group

Figure 7: Contribution of YLLs and YLDs to DALYs in states grouped by epidemiological transition level, 1990 and 2016

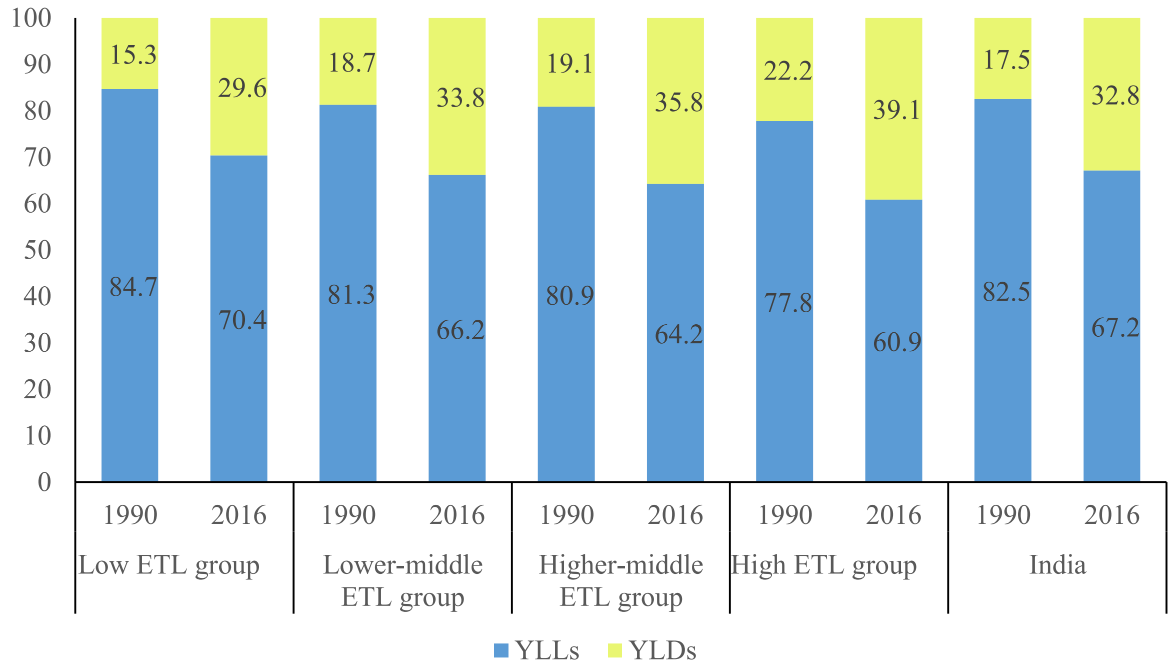

Figure 8: Comparison of top 30 causes of DALYs, YLLs and YLDs in India, 2016

Leading causes DALYs	Leading causes YLDs	Leading causes YLLs
Ischemic heart disease	Iron-deficiency anemia	Ischemic heart disease
COPD	Sense organ diseases	Lower respiratory infect
Diarrheal diseases	Low back & neck pain	Diarrheal diseases
Lower respiratory infect	Migraine	COPD
Cerebrovascular disease	Skin diseases	Cerebrovascular disease
Iron-deficiency anemia	Depressive disorders	Tuberculosis
Neonatal preterm birth	Other musculoskeletal	Neonatal preterm birth
Tuberculosis	COPD	Self-harm
Sense organ diseases	Diabetes	Road injuries
Road injuries	Anxiety disorders	Other neonatal
Self-harm	Oral disorders	Congenital defects
Low back & neck pain	Falls	Neonatal encephalopathy
Diabetes	Neonatal preterm birth	Chronic kidney disease
Other neonatal	Osteoarthritis	Diabetes
Migraine	Schizophrenia	Intestinal infectious
Skin diseases	Diarrheal diseases	Falls
Falls	Gynecological diseases	HIV/AIDS
Congenital defects	Drug use disorders	Asthma
Other musculoskeletal	Hemoglobinopathies	Meningitis
Chronic kidney disease	Road injuries	Drowning
Depressive disorders	Protein-energy malnutrition	Rheumatic heart disease
Neonatal encephalopathy	Other mental & substance	Malaria
Asthma	Autistic spectrum	Hepatitis
Intestinal infectious	Congenital defects	Hypertensive heart disease
HIV/AIDS	Alcohol use disorders	Encephalitis
Anxiety disorders	Asthma	Animal contact
Meningitis	Tension headache	Other neoplasms
Rheumatic heart disease	Chronic kidney disease	Neonatal sepsis
Protein-energy malnutrition	Intellectual disability	Interpersonal violence
Drowning	Bipolar disorder	Ileus & obstruction

Figure 9: Percent contribution of top 10 risk factors to DALYs in states grouped by epidemiological transition level in 2016

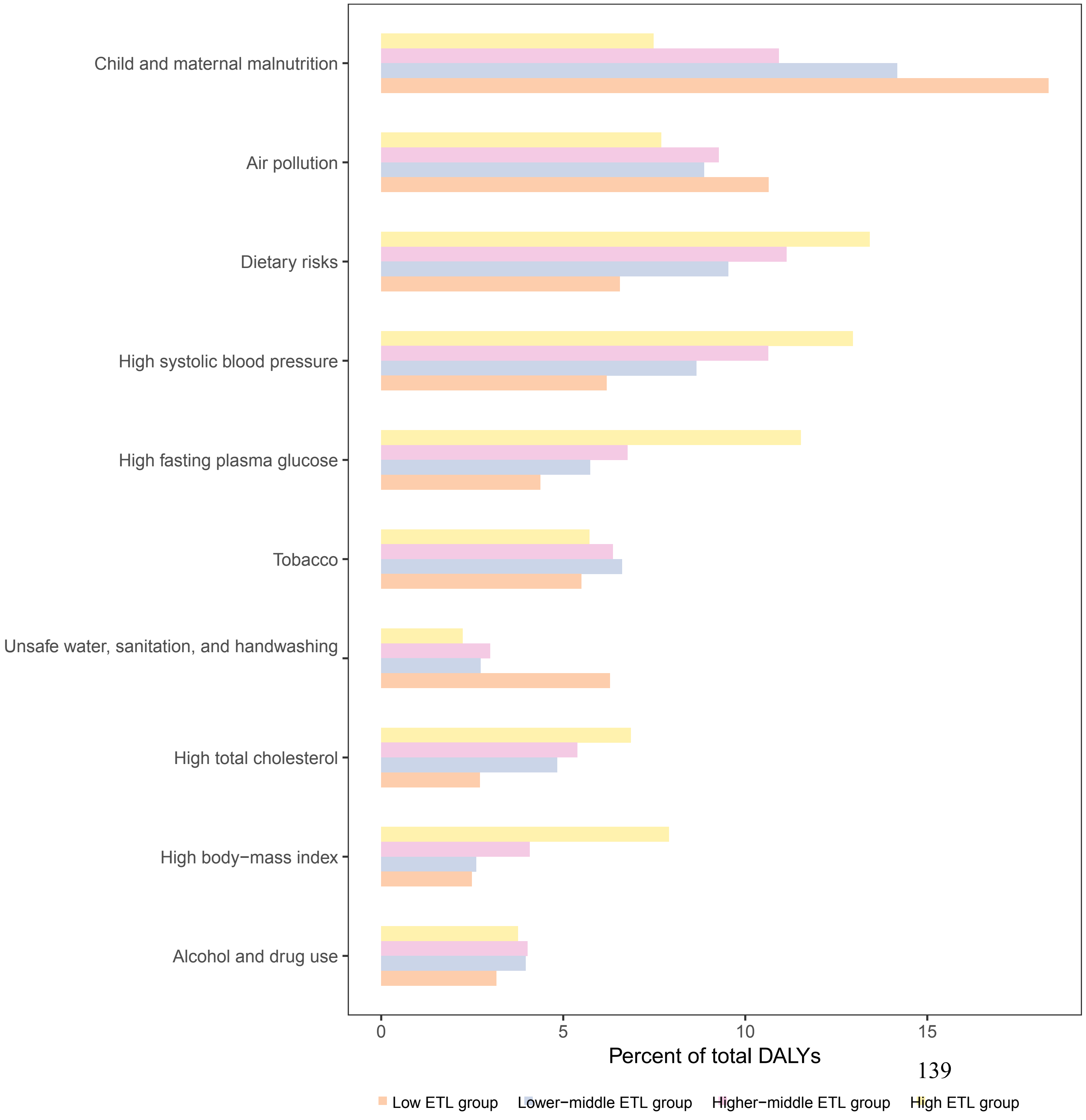

Figure 10: Age-standardised DALY rates attributable to risk factors in the states of India, 2016

	Child and maternal malnutrition	Air pollution	Dietary risks	High systolic blood pressure	High fasting plasma glucose	Tobacco	Unsafe water, sanitation, and handwashing	High total cholesterol	High body-mass index	Alcohol and drug use	Impaired kidney function	Occupational risks	Low physical activity	Other environmental risks	Unsafe sex	Low bone mineral density	Sexual abuse and violence
**India**	**5633**	**4528**	**4077**	**4021**	**2860**	**2651**	**2127**	**1813**	**1524**	**1358**	**1276**	**1241**	**480**	**466**	**406**	**346**	**110**
**Low ETL Group**	**6559**	**5594**	**3592**	**3528**	**2504**	**2952**	**3164**	**1424**	**1292**	**1442**	**1099**	**1364**	**400**	**488**	**395**	**348**	**108**
Bihar	6514	5785	3969	3750	2270	2570	3615	1475	804	1069	1135	1312	422	549	499	313	119
Jharkhand	6420	4471	3564	3710	2428	1472	4798	1137	1163	1058	1148	1186	385	454	490	318	113
Uttar Pradesh	6731	6263	3374	3104	2485	3578	3170	1331	1430	1612	979	1533	376	519	296	391	98
Rajasthan	6679	6135	3247	3049	1923	3205	2004	1792	1323	1435	890	1506	337	348	343	294	89
Meghalaya	4960	3116	2653	2902	2155	3373	2057	852	835	1828	1067	1037	260	341	230	253	85
Assam	7392	5021	3911	4531	2914	3348	3164	1007	1910	1779	1405	1273	340	492	358	253	110
Chhattisgarh	6779	4908	4032	4226	2935	2285	3128	1646	1439	1295	1287	1147	426	519	448	357	109
Madhya Pradesh	6470	5101	4001	3954	3106	2974	2498	1820	1214	1478	1131	1286	567	486	440	337	109
Odisha	5282	3912	3221	3675	2490	1871	3813	936	1389	1533	1419	1034	333	464	540	407	151
**Lower-middle ETL Group**	**5275**	**4043**	**4239**	**3987**	**2719**	**2929**	**1240**	**2031**	**1084**	**1455**	**1200**	**1243**	**485**	**400**	**452**	**348**	**85**
Arunachal Pradesh	4120	2594	2413	2753	2226	2526	1778	703	875	1352	992	1027	260	317	569	329	143
Mizoram	3433	2441	1601	1561	1644	5177	1279	436	707	1368	843	1159	212	234	575	325	91
Nagaland	3588	2628	2879	3804	2102	1987	886	1180	827	1731	1152	951	321	328	632	278	79
Uttarakhand	5950	4393	3322	3513	2594	4051	1278	1697	2080	1405	1061	1361	546	401	442	386	99
Gujarat	5397	4095	4577	4116	2739	2668	1161	2239	934	1464	1217	1240	499	402	438	362	80
Tripura	5559	4769	4018	4588	3197	3616	1947	1478	1013	1325	1417	1277	425	437	293	204	94
Sikkim	3395	2552	2454	3189	2175	2443	618	1185	1495	1295	1033	1127	308	331	235	353	76
Manipur	3086	2643	3180	3459	3015	3405	1882	1180	1182	1612	1275	1051	333	432	852	226	112
**Higher-middle ETL Group**	**4602**	**3890**	**4470**	**4436**	**2849**	**2538**	**1351**	**2083**	**1506**	**1341**	**1395**	**1176**	**506**	**444**	**437**	**331**	**115**
Haryana	5211	5271	4623	4428	3000	3950	1335	2806	2103	1811	1363	1404	561	388	316	341	116
Delhi	4505	2856	3318	3379	3611	2285	542	1889	2041	1305	1189	1087	437	369	285	302	77
Telangana	4479	3354	3680	3365	2330	1882	1830	1802	1577	947	1086	1117	422	450	579	389	121
Andhra Pradesh	4936	3674	4662	4461	2765	2434	1702	2290	1997	1113	1263	1158	719	486	507	397	136
Jammu and Kashmir	4374	4392	4421	4350	2392	4290	1114	2041	2062	1145	1483	1339	468	357	213	303	71
Karnataka	4664	3529	4013	4566	3724	2282	1545	2160	938	1465	1397	1247	519	569	513	342	108
West Bengal	4619	4842	5279	5411	2500	3410	1380	2058	1162	1250	1620	1177	424	409	406	254	108
Maharashtra	4363	3426	4375	4066	2767	1833	1090	1958	1594	1486	1382	1119	502	419	426	341	123
Union Territories other than Delhi	3486	1981	3109	3649	3017	1834	565	1592	2360	1400	1380	931	384	305	340	462	99
**High ETL Group**	**3237**	**2633**	**4422**	**4403**	**3948**	**1852**	**870**	**2166**	**2466**	**1103**	**1527**	**1040**	**638**	**482**	**331**	**376**	**112**
Himachal Pradesh	3756	2774	2472	2779	1494	2073	846	1338	1029	1121	881	1180	286	279	353	320	65
Punjab	4224	4115	5529	5960	3973	1842	1038	2374	3224	1188	1701	1258	662	380	388	313	81
Goa	2338	1613	2680	3204	2662	864	329	1465	1873	1187	1071	875	375	252	328	331	80
Tamil Nadu	3538	2630	5100	4499	4812	1930	1096	2408	2662	1183	1685	1025	737	646	332	474	148
Kerala	1722	1634	2873	3496	2895	1698	387	1736	1854	879	1251	902	515	307	281	274	72

Lower than national mean
Indistinguishable from national mean
Higher than national mean
